# Estimated prebiotic intake and depression in US adults: repeated cross-sectional analyses of NHANES 2015–2018 and 2021–2023

**DOI:** 10.1017/jns.2026.10136

**Published:** 2026-07-31

**Authors:** Cassandra Boyd, John Gieng

**Affiliations:** Department of Nutrition, Food Science, and Packaging, San José State University, USA

**Keywords:** Depression, NHANES, Nutritional epidemiology, Prebiotic intake

## Abstract

Prebiotics are selectively utilised dietary substrates that may influence depression through gut microbiota-related pathways, but population-level estimates of intake and associations with depression are limited. This repeated cross-sectional study estimated intake of International Scientific Association for Probiotics and Prebiotics (ISAPP)-recognised prebiotics and examined associations with depression among US adults using National Health and Nutrition Examination Survey (NHANES) 2015–2018 and NHANES August 2021–August 2023, analysed as independent survey periods. Estimated intakes of galactooligosaccharides and inulin-type fructans were assigned to foods using published source-food values, Food and Nutrient Database for Dietary Studies food codes, and ingredient-level information for mixed dishes. Depression was defined as Patient Health Questionnaire-9 score of 10 or greater. Survey-weighted analyses estimated intake distributions, and logistic regression models estimated ORs and 95% CIs for depression per 5 g/day higher estimated prebiotic intake in restricted adult samples. Mean estimated prebiotic intake was 6.44 g/day in NHANES 2015–2018 and 6.09 g/day in NHANES 2021–2023. In NHANES 2015–2018, higher estimated intake was associated with lower odds of depression overall (OR: 0.69; 95% CI: 0.49–0.98) and among females (OR: 0.65; 95% CI: 0.45–0.94), but not among males. In NHANES 2021–2023, estimated intake was not associated with depression overall (OR: 1.03; 95% CI: 0.74–1.43) or in sex-stratified models. These repeated cross-sectional findings provide nationally representative estimates of intake of ISAPP-recognised prebiotics and indicate that the inverse association observed in 2015–2018 was not replicated in 2021–2023. Prospective studies are needed to clarify whether prebiotic intake is associated with depression risk.

## Introduction

Depression is a major public health concern in the United States, with evidence of increasing prevalence over time and persistent differences across population subgroups.^(1,2)^ Depression is also socially patterned, including by education and other socio-economic indicators, which may overlap with dietary behaviours, food access, and other health-related exposures.^(3)^ Although depression is multifactorial and cannot be explained by diet alone, diet is a modifiable exposure that can influence gut microbial composition and metabolism. Growing evidence suggests that major depressive disorder is associated with alterations in gut microbiota composition, supporting interest in the gut–brain axis as one potential pathway through which diet-related exposures may influence depressive symptoms.^(4)^

Prebiotics are selectively utilised dietary substrates that confer a health benefit through the gut microbiota.^(5)^ Because the definition and scope of prebiotics continue to evolve, the present study focused on International Scientific Association for Probiotics and Prebiotics (ISAPP)-recognised prebiotics relevant to the US diet, primarily galactooligosaccharides (GOS) and inulin-type fructans, including inulin, fructooligosaccharides (FOS), and oligofructose.^(5,6)^ These compounds are present in foods commonly consumed in the United States, including cereals and grains, fruits, vegetables, pulses, and legumes.^(5,7,8)^ However, intake depends not only on whether these foods are consumed, but also on portion size, food preparation, mixed-dish ingredients, and frequency of consumption.

Estimated effective doses of prebiotics have been reported to range from 5 g/day to 15 g/day for adults, although this range should be interpreted as a physiological benchmark rather than a diagnostic or dietary cutoff.^(9)^ This benchmark is useful for evaluating whether estimated habitual intake approaches quantities that may be biologically relevant. Through their effects on gut microbial metabolism, prebiotics may plausibly influence depressive symptoms through pathways involving short-chain fatty acid production, immune-inflammatory signalling, gut barrier function, and neuroendocrine regulation.^(10)^ These mechanisms provide a rationale for studying prebiotic intake in relation to depressive symptoms, but they do not establish that higher prebiotic intake reduces depression risk in free-living populations.

Human evidence linking prebiotics with depression remains limited and inconsistent. Randomised trials of prebiotics and synbiotics have reported mixed effects on mood-related outcomes,^(11,12)^ and systematic reviews of prebiotics, probiotics, synbiotics, and depression have emphasised heterogeneity in interventions, populations, doses, durations, and outcome measures.^(13,14)^ More broadly, higher fibre intake and greater adherence to fibre-rich dietary patterns have been associated with lower depressive symptom burden in observational studies, although intervention findings remain mixed.^(15)^ These inconsistencies may reflect differences between supplement-based interventions and habitual food intake, variation in baseline diet and gut microbiota, differences in depression assessment, and limited power in some studies.

Population-level estimates of prebiotic intake in the United States also remain limited. Prior US work has estimated intake of selected components, including inulin and oligofructose^(16)^ and resistant starch,^(17)^ but fewer analyses have estimated intake of multiple ISAPP-recognised prebiotic classes across food-code-level dietary data. Estimating intake from the National Health and Nutrition Examination Survey (NHANES) requires linkage between dietary recalls, Food and Nutrient Database for Dietary Studies (FNDDS) food codes, and published prebiotic composition values. This is particularly important for mixed dishes, where prebiotic-containing ingredients may contribute to intake even when the reported food is not itself a single prebiotic-rich item.

Accordingly, this study aimed to identify commonly consumed foods containing ISAPP-recognised prebiotics, estimate their intake among US adults, and examine associations between estimated prebiotic intake and depression using repeated cross-sectional data from NHANES 2015–2018 and NHANES August 2021–August 2023, analysed as independent survey periods. We hypothesised that higher estimated prebiotic intake would be associated with lower odds of depression.

## Methods

### Study design

This repeated cross-sectional study analysed publicly available data from NHANES 2015–2016, 2017–2018, and August 2021–August 2023. NHANES is a repeated cross-sectional survey conducted by the Centers for Disease Control and Prevention that uses a complex, multistage probability sampling design to obtain nationally representative samples of the noninstitutionalized US population.^(18–23)^ NHANES data are collected through interviews and standardised health examinations. NHANES protocols were approved by the National Center for Health Statistics Research Ethics Review Board, and all participants provided written informed consent. The present secondary analysis used publicly available, de-identified data and was therefore exempt from additional Institutional Review Board oversight.

NHANES 2015–2016 and 2017–2018 were combined and analysed as the 2015–2018 survey period. NHANES August 2021–August 2023 was analysed as a separate survey period because 2019–2020 field operations were suspended during the COVID-19 pandemic and the subsequent cycle differed in timing, survey context, sample design, questionnaires, and dietary recall procedures.^(20–25)^ The two survey periods were not pooled because the objective was to present period-specific estimates and associations rather than a continuous trend analysis. Participants in each survey period represent independent cross-sectional samples; therefore, cross-period comparisons describe population-level differences between survey periods and do not represent within-person change over time. NHANES survey methods and analytic guidance were followed for all survey-weighted analyses.^(23)^

NHANES dietary recall data were merged with the USDA FNDDS, which provides standardised food codes, gram amounts, food descriptions, nutrient values, and related food-composition information for reported dietary items.^(24)^ For NHANES 2021–2023, the corresponding FNDDS/What We Eat in America documentation was used to support food-code assignment and interpretation of 24-hour dietary recall files.^(25)^ This linkage allowed estimated prebiotic values to be assigned to reported foods and used to calculate participant-level estimated prebiotic intake. Because this was a secondary analysis of fixed NHANES samples, a formal a priori sample size calculation was not performed. Sample adequacy was assessed based on the precision of the primary estimates and the size of the analytic samples used for descriptive and regression analyses.

### Analytic samples

For NHANES 2015–2018, the descriptive analytic sample included adults aged 18 years or older with at least one reliable 24-hour dietary recall, complete Patient Health Questionnaire-9 (PHQ-9) data, valid dietary sample weights, and complete covariate data needed for descriptive analyses. The restricted regression sample was used to reduce heterogeneity in the diet–depression association and excluded participants with BMI outside 18–30 kg/m^2^, excessive alcohol intake, reported depression medication use, or missing total energy intake, sleep, or employment data.

For NHANES 2021–2023, the descriptive analytic sample was constructed using comparable eligibility criteria: adults aged 18 years or older with at least one reliable dietary recall, complete PHQ-9 data, valid dietary sample weights, and required demographic and covariate data. The restricted regression sample excluded participants with BMI outside 18–30 kg/m^2^, excessive alcohol intake, missing total energy intake, missing sleep adequacy, or incomplete covariate data required for the regression model. Depression medication exclusion could not be replicated in the same way in 2021–2023 because public prescription medication files did not provide the detailed medication-name or indication information needed to identify depression medication use comparably with 2015–2018. Therefore, medication use was not used as an exclusion criterion in the primary 2021–2023 restricted sample. A conservative sensitivity analysis excluding participants reporting any prescription medication use was conducted for 2021–2023.

### Assessment of prebiotic intake

Prebiotic intake was estimated from foods and beverages only; supplements and medications were excluded. A literature review was conducted to identify ISAPP-recognised prebiotic-containing foods and to estimate their content of GOS and inulin-type fructans, including inulin, FOS, and oligofructose. When multiple published estimates were available, we prioritised the closest food-specific match to the FNDDS item; US-specific values were used when available, although such data were limited for many foods. These prebiotics were summed for each food to determine estimated total prebiotic content. Source-food values were derived from published food-composition literature and are summarised in Supplementary Table 1.^(7,8,26–34)^

Because of gaps and variability in the literature, several assumptions were required. Cooked and raw forms were assumed to have the same prebiotic content unless source-specific information suggested otherwise, midpoint values were used when prebiotic content was reported as a range, and comparable food subtypes within FNDDS were assigned the same estimated value when food-specific values were unavailable. Frozen foods were assumed to have comparable prebiotic content as fresh versions when food-specific frozen values were unavailable.^(33,34)^ For FNDDS mixed dishes with ingredient-level information, prebiotic content was estimated by summing the amounts contributed by component ingredients. Foods containing less than 0.5 g total estimated prebiotics per 100 g were excluded from source-food assignment.

To assign estimated prebiotic values to FNDDS foods, FNDDS food descriptions and ingredient information were matched to comparable foods listed in Supplementary Table 1. For mixed dishes, prebiotic content was estimated by summing the amounts contributed by component ingredients. The resulting FNDDS food-code lookup values were compiled separately for NHANES 2015–2018 and NHANES 2021–2023 because FNDDS food codes and ingredient structures may differ across survey periods. For NHANES 2021–2023, food-code assignments were developed using the corresponding 2021–2023 FNDDS foods and ingredient information. When a food code with a positive 2015–2018 assignment was also present in the 2021–2023 FNDDS files, the 2015–2018 assigned value was carried forward to maintain consistency across periods; 2021–2023-specific values were assigned for positive foods not represented by the 2015–2018 positive lookup. Moisture-adjusted source-food values were used when needed to align source-food values with reported food forms. The period-specific FNDDS food-code lookup values are provided in Supplementary Table 2.

Participant-level intake was estimated by multiplying the estimated prebiotic content of each reported food by the gram amount consumed, summing across all foods reported by each participant, and converting to g/day. Day 1 dietary data were collected during the Mobile Examination Center visit for NHANES 2015–2018, with Day 2 collected by telephone 3–10 days later. In NHANES 2021–2023, both Day 1 and Day 2 dietary recalls were collected by telephone according to cycle-specific dietary procedures.^(25)^ When both reliable recall days were available, intakes were averaged; when only one reliable recall day was available, that single day was used to estimate participant intake.

### Depression status and covariates

Depression was defined using the Patient Health Questionnaire-9 (PHQ-9), a nine-item screening instrument assessing depressive symptoms over the previous two weeks. Each item is scored from 0 to 3, yielding a total score from 0 to 27. Participants with scores of 0–9 were classified as not having depression, and participants with scores of 10 or greater were classified as having depression. This threshold was used to identify clinically relevant depressive symptoms.

Covariates were selected a priori based on prior literature and plausibility as factors related to dietary intake and depression. Covariates included age, sex, race/ethnicity, education, poverty ratio, employment status, sleep adequacy, total energy intake, BMI, alcohol intake, and, for NHANES 2015–2018, reported depression medication use. Sleep adequacy was based on self-reported sleep duration or cycle-specific sleep adequacy variables. Excessive alcohol intake was defined using sex-specific daily alcohol intake limits. Total energy intake was included to account for overall intake volume and improve interpretation of dietary composition.

### Statistical analysis

Analyses were performed using IBM SPSS Statistics, version 29.0 (IBM Corp., Armonk, NY, USA). Survey-weighted analyses accounted for NHANES sample weights, strata, and primary sampling units. Dietary sample weights were used because estimated prebiotic intake was derived from 24-hour dietary recall data. For NHANES 2015–2018, the four-year dietary weight was used. For NHANES 2021–2023, the cycle-specific dietary weight was used. All weighting decisions followed NHANES analytic guidance and weighting tutorials.^(23,43)^

Continuous variables were presented as weighted means and SEs, and categorical variables were presented as weighted percentages. For descriptive analyses of prebiotic intake, values were summarised both as absolute intake in g/day and as energy-adjusted intake in g/1000 kcal/day. Energy-adjusted prebiotic intake was calculated as estimated prebiotic intake divided by total energy intake and multiplied by 1000. Differences between groups were evaluated using survey-weighted general linear models for continuous variables and survey-weighted χ^2^ tests for categorical variables when applicable.

Survey-weighted logistic regression was used to evaluate the association between estimated prebiotic intake and depression in the restricted regression samples. In the main models, estimated prebiotic intake was modelled per 5 g/day higher intake. This 5 g/day scaling value was selected a priori because prior literature reports an estimated adult prebiotic effective-dose range of 5–15 g/day; therefore, it was used as a lower-bound reference value rather than a diagnostic, dietary, or analytic cutoff.^(9)^ The overall 2015–2018 model was adjusted for age, sex, race/ethnicity, education, poverty ratio, employment status, sleep adequacy, and total energy intake. Sex-stratified models were adjusted for age, race/ethnicity, education, poverty ratio, employment status, sleep adequacy, and total energy intake. The same model structure was applied to NHANES 2021–2023 except that depression medication exclusion could not be replicated in the primary analysis. Sensitivity analyses were conducted to evaluate the robustness of the regression findings. For both survey periods, sensitivity analyses included a simplified covariate model, a two-day-only dietary recall sample, and an energy-adjusted exposure model using estimated prebiotic intake in g/1000 kcal. For NHANES 2021–2023, an additional conservative sensitivity analysis excluded participants reporting any prescription medication use because depression medication use could not be identified comparably with NHANES 2015–2018. Adjusted ORs and 95% CIs were reported. Analyses were conducted on complete cases, with no imputation for missing data. Statistical significance was set at P less than or equal to 0.05.

## Results

### Participant selection

**Figure 1.**
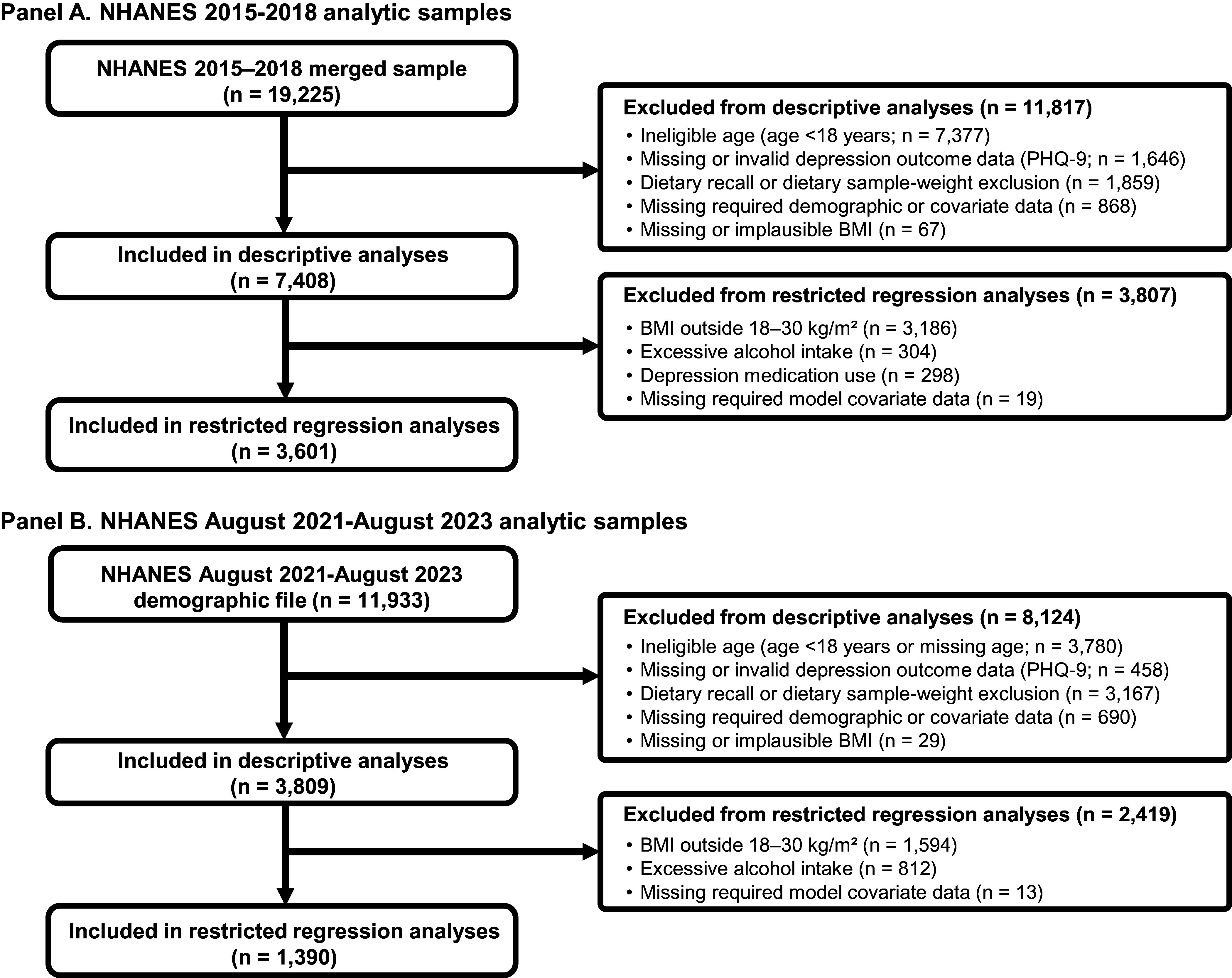
Figure 1 long description. Flow diagrams of participant selection for repeated cross-sectional analyses of (A) NHANES 2015–2018 and (B) NHANES August 2021–August 2023 analytic samples. Values are unweighted participant counts. In Panel A, dietary recall or dietary sample-weight exclusions include no reliable dietary recall and missing or nonpositive four-year dietary sample weight. In Panel B, dietary recall or dietary sample-weight exclusions include no reliable dietary recall and missing or nonpositive dietary sample weight. Missing required demographic or covariate data include period-specific covariates required for the descriptive analytic sample. Missing required model covariate data include covariates required for the restricted regression model. Depression medication use was not applied as an exclusion criterion in NHANES August 2021–August 2023 because comparable public medication-name or indication data were unavailable.

For NHANES 2015–2018, 19,225 participants were available in the merged files used for sample construction. The descriptive analytic sample included 7,408 adults. Participants were excluded from the descriptive sample because of age less than 18 years (*n* = 7,377), missing or invalid PHQ-9 data (*n* = 1,646), no valid dietary recall (*n* = 491), missing or nonpositive four-year dietary sample weight (*n* = 1,368), missing education data (*n* = 3), missing depression medication data (*n* = 32), missing poverty ratio (*n* = 833), or missing or implausible BMI (*n* = 67). From the descriptive sample, 3,807 participants were excluded from the restricted regression sample because of BMI outside 18–30 kg/m^2^ (*n* = 3,186), excessive alcohol intake (*n* = 304), depression medication use (*n* = 298), missing sleep data (*n* = 14), or missing employment status (*n* = 5), resulting in a restricted regression sample of 3,601 participants. Participant selection is shown in Figure 1A.

For NHANES 2021–2023, 11,933 participants were available in the demographic file. The descriptive analytic sample included 3,809 adults. Participants were excluded because of age less than 18 years or missing age (*n* = 3,780), missing or invalid PHQ-9 data (*n* = 458), no reliable dietary recall or missing/nonpositive dietary sample weight (*n* = 3,167), missing sex, race/ethnicity, or education data (*n* = 659), missing or invalid alcohol classification (*n* = 31), or missing or implausible BMI (*n* = 29). From the descriptive sample, 2,419 participants were excluded from the restricted regression sample because of BMI outside 18–30 kg/m^2^ (*n* = 1,594), excessive alcohol intake (*n* = 812), missing total energy intake (*n* = 2), or missing sleep adequacy (*n* = 11), resulting in a restricted regression sample of 1,390 participants. Participant selection is shown in Figure 1B.

### Participant characteristics

In NHANES 2015–2018, the descriptive analytic sample included 7,408 adults and represented approximately 214 million US adults after application of survey weights. The weighted prevalence of depression was 8.3% (Table 1). In NHANES 2021–2023, the descriptive analytic sample included 3,809 adults and represented approximately 218 million US adults after application of survey weights. The weighted prevalence of depression was 11.4%. The restricted regression samples included 3,601 adults in NHANES 2015–2018 and 1,390 adults in NHANES 2021–2023. Characteristics of the restricted regression samples by depression status are shown in Table 4. Sample-comparison analyses for the descriptive and restricted samples are shown in Supplementary Tables 3 and 4.

**Table 1.** Demographic characteristics of US adults in the descriptive analytic samples, NHANES 2015–2018 and 2021–2023 Table 1 long description.

Characteristic	2015–2018 value	SE	*n*	2021–2023 value	SE	*n*
Age, years	47.2	0.5	7,408	46.6	0.6	3,809
**Sex**						
Male	48.7		3,558	49.1		1,681
Female	51.3		3,850	50.9		2,128
**Depression status**						
Not depressed	91.7		6,777	88.6		3,361
Depressed	8.3		631	11.4		448
Poverty ratio	3.05	0.06	7,408	3.16	0.11	3,809
**Race/ethnicity**						
Mexican American	8.8		1,093	6.7		225
Other Hispanic	5.9		770	9.1		346
Non-Hispanic White	65.2		2,783	63.3		2,446
Non-Hispanic Black	10.4		1,636	10.3		398
Non-Hispanic Asian	5.4		778	5.5		163
Other race/multiracial	4.3		348	5.1		231
**Education**						
Did not graduate high school	10.9		1,347	7.5		348
High school/GED	25.2		1,818	23.2		731
Some college/AA	32.2		2,378	30.8		1,181
College graduate or above	31.8		1,865	38.5		1,549
BMI, kg/m^2^	29.6	0.2	7,408	29.8	0.3	3,809
**BMI category**						
Underweight	1.4		106	1.1		45
Normal	25.8		1,865	24.6		942
Overweight	30.8		2,284	33.4		1,238
Obese	42.1		3,153	40.9		1,584

Abbreviations: NHANES, National Health and Nutrition Examination Survey; GED, general educational development; AA, associate degree. Values are weighted means and SEs for continuous variables and weighted percentages for categorical variables; n values are unweighted counts. Depression was defined as a Patient Health Questionnaire-9 score of 10 or greater. The descriptive analytic samples included adults aged 18 years or older with at least one reliable 24-hour dietary recall, complete depression outcome data, valid dietary sample weights, and complete demographic and covariate data required for descriptive analyses. Analyses accounted for the complex NHANES survey design, including dietary sample weights, strata, and primary sampling units. NHANES 2015–2018 and NHANES 2021–2023 were analysed as separate survey periods; no formal cross-period statistical tests are shown.

### Food sources and FNDDS prebiotic assignments

Supplementary Table 1 identified 31 unique source foods, represented by multiple food-form-specific rows where applicable, containing ISAPP-recognised prebiotics. In summary, five fruits, two grains, six legumes, one sweetener, and seventeen vegetables contained 0.5 g/100 g or more total estimated prebiotics. The source-food values in Supplementary Table 1 were used to assign estimated prebiotic values to FNDDS food codes and mixed-dish ingredients.

Supplementary Table 2 provides period-specific FNDDS food-code estimated prebiotic values for NHANES 2015–2018 and NHANES 2021–2023. For NHANES 2015–2018, 3,191 of 8,689 FNDDS food codes (36.7%) had a positive estimated prebiotic value. Most positive values were low to moderate in concentration: 2,991 food codes (34.4% of all food codes) contained 1.0–49.9 mg/g, 33 food codes (0.4%) contained 50.0–99.9 mg/g, and 13 food codes (0.1%) contained 100.0 mg/g or more. In addition, 154 food codes (1.8%) contained less than 1.0 mg/g. For NHANES 2021–2023, 2,007 of 5,431 FNDDS food codes (37.0%) had a positive estimated prebiotic value. Of these, 1,866 food codes (34.4% of all food codes) contained 1.0–49.9 mg/g, 7 food codes (0.1%) contained 50.0–99.9 mg/g, and 10 food codes (0.2%) contained 100.0 mg/g or more. In addition, 124 food codes (2.3%) contained less than 1.0 mg/g. Because many FNDDS foods are variations of the same underlying foods, similar items were consolidated to identify the foods with the highest estimated prebiotic concentrations (Table 2).

**Table 2.** FNDDS foods with the highest estimated ISAPP-recognised prebiotic content Table 2 long description.

Food	Total prebiotic in food, mg/g food	Food portion containing 5 g prebiotics, oz food
Dandelion greens	155.5–243.0	0.7–1.1
Jerusalem artichoke	209.7	0.8
Garlic	191.5–192.6	0.9
Leek	123.0–127.7	1.4
Onions	78.9–106.1	1.7–2.2
Onion rings	57.8–58.1	3.0–3.1
Peas and onions	54.5–55.9	3.2
Creamed onions	50.8	3.5
Cowpeas	50.2–50.4	3.5
Asparagus	50.0	3.5

Abbreviations: FNDDS, Food and Nutrient Database for Dietary Studies; ISAPP, International Scientific Association for Probiotics and Prebiotics. Values are expressed as estimated total ISAPP-recognised prebiotic content in mg/g food and the estimated food portion in ounces required to provide 5 g prebiotics. Ranges reflect variation across food codes, food forms, preparations, or survey-period-specific FNDDS assignments where applicable. Source-food composition references are provided in Supplementary Table 1, and period-specific FNDDS food-code assignments are provided in Supplementary Table 2.

### Estimated prebiotic intake

In NHANES 2015–2018, mean estimated intake of ISAPP-recognised prebiotics was 6.44 g/day (SE 0.08; 95% CI 6.28–6.60) in the descriptive analytic sample (Table 3). Energy-adjusted estimated intake was 3.14 g/1000 kcal (SE 0.03; 95% CI 3.07–3.20). In NHANES 2021–2023, mean estimated prebiotic intake was 6.09 g/day (SE 0.17; 95% CI 5.71–6.46) in the descriptive analytic sample. Energy-adjusted estimated intake was 2.93 g/1000 kcal (SE 0.04; 95% CI 2.85–3.02). Thus, average estimated intake was modestly lower in 2021–2023 in both absolute and energy-adjusted terms, although estimates were generally similar across periods. Detailed subgroup estimates of estimated prebiotic intake are provided in Supplementary Table 5.

**Table 3.** Estimated ISAPP-recognised prebiotic intake among US adults, NHANES 2015–2018 and 2021–2023 Table 3 long description.

Survey Period	*n*	g/day	SE	95% CI	g/1000 kcal/day	SE	95% CI
2015–2018	7,408	6.44	0.08	6.28–6.60	3.14	0.03	3.07–3.20
2021–2023	3,809	6.09	0.17	5.71–6.46	2.93	0.04	2.85–3.02

Abbreviations: NHANES, National Health and Nutrition Examination Survey; ISAPP, International Scientific Association for Probiotics and Prebiotics. Values are presented as unweighted sample size (n), weighted mean estimated prebiotic intake, SE, and 95% CI. Estimated prebiotic intake reflects the sum of estimated ISAPP-recognised prebiotics from foods and beverages reported in one or two reliable 24-hour dietary recalls. Energy-adjusted estimated prebiotic intake was calculated as estimated prebiotic intake divided by total energy intake and multiplied by 1000. The descriptive analytic samples included adults aged 18 years or older with at least one reliable 24-hour dietary recall, complete depression outcome data, valid dietary sample weights, and complete demographic and covariate data required for descriptive analyses. Analyses accounted for the complex NHANES survey design, including dietary sample weights, strata, and primary sampling units. NHANES 2015–2018 and NHANES 2021–2023 were analysed as separate survey periods; no formal cross-period statistical tests are shown.

In the restricted regression samples, mean estimated prebiotic intake was lower among participants with depression than among participants without depression in both survey periods (Table 4). In NHANES 2015–2018, mean estimated prebiotic intake was 6.56 g/day overall, 6.61 g/day among participants without depression, and 5.58 g/day among participants with depression. In NHANES 2021–2023, mean estimated prebiotic intake was 6.79 g/day overall, 6.90 g/day among participants without depression, and 5.78 g/day among participants with depression.

**Table 4.** Characteristics of restricted regression samples by depression status, NHANES 2015–2018 and 2021–2023 Table 4 long description.

	2015–2018			2021–2023		
Characteristic	Overall	Not depressed	Depressed	Overall	Not depressed	Depressed
Sample size, *n*	3,601	3,430	171	1,390	1,258	132
Age, years, mean (SE)	46.0 (0.6)	46.3 (0.6)	39.0 (1.7)	50.3 (0.9)	50.8 (0.9)	45.6 (1.6)
**Sex, *n* (%)**						
Male	1,843 (50.0%)	1,769 (50.3%)	74 (43.7%)	724 (55.8%)	662 (55.9%)	62 (54.8%)
Female	1,758 (50.0%)	1,661 (49.7%)	97 (56.3%)	666 (44.2%)	596 (44.1%)	70 (45.2%)
**Race/ethnicity, *n* (%)**						
Mexican American	492 (8.4%)	465 (8.4%)	27 (9.1%)	66 (5.6%)	59 (5.6%)	7 (5.4%)
Other Hispanic	375 (6.3%)	353 (6.2%)	22 (6.4%)	111 (7.8%)	95 (7.4%)	16 (11.8%)
Non-Hispanic White	1,247 (63.4%)	1,185 (63.4%)	62 (62.1%)	898 (63.2%)	821 (63.4%)	77 (61.1%)
Non-Hispanic Black	734 (9.9%)	697 (9.7%)	37 (14.8%)	121 (8.2%)	106 (7.8%)	15 (11.3%)
Non-Hispanic Asian	622 (8.9%)	608 (9.1%)	14 (4.7%)	104 (10.1%)	99 (10.8%)	5 (4.6%)
Other race/multiracial	131 (3.1%)	122 (3.1%)	9 (2.9%)	90 (5.1%)	78 (5.0%)	12 (5.7%)
**Education, *n* (%)**						
Did not graduate high school	629 (10.9%)	581 (10.6%)	48 (17.7%)	122 (6.2%)	102 (5.7%)	20 (10.6%)
High school/GED	871 (24.1%)	819 (23.8%)	52 (29.4%)	239 (22.0%)	210 (21.7%)	29 (25.3%)
Some college/AA	1,012 (28.5%)	965 (28.5%)	47 (28.5%)	340 (24.0%)	302 (23.5%)	38 (28.2%)
College graduate or above	1,089 (36.5%)	1,065 (37.1%)	24 (24.5%)	689 (47.8%)	644 (49.2%)	45 (35.9%)
Poverty ratio, mean (SE)	3.11 (0.08)	3.14 (0.07)	2.50 (0.25)	3.30 (0.13)	3.39 (0.12)	2.45 (0.26)
Total energy intake, kcal/day, mean (SE)	2,041 (24)	2,043 (26)	2,007 (95)	2,157 (42)	2,186 (41)	1,896 (97)
Estimated prebiotic intake, g/day, mean (SE)	6.56 (0.12)	6.61 (0.13)	5.58 (0.48)	6.79 (0.42)	6.90 (0.43)	5.78 (0.61)

Abbreviations: NHANES, National Health and Nutrition Examination Survey; GED, general educational development; AA, associate degree. Values are presented as weighted mean (SE) for continuous variables and unweighted n with weighted column percentage for categorical variables. Depression was defined as a Patient Health Questionnaire-9 score of 10 or greater. The restricted regression samples included adults aged 18 years or older with complete depression outcome and estimated prebiotic intake data, BMI 18-30 kg/m^2^, non-excessive alcohol intake, valid total energy intake, sleep adequacy data, employment status, and complete covariate data required for the corresponding period-specific regression model. For NHANES 2015–2018, participants reporting depression medication use were also excluded. Analyses accounted for the complex NHANES survey design, including dietary sample weights, strata, and primary sampling units. NHANES 2015–2018 and NHANES 2021–2023 were analysed as separate survey periods.

### Association between estimated prebiotic intake and depression

In the NHANES 2015–2018 restricted regression sample, higher estimated prebiotic intake was associated with lower odds of depression after multivariable adjustment (Table 5). The adjusted OR per 5 g/day higher estimated intake was 0.69 (95% CI: 0.49–0.98; *P* = 0.038) overall. In sex-stratified models, the association was significant among females (OR: 0.65; 95% CI: 0.45–0.94; *P* = 0.022), but not among males (OR: 0.69; 95% CI: 0.45–1.07; *P* = 0.098).

**Table 5.** Survey-weighted logistic regression analysis of estimated prebiotic intake and depression status in restricted regression samples, NHANES 2015–2018 and 2021–2023 Table 5 long description.

	2015–2018				2021–2023			
Model	*n*	Adjusted OR	95% CI	*P* value	*n*	Adjusted OR	95% CI	*P* value
Overall	3,601	0.69	0.49–0.98	0.038	1,390	1.03	0.74–1.43	0.867
Male	1,843	0.69	0.45–1.07	0.098	724	1.13	0.81–1.57	0.460
Female	1,758	0.65	0.45–0.94	0.022	666	0.93	0.53–1.60	0.767

Abbreviations: NHANES, National Health and Nutrition Examination Survey. ORs and 95% CIs were estimated using survey-weighted logistic regression and represent the odds of depression per 5 g/day higher estimated prebiotic intake. Overall models were adjusted for age, sex, race/ethnicity, education, poverty ratio, employment status, sleep adequacy, and total energy intake. Sex-stratified models were adjusted for age, race/ethnicity, education, poverty ratio, employment status, sleep adequacy, and total energy intake. Analyses accounted for the complex NHANES survey design, including dietary sample weights, strata, and primary sampling units.

In the NHANES 2021–2023 restricted regression sample, estimated prebiotic intake was not associated with depression after multivariable adjustment (Table 5). The adjusted OR per 5 g/day higher estimated intake was 1.03 (95% CI: 0.74–1.43; *P* = 0.867) overall, 1.13 among males (95% CI: 0.81–1.57; *P* = 0.460), and 0.93 among females (95% CI: 0.53–1.60; *P* = 0.767). Although mean estimated intake was descriptively lower among participants with depression in the restricted 2021–2023 sample, this difference did not translate into a statistically significant adjusted association. Sensitivity analyses were generally consistent with the primary period-specific findings. In NHANES 2015–2018, simplified, two-day-only, and energy-adjusted models supported an inverse association overall or among females. In NHANES 2021–2023, sensitivity analyses using simplified covariate adjustment, a two-day-only dietary recall sample, energy-adjusted prebiotic intake, and exclusion of participants reporting any prescription medication use produced no statistically significant associations (Supplementary Table 6).

## Discussion

This repeated cross-sectional study analysed NHANES 2015–2018 and NHANES 2021–2023 as independent survey periods to estimate intake of ISAPP-recognised prebiotics and examine associations with depression among US adults. Mean estimated prebiotic intake was broadly similar across periods, although it was modestly lower in NHANES 2021–2023 than in NHANES 2015–2018. In NHANES 2015–2018, mean estimated prebiotic intake was 6.44 g/day in the descriptive analytic sample, and higher estimated intake was associated with lower odds of depression overall and among females, but not among males, after adjustment for demographic, socio-economic, sleep, employment, and dietary covariates. In NHANES 2021–2023, mean estimated prebiotic intake was 6.09 g/day, and estimated prebiotic intake was not associated with depression after multivariable adjustment. These period-specific findings indicate that the inverse association observed in 2015–2018 was not replicated in the later NHANES period, despite broadly similar mean estimated intake. These results should therefore be interpreted cautiously and viewed as hypothesis-generating rather than as evidence of a consistent protective association.

The food-source and FNDDS assignment analyses showed that ISAPP-recognised prebiotics are present across multiple food categories in the US diet. Some foods with high estimated prebiotic concentrations, such as dandelion greens and Jerusalem artichokes, are not commonly consumed in the US diet. More widely consumed foods, including garlic, leeks, onions, grains, and legumes, may contribute more meaningfully to habitual prebiotic intake, particularly when consumed regularly or as ingredients in mixed dishes. This supports the interpretation that achieving prebiotic intake within the estimated effective range is more likely to depend on overall dietary patterns and repeated inclusion of multiple prebiotic-containing foods than on any single high-prebiotic food source. The FNDDS assignment process also highlights the importance of accounting for mixed dishes, because prebiotic-containing ingredients may contribute to intake even when the reported food is a composite item.

Subgroup differences in estimated prebiotic intake should be interpreted cautiously. Detailed subgroup estimates are provided in Supplementary Table 5. In general, absolute estimated intake may vary across demographic and socio-economic groups partly because of differences in total intake volume, food choices, and dietary patterns. Energy-adjusted estimates help account for total energy intake but do not eliminate potential confounding by broader dietary quality, food access, or structural dietary context. These findings are broadly consistent with prior literature showing that fibre intake and dietary quality vary by income, race/ethnicity, age, food source, and food environment.^(44–55)^ However, these contextual factors were not directly tested in the present analysis, and subgroup differences in estimated prebiotic intake should not be interpreted as evidence that demographic or socio-economic characteristics causally modify the relationship between prebiotic intake and depression.

Depression prevalence and restricted-sample findings should also be interpreted in context. The prevalence of depression in the NHANES 2015–2018 descriptive analytic sample and the higher prevalence among females were broadly consistent with prior US estimates and epidemiological literature.^(2,56–58)^ The lower weighted prevalence observed in the restricted regression samples likely reflects the analytic restrictions applied to those samples, including exclusions based on BMI, alcohol intake, missing covariates, and, in 2015–2018, reported depression medication use. Sample-comparison analyses showed that restricted regression samples differed from excluded participants on several characteristics. Therefore, results from the restricted samples should be interpreted as estimates for more selective analytic subgroups rather than as fully generalisable estimates for all US adults.

Cross-period differences in estimated prebiotic intake and model results should be interpreted within the repeated cross-sectional design. NHANES 2015–2018 and NHANES August 2021–August 2023 consist of independent population samples, so differences between periods do not represent longitudinal change within the same participants. The modestly lower mean estimated intake in 2021–2023 may reflect differences in sample composition, reported food choices, total energy intake, FNDDS food-code structure, dietary recall procedures, post-pandemic food access or dietary behaviours, or random sampling variation. NHANES 2021–2023 followed the suspension of NHANES field operations during the COVID-19 pandemic and used updated survey procedures and dietary data collection methods.^(20–25)^ For these reasons, the two periods are best interpreted as separate nationally representative snapshots rather than as a continuous trend from 2015 through 2023. Analysing both periods allowed evaluation of whether the inverse association between estimated prebiotic intake and depression observed in 2015–2018 was also evident in a more recent NHANES sample.

The absence of a statistically significant association in NHANES 2021–2023 should be considered when interpreting the overall findings. The point estimate for the overall 2021–2023 model was close to the null, and the sex-stratified models and sensitivity analyses also did not show statistically significant associations. Thus, the later period does not provide confirmatory evidence for the inverse association observed in 2015–2018. The lack of replication may reflect period-specific variability, residual confounding in either period, differences in restricted sample composition, dietary and mental health disruptions following the COVID-19 pandemic, exposure measurement error, or chance. The smaller 2021–2023 restricted regression sample may also have reduced precision, but the near-null overall point estimate suggests that imprecision alone does not fully explain the result. Accordingly, the 2015–2018 finding should not be interpreted as a consistent population-level association or as evidence that higher prebiotic intake reduces depression risk.

The inverse association observed in 2015–2018 remains biologically plausible, but the lack of a corresponding association in 2021–2023 emphasises that the evidence remains uncertain. Major depressive disorder has been associated with disturbances in the gut microbiota.^(4)^ Through effects on gut microbial metabolism, prebiotics may plausibly influence depressive symptoms through pathways involving short-chain fatty acid production, immune-inflammatory signalling, gut barrier function, and neuroendocrine regulation.^(10)^ However, human studies of prebiotics, probiotics, synbiotics, fibre, and depression have produced mixed findings.^(11–15)^ The present repeated cross-sectional results are consistent with that broader uncertainty and should be viewed as hypothesis-generating rather than confirmatory.

The sex-stratified results also require cautious interpretation. In NHANES 2015–2018, the inverse association was statistically significant among females but not males. In NHANES 2021–2023, neither the male nor female sex-stratified model showed a statistically significant association. The female point estimate in 2021–2023 was close to the null, and the male point estimate was not inverse. These findings do not establish that the association differs by sex. The observed pattern may reflect differences in dietary intake, total energy intake, depression prevalence, sample size, residual confounding, or chance. Future studies with larger samples and formal interaction testing are needed before drawing conclusions about sex-specific associations between prebiotic intake and depression.

## Strengths and limitations

This study has several strengths. It used nationally representative NHANES data, accounted for the complex survey design, and estimated prebiotic intake using a food-code-level approach focused on ISAPP-recognised prebiotic classes. The analysis incorporated FNDDS food-code and ingredient-level information to estimate prebiotic intake from both individual foods and mixed dishes. Food-code assignments for NHANES 2021–2023 used the corresponding FNDDS files, while values for foods represented across periods were harmonised to improve consistency. The study also included sample-comparison analyses and sensitivity analyses to evaluate the robustness and generalizability of the findings. The repeated cross-sectional design allowed estimated prebiotic intake and its association with depression to be evaluated in both a pre-pandemic survey period and a more recent NHANES period, while preserving period-specific interpretation.

Several limitations should be considered. The repeated cross-sectional design, with independent samples in each survey period, prevents determination of temporality or causality, and reverse causality remains possible because depressive symptoms may influence appetite, dietary behaviours, energy intake, and consumption of prebiotic-containing foods.^(59)^ Depression was also defined using the PHQ-9 rather than clinical diagnosis. Although the PHQ-9 is a validated screening instrument,^(35)^ it does not capture all aspects of depressive disorder, treatment history, chronicity, or symptom context. In addition, depressive symptoms may fluctuate over time, whereas dietary intake was measured over one or two recall days. Residual confounding is also possible. Although models adjusted for demographic, socio-economic, sleep, employment, and dietary factors, unmeasured or imperfectly measured factors may have influenced the results. Sleep was represented using self-reported sleep duration or adequacy rather than a validated measure of sleep quality, insomnia symptoms, sleep disturbance, or circadian disruption.^(37,38)^ Food insecurity, stress, chronic disease burden, supplement use, gastrointestinal conditions, and overall dietary pattern were also not fully captured.

Dietary exposure measurement is another important limitation. Prebiotic intake was estimated using 24-hour dietary recalls, which are subject to recall error, day-to-day variation, and misreporting, and one or two recalls may not fully capture usual intake of prebiotic-containing foods. Prebiotic composition was estimated from published values and matched to FNDDS foods using assumptions because source-food data were incomplete for many items. Values may vary by cultivar, growing conditions, processing, cooking, and formulation. For mixed dishes, estimates were based on FNDDS standard recipe ingredients rather than the exact recipes consumed by participants. These sources of error may have led to exposure misclassification.

Finally, the restricted regression samples may not represent all US adults. These samples excluded participants with BMI outside 18–30 kg/m^2^, excessive alcohol intake, and missing covariates; the 2015–2018 restricted sample also excluded participants reporting depression medication use. Sample-comparison analyses showed that included and excluded participants differed on several characteristics. In NHANES 2021–2023, depression medication use could not be identified comparably, and the sensitivity analysis excluding any prescription medication use was conservative but not equivalent. The 2015–2018 and 2021–2023 analyses were also not pooled and should not be interpreted as a continuous 2015–2023 trend or as repeated measures of the same individuals. The 2021–2023 cycle occurred after major disruptions related to the COVID-19 pandemic and differed from earlier cycles in survey context and procedures.^(20–25)^ Therefore, differences across periods cannot be attributed to a single cause.

### Contributions and clinical relevance

This study adds to the literature by providing period-specific population-level estimates of ISAPP-recognised prebiotic intake among US adults using repeated cross-sectional data from NHANES 2015–2018 and NHANES 2021–2023, and by integrating published prebiotic composition data with FNDDS food-code and ingredient-level data. To our knowledge, this is among the first nationally representative analyses to estimate intake of ISAPP-recognised prebiotics while also examining their association with depression status across two NHANES survey periods. The findings from NHANES 2015–2018 suggested an inverse association between estimated prebiotic intake and depression in the restricted regression sample; however, this association was not evident in NHANES 2021–2023. These observational findings do not support treatment recommendations. However, they suggest that habitual intake of prebiotic-containing foods may be relevant to broader dietary assessment and warrants further study in prospective and interventional research.

## Conclusion

In these repeated cross-sectional analyses of US adults, higher estimated intake of ISAPP-recognised prebiotics was associated with lower odds of depression in the NHANES 2015–2018 restricted regression sample, with a significant association also observed among females. However, this inverse association was not replicated in the NHANES 2021–2023 analysis. These findings should be interpreted cautiously given the observational design, independent survey-period samples, restricted regression samples, potential reverse causality, and measurement error in estimated prebiotic intake. Prospective studies and intervention trials are needed to clarify whether higher habitual prebiotic intake is consistently associated with lower depressive symptoms and to better define its potential relevance to mental health.

## Supporting information

10.1017/jns.2026.10136.sm001 Boyd and Gieng supplementary material 1 Boyd and Gieng supplementary material

10.1017/jns.2026.10136.sm002 Boyd and Gieng supplementary material 2 Boyd and Gieng supplementary material

10.1017/jns.2026.10136.sm003 Boyd and Gieng supplementary material 3 Boyd and Gieng supplementary material

10.1017/jns.2026.10136.sm004 Boyd and Gieng supplementary material 4 Boyd and Gieng supplementary material

10.1017/jns.2026.10136.sm005 Boyd and Gieng supplementary material 5 Boyd and Gieng supplementary material

10.1017/jns.2026.10136.sm006 Boyd and Gieng supplementary material 6 Boyd and Gieng supplementary material

## Data Availability

The data underlying this article are publicly available from the National Health and Nutrition Examination Survey (NHANES) at https://www.cdc.gov/nchs/nhanes/.

## Supplementary material

The supplementary material for this article can be found at https://doi.org/10.1017/jns.2026.10136.

## Figure 1. Long description

A flow diagram of participant selection for repeated cross-sectional analyses. Panel A: NHANES 2015-2018 analytic samples. The diagram starts with a merged sample of 19,225 participants. 11,817 participants are excluded from descriptive analyses due to ineligibility, missing or invalid data, and other criteria. 7,408 participants are included in descriptive analyses. 3,807 participants are excluded from restricted regression analyses due to BMI outside 18-30 kg/m^2, excessive alcohol intake, depression medication use, and missing required model covariate data. 3,601 participants are included in restricted regression analyses. Panel B: NHANES August 2021-August 2023 analytic samples. The diagram starts with a demographic file of 11,933 participants. 8,124 participants are excluded from descriptive analyses due to ineligibility, missing or invalid data, and other criteria. 3,809 participants are included in descriptive analyses. 2,419 participants are excluded from restricted regression analyses due to BMI outside 18-30 kg/m^2, excessive alcohol intake, and missing required model covariate data. 1,390 participants are included in restricted regression analyses.

Navigate back to Figure 1..

## Table 1. Long description

A table comparing demographic characteristics of US adults in two different time periods, 2015-2018 and 2021-2023. The table has 12 rows and 7 columns. The columns are labeled as follows: Characteristic, 2015-2018 value, SE, n, 2021-2023 value, SE, n. The rows are labeled as follows: Age, Sex (Male, Female), Depression status (Not depressed, Depressed), Poverty ratio, Race/ethnicity (Mexican American, Other Hispanic, Non-Hispanic White, Non-Hispanic Black, Non-Hispanic Asian, Other race/multiracial), Education (Did not graduate high school, High school/GED, Some college/AA, College graduate or above), BMI, BMI category (Underweight, Normal, Overweight, Obese). Row 1: Age, years, 47.2, 0.5, 7,408, 46.6, 0.6, 3,809. Row 2: Sex, Male, 48.7,, 3,558, 49.1,, 1,681. Row 3: Sex, Female, 51.3,, 3,850, 50.9,, 2,128. Row 4: Depression status, Not depressed, 91.7,, 6,777, 88.6,, 3,361. Row 5: Depression status, Depressed, 8.3,, 631, 11.4,, 448. Row 6: Poverty ratio, 3.05, 0.06, 7,408, 3.16, 0.11, 3,809. Row 7: Race/ethnicity, Mexican American, 8.8,, 1,093, 6.7,, 225. Row 8: Race/ethnicity, Other Hispanic, 5.9,, 770, 9.1,, 346. Row 9: Race/ethnicity, Non-Hispanic White, 65.2,, 2,783, 63.3,, 2,446. Row 10: Race/ethnicity, Non-Hispanic Black, 10.4,, 1,636, 10.3,, 398. Row 11: Race/ethnicity, Non-Hispanic Asian, 5.4,, 778, 5.5,, 163. Row 12: Race/ethnicity, Other race/multiracial, 4.3,, 348, 5.1,, 231. Row 13: Education, Did not graduate high school, 10.9,, 1,347, 7.5,, 348. Row 14: Education, High school/GED, 25.2,, 1,818, 23.2,, 731. Row 15: Education, Some college/AA, 32.2,, 2,378, 30.8,, 1,181. Row 16: Education, College graduate or above, 31.8,, 1,865, 38.5,, 1,549. Row 17: BMI, kg per square meter, 29.6, 0.2, 7,408, 29.8, 0.3, 3,809. Row 18: BMI category, Underweight, 1.4,, 106, 1.1,, 45. Row 19: BMI category, Normal, 25.8,, 1,865, 24.6,, 942. Row 20: BMI category, Overweight, 30.8,, 2,284, 33.4,, 1,238. Row 21: BMI category, Obese, 42.1,, 3,153, 40.9,, 1,584.

Navigate back to Table 1..

## Table 2. Long description

A table comparing the total prebiotic content in various foods and the food portion containing 5 grams of prebiotics. The table has 11 rows and 3 columns. The columns are labeled Food, Total prebiotic in food, mg/g food, and Food portion containing 5 g prebiotics, oz food. The row labels are Dandelion greens, Jerusalem artichoke, Garlic, Leek, Onions, Onion rings, Peas and onions, Creamed onions, Cowpeas, and Asparagus. The values in the table are as follows: Row 1: Dandelion greens, 155.5-243.0, 0.7-1.1. Row 2: Jerusalem artichoke, 209.7, 0.8. Row 3: Garlic, 191.5-192.6, 0.9. Row 4: Leek, 123.0-127.7, 1.4. Row 5: Onions, 78.9-106.1, 1.7-2.2. Row 6: Onion rings, 57.8-58.1, 3.0-3.1. Row 7: Peas and onions, 54.5-55.9, 3.2. Row 8: Creamed onions, 50.8, 3.5. Row 9: Cowpeas, 50.2-50.4, 3.5. Row 10: Asparagus, 50.0, 3.5.

Navigate back to Table 2..

## Table 3. Long description

A table comparing prebiotic intake among US adults across two survey periods. The table has two rows and six columns. The columns are labeled Survey Period, n, g/day, SE, 95% CI, g/1000 kcal/day, SE, and 95% CI. The row labels are 2015-2018 and 2021-2023. Row 1: 2015-2018, 7408, 6.44, 0.08, 6.28-6.60, 3.14, 0.03, 3.07-3.20. Row 2: 2021-2023, 3809, 6.09, 0.17, 5.71-6.46, 2.93, 0.04, 2.85-3.02.

Navigate back to Table 3..

## Table 4. Long description

The table compares characteristics of restricted regression samples by depression status in NHANES 2015-2018 and 2021-2023. It has 12 rows and 6 columns. The columns are labeled 2015-2018 Overall, 2015-2018 Not depressed, 2015-2018 Depressed, 2021-2023 Overall, 2021-2023 Not depressed, and 2021-2023 Depressed. The rows are labeled Sample size, n; Age, years, mean (SE); Sex, n (%); Male; Female; Race/ethnicity, n (%); Mexican American; Other Hispanic; Non-Hispanic White; Non-Hispanic Black; Non-Hispanic Asian; Other race/multiracial; Education, n (%); Did not graduate high school; High school/GED; Some college/AA; College graduate or above; Poverty ratio, mean (SE); Total energy intake, kcal/day, mean (SE); Estimated prebiotic intake, g/day, mean (SE). Row 1: Sample size, n: 3601, 3430, 171, 1390, 1258, 132. Row 2: Age, years, mean (SE): 46.0 (0.6), 46.3 (0.6), 39.0 (1.7), 50.3 (0.9), 50.8 (0.9), 45.6 (1.6). Row 3: Sex, n (%): Male: 1843 (50.0%), 1769 (50.3%), 74 (43.7%), 724 (55.8%), 662 (55.9%), 62 (54.8%). Female: 1758 (50.0%), 1661 (49.7%), 97 (56.3%), 666 (44.2%), 596 (44.1%), 70 (45.2%). Row 4: Race/ethnicity, n (%): Mexican American: 492 (8.4%), 465 (8.4%), 27 (9.1%), 66 (5.6%), 59 (5.6%), 7 (5.4%). Other Hispanic: 375 (6.3%), 353 (6.2%), 22 (6.4%), 111 (7.8%), 95 (7.4%), 16 (11.8%). Non-Hispanic White: 1247 (63.4%), 1185 (63.4%), 62 (62.1%), 898 (63.2%), 821 (63.4%), 77 (61.1%). Non-Hispanic Black: 734 (9.9%), 697 (9.7%), 37 (14.8%), 121 (8.2%), 106 (7.8%), 15 (11.3%). Non-Hispanic Asian: 622 (8.9%), 608 (9.1%), 14 (4.7%), 104 (10.1%), 99 (10.8%), 5 (4.6%). Other race/multiracial: 131 (3.1%), 122 (3.1%), 9 (2.9%), 90 (5.1%), 78 (5.0%), 12 (5.7%). Row 5: Education, n (%): Did not graduate high school: 629 (10.9%), 581 (10.6%), 48 (17.7%), 122 (6.2%), 102 (5.7%), 20 (10.6%). High school/GED: 871 (24.1%), 819 (23.8%), 52 (29.4%), 239 (22.0%), 210 (21.7%), 29 (25.3%). Some college/AA: 1012 (28.5%), 965 (28.5%), 47 (28.5%), 340 (24.0%), 302 (23.5%), 38 (28.2%). College graduate or above: 1089 (36.5%), 1065 (37.1%), 24 (24.5%), 689 (47.8%), 644 (49.2%), 45 (35.9%). Row 6: Poverty ratio, mean (SE): 3.11 (0.08), 3.14 (0.07), 2.50 (0.25), 3.30 (0.13), 3.39 (0.12), 2.45 (0.26). Row 7: Total energy intake, kcal/day, mean (SE): 2041 (24), 2043 (26), 2007 (95), 2157 (42), 2186 (41), 1896 (97). Row 8: Estimated prebiotic intake, g/day, mean (SE): 6.56 (0.12), 6.61 (0.13), 5.58 (0.48), 6.79 (0.42), 6.90 (0.43), 5.78 (0.61).

Navigate back to Table 4..

## Table 5. Long description

A table comparing prebiotic intake and depression status across different years and demographics. The table has two main sections: 2015-2018 and 2021-2023. Each section includes columns for Model, n, Adjusted OR, 95% CI, and P value. The rows are categorized into Overall, Male, and Female. Row 1: Overall, 3601, Adjusted OR 0.69, 95% CI 0.49-0.98, P value 0.038. Row 2: Male, 1843, Adjusted OR 0.69, 95% CI 0.45-1.07, P value 0.098. Row 3: Female, 1758, Adjusted OR 0.65, 95% CI 0.45-0.94, P value 0.022. Row 4: Overall, 1390, Adjusted OR 1.03, 95% CI 0.74-1.43, P value 0.867. Row 5: Male, 724, Adjusted OR 1.13, 95% CI 0.81-1.57, P value 0.460. Row 6: Female, 666, Adjusted OR 0.93, 95% CI 0.53-1.60, P value 0.767.

Navigate back to Table 5..

## References

[1] Weinberger AH , Gbedemah M , Martinez AM , Nash D , Galea S , Goodwin RD. Trends in depression prevalence in the USA from 2005 to 2015: widening disparities in vulnerable groups. Psychol Med. 2018;48:1308–1315. 29021005 10.1017/S0033291717002781

[2] Brody DJ , Pratt LA , Hughes JP. Prevalence of depression among adults aged 20 and over: United States, 2013–2016. NCHS Data Brief no. 303. National Center for Health Statistics; 2018. 29638213

[3] Organisation for Economic Co-operation and Development. How is depression related to education?; 2018. Accessed June 2026. https://www.oecd-ilibrary.org/education/how-is-depression-related-to-education_782fc82d-en.

[4] Nikolova VL , Smith MRB , Hall LJ , et al. Perturbations in gut microbiota composition in psychiatric disorders: a review and meta-analysis. JAMA Psychiatry. 2021;78:1343–1354. 34524405 10.1001/jamapsychiatry.2021.2573 PMC8444066

[5] Gibson GR , Hutkins R , Sanders ME , et al. Expert consensus document: The International Scientific Association for Probiotics and Prebiotics (ISAPP) consensus statement on the definition and scope of prebiotics. Nat Rev Gastroenterol Hepatol. 2017;14:491–502. 28611480 10.1038/nrgastro.2017.75

[6] Hutkins R , Walter J , Gibson GR , et al. Classifying compounds as prebiotics: scientific perspectives and recommendations. Nat Rev Gastroenterol Hepatol. 2025;22:54–70. 39358591 10.1038/s41575-024-00981-6

[7] Fiori F , Concina F , Turati F , et al. Quantification of naturally occurring prebiotic fiber in Italian foods. J Food Compost Anal. 2022;112:104678.

[8] Jovanovic-Malinovska R , Kuzmanova S , Winkelhausen E. Oligosaccharide profile in fruits and vegetables as sources of prebiotics and functional foods. Int J Food Prop. 2014;17:949–965.

[9] Reid G , Sanders ME , Gaskins HR , et al. New scientific paradigms for probiotics and prebiotics. J Clin Gastroenterol. 2003;37:105–118. 12869879 10.1097/00004836-200308000-00004

[10] Yang Y , Zhou B , Zhang S , et al. Prebiotics for depression: how does the gut microbiota play a role? Front Nutr. 2023;10:1206468. 37485386 10.3389/fnut.2023.1206468 PMC10358272

[11] Jackson PP , Wijeyesekera A , Williams CM , et al. Inulin-type fructans and 2’-fucosyllactose alter both microbial composition and appear to alleviate stress-induced mood state in a working population compared to placebo (maltodextrin): the EFFICAD trial, a randomized, controlled trial. Am J Clin Nutr. 2023;118:938–955. 37657523 10.1016/j.ajcnut.2023.08.016 PMC10636234

[12] Freijy TM , Cribb L , Oliver G , et al. Effects of a high-prebiotic diet versus probiotic supplements versus synbiotics on adult mental health: the “Gut Feelings” randomised controlled trial. Front Neurosci. 2022;16:1097278. 36815026 10.3389/fnins.2022.1097278 PMC9940791

[13] Liu RT , Walsh RFL , Sheehan AE. Prebiotics and probiotics for depression and anxiety: a systematic review and meta-analysis of controlled clinical trials. Neurosci Biobehav Rev. 2019;102:13–23. 31004628 10.1016/j.neubiorev.2019.03.023 PMC6584030

[14] Alli SR , Gorbovskaya I , Liu JCW , et al. The gut microbiome in depression and potential benefit of prebiotics, probiotics and synbiotics: a systematic review of clinical trials and observational studies. Int J Mol Sci. 2022;23:4494. 35562885 10.3390/ijms23094494 PMC9101152

[15] Aslam H , Lotfaliany M , So D , et al. Fiber intake and fiber intervention in depression and anxiety: a systematic review and meta-analysis of observational studies and randomized controlled trials. Nutr Rev. 2024;82:1678–1695. 38007616 10.1093/nutrit/nuad143 PMC11551482

[16] Moshfegh AJ , Friday JE , Goldman JP , et al. Presence of inulin and oligofructose in the diets of Americans. J Nutr. 1999;129:1407S–1411S. 10395608 10.1093/jn/129.7.1407S

[17] Miketinas DC , Shankar K , Maiya M , et al. Usual dietary intake of resistant starch in US adults from NHANES 2015-2016. J Nutr. 2020;150:2738–2747. 32840627 10.1093/jn/nxaa232

[18] Centers for Disease Control and Prevention, National Center for Health Statistics. NHANES 2015-2016 procedure manuals; 2016. Accessed June 2026. https://wwwn.cdc.gov/nchs/nhanes/continuousnhanes/manuals.aspx?BeginYear=2015.

[19] Centers for Disease Control and Prevention, National Center for Health Statistics. NHANES 2017-2018 procedure manuals. 2018. Accessed June 2026. https://wwwn.cdc.gov/nchs/nhanes/continuousnhanes/manuals.aspx?BeginYear=2017.

[20] Centers for Disease Control and Prevention, National Center for Health Statistics. NHANES August 2021-August 2023 questionnaires, datasets, and related documentation. 2024. Accessed June 2026. https://wwwn.cdc.gov/nchs/nhanes/continuousnhanes/default.aspx?Cycle=2021-2023.

[21] Centers for Disease Control and Prevention, National Center for Health Statistics. NHANES August 2021-August 2023 procedure manuals. 2021. Accessed June 2026. https://wwwn.cdc.gov/nchs/nhanes/continuousnhanes/manuals.aspx?Cycle=2021-2023.

[22] Centers for Disease Control and Prevention, National Center for Health Statistics. August 2021–August 2023 dietary data: continuous NHANES. 2024. Accessed June 2026. https://wwwn.cdc.gov/nchs/nhanes/search/datapage.aspx?Component=Dietary&Cycle=2021-2023.

[23] Centers for Disease Control and Prevention, National Center for Health Statistics. NHANES survey methods and analytic guidelines; n.d. Accessed June 2026. https://wwwn.cdc.gov/nchs/nhanes/analyticguidelines.aspx.

[24] U.S. Department of Agriculture, Agricultural Research Service, Food Surveys Research Group. Food and Nutrient Database for Dietary Studies (FNDDS); 2024. Accessed June 2026. https://www.ars.usda.gov/northeast-area/beltsville-md-bhnrc/beltsville-human-nutrition-research-center/food-surveys-research-group/docs/fndds/.

[25] U.S. Department of Agriculture, Agricultural Research Service, Food Surveys Research Group. What We Eat in America, NHANES August 2021-August 2023; 2026. Accessed June 2026. https://www.ars.usda.gov/northeast-area/beltsville-md-bhnrc/beltsville-human-nutrition-research-center/food-surveys-research-group/docs/wweianhanes-2021-2023/.

[26] Campbell JM , Bauer LL , Fahey GC , et al. Selected fructooligosaccharide (1-kestose, nystose, and 1F-beta-fructofuranosylnystose) composition of foods and feeds. J Agric Food Chem. 1997;45:3076–3082.

[27] Biesiekierski JR , Rosella O , Rose R , et al. Quantification of fructans, galacto-oligosaccharides and other short-chain carbohydrates in processed grains and cereals. J Hum Nutr Diet. 2011;24:154–176. 21332832 10.1111/j.1365-277X.2010.01139.x

[28] Dwivedi S , Sahrawat K , Puppala N , et al. Plant prebiotics and human health: biotechnology to breed prebiotic-rich nutritious food crops. Electron J Biotechnol. 2014;17:238–245.

[29] Mudannayake DC , Wimalasiri KMS , Silva KFST , et al. Selected Sri Lankan food plants and other herbs as potential sources of inulin-type fructans. J Natl Sci Found Sri Lanka. 2015;43:35–43.

[30] Muir JG , Rose R , Rosella O , et al. Measurement of short-chain carbohydrates in common Australian vegetables and fruits by high-performance liquid chromatography (HPLC). J Agric Food Chem. 2009;57:554–565. 19123815 10.1021/jf802700e

[31] Onyenekwe PC , Njoku GC , Ameh DA. Effect of cowpea (Vigna unguiculata) processing methods on flatus causing oligosaccharides. Nutr Res. 2000; 20:349–358.

[32] Sangeetha PT , Ramesh MN , Prapulla SG. Recent trends in the microbial production, analysis and application of fructooligosaccharides. Trends Food Sci Technol. 2005; 16:442–457.

[33] Słupski J , Korus A. Changes in the content of low molecular weight carbohydrates in frozen immature bean seeds depending on type and method of processing prior to freezing. Int J Refrig. 2014; 43:187–193.

[34] García-Domingo C , Rupérez P , Saura-Calixto F. Indigestible fraction and starch availability in peas measured in vitro. Z Lebensm Unters Forsch A. 1997; 205:43–47.

[35] Martin A , Rief W , Klaiberg A , et al. Validity of the Brief Patient Health Questionnaire Mood Scale (PHQ-9) in the general population. Gen Hosp Psychiatry. 2006; 28:71–77. 16377369 10.1016/j.genhosppsych.2005.07.003

[36] U.S. Department of Agriculture and U.S. Department of Health and Human Services. Dietary Guidelines for Americans, 2020-2025, 9th ed.; 2020. Accessed June 2026. https://www.dietaryguidelines.gov/sites/default/files/2020-12/Dietary_Guidelines_for_Americans_2020-2025.pdf.

[37] Zhai L , Zhang H , Zhang D. Sleep duration and depression among adults: a meta-analysis of prospective studies. Depress Anxiety. 2015; 32:664–670. 26047492 10.1002/da.22386

[38] Ikonte CJ , Mun JG , Reider CA , et al. Micronutrient inadequacy in short sleep: analysis of the NHANES 2005-2016. Nutrients. 2019; 11:2335. 31581561 10.3390/nu11102335 PMC6835726

[39] Hergenrather KC , Zeglin RJ , McGuire-Kuletz M , et al. Employment as a social determinant of health: a review of longitudinal studies exploring the relationship between employment status and mental health. Rehabil Res Policy Educ. 2015; 29:261–290.

[40] Zhai L , Wang J , Liu Y , et al. Involuntary retirement and depression among adults: a systematic review and meta-analysis of longitudinal studies. Front Psychiatry. 2022; 13:747334. 35185644 10.3389/fpsyt.2022.747334 PMC8854640

[41] Willett W. Implications of total energy intake for epidemiologic analyses. In Nutritional Epidemiology. Oxford University Press, 3rd; 2012: 260–286.

[42] Willett WC , Howe GR , Kushi LH. Adjustment for total energy intake in epidemiologic studies. Am J Clin Nutr. 1997; 65:1220S–1228S. 9094926 10.1093/ajcn/65.4.1220S

[43] Centers for Disease Control and Prevention, National Center for Health Statistics. NHANES tutorials: Weighting module; n.d. Accessed June 2026. https://wwwn.cdc.gov/nchs/nhanes/tutorials/weighting.aspx.

[44] Quagliani D , Felt-Gunderson P. Closing America’s fiber intake gap: communication strategies from a food and fiber summit. Am J Lifestyle Med. 2017; 11:80–85. 30202317 10.1177/1559827615588079 PMC6124841

[45] Saksena MJ , Okrent AM , Hamrick KS , editors. America’s eating habits: food away from home. Economic Information Bulletin no. 196. Washington, DC: U.S. Department of Agriculture, Economic Research Service. 2018. Accessed June 2026. https://www.ers.usda.gov/publications/pub-details?pubid=90227.

[46] Clegg ME , Williams EA. Optimizing nutrition in older people. Maturitas. 2018; 112:34–38. 29704915 10.1016/j.maturitas.2018.04.001

[47] Giezenaar C , Chapman I , Luscombe-Marsh N , et al. Ageing is associated with decreases in appetite and energy intake: a meta-analysis in healthy adults. Nutrients. 2016; 8:28. 26751475 10.3390/nu8010028 PMC4728642

[48] Lin BH , Guthrie J , Smith T. Dietary quality by food source and demographics in the United States, 1977-2018. Economic Information Bulletin no. 249. Washington, DC: U.S. Department of Agriculture, Economic Research Service. 2023. Accessed June 2026. https://www.ers.usda.gov/publications/pub-details?pubid=105955.

[49] Storey M , Anderson P. Income and race/ethnicity influence dietary fiber intake and vegetable consumption. Nutr Res. 2014; 34:844–850. 25262170 10.1016/j.nutres.2014.08.016

[50] Valerino-Perea S , Lara-Castor L , Armstrong MEG , et al. Definition of the traditional Mexican diet and its role in health: a systematic review. Nutrients. 2019; 11:2803. 31744179 10.3390/nu11112803 PMC6893605

[51] Testa A , Jackson DB. Food insecurity, food deserts, and waist-to-height ratio: variation by sex and race/ethnicity. J Community Health. 2019; 44:444–451. 30560310 10.1007/s10900-018-00601-w

[52] Cooksey Stowers K , Jiang Q , Atoloye A , et al. Racial differences in perceived food swamp and food desert exposure and disparities in self-reported dietary habits. Int J Environ Res Public Health. 2020; 17:7143. 33003573 10.3390/ijerph17197143 PMC7579470

[53] Chai W , Fan JX , Wen M. Association of individual and neighborhood factors with home food availability: evidence from the National Health and Nutrition Examination Survey. J Acad Nutr Diet. 2018; 118:815–823. 29396154 10.1016/j.jand.2017.11.009 PMC5924612

[54] Lucan SC , Maroko AR , Patel AN , et al. Healthful and less-healthful foods and drinks from storefront and non-storefront businesses: implications for “food deserts”, “food swamps” and food-source disparities. Public Health Nutr. 2020; 23:1428–1439. 32223780 10.1017/S1368980019004427 PMC7196006

[55] McAnulty JT , Akabas SR , Thuppal SV , et al. Fiber intake varies by poverty-income ratio and race/ethnicity in US adults. Nutr Today. 2017; 52:73–79.

[56] Jung SJ , Woo HT , Cho S , et al. Association between body size, weight change and depression: systematic review and meta-analysis. Br J Psychiatry. 2017; 211:14–21. 28428339 10.1192/bjp.bp.116.186726

[57] Lee B , Wang Y , Carlson SA , et al. National, state-level, and county-level prevalence estimates of adults aged 18 years and older self-reporting a lifetime diagnosis of depression: United States, 2020. MMWR Morb Mortal Wkly Rep. 2023; 72:644–650. 10.15585/mmwr.mm7224a1. 37318995 PMC10328468

[58] Musliner KL , Munk-Olsen T , Eaton WW , et al. Heterogeneity in long-term trajectories of depressive symptoms: patterns, predictors and outcomes. J Affect Disord. 2016; 192:199–211. 26745437 10.1016/j.jad.2015.12.030 PMC4761648

[59] Molero P , De Lorenzi F , Gędek A , et al. Diet quality and depression risk: a systematic review and meta-analysis of prospective studies. J Affect Disord. 2025; 382:154–166. 40158860 10.1016/j.jad.2025.03.162

