# Supplementary material for: Estimated prebiotic intake and depression in US adults: repeated cross-sectional analyses of NHANES 2015–2018 and 2021–2023
Source: J Nutr Sci. 2026 Jul 31;15:e70. doi: 10.1017/jns.2026.10136 (PMC13439025; doi:10.1017/jns.2026.10136)
Supplement: Supplementary file 1 [file S2048679026101360sup001.docx]

**Supplementary Table 1**
**Estimated prebiotic content of source foods used to assign ISAPP-recognized prebiotic values**

| **Food item group** | **Food item** | **Prebiotic types present** | **Individual prebiotic values, g/100 g food** | **Total prebiotic, g/100 g food** | **Total prebiotic, mg/g food** | **Reference ID(s)** |
| --- | --- | --- | --- | --- | --- | --- |
| Fruit | Banana, dried | Inulin, oligofructose | Inulin: 0.9–2.0  Inulin midpoint: 1.45  Oligofructose: 0.9–2.0  Oligofructose midpoint: 1.45 | 2.9 | 29 | 6 |
| Fruit | Banana, raw | Inulin, oligofructose | Inulin: 0.3–0.7  Inulin midpoint: 0.50  Oligofructose: 0.3–0.7  Oligofructose midpoint: 0.50 | 1 | 10 | 6 |
| Fruit | Blueberry | FOS | FOS: 0.50 | 0.5 | 5 | 5 |
| Fruit | Pear | FOS | FOS: 0.56 | 0.56 | 5.6 | 5 |
| Fruit | Raspberry | FOS | FOS: 0.51 | 0.51 | 5.1 | 5 |
| Fruit | Watermelon | FOS | FOS: 0.81 | 0.81 | 8.1 | 5 |
| Grains | Rye, baked | Inulin, oligofructose | Inulin: 0.5–0.9  Inulin midpoint: 0.70  Oligofructose: 0.5–0.9  Oligofructose midpoint: 0.70 | 1.4 | 14 | 6 |
| Grains | Wheat bran | Inulin, oligofructose | Inulin: 2.5  Oligofructose: 2.5 | 5 | 50 | 6 |
| Grains | Wheat flour, baked | Inulin, oligofructose | Inulin: 1.0–3.8  Inulin midpoint: 2.40  Oligofructose: 1.0–3.8  Oligofructose midpoint: 2.40 | 4.8 | 48 | 6 |
| Grains | Wheat flour, boiled | Inulin, oligofructose | Inulin: 0.2–0.6  Inulin midpoint: 0.40  Oligofructose: 0.2–0.6  Oligofructose midpoint: 0.40 | 0.8 | 8 | 6 |
| Legumes | Chickpea, cooked/canned | Raffinose, stachyose | Raffinose: 0.68  Stachyose: 0.57  GOS total: 1.25 | 1.25 | 12.5 | 37 |
| Legumes | Cowpeas | Raffinose, stachyose | Raffinose: 1.19  Stachyose: 3.88  GOS total: 5.07 | 5.07 | 50.7 | 38 |
| Legumes | Kidney beans (boiled) | Raffinose, stachyose, FOS | FOS: 0.51  Raffinose: 0.28  Stachyose: 1.16  GOS total: 1.44 | 1.95 | 19.5 | 34 |
| Legumes | Lima beans (cooked) | Raffinose, stachyose | Raffinose: 0.18  Stachyose: 1.16  GOS total: 1.34 | 1.3 | 13 | 34 |
| Legumes | Soybeans (boiled) | Raffinose, stachyose | Raffinose: 0.15  Stachyose: 0.64  GOS total: 0.79 | 0.79 | 7.9 | 34 |
| Legumes | Split peas (boiled) | Raffinose, stachyose | Raffinose: 0.33  Stachyose: 1.55  GOS total: 1.88 | 1.88 | 18.8 | 34 |
| Sweeteners | Honey | FOS | FOS: 0.75 | 0.75 | 7.5 | 39 |
| Vegetables | Asparagus, boiled | Inulin, oligofructose | Inulin: 1.4–2.0  Inulin midpoint: 1.70  Oligofructose: 1.4–2.0  Oligofructose midpoint: 1.70 | 3.4 | 34 | 6 |
| Vegetables | Asparagus, raw | Inulin, oligofructose | Inulin: 2.0–3.0  Inulin midpoint: 2.50  Oligofructose: 2.0–3.0  Oligofructose midpoint: 2.50 | 5 | 50 | 6 |
| Vegetables | Beets | Inulin-type fructans | Inulin-type fructans: 0.60 | 0.6 | 6 | 36 |
| Vegetables | Broccoli | FOS | FOS: 0.78 | 0.78 | 7.8 | 5 |
| Vegetables | Brussels sprouts | FOS | FOS: 0.82 | 0.82 | 8.2 | 5 |
| Vegetables | Cabbage, common | FOS | FOS: 0.82 | 0.82 | 8.2 | 5 |
| Vegetables | Cabbage, red | FOS | FOS: 0.61 | 0.61 | 6.1 | 5 |
| Vegetables | Chicory root | Inulin, oligofructose | Inulin: 35.7–47.6  Inulin midpoint: 41.65  Oligofructose: 19.6–26.2  Oligofructose midpoint: 22.90 | 64.55 | 645.5 | 6 |
| Vegetables | Dandelion greens, cooked | Inulin, oligofructose | Inulin: 8.1–10.1  Inulin midpoint: 9.10  Oligofructose: 6.5–8.1  Oligofructose midpoint: 7.30 | 16.4 | 164 | 6 |
| Vegetables | Dandelion greens, raw | Inulin, oligofructose | Inulin: 12.0–15.0  Inulin midpoint: 13.50  Oligofructose: 9.6–12.0  Oligofructose midpoint: 10.80 | 24.3 | 243 | 6 |
| Vegetables | Fennel | FOS | FOS: 0.61 | 0.61 | 6.1 | 5 |
| Vegetables | Garlic, dried | Inulin, oligofructose | Inulin: 20.3–36.1  Inulin midpoint: 28.20  Oligofructose: 8.1–14.5  Oligofructose midpoint: 11.30 | 39.5 | 395 | 6 |
| Vegetables | Garlic, raw | FOS, GOS, inulin-type fructan, inulin, oligofructose | FOS: 1.20  GOS: 0.56  Inulin-type fructan: 19.4–29.2  Inulin-type fructan midpoint: 24.30  Inulin: 9.0–16.0  Inulin midpoint: 12.50 | 43.56 | 435.6 | 5, 35, 6 |
| Vegetables | Globe artichoke | Inulin | Inulin: 2.0–6.8  Inulin MIDPOINT: 4.40 | 4.4 | 44 | 6 |
| Vegetables | Jerusalem artichoke | Inulin-type fructan, inulin, oligofructose, GOS, FOS | Inulin-type fructan: 19.4–29.2  Inulin-type fructan midpoint: 24.30  Inulin: 16.0–20.0  Inulin midpoint: 18.00  Oligofructose: 12.0–15.0  Oligofructose midpoint: 13.50 | 57.42 | 574.2 | 35, 6, 5 |
| Vegetables | Kale | FOS | FOS: 0.51 | 0.51 | 5.1 | 5 |
| Vegetables | Leek | FOS, inulin, oligofructose | FOS: 1.07  Inulin: 3.0–10.0  Inulin midpoint: 6.50  Oligofructose: 2.4–8.0  Oligofructose midpoint: 5.20 | 12.77 | 127.7 | 5, 6 |
| Vegetables | Lettuce | FOS | FOS: 0.50 | 0.5 | 5 | 4 |
| Vegetables | Onion | FOS, inulin, oligofructose | FOS: 2.01  Inulin: 1.1–7.5  Inulin midpoint: 4.30  Oligofructose: 1.1–7.5  Oligofructose midpoint: 4.30 | 10.61 | 106.1 | 5, 6 |
| Vegetables | Onion, cooked | Inulin, oligofructose | Inulin: 0.8–5.3  Inulin midpoint: 3.05  Oligofructose: 0.8–5.3  Oligofructose midpoint: 3.05 | 7.1 | 71 | 6 |
| Vegetables | Onion, dried | Inulin, oligofructose | Inulin: 4.7–31.9  Inulin midpoint: 18.30  Oligofructose: 4.7–31.9  Oligofructose midpoint: 18.30 | 39.65 | 396.5 | 6 |
| Vegetables | Shallot | Inulin-type fructan | Inulin-type fructan: 3.6–8.8 | 6.2 | 62 | 35 |
| Vegetables | Snow peas | Nystose, kestose | Nystose: 0.51  Kestose: 0.13  FOS total: 0.64 | 0.64 | 6.4 | 37 |

Abbreviations: ISAPP, International Scientific Association for Probiotics and Prebiotics; FOS, fructooligosaccharides; GOS, galactooligosaccharides. Values are from source-food composition literature and are expressed per 100 g food unless otherwise indicated. Total estimated prebiotic content was calculated by summing available values for ISAPP-recognized prebiotic types assigned to each source food. Reference IDs correspond to the final manuscript reference list.
