# Supplementary material for: Estimated prebiotic intake and depression in US adults: repeated cross-sectional analyses of NHANES 2015–2018 and 2021–2023
Source: J Nutr Sci. 2026 Jul 31;15:e70. doi: 10.1017/jns.2026.10136 (PMC13439025; doi:10.1017/jns.2026.10136)
Supplement: Supplementary file 2 [file S2048679026101360sup002.docx]

**Supplementary Table 2**
**FNDDS food-code estimated prebiotic values for NHANES 2015-2018 and 2021-2023**

| **FNDDS food code** | **2015-2018 food description** | **2015-2018 estimated prebiotic content, mg/g food** | **2021-2023 food description** | **2021-2023 estimated prebiotic content, mg/g food** |
| --- | --- | --- | --- | --- |
| 11440020 | Dill dip, yogurt based | 0.785 |  |  |
| 11440030 | Onion dip, yogurt based | 6.177 | Onion dip, yogurt based | 6.177 |
| 11440060 | Tzatziki dip | 1.427 | Tzatziki dip | 1.427 |
| 11440070 | Vegetable dip, yogurt based | 0.785 | Vegetable dip, yogurt based | 0.785 |
| 11461200 | Yogurt, frozen, sandwich | 7.337 | Frozen yogurt sandwich | 7.337 |
| 11551050 | Licuado or Batido | 1.763 | Licuado or Batido | 1.763 |
| 11553100 | Fruit smoothie, NFS | 2.191 | Fruit smoothie, NFS | 2.191 |
| 11553110 | Fruit smoothie, with whole fruit and dairy | 2.190 | Fruit smoothie, with whole fruit and dairy | 2.190 |
| 11553120 | Fruit smoothie, with whole fruit and dairy, added protein | 0.938 | Fruit smoothie, with whole fruit and dairy, added protein | 0.938 |
| 11553130 |  |  | Fruit smoothie juice drink, with dairy | 10.000 |
| 12350210 | Dill dip, regular | 0.787 |  |  |
| 12350215 | Dill dip, light | 0.747 |  |  |
| 12350220 | Onion dip, regular | 6.191 | Onion dip, regular | 6.191 |
| 12350225 | Onion dip, light | 5.941 | Onion dip, light | 5.941 |
| 12350250 | Vegetable dip, regular | 0.787 | Vegetable dip, regular | 0.787 |
| 12350255 | Vegetable dip, light | 0.747 | Vegetable dip, light | 0.747 |
| 13120300 | Ice cream bar, cake covered | 14.400 |  |  |
| 13120400 | Ice cream bar or stick with fruit | 2.244 |  |  |
| 13121120 |  |  | Banana split | 2.449 |
| 13121500 | Ice cream sundae, fudge topping, with cake, with whipped cream | 1.994 |  |  |
| 13122500 | Ice cream pie, with cookie crust, fudge topping, and whipped cream | 8.204 |  |  |
| 13126000 | Ice cream, fried | 2.926 | Ice cream, fried | 2.926 |
| 13135010 | Ice cream sandwich, made with light chocolate ice cream | 6.960 | Light ice cream sandwich, chocolate | 6.960 |
| 13161500 | Milk dessert sandwich bar, frozen, made from lowfat milk | 6.960 |  |  |
| 13161520 | Milk dessert sandwich bar, frozen, with low-calorie sweetener, made from lowfat milk | 9.523 |  |  |
| 13170000 | Baked Alaska | 3.603 |  |  |
| 13210110 | Pudding, bread | 6.475 | Pudding, bread | 6.475 |
| 13210150 | Bread pudding made with evaporated milk and rum | 13.070 |  |  |
| 13210160 | Diplomat pudding, Puerto Rican style | 4.648 |  |  |
| 13210180 | Pudding, Mexican bread | 10.346 |  |  |
| 13210190 | Pudding, Mexican bread, low fat | 19.279 |  |  |
| 13210810 | Pumpkin pudding, Puerto Rican style | 1.968 |  |  |
| 13241000 | Pudding, with fruit and vanilla wafers | 4.341 | Banana pudding | 4.341 |
| 13250100 | Mousse, not chocolate | 1.326 |  |  |
| 13252590 |  |  | Trifle | 1.400 |
| 13252600 | Tiramisu | 3.987 | Tiramisu | 3.987 |
| 13411000 | White sauce, milk sauce | 2.591 | White sauce or gravy | 2.591 |
| 13412000 | Milk gravy, quick gravy | 2.157 |  |  |
| 14202010 |  |  | Cheese, cottage, with fruit | 0.840 |
| 14610210 |  |  | Cheese, cottage cheese, with gelatin dessert and fruit | 2.023 |
| 14610250 | Cheese, cottage cheese, with gelatin dessert and vegetables | 0.151 | Cheese, cottage cheese, with gelatin dessert and vegetables | 0.151 |
| 14620110 | Artichoke dip | 10.742 | Artichoke dip | 10.742 |
| 14620115 |  |  | Spinach and artichoke dip | 5.828 |
| 14620130 | Seafood dip | 10.742 | Seafood dip | 10.742 |
| 14620200 | Cheese dip | 4.644 | Cheese dip | 4.644 |
| 14620300 |  |  | Topping from cheese pizza | 27.481 |
| 14620310 | Topping from vegetable pizza | 10.676 | Topping from vegetable pizza | 10.676 |
| 14620320 |  |  | Topping from meat pizza | 17.391 |
| 14620330 | Topping from meat and vegetable pizza | 8.755 | Topping from meat and vegetable pizza | 8.755 |
| 14630100 | Cheese fondue | 0.552 | Cheese fondue | 0.552 |
| 14630200 | Cheese souffle | 2.060 | Cheese souffle | 2.060 |
| 14630300 | Welsh rarebit | 0.964 | Welsh rarebit | 0.964 |
| 14640000 | Cheese sandwich, NFS | 28.235 | Cheese sandwich, NFS | 28.235 |
| 14640002 | Cheese sandwich, American cheese, on white bread, no spread | 28.235 | Cheese sandwich, American cheese, on white bread | 28.235 |
| 14640004 | Cheese sandwich, American cheese, on wheat bread, no spread | 28.235 | Cheese sandwich, American cheese, on wheat bread | 28.235 |
| 14640006 | Cheese sandwich, American cheese, on whole wheat bread, no spread | 28.235 |  |  |
| 14640008 | Cheese sandwich, Cheddar cheese, on white bread, no spread | 28.235 | Cheese sandwich, cheddar cheese, on white bread | 28.235 |
| 14640010 | Cheese sandwich, Cheddar cheese, on wheat bread, no spread | 28.235 | Cheese sandwich, cheddar cheese, on wheat bread | 28.235 |
| 14640012 | Cheese sandwich, Cheddar cheese, on whole wheat bread, no spread | 28.235 |  |  |
| 14640014 | Cheese sandwich, reduced fat American cheese, on white bread, no spread | 28.235 | Cheese sandwich, reduced fat cheese, on white bread | 28.235 |
| 14640016 | Cheese sandwich, reduced fat American cheese, on wheat bread, no spread | 28.235 | Cheese sandwich, reduced fat cheese, on wheat bread | 28.235 |
| 14640018 | Cheese sandwich, reduced fat American cheese, on whole wheat bread, no spread | 28.235 |  |  |
| 14640020 | Cheese sandwich, reduced fat Cheddar cheese, on white bread, no spread | 28.235 |  |  |
| 14640022 | Cheese sandwich, reduced fat Cheddar cheese, on wheat bread, no spread | 28.235 |  |  |
| 14640024 | Cheese sandwich, reduced fat Cheddar cheese, on whole wheat bread, no spread | 28.235 |  |  |
| 14640026 | Cheese sandwich, American cheese, on white bread, with mayonnaise | 24.828 |  |  |
| 14640028 | Cheese sandwich, American cheese, on wheat bread, with mayonnaise | 24.828 |  |  |
| 14640030 | Cheese sandwich, American cheese, on whole wheat bread, with mayonnaise | 24.828 |  |  |
| 14640032 | Cheese sandwich, Cheddar cheese, on white bread, with mayonnaise | 24.828 |  |  |
| 14640034 | Cheese sandwich, Cheddar cheese, on wheat bread, with mayonnaise | 24.828 |  |  |
| 14640036 | Cheese sandwich, Cheddar cheese, on whole wheat bread, with mayonnaise | 24.828 |  |  |
| 14640038 | Cheese sandwich, reduced fat American cheese, on white bread, with mayonnaise | 24.828 |  |  |
| 14640040 | Cheese sandwich, reduced fat American cheese,, on wheat bread, with mayonnaise | 24.828 |  |  |
| 14640042 | Cheese sandwich, reduced fat American cheese, on whole wheat bread, with mayonnaise | 24.828 |  |  |
| 14640044 | Cheese sandwich, reduced fat Cheddar cheese, on white bread, with mayonnaise | 24.828 |  |  |
| 14640046 | Cheese sandwich, reduced fat Cheddar cheese, on wheat bread, with mayonnaise | 24.828 |  |  |
| 14640048 | Cheese sandwich, reduced fat Cheddar cheese, on whole wheat bread, with mayonnaise | 24.828 |  |  |
| 14640050 | Cheese sandwich, American cheese, on white bread, with butter | 24.828 |  |  |
| 14640052 | Cheese sandwich, American cheese, on wheat bread, with butter | 24.828 |  |  |
| 14640054 | Cheese sandwich, American cheese, on whole wheat bread, with butter | 24.828 |  |  |
| 14640056 | Cheese sandwich, Cheddar cheese, on white bread, with butter | 24.828 |  |  |
| 14640058 | Cheese sandwich, Cheddar cheese, on wheat bread, with butter | 24.828 |  |  |
| 14640060 | Cheese sandwich, Cheddar cheese, on whole wheat bread, with butter | 24.828 |  |  |
| 14640062 | Cheese sandwich, reduced fat American cheese, on white bread, with butter | 24.828 |  |  |
| 14640064 | Cheese sandwich, reduced fat American cheese, on wheat bread, with butter | 24.828 |  |  |
| 14640066 | Cheese sandwich, reduced fat American cheese, on whole wheat bread, with butter | 24.828 |  |  |
| 14640068 | Cheese sandwich, reduced fat Cheddar cheese, on white bread, with butter | 24.828 |  |  |
| 14640070 | Cheese sandwich, reduced fat Cheddar cheese, on wheat bread, with butter | 24.828 |  |  |
| 14640072 | Cheese sandwich, reduced fat Cheddar cheese, on whole wheat bread, with butter | 24.828 |  |  |
| 14640100 | Grilled cheese sandwich, NFS | 24.828 | Grilled cheese sandwich, NFS | 24.828 |
| 14640105 | Grilled cheese sandwich, American cheese, on white bread | 24.828 | Grilled cheese sandwich, American cheese, on white bread | 24.828 |
| 14640110 | Grilled cheese sandwich, American cheese, on wheat bread | 24.828 | Grilled cheese sandwich, American cheese, on wheat bread | 24.828 |
| 14640115 | Grilled cheese sandwich, American cheese, on whole wheat bread | 24.828 |  |  |
| 14640125 | Grilled cheese sandwich, Cheddar cheese, on white bread | 24.828 | Grilled cheese sandwich, cheddar cheese, on white bread | 24.828 |
| 14640130 | Grilled cheese sandwich, Cheddar cheese, on wheat bread | 24.828 | Grilled cheese sandwich, cheddar cheese, on wheat bread | 24.828 |
| 14640135 | Grilled cheese sandwich, Cheddar cheese, on whole wheat bread | 24.828 |  |  |
| 14640155 | Grilled cheese sandwich, reduced fat American cheese, on white bread | 24.828 | Grilled cheese sandwich, reduced fat cheese, on white bread | 24.828 |
| 14640160 | Grilled cheese sandwich, reduced fat American cheese, on wheat bread | 24.828 | Grilled cheese sandwich, reduced fat cheese, on wheat bread | 24.828 |
| 14640165 | Grilled cheese sandwich, reduced fat American cheese, on whole wheat bread | 24.828 |  |  |
| 14640185 | Grilled cheese sandwich, reduced fat Cheddar cheese, on white bread | 24.828 |  |  |
| 14640190 | Grilled cheese sandwich, reduced fat Cheddar cheese, on wheat bread | 24.828 |  |  |
| 14640195 | Grilled cheese sandwich, reduced fat Cheddar cheese, on whole wheat bread | 24.828 |  |  |
| 14650100 | Cheese sauce | 4.644 | Cheese sauce | 4.644 |
| 14650150 | Cheese sauce made with lowfat cheese | 1.650 |  |  |
| 14710200 | Beer cheese soup, made with milk | 2.768 |  |  |
| 21103110 | Beef steak, breaded or floured, baked or fried, NS as to fat eaten | 6.365 |  |  |
| 21103120 | Beef steak, breaded or floured, baked or fried, lean and fat eaten | 6.365 |  |  |
| 21103130 | Beef steak, breaded or floured, baked or fried, lean only eaten | 6.365 |  |  |
| 21104110 | Beef steak, battered, fried, NS as to fat eaten | 4.886 | Beef, steak, country fried | 4.886 |
| 21104120 | Beef steak, battered, fried, lean and fat eaten | 5.405 |  |  |
| 21104130 | Beef steak, battered, fried, lean only eaten | 5.405 |  |  |
| 22000300 | Pork, NS as to cut, breaded or floured, fried, NS as to fat eaten | 8.072 |  |  |
| 22000310 | Pork, NS as to cut, breaded or floured, fried, lean and fat eaten | 8.072 |  |  |
| 22000320 | Pork, NS as to cut, breaded or floured, fried, lean only eaten | 8.072 |  |  |
| 22002100 | Pork, ground or patty, breaded, cooked | 6.787 |  |  |
| 22101130 | Pork chop, breaded or floured, broiled or baked, NS as to fat eaten | 4.413 |  |  |
| 22101140 | Pork chop, breaded or floured, broiled or baked, lean and fat eaten | 4.413 |  |  |
| 22101150 | Pork chop, breaded or floured, broiled or baked, lean only eaten | 4.413 |  |  |
| 22101300 | Pork chop, breaded or floured, fried, NS as to fat eaten | 8.072 | Pork, chop, coated, NS as to fat eaten | 8.072 |
| 22101310 | Pork chop, breaded or floured, fried, lean and fat eaten | 8.072 | Pork, chop, coated, lean and fat eaten | 8.072 |
| 22101320 | Pork chop, breaded or floured, fried, lean only eaten | 8.072 | Pork, chop, coated, lean only eaten | 8.072 |
| 22101400 | Pork chop, battered, fried, NS as to fat eaten | 5.292 |  |  |
| 22101410 | Pork chop, battered, fried, lean and fat eaten | 5.292 |  |  |
| 22101420 | Pork chop, battered, fried, lean only eaten | 5.292 |  |  |
| 22201050 | Pork steak or cutlet, battered, fried, NS as to fat eaten | 2.938 |  |  |
| 22201060 | Pork steak or cutlet, battered, fried, lean and fat eaten | 2.938 |  |  |
| 22201070 | Pork steak or cutlet, battered, fried, lean only eaten | 2.938 |  |  |
| 22201300 | Pork steak or cutlet, breaded or floured, broiled or baked, NS as to fat eaten | 4.413 |  |  |
| 22201310 | Pork steak or cutlet, breaded or floured, broiled or baked, lean and fat eaten | 4.413 |  |  |
| 22201320 | Pork steak or cutlet, breaded or floured, broiled or baked, lean only eaten | 4.413 |  |  |
| 22201400 | Pork steak or cutlet, breaded or floured, fried, NS as to fat eaten | 8.247 | Pork, steak, coated | 8.247 |
| 22201410 | Pork steak or cutlet, breaded or floured, fried, lean and fat eaten | 8.072 |  |  |
| 22201420 | Pork steak or cutlet, breaded or floured, fried, lean only eaten | 8.072 |  |  |
| 22210310 | Pork, tenderloin, breaded, fried | 7.575 |  |  |
| 22210450 | Pork, tenderloin, battered, fried | 2.938 |  |  |
| 22300150 | Ham, breaded or floured, fried, NS as to fat eaten | 4.064 |  |  |
| 22300160 | Ham, breaded or floured, fried, lean and fat eaten | 4.064 |  |  |
| 22300170 | Ham, breaded or floured, fried, lean only eaten | 3.923 |  |  |
| 22402510 | Fried pork chunks, Puerto Rican style | 2.495 |  |  |
| 23220020 | Mock chicken legs, cooked | 2.649 | Mock chicken legs | 2.649 |
| 23220030 | Veal patty, breaded, cooked | 4.546 |  |  |
| 23311120 | Rabbit, NS as to domestic or wild, breaded, fried | 3.377 |  |  |
| 23321250 | Venison/deer steak, breaded or floured, cooked, NS as to cooking method | 8.134 |  |  |
| 24127200 | Chicken breast, fried, coated, skin / coating eaten, from raw | 7.200 | Chicken breast, fried, coated, skin / coating eaten, from raw | 7.200 |
| 24127202 | Chicken breast, fried, coated, prepared skinless, coating eaten, from raw | 7.200 | Chicken breast, fried, coated, prepared skinless, coating eaten, from raw | 7.200 |
| 24127210 | Chicken breast, fried, coated, skin / coating eaten, from pre-cooked | 8.631 | Chicken breast, fried, coated, skin / coating eaten, from pre-cooked | 8.631 |
| 24127220 | Chicken breast, fried, coated, skin / coating eaten, from fast food / restaurant | 7.200 | Chicken breast, fried, coated, skin / coating eaten, from fast food / restaurant | 7.200 |
| 24127221 | Chicken breast, fried, coated, skin / coating not eaten, from fast food / restaurant | 7.200 | Chicken breast, fried, coated, skin / coating not eaten, from fast food / restaurant | 7.200 |
| 24127500 | Chicken breast, baked, coated, skin / coating eaten | 4.800 | Chicken breast, baked, coated, skin / coating eaten | 4.800 |
| 24137300 | Chicken leg, drumstick and thigh, fried, coated, skin / coating eaten | 7.157 | Chicken leg, drumstick and thigh, fried, coated, skin / coating eaten | 7.157 |
| 24137310 | Chicken leg, drumstick and thigh, baked, coated, skin / coating eaten | 4.762 | Chicken leg, drumstick and thigh, baked, coated, skin / coating eaten | 4.762 |
| 24147300 | Chicken drumstick, fried, coated, skin / coating eaten, from raw | 7.157 | Chicken drumstick, fried, coated, skin / coating eaten, from raw | 7.157 |
| 24147302 | Chicken drumstick, fried, coated, prepared skinless, coating eaten, from raw | 7.143 | Chicken drumstick, fried, coated, prepared skinless, coating eaten, from raw | 7.143 |
| 24147310 | Chicken drumstick, fried, coated, skin / coating eaten, from pre-cooked | 8.563 | Chicken drumstick, fried, coated, skin / coating eaten, from pre-cooked | 8.563 |
| 24147320 | Chicken drumstick, fried, coated, skin / coating eaten, from fast food / restaurant | 7.325 | Chicken drumstick, fried, coated, skin / coating eaten, from fast food / restaurant | 7.325 |
| 24147321 | Chicken drumstick, fried, coated, skin / coating not eaten, from fast food / restaurant | 7.325 | Chicken drumstick, fried, coated, skin / coating not eaten, from fast food / restaurant | 7.325 |
| 24147400 | Chicken drumstick, baked, coated, skin / coating eaten | 4.762 | Chicken drumstick, baked, coated, skin / coating eaten | 4.762 |
| 24157300 | Chicken thigh, fried, coated, skin / coating eaten, from raw | 7.157 | Chicken thigh, fried, coated, skin / coating eaten, from raw | 7.157 |
| 24157302 | Chicken thigh, fried, coated, prepared skinless, coating eaten, from raw | 7.143 | Chicken thigh, fried, coated, prepared skinless, coating eaten, from raw | 7.143 |
| 24157310 | Chicken thigh, fried, coated, skin / coating eaten, from pre-cooked | 8.563 | Chicken thigh, fried, coated, skin / coating eaten, from pre-cooked | 8.563 |
| 24157320 | Chicken thigh, fried, coated, skin / coating eaten, from fast food | 7.325 | Chicken thigh, fried, coated, skin / coating eaten, from fast food | 7.325 |
| 24157321 | Chicken thigh, fried, coated, skin / coating not eaten, from fast food | 7.325 | Chicken thigh, fried, coated, skin / coating not eaten, from fast food | 7.325 |
| 24157330 | Chicken thigh, fried, coated, skin / coating eaten, from restaurant | 7.325 | Chicken thigh, fried, coated, skin / coating eaten, from restaurant | 7.325 |
| 24157331 | Chicken thigh, fried, coated, skin / coating not eaten, from restaurant | 7.325 | Chicken thigh, fried, coated, skin / coating not eaten, from restaurant | 7.325 |
| 24157400 | Chicken thigh, baked, coated, skin / coating eaten | 4.762 | Chicken thigh, baked, coated, skin / coating eaten | 4.762 |
| 24167200 | Chicken wing, fried, coated, from raw | 7.143 | Chicken wing, fried, coated, from raw | 7.143 |
| 24167210 | Chicken wing, fried, coated, from pre-cooked | 8.563 | Chicken wing, fried, coated, from pre-cooked | 8.563 |
| 24167220 | Chicken wing, fried, coated, from fast food | 7.325 | Chicken wing, fried, coated, from fast food | 7.325 |
| 24167230 | Chicken wing, fried, coated, from restaurant | 7.325 | Chicken wing, fried, coated, from restaurant | 7.325 |
| 24167300 | Chicken wing, baked, coated | 4.762 | Chicken wing, baked, coated | 4.762 |
| 24168030 | Chicken "wings", boneless, with hot sauce, from fast food / restaurant | 2.386 | Chicken "wings", boneless, with hot sauce, from fast food / restaurant | 2.386 |
| 24168031 | Chicken "wings", boneless, with hot sauce, from other sources | 2.386 | Chicken "wings", boneless, with hot sauce, from other sources | 2.386 |
| 24198840 | Fried chicken chunks, Puerto Rican style | 7.904 |  |  |
| 24201060 | Turkey, light meat, breaded, baked or fried, skin not eaten | 4.305 | Turkey, light meat, breaded, baked or fried, skin not eaten | 4.305 |
| 24201070 | Turkey, light meat, breaded, baked or fried, skin eaten | 4.305 | Turkey, light meat, breaded, baked or fried, skin eaten | 4.305 |
| 24201360 | Turkey, light or dark meat, fried, coated, skin not eaten | 0.792 | Turkey, light or dark meat, fried, coated, skin not eaten | 0.792 |
| 24201370 | Turkey, light or dark meat, fried, coated, skin eaten | 0.792 | Turkey, light or dark meat, fried, coated, skin eaten | 0.792 |
| 24208000 | Turkey, nuggets | 11.429 | Turkey, nuggets | 11.429 |
| 24301210 | Duck, coated, fried | 2.955 |  |  |
| 24302010 | Duck, pressed, Chinese | 4.567 | Duck, Peking | 4.567 |
| 24402110 | Dove, fried | 0.818 | Dove, fried | 0.818 |
| 25110450 | Chicken liver, fried | 2.810 | Liver, chicken | 2.810 |
| 25160130 | Tongue pot roast, Puerto Rican style | 0.416 | Tongue pot roast, Puerto Rican style | 0.416 |
| 26100130 | Fish, NS as to type, coated, baked or broiled, made with oil | 5.986 | Fish, NS as to type, baked or broiled, coated | 5.986 |
| 26100131 | Fish, NS as to type, coated, baked or broiled, made with butter | 5.975 |  |  |
| 26100132 | Fish, NS as to type, coated, baked or broiled, made with margarine | 5.972 |  |  |
| 26100133 | Fish, NS as to type, coated, baked or broiled, made without fat | 6.259 |  |  |
| 26100134 | Fish, NS as to type, coated, baked or broiled, made with cooking spray | 6.241 |  |  |
| 26100140 | Fish, NS as to type, coated, fried, made with oil | 5.528 | Fish, NS as to type, fried | 5.528 |
| 26100141 | Fish, NS as to type, coated, fried, made with butter | 5.528 |  |  |
| 26100142 | Fish, NS as to type, coated, fried, made with margarine | 5.528 |  |  |
| 26100143 | Fish, NS as to type, coated, fried, made without fat | 5.971 |  |  |
| 26100144 | Fish, NS as to type, coated, fried, made with cooking spray | 5.952 |  |  |
| 26103130 | Barracuda, coated, baked or broiled, fat added in cooking | 5.986 |  |  |
| 26103131 | Barracuda, coated, baked or broiled, fat not added in cooking | 6.259 |  |  |
| 26103140 | Barracuda, coated, fried | 5.528 |  |  |
| 26105110 | Carp, cooked, NS as to cooking method | 5.528 | Fish, carp | 5.528 |
| 26105130 | Carp, coated, baked or broiled, fat added in cooking | 5.986 |  |  |
| 26105131 | Carp, coated, baked or broiled, fat not added in cooking | 6.259 |  |  |
| 26105140 | Carp, coated, fried | 5.528 |  |  |
| 26107110 | Catfish, cooked, NS as to cooking method | 5.531 | Fish, catfish, NFS | 5.531 |
| 26107130 | Catfish, coated, baked or broiled, made with oil | 5.986 | Fish, catfish, baked or broiled, coated | 5.986 |
| 26107131 | Catfish, coated, baked or broiled, made with butter | 5.975 |  |  |
| 26107132 | Catfish, coated, baked or broiled, made with margarine | 5.972 |  |  |
| 26107133 | Catfish, coated, baked or broiled, made without fat | 6.259 |  |  |
| 26107134 | Catfish, coated, baked or broiled, made with cooking spray | 6.241 |  |  |
| 26107140 | Catfish, coated, fried, made with oil | 5.528 | Fish, catfish, fried | 5.528 |
| 26107141 | Catfish, coated, fried, made with butter | 5.528 |  |  |
| 26107142 | Catfish, coated, fried, made with margarine | 5.528 |  |  |
| 26107143 | Catfish, coated, fried, made without fat | 5.971 |  |  |
| 26107144 | Catfish, coated, fried, made with cooking spray | 5.952 |  |  |
| 26109130 | Cod, coated, baked or broiled, made with oil | 5.986 | Fish, cod, baked or broiled, coated | 5.986 |
| 26109131 | Cod, coated, baked or broiled, made with butter | 5.975 |  |  |
| 26109132 | Cod, coated, baked or broiled, made with margarine | 5.972 |  |  |
| 26109133 | Cod, coated, baked or broiled, made without fat | 6.259 |  |  |
| 26109134 | Cod, coated, baked or broiled, made with cooking spray | 6.241 |  |  |
| 26109140 | Cod, coated, fried, made with oil | 5.528 | Fish, cod, fried | 5.528 |
| 26109141 | Cod, coated, fried, made with butter | 5.528 |  |  |
| 26109142 | Cod, coated, fried, made with margarine | 5.528 |  |  |
| 26109143 | Cod, coated, fried, made without fat | 5.971 |  |  |
| 26109144 | Cod, coated, fried, made with cooking spray | 5.952 |  |  |
| 26111130 | Croaker, coated, baked or broiled, fat added in cooking | 5.986 |  |  |
| 26111131 | Croaker, coated, baked or broiled, fat not added in cooking | 6.259 |  |  |
| 26111140 | Croaker, coated, fried | 5.528 |  |  |
| 26113110 | Eel, cooked, NS as to cooking method | 4.879 | Fish, eel | 4.879 |
| 26115110 | Flounder, cooked, NS as to cooking method | 5.531 | Fish, flounder, NFS | 5.531 |
| 26115130 | Flounder, coated, baked or broiled, made with oil | 5.986 | Fish, flounder, baked or broiled, coated | 5.986 |
| 26115131 | Flounder, coated, baked or broiled, made with butter | 5.975 |  |  |
| 26115132 | Flounder, coated, baked or broiled, made with margarine | 5.972 |  |  |
| 26115133 | Flounder, coated, baked or broiled, made without fat | 6.259 |  |  |
| 26115134 | Flounder, coated, baked or broiled, made with cooking spray | 6.241 |  |  |
| 26115140 | Flounder, coated, fried, made with oil | 5.528 | Fish, flounder, fried | 5.528 |
| 26115141 | Flounder, coated, fried, made with butter | 5.528 |  |  |
| 26115142 | Flounder, coated, fried, made with margarine | 5.528 |  |  |
| 26115143 | Flounder, coated, fried, made without fat | 5.971 |  |  |
| 26115144 | Flounder, coated, fried, made with cooking spray | 5.952 |  |  |
| 26117130 | Haddock, coated, baked or broiled, fat added in cooking | 5.986 | Fish, haddock, baked or broiled, coated | 5.986 |
| 26117131 | Haddock, coated, baked or broiled, fat not added in cooking | 6.259 |  |  |
| 26117140 | Haddock, coated, fried | 5.528 | Fish, haddock, fried | 5.528 |
| 26118030 | Halibut, coated, baked or broiled, made with oil | 5.986 |  |  |
| 26118031 | Halibut, coated, baked or broiled, made with butter | 5.975 |  |  |
| 26118032 | Halibut, coated, baked or broiled, made with margarine | 5.972 |  |  |
| 26118033 | Halibut, coated, baked or broiled, made without fat | 6.259 |  |  |
| 26118034 | Halibut, coated, baked or broiled, made with cooking spray | 6.241 |  |  |
| 26118040 | Halibut, coated, fried, made with oil | 5.528 |  |  |
| 26118041 | Halibut, coated, fried, made with butter | 5.528 |  |  |
| 26118042 | Halibut, coated, fried, made with margarine | 5.528 |  |  |
| 26118043 | Halibut, coated, fried, made without fat | 5.971 |  |  |
| 26118044 | Halibut, coated, fried, made with cooking spray | 5.952 |  |  |
| 26119130 | Herring, coated, baked or broiled, fat added in cooking | 5.986 |  |  |
| 26119131 | Herring, coated, baked or broiled, fat not added in cooking | 6.259 |  |  |
| 26119140 | Herring, coated, fried | 5.528 |  |  |
| 26121131 | Mackerel, coated, baked or broiled, fat added in cooking | 5.983 | Fish, mackerel, baked or broiled, coated | 5.983 |
| 26121132 | Mackerel, coated, baked or broiled, fat not added in cooking | 6.257 |  |  |
| 26121140 | Mackerel, coated, fried | 5.526 | Fish, mackerel, fried | 5.526 |
| 26121160 | Mackerel, pickled | 28.754 |  |  |
| 26123110 | Mullet, cooked, NS as to cooking method | 5.531 | Fish, mullet | 5.531 |
| 26123130 | Mullet, coated, baked or broiled, fat added in cooking | 5.986 |  |  |
| 26123131 | Mullet, coated, baked or broiled, fat not added in cooking | 6.259 |  |  |
| 26123140 | Mullet, coated, fried | 5.528 |  |  |
| 26125130 | Ocean perch, coated, baked or broiled, fat added in cooking | 5.986 |  |  |
| 26125131 | Ocean perch, coated, baked or broiled, fat not added in cooking | 6.259 |  |  |
| 26125140 | Ocean perch, coated, fried | 5.528 |  |  |
| 26127110 | Perch, cooked, NS as to cooking method | 5.531 | Fish, perch, NFS | 5.531 |
| 26127130 | Perch, coated, baked or broiled, made with oil | 5.986 | Fish, perch, baked or broiled, coated | 5.986 |
| 26127131 | Perch, coated, baked or broiled, made with butter | 5.975 |  |  |
| 26127132 | Perch, coated, baked or broiled, made with margarine | 5.972 |  |  |
| 26127133 | Perch, coated, baked or broiled, made without fat | 6.259 |  |  |
| 26127134 | Perch, coated, baked or broiled, made with cooking spray | 6.241 |  |  |
| 26127140 | Perch, coated, fried, made with oil | 5.528 | Fish, perch, fried | 5.528 |
| 26127141 | Perch, coated, fried, made with butter | 5.528 |  |  |
| 26127142 | Perch, coated, fried, made with margarine | 5.528 |  |  |
| 26127143 | Perch, coated, fried, made without fat | 5.971 |  |  |
| 26127144 | Perch, coated, fried, made with cooking spray | 5.952 |  |  |
| 26129110 | Pike, cooked, NS as to cooking method | 7.806 | Fish, pike | 7.806 |
| 26129130 | Pike, coated, baked or broiled, fat added in cooking | 5.986 |  |  |
| 26129131 | Pike, coated, baked or broiled, fat not added in cooking | 6.259 |  |  |
| 26129140 | Pike, coated, fried | 5.528 |  |  |
| 26131130 | Pompano, coated, baked or broiled, fat added in cooking | 5.986 | Fish, pompano, baked or broiled, coated | 5.986 |
| 26131131 | Pompano, coated, baked or broiled, fat not added in cooking | 6.259 |  |  |
| 26131140 | Pompano, coated, fried | 5.528 | Fish, pompano, fried | 5.528 |
| 26133130 | Porgy, coated, baked or broiled, fat added in cooking | 5.986 |  |  |
| 26133131 | Porgy, coated, baked or broiled, fat not added in cooking | 6.259 |  |  |
| 26133140 | Porgy, coated, fried | 5.528 |  |  |
| 26135130 | Ray, coated, baked or broiled, fat added in cooking | 5.986 |  |  |
| 26135131 | Ray, coated, baked or broiled, fat not added in cooking | 6.259 |  |  |
| 26135140 | Ray, coated, fried | 5.528 |  |  |
| 26137130 | Salmon, coated, baked or broiled, made with oil | 5.986 | Fish, salmon, baked or broiled, coated | 5.986 |
| 26137131 | Salmon, coated, baked or broiled, made with butter | 5.975 |  |  |
| 26137132 | Salmon, coated, baked or broiled, made with margarine | 5.972 |  |  |
| 26137133 | Salmon, coated, baked or broiled, made without fat | 6.259 |  |  |
| 26137134 | Salmon, coated, baked or broiled, made with cooking spray | 6.241 |  |  |
| 26137140 | Salmon, coated, fried, made with oil | 5.528 | Fish, salmon, fried | 5.528 |
| 26137141 | Salmon, coated, fried, made with butter | 5.528 |  |  |
| 26137142 | Salmon, coated, fried, made with margarine | 5.528 |  |  |
| 26137143 | Salmon, coated, fried, made without fat | 5.971 |  |  |
| 26137144 | Salmon, coated, fried, made with cooking spray | 5.952 |  |  |
| 26141130 | Sea bass, coated, baked or broiled, fat added in cooking | 5.986 | Fish, bass, baked or broiled, coated | 5.986 |
| 26141131 | Sea bass, coated, baked or broiled, fat not added in cooking | 6.259 |  |  |
| 26141140 | Sea bass, coated, fried | 5.528 | Fish, bass, fried | 5.528 |
| 26141180 | Sea bass, pickled | 28.754 |  |  |
| 26143130 | Shark, coated, baked or broiled, fat added in cooking | 5.986 |  |  |
| 26143131 | Shark, coated, baked or broiled, fat not added in cooking | 6.259 |  |  |
| 26143140 | Shark, coated, fried | 5.528 |  |  |
| 26147140 | Sturgeon, coated, fried | 5.528 |  |  |
| 26149130 | Swordfish, coated, baked or broiled, fat added in cooking | 5.986 |  |  |
| 26149131 | Swordfish, coated, baked or broiled, fat not added in cooking | 6.259 |  |  |
| 26149140 | Swordfish, coated, fried | 5.528 |  |  |
| 26151130 | Trout, coated, baked or broiled, made with oil | 5.986 | Fish, trout, baked or broiled, coated | 5.986 |
| 26151131 | Trout, coated, baked or broiled, made with butter | 5.975 |  |  |
| 26151132 | Trout, coated, baked or broiled, made with margarine | 5.972 |  |  |
| 26151133 | Trout, coated, baked or broiled, made without fat | 6.259 |  |  |
| 26151134 | Trout, coated, baked or broiled, made with cooking spray | 6.241 |  |  |
| 26151140 | Trout, coated, fried, made with oil | 5.528 | Fish, trout, fried | 5.528 |
| 26151141 | Trout, coated, fried, made with butter | 5.528 |  |  |
| 26151142 | Trout, coated, fried, made with margarine | 5.528 |  |  |
| 26151143 | Trout, coated, fried, made without fat | 5.971 |  |  |
| 26151144 | Trout, coated, fried, made with cooking spray | 5.952 |  |  |
| 26153130 | Tuna, fresh, coated, baked or broiled, fat added in cooking | 5.986 |  |  |
| 26153131 | Tuna, fresh, coated, baked or broiled, fat not added | 6.259 |  |  |
| 26153140 | Tuna, fresh, coated, fried | 5.528 |  |  |
| 26157110 | Whiting, cooked, NS as to cooking method | 5.531 | Fish, whiting, NFS | 5.531 |
| 26157130 | Whiting, coated, baked or broiled, made with oil | 5.986 | Fish, whiting, baked or broiled, coated | 5.986 |
| 26157131 | Whiting, coated, baked or broiled, made with butter | 5.975 |  |  |
| 26157132 | Whiting, coated, baked or broiled, made with margarine | 5.972 |  |  |
| 26157133 | Whiting, coated, baked or broiled, made without fat | 6.259 |  |  |
| 26157134 | Whiting, coated, baked or broiled, made with cooking spray | 6.241 |  |  |
| 26157140 | Whiting, coated, fried, made with oil | 5.528 | Fish, whiting, fried | 5.528 |
| 26157141 | Whiting, coated, fried, made with butter | 5.528 |  |  |
| 26157142 | Whiting, coated, fried, made with margarine | 5.528 |  |  |
| 26157143 | Whiting, coated, fried, made without fat | 5.971 |  |  |
| 26157144 | Whiting, coated, fried, made with cooking spray | 5.952 |  |  |
| 26158020 | Tilapia, coated, baked or broiled, made with oil | 5.986 | Fish, tilapia, baked or broiled, coated | 5.986 |
| 26158021 | Tilapia, coated, baked or broiled, made with butter | 5.975 |  |  |
| 26158022 | Tilapia, coated, baked or broiled, made with margarine | 5.972 |  |  |
| 26158023 | Tilapia, coated, baked or broiled, made without fat | 6.259 |  |  |
| 26158024 | Tilapia, coated, baked or broiled, made with cooking spray | 6.241 |  |  |
| 26158030 | Tilapia, coated, fried, made with oil | 5.528 | Fish, tilapia, fried | 5.528 |
| 26158031 | Tilapia, coated, fried, made with butter | 5.528 |  |  |
| 26158032 | Tilapia, coated, fried, made with margarine | 5.528 |  |  |
| 26158033 | Tilapia, coated, fried, made without fat | 5.971 |  |  |
| 26158034 | Tilapia, coated, fried, made with cooking spray | 5.952 |  |  |
| 26203110 | Frog legs, NS as to cooking method | 5.528 | Frog legs | 5.528 |
| 26205110 | Octopus, cooked, NS as to cooking method | 5.528 | Octopus | 5.528 |
| 26213130 | Squid, coated, baked or broiled, fat added in cooking | 5.986 |  |  |
| 26213131 | Squid, coated, baked or broiled, fat not added in cooking | 6.259 |  |  |
| 26213140 | Squid, coated, fried | 5.528 | Calamari, fried | 5.528 |
| 26301140 | Abalone, floured or breaded, fried | 5.528 |  |  |
| 26303130 | Clams, coated, baked or broiled, fat added in cooking | 5.986 |  |  |
| 26303131 | Clams, coated, baked or broiled, fat not added in cooking | 6.259 |  |  |
| 26303140 | Clams, coated, fried | 5.528 | Clams, fried | 5.528 |
| 26305130 | Crab, coated, baked or broiled, fat added in cooking | 5.986 |  |  |
| 26305131 | Crab, coated, baked or broiled, fat not added in cooking | 6.259 |  |  |
| 26307140 | Crab, soft shell, coated, fried | 5.528 | Crab, soft shell | 5.528 |
| 26309140 | Crayfish, coated, fried | 5.528 | Crayfish, fried | 5.528 |
| 26311140 | Lobster, coated, fried | 5.528 |  |  |
| 26311170 | Lobster, coated, baked or broiled, fat added in cooking | 5.986 |  |  |
| 26311171 | Lobster, coated, baked or broiled, fat not added in cooking | 6.259 |  |  |
| 26315110 | Oysters, cooked, NS as to cooking method | 5.528 |  |  |
| 26315140 | Oysters, coated, fried | 5.528 | Oysters, fried | 5.528 |
| 26315160 | Oysters, coated, baked or broiled, fat added in cooking | 5.986 |  |  |
| 26315161 | Oysters, coated, baked or broiled, fat not added in cooking | 6.259 |  |  |
| 26317140 | Scallops, coated, fried | 5.528 | Scallops, fried | 5.528 |
| 26317160 | Scallops, coated, baked or broiled, fat added in cooking | 5.986 |  |  |
| 26317161 | Scallops, coated, baked or broiled, fat not added in cooking | 6.259 |  |  |
| 26319110 | Shrimp, cooked, NS as to cooking method | 5.528 | Shrimp, NFS | 5.528 |
| 26319140 | Shrimp, coated, fried, made with oil | 5.528 | Shrimp, fried | 5.528 |
| 26319141 | Shrimp, coated, fried, made with butter | 5.528 |  |  |
| 26319142 | Shrimp, coated, fried, made with margarine | 5.528 |  |  |
| 26319143 | Shrimp, coated, fried, made without fat | 5.971 |  |  |
| 26319144 | Shrimp, coated, fried, made with cooking spray | 5.952 |  |  |
| 26319160 | Shrimp, coated, baked or broiled, made with oil | 5.986 | Shrimp, baked or broiled, coated | 5.986 |
| 26319161 | Shrimp, coated, baked or broiled, made with butter | 5.975 |  |  |
| 26319162 | Shrimp, coated, baked or broiled, made with margarine | 5.972 |  |  |
| 26319163 | Shrimp, coated, baked or broiled, made without fat | 6.259 |  |  |
| 26319164 | Shrimp, coated, baked or broiled, made with cooking spray | 6.241 |  |  |
| 27111100 | Beef goulash | 16.765 |  |  |
| 27111200 | Beef burgundy | 21.766 | Beef burgundy | 21.766 |
| 27111300 | Beef stew, no potatoes, tomato-based sauce, Mexican style | 11.947 | Guisada, beef | 11.947 |
| 27111310 | Beef stew, no potatoes, tomato-based sauce, with chili peppers, Mexican style | 8.938 |  |  |
| 27111400 | Chili con carne, NS as to beans | 12.292 | Chili, NFS | 12.292 |
| 27111405 |  |  | Chili with meat, from restaurant | 7.557 |
| 27111406 | Chili con carne with beans, home recipe | 12.294 | Chili with meat and beans | 12.294 |
| 27111407 |  |  | Chili with meat, canned | 11.071 |
| 27111410 | Chili con carne with beans | 6.141 |  |  |
| 27111420 |  |  | Chili with meat | 3.964 |
| 27111500 | Beef sloppy joe, no bun | 8.646 | Sloppy joe, no bun | 8.646 |
| 27113100 | Beef stroganoff | 7.907 | Beef stroganoff | 7.907 |
| 27113200 | Creamed chipped or dried beef | 1.665 | Creamed chipped or dried beef | 1.665 |
| 27113300 | Swedish meatballs with cream or white sauce | 6.430 | Swedish meatballs with cream or white sauce | 6.430 |
| 27115000 | Beef with soy-based sauce | 23.303 | Beef with soy-based sauce | 23.303 |
| 27116100 | Beef curry | 5.763 | Beef curry | 5.763 |
| 27116300 | Beef with sweet and sour sauce | 9.954 | Beef with sweet and sour sauce | 9.954 |
| 27116350 | Stewed seasoned ground beef, Mexican style | 4.651 | Stewed seasoned ground beef, Mexican style | 4.651 |
| 27116400 | Steak tartare | 9.279 | Steak tartare | 9.279 |
| 27118110 | Meatballs, Puerto Rican style | 13.131 | Meatballs, Puerto Rican style | 13.131 |
| 27118120 | Stewed seasoned ground beef, Puerto Rican style | 1.315 | Stewed seasoned ground beef, Puerto Rican style | 1.315 |
| 27118140 | Stuffed pot roast, Puerto Rican style, NFS | 6.867 |  |  |
| 27118180 | Beef stew, meat with gravy, no potatoes, Puerto Rican style | 0.518 |  |  |
| 27120060 | Sweet and sour pork | 9.954 | Sweet and sour pork | 9.954 |
| 27120080 | Ham stroganoff | 8.160 | Ham stroganoff | 8.160 |
| 27120120 | Sausage gravy | 1.716 | Sausage gravy | 1.716 |
| 27120130 | Pork stew, no potatoes, tomato-based sauce, Mexican style | 11.947 | Guisada, pork | 11.947 |
| 27120150 | Pork or ham with soy-based sauce | 23.303 | Pork or ham with soy-based sauce | 23.303 |
| 27120160 | Pork curry | 5.740 |  |  |
| 27121000 | Pork with chili and tomatoes | 5.779 | Pork with chili and tomatoes | 5.779 |
| 27121410 | Chili con carne with beans, made with pork | 12.270 |  |  |
| 27130050 | Lamb or mutton goulash | 16.765 |  |  |
| 27130100 | Lamb or mutton curry | 5.943 |  |  |
| 27133010 | Stewed goat, Puerto Rican style | 10.859 | Stewed goat, Puerto Rican style | 10.859 |
| 27135040 | Veal with butter sauce | 0.771 |  |  |
| 27135050 | Veal Marsala | 3.660 | Veal Marsala | 3.660 |
| 27135110 | Veal parmigiana | 3.475 | Veal parmigiana | 3.475 |
| 27135150 | Veal cordon bleu | 5.886 | Veal cordon bleu | 5.886 |
| 27136100 | Chili con carne with venison/deer and beans | 12.289 |  |  |
| 27141000 | Chicken or turkey cacciatore | 1.003 | Chicken or turkey cacciatore | 1.003 |
| 27141030 |  |  | Spaghetti sauce with poultry | 39.000 |
| 27141050 | Stewed chicken with tomato-based sauce, Mexican style | 6.490 | Stewed chicken with tomato-based sauce, Mexican style | 6.490 |
| 27141500 | Chili con carne with chicken or turkey and beans | 12.394 | Chili with chicken and beans | 12.394 |
| 27141505 |  |  | Chili with chicken | 3.964 |
| 27141510 |  |  | Chili, white | 3.778 |
| 27142100 | Chicken or turkey fricassee | 1.838 | Chicken or turkey fricassee | 1.838 |
| 27146150 | Chicken curry | 5.780 | Chicken curry | 5.780 |
| 27146250 | Chicken or turkey cordon bleu | 5.886 | Chicken or turkey cordon bleu | 5.886 |
| 27146300 | Chicken or turkey parmigiana | 3.475 | Chicken or turkey parmigiana | 3.475 |
| 27146400 | Chicken kiev | 2.484 | Chicken kiev | 2.484 |
| 27148010 | Stuffed chicken, drumstick or breast, Puerto Rican style | 3.646 | Stuffed chicken, drumstick or breast, Puerto Rican style | 3.646 |
| 27150020 | Crab, deviled | 6.172 |  |  |
| 27150030 | Crab imperial | 3.633 |  |  |
| 27150050 | Fish timbale or mousse | 0.489 | Fish timbale or mousse | 0.489 |
| 27150060 | Lobster newburg | 0.807 |  |  |
| 27150100 | Shrimp curry | 5.822 |  |  |
| 27150130 | Seafood newburg | 0.807 | Seafood thermidor | 0.807 |
| 27150140 | Clam sauce, white | 1.665 | Seafood sauce | 1.665 |
| 27150151 |  |  | Spaghetti sauce with seafood | 39.000 |
| 27150160 | Shrimp with lobster sauce | 1.888 | Shrimp with lobster sauce | 1.888 |
| 27150170 | Sweet and sour shrimp | 10.006 | Sweet and sour shrimp | 10.006 |
| 27150190 | Lobster sauce | 3.506 |  |  |
| 27150230 | Shrimp scampi | 1.553 | Shrimp scampi | 1.553 |
| 27150320 | Fish curry | 5.757 | Fish curry | 5.757 |
| 27151030 | Ceviche | 7.977 | Ceviche | 7.977 |
| 27151040 | Crabs in tomato-based sauce, Puerto Rican style | 11.448 | Crabs in tomato-based sauce, Puerto Rican style | 11.448 |
| 27151050 | Shrimp in garlic sauce, Puerto Rican style | 3.254 | Shrimp in garlic sauce, Puerto Rican style | 3.254 |
| 27151070 | Stewed codfish, no potatoes, Puerto Rican style | 16.311 | Stew, fish | 16.311 |
| 27161010 | Meat loaf, Puerto Rican style | 16.393 | Meat loaf, Puerto Rican style | 16.393 |
| 27162040 |  |  | Spaghetti sauce with meat | 39.000 |
| 27162500 | Stewed, seasoned, ground beef and pork, Mexican style | 5.245 | Stewed, seasoned, ground beef and pork, Mexican style | 5.245 |
| 27211100 | Beef stew with potatoes, tomato-based sauce | 6.457 |  |  |
| 27211110 | Beef stew with potatoes, tomato-based sauce, Mexican style | 9.713 |  |  |
| 27211150 | Beef goulash with potatoes | 11.842 |  |  |
| 27211200 | Beef stew with potatoes, gravy | 7.042 |  |  |
| 27211300 | Beef, roast, hash | 13.275 | Beef, roast, hash | 13.275 |
| 27211550 | Stewed, seasoned, ground beef with potatoes, Mexican style | 2.521 | Stewed, seasoned, ground beef with potatoes, Mexican style | 2.521 |
| 27212000 | Beef and noodles, no sauce | 30.939 | Beef and noodles, no sauce | 30.939 |
| 27212050 | Beef and macaroni with cheese sauce | 6.292 | Beef and macaroni with cheese sauce | 6.292 |
| 27212100 | Beef and noodles with tomato-based sauce | 3.215 | Beef and noodles with tomato-based sauce | 3.215 |
| 27212120 | Chili con carne with beans and macaroni | 10.307 | Chili with macaroni | 10.307 |
| 27212150 | Beef goulash with noodles | 25.740 |  |  |
| 27212200 | Beef and noodles with gravy | 24.768 | Beef and noodles with gravy | 24.768 |
| 27212300 | Beef and noodles with cream or white sauce | 22.326 | Beef and noodles with cream or white sauce | 22.326 |
| 27212350 | Beef stroganoff with noodles | 13.927 | Beef stroganoff with noodles | 13.927 |
| 27212400 | Beef and noodles with mushroom sauce | 22.590 | Beef and noodles with mushroom sauce | 22.590 |
| 27212500 | Beef and noodles with soy-based sauce | 18.838 | Beef and noodles with soy-based sauce | 18.838 |
| 27213010 | Biryani with meat | 14.651 | Biryani with meat | 14.651 |
| 27213120 | Porcupine balls with tomato-based sauce | 8.002 | Porcupine balls with tomato-based sauce | 8.002 |
| 27213420 | Porcupine balls with mushroom sauce | 8.017 | Porcupine balls with mushroom sauce | 8.017 |
| 27213500 | Beef and rice with soy-based sauce | 19.215 | Beef and rice with soy-based sauce | 19.215 |
| 27213600 | Beef and rice with cheese sauce | 0.961 | Beef and rice with cheese sauce | 0.961 |
| 27214100 | Meat loaf made with beef | 16.224 | Meat loaf made with beef | 16.224 |
| 27214110 | Meat loaf made with beef, with tomato-based sauce | 12.539 | Meat loaf made with beef, with tomato-based sauce | 12.539 |
| 27214300 | Beef wellington | 6.333 | Beef wellington | 6.333 |
| 27214600 | Creamed dried beef on toast | 6.529 |  |  |
| 27218110 | Stuffed pot roast, with potatoes, Puerto Rican style | 4.606 |  |  |
| 27218210 | Beef stew with potatoes, Puerto Rican style | 0.438 | Beef stew with potatoes, Puerto Rican style | 0.438 |
| 27218310 | Stewed corned beef, Puerto Rican style | 1.258 |  |  |
| 27220010 | Meat loaf made with ham | 5.330 | Meat loaf made with ham | 5.330 |
| 27220020 | Ham and noodles with cream or white sauce | 22.770 | Ham and noodles with cream or white sauce | 22.770 |
| 27220050 | Ham or pork with stuffing | 25.237 | Ham or pork with stuffing | 25.237 |
| 27220080 | Ham croquette | 5.209 | Ham croquette | 5.209 |
| 27220190 | Sausage and noodles with cream or white sauce | 21.739 | Sausage and noodles with cream or white sauce | 21.739 |
| 27220210 | Ham and noodles, no sauce | 28.777 | Ham and noodles, no sauce | 28.777 |
| 27221100 | Stewed pig's feet, Puerto Rican style | 1.061 | Stewed pig's feet, Puerto Rican style | 1.061 |
| 27221110 | Stuffed pork roast, Puerto Rican style | 8.031 |  |  |
| 27221150 | Pork stew, with potatoes, tomato-based sauce, Mexican style | 10.718 | Stew, pork | 10.718 |
| 27221170 |  |  | Stew, pork, with pasta | 11.842 |
| 27230010 | Lamb or mutton loaf | 8.450 | Lamb or mutton loaf | 8.450 |
| 27233000 | Lamb or mutton and noodles with gravy | 25.157 | Lamb or mutton and noodles with gravy | 25.157 |
| 27235000 | Meat loaf made with venison/deer | 12.239 | Meat loaf made with venison/deer | 12.239 |
| 27235750 | Veal and noodles with cream or white sauce | 21.521 |  |  |
| 27236000 | Venison or deer and noodles with cream or white sauce | 21.521 | Venison or deer and noodles with cream or white sauce | 21.521 |
| 27241000 | Chicken or turkey hash | 2.240 | Chicken or turkey hash | 2.240 |
| 27241010 | Chicken or turkey and potatoes with gravy | 2.744 | Chicken or turkey and potatoes with gravy | 2.744 |
| 27242000 | Chicken or turkey and noodles, no sauce | 28.037 | Chicken or turkey and noodles, no sauce | 28.037 |
| 27242200 | Chicken or turkey and noodles with gravy | 24.515 | Chicken or turkey and noodles with gravy | 24.515 |
| 27242250 | Chicken or turkey and noodles with mushroom sauce | 22.440 | Chicken or turkey and noodles with mushroom sauce | 22.440 |
| 27242300 | Chicken or turkey and noodles with cream or white sauce | 22.642 | Chicken or turkey and noodles with cream or white sauce | 22.642 |
| 27242310 | Chicken or turkey and noodles with cheese sauce | 21.739 | Chicken or turkey and noodles with cheese sauce | 21.739 |
| 27242350 | Chicken or turkey tetrazzini | 8.114 | Chicken or turkey tetrazzini | 8.114 |
| 27242400 | Chicken or turkey and noodles with tomato-based sauce | 24.410 | Chicken or turkey and noodles with tomato-based sauce | 24.410 |
| 27242500 | Chicken or turkey and noodles with soy-based sauce | 18.838 | Chicken or turkey and noodles with soy-based sauce | 18.838 |
| 27243100 | Biryani with chicken | 16.286 | Biryani with chicken | 16.286 |
| 27243300 | Chicken or turkey and rice with cream sauce | 5.968 | Chicken or turkey and rice with cream sauce | 5.968 |
| 27243600 | Chicken or turkey and rice with soy-based sauce | 19.215 | Chicken or turkey and rice with soy-based sauce | 19.215 |
| 27243700 | Chicken in cheese sauce with Spanish rice | 1.392 |  |  |
| 27246100 | Chicken or turkey with dumplings | 4.649 | Chicken or turkey with dumplings | 4.649 |
| 27246200 | Chicken or turkey with stuffing | 1.997 | Chicken or turkey with stuffing | 1.997 |
| 27246300 | Chicken or turkey cake, patty, or croquette | 5.178 | Chicken or turkey cake, patty, or croquette | 5.178 |
| 27246400 | Chicken or turkey souffle | 1.881 | Chicken or turkey souffle | 1.881 |
| 27246500 | Meat loaf made with chicken or turkey | 15.967 | Meat loaf made with chicken or turkey | 15.967 |
| 27246505 | Meat loaf made with chicken or turkey, with tomato-based sauce | 11.909 | Meat loaf made with chicken or turkey, with tomato-based sauce | 11.909 |
| 27250020 | Clams, stuffed | 19.226 |  |  |
| 27250030 | Codfish ball or cake | 1.585 |  |  |
| 27250040 | Crab cake | 5.205 | Crab, cake | 5.205 |
| 27250050 | Fish cake or patty, NS as to fish | 1.546 | Fish, cake or patty | 1.546 |
| 27250060 | Gefilte fish | 3.185 | Gefilte fish | 3.185 |
| 27250070 | Salmon cake or patty | 5.205 | Fish, salmon cake or patty | 5.205 |
| 27250080 | Salmon loaf | 7.910 |  |  |
| 27250110 | Scallops and noodles with cheese sauce | 22.822 |  |  |
| 27250120 | Shrimp and noodles, no sauce | 29.254 | Shrimp and noodles, no sauce | 29.254 |
| 27250122 | Shrimp and noodles with gravy | 23.278 | Shrimp and noodles with gravy | 23.278 |
| 27250124 | Shrimp and noodles with mushroom sauce | 22.378 | Shrimp and noodles with mushroom sauce | 22.378 |
| 27250126 | Shrimp and noodles with cream or white sauce | 23.798 | Shrimp and noodles with cream or white sauce | 23.798 |
| 27250128 | Shrimp and noodles with soy-based sauce | 18.797 | Shrimp and noodles with soy-based sauce | 18.797 |
| 27250130 | Shrimp and noodles with cheese sauce | 21.220 | Shrimp and noodles with cheese sauce | 21.220 |
| 27250132 | Shrimp and noodles with tomato sauce | 24.365 | Shrimp and noodles with tomato sauce | 24.365 |
| 27250150 | Tuna loaf | 8.088 |  |  |
| 27250160 | Tuna cake or patty | 5.193 | Tuna cake or patty | 5.193 |
| 27250210 | Clam cake or patty | 5.178 | Clam cake or patty | 5.178 |
| 27250220 | Oyster fritter | 3.277 |  |  |
| 27250250 | Flounder with crab stuffing | 6.993 | Flounder with crab stuffing | 6.993 |
| 27250260 | Lobster with bread stuffing, baked | 12.507 | Lobster with bread stuffing, baked | 12.507 |
| 27250270 | Clams Casino | 21.698 | Clams Casino | 21.698 |
| 27250300 | Mackerel cake or patty | 1.455 |  |  |
| 27250310 | Haddock cake or patty | 1.546 |  |  |
| 27250400 | Shrimp cake or patty | 5.208 | Shrimp, cake or patty | 5.208 |
| 27250410 | Shrimp with crab stuffing | 4.817 | Shrimp with crab stuffing | 4.817 |
| 27250450 | Shrimp toast, fried | 7.857 | Shrimp toast | 7.857 |
| 27250550 | Seafood souffle | 1.876 | Seafood souffle | 1.876 |
| 27250610 | Tuna noodle casserole with cream or white sauce | 16.063 | Tuna noodle casserole with cream or white sauce | 16.063 |
| 27250630 | Tuna noodle casserole with mushroom sauce | 15.375 | Tuna noodle casserole with mushroom sauce | 15.375 |
| 27250900 | Fish and noodles with mushroom sauce | 22.328 | Fish and noodles with mushroom sauce | 22.328 |
| 27250950 | Shellfish and noodles with tomato-based sauce | 24.363 | Shellfish and noodles with tomato-based sauce | 24.363 |
| 27251010 | Stewed salmon, Puerto Rican style | 11.110 | Stewed salmon, Puerto Rican style | 11.110 |
| 27260010 | Meat loaf, NS as to type of meat | 16.235 | Meat loaf, NS as to type of meat | 16.235 |
| 27260050 | Meatballs, with breading, NS as to type of meat, with gravy | 10.116 | Meatballs, with breading, NS as to type of meat, with gravy | 10.116 |
| 27260080 | Meat loaf made with beef and pork | 16.187 | Meat loaf made with beef and pork | 16.187 |
| 27260090 | Meat loaf made with beef, veal and pork | 16.187 | Meat loaf made with beef, veal and pork | 16.187 |
| 27260100 | Meat loaf made with beef and pork, with tomato-based sauce | 12.517 | Meat loaf made with beef and pork, with tomato-based sauce | 12.517 |
| 27260510 | Liver dumpling | 9.707 |  |  |
| 27261000 | Breaded brains, Puerto Rican style | 7.377 |  |  |
| 27261500 | Stewed, seasoned, ground beef and pork with potatoes, Mexican style | 3.913 | Stewed, seasoned, ground beef and pork with potatoes, Mexican style | 3.913 |
| 27311110 | Beef, potatoes, and vegetables including carrots, broccoli, and/or dark-green leafy; no sauce | 0.881 | Beef, potatoes, and vegetables including carrots, broccoli, and/or dark-green leafy; no sauce | 0.881 |
| 27311210 | Corned beef, potatoes, and vegetables including carrots, broccoli, and/or dark-green leafy; no sauce | 0.894 | Corned beef, potatoes, and vegetables including carrots, broccoli, and/or dark-green leafy; no sauce | 0.894 |
| 27311310 | Beef stew with potatoes and vegetables including carrots, broccoli, and/or dark-green leafy; tomato-based sauce | 3.644 | Stew, beef, canned | 3.644 |
| 27311320 | Beef stew with potatoes and vegetables excluding carrots, broccoli, and dark-green leafy; tomato-based sauce | 7.940 |  |  |
| 27311410 | Beef stew with potatoes and vegetables including carrots, broccoli, and/or dark-green leafy; gravy | 7.401 | Stew, beef | 7.401 |
| 27311420 | Beef stew with potatoes and vegetables excluding carrots, broccoli, and dark-green leafy; gravy | 8.172 |  |  |
| 27311430 |  |  | Stew, beef, with pasta | 11.842 |
| 27311510 | Shepherd's pie with beef | 2.853 | Shepherd's pie | 2.853 |
| 27311600 | Beef, potatoes, and vegetables including carrots, broccoli, and/or dark-green leafy; gravy | 0.642 | Beef, potatoes, and vegetables including carrots, broccoli, and/or dark-green leafy; gravy | 0.642 |
| 27311610 | Beef, potatoes, and vegetables including carrots, broccoli, and/or dark-green leafy; cream sauce, white sauce, or mushroom sauce | 0.596 | Beef, potatoes, and vegetables including carrots, broccoli, and/or dark-green leafy; cream sauce, white sauce, or mushroom sauce | 0.596 |
| 27311625 | Beef, potatoes, and vegetables including carrots, broccoli, and/or dark-green leafy; tomato-based sauce | 0.639 | Beef, potatoes, and vegetables including carrots, broccoli, and/or dark-green leafy; tomato-based sauce | 0.639 |
| 27311635 | Beef, potatoes, and vegetables including carrots, broccoli, and/or dark-green leafy; cheese sauce | 0.676 | Beef, potatoes, and vegetables including carrots, broccoli, and/or dark-green leafy; cheese sauce | 0.676 |
| 27311645 | Beef, potatoes, and vegetables including carrots, broccoli, and/or dark-green leafy; soy-based sauce | 17.187 | Beef, potatoes, and vegetables including carrots, broccoli, and/or dark-green leafy; soy-based sauce | 17.187 |
| 27311650 | Beef, potatoes, and vegetables excluding carrots, broccoli, and dark-green leafy; soy-based sauce | 16.453 | Beef, potatoes, and vegetables excluding carrots, broccoli, and dark-green leafy; soy-based sauce | 16.453 |
| 27313010 | Beef, noodles, and vegetables including carrots, broccoli, and/or dark-green leafy; no sauce | 28.323 | Beef, noodles, and vegetables including carrots, broccoli, and/or dark-green leafy; no sauce | 28.323 |
| 27313020 | Beef, noodles, and vegetables excluding carrots, broccoli, and dark-green leafy; no sauce | 27.517 | Beef, noodles, and vegetables excluding carrots, broccoli, and dark-green leafy; no sauce | 27.517 |
| 27313110 | Beef chow mein or chop suey with noodles | 20.556 | Beef chow mein or chop suey with noodles | 20.556 |
| 27313150 | Beef, noodles, and vegetables including carrots, broccoli, and/or dark-green leafy; soy-based sauce | 15.062 | Beef, noodles, and vegetables including carrots, broccoli, and/or dark-green leafy; soy-based sauce | 15.062 |
| 27313160 | Beef, noodles, and vegetables excluding carrots, broccoli, and dark-green leafy; soy-based sauce | 14.544 | Beef, noodles, and vegetables excluding carrots, broccoli, and dark-green leafy; soy-based sauce | 14.544 |
| 27313210 | Beef, noodles, and vegetables including carrots, broccoli, and/or dark-green leafy; tomato-based sauce | 20.886 | Beef, noodles, and vegetables including carrots, broccoli, and/or dark-green leafy; tomato-based sauce | 20.886 |
| 27313220 | Beef, noodles, and vegetables excluding carrots, broccoli, and dark-green leafy; tomato-based sauce | 20.228 | Beef, noodles, and vegetables excluding carrots, broccoli, and dark-green leafy; tomato-based sauce | 20.228 |
| 27313310 | Beef, noodles, and vegetables including carrots, broccoli, and/or dark-green leafy; mushroom sauce | 19.480 | Beef, noodles, and vegetables including carrots, broccoli, and/or dark-green leafy; mushroom sauce | 19.480 |
| 27313320 | Beef, noodles, and vegetables excluding carrots, broccoli, and dark-green leafy; mushroom sauce | 18.868 | Beef, noodles, and vegetables excluding carrots, broccoli, and dark-green leafy; mushroom sauce | 18.868 |
| 27313410 | Beef, noodles, and vegetables including carrots, broccoli, and/or dark-green leafy; gravy | 21.011 | Beef, noodles, and vegetables including carrots, broccoli, and/or dark-green leafy; gravy | 21.011 |
| 27313420 | Beef, noodles, and vegetables excluding carrots, broccoli, and dark-green leafy; gravy | 20.346 | Beef, noodles, and vegetables excluding carrots, broccoli, and dark-green leafy; gravy | 20.346 |
| 27315010 | Beef, rice, and vegetables including carrots, broccoli, and/or dark-green leafy; no sauce | 0.885 | Beef, rice, and vegetables including carrots, broccoli, and/or dark-green leafy; no sauce | 0.885 |
| 27315210 | Beef, rice, and vegetables including carrots, broccoli, and/or dark-green leafy; tomato-based sauce | 0.650 | Beef, rice, and vegetables including carrots, broccoli, and/or dark-green leafy; tomato-based sauce | 0.650 |
| 27315250 | Stuffed cabbage rolls with beef and rice | 3.805 | Stuffed cabbage rolls with beef and rice | 3.805 |
| 27315270 | Stuffed grape leaves with beef and rice | 8.168 | Stuffed grape leaves with beef and rice | 8.168 |
| 27315310 | Beef, rice, and vegetables including carrots, broccoli, and/or dark-green leafy; mushroom sauce | 0.607 | Beef, rice, and vegetables including carrots, broccoli, and/or dark-green leafy; mushroom sauce | 0.607 |
| 27315330 | Beef, rice, and vegetables including carrots, broccoli, and/or dark-green leafy; cheese sauce | 0.676 | Beef, rice, and vegetables including carrots, broccoli, and/or dark-green leafy; cheese sauce | 0.676 |
| 27315410 | Beef, rice, and vegetables including carrots, broccoli, and/or dark-green leafy; gravy | 0.655 | Beef, rice, and vegetables including carrots, broccoli, and/or dark-green leafy; gravy | 0.655 |
| 27315510 | Beef, rice, and vegetables including carrots, broccoli, and/or dark-green leafy; soy-based sauce | 15.284 | Beef, rice, and vegetables including carrots, broccoli, and/or dark-green leafy; soy-based sauce | 15.284 |
| 27315520 | Beef, rice, and vegetables excluding carrots, broccoli, and dark-green leafy; soy-based sauce | 14.761 | Beef, rice, and vegetables excluding carrots, broccoli, and dark-green leafy; soy-based sauce | 14.761 |
| 27317010 | Beef pot pie | 9.416 | Pot pie, beef | 9.416 |
| 27317100 | Beef, dumplings, and vegetables including carrots, broccoli, and/or dark-green leafy; gravy | 0.660 | Beef, dumplings, and vegetables including carrots, broccoli, and/or dark-green leafy; gravy | 0.660 |
| 27319010 | Stuffed green pepper, Puerto Rican style | 6.585 | Stuffed green pepper, Puerto Rican style | 6.585 |
| 27320020 | Ham pot pie | 9.133 |  |  |
| 27320025 | Ham or pork, noodles and vegetables excluding carrots, broccoli, and dark-green leafy; no sauce | 27.542 | Ham or pork, noodles and vegetables excluding carrots, broccoli, and dark-green leafy; no sauce | 27.542 |
| 27320027 | Ham or pork, noodles, and vegetables including carrots, broccoli, and/or dark-green leafy; no sauce | 28.369 | Ham or pork, noodles, and vegetables including carrots, broccoli, and/or dark-green leafy; no sauce | 28.369 |
| 27320030 | Ham or pork, noodles and vegetables excluding carrots, broccoli, and dark-green leafy; cheese sauce | 20.790 | Ham or pork, noodles and vegetables excluding carrots, broccoli, and dark-green leafy; cheese sauce | 20.790 |
| 27320040 | Pork, potatoes, and vegetables including carrots, broccoli, and/or dark-green leafy; no sauce | 0.880 | Pork, potatoes, and vegetables including carrots, broccoli, and/or dark-green leafy; no sauce | 0.880 |
| 27320070 | Ham or pork, noodles, and vegetables including carrots, broccoli, and/or dark-green leafy; tomato-based sauce | 20.911 | Ham or pork, noodles, and vegetables including carrots, broccoli, and/or dark-green leafy; tomato-based sauce | 20.911 |
| 27320080 | Sausage, noodles, and vegetables excluding carrots, broccoli, and dark-green leafy; tomato-based sauce | 20.619 | Sausage, noodles, and vegetables excluding carrots, broccoli, and dark-green leafy; tomato-based sauce | 20.619 |
| 27320090 | Sausage, noodles, and vegetables including carrots, broccoli, and/or dark-green leafy; tomato-based sauce | 21.289 | Sausage, noodles, and vegetables including carrots, broccoli, and/or dark-green leafy; tomato-based sauce | 21.289 |
| 27320100 | Pork, potatoes, and vegetables including carrots, broccoli, and/or dark-green leafy; tomato-based sauce | 0.639 | Pork, potatoes, and vegetables including carrots, broccoli, and/or dark-green leafy; tomato-based sauce | 0.639 |
| 27320120 | Sausage, potatoes, and vegetables including carrots, broccoli, and/or dark-green leafy; gravy | 0.656 | Sausage, potatoes, and vegetables including carrots, broccoli, and/or dark-green leafy; gravy | 0.656 |
| 27320140 | Pork, potatoes, and vegetables including carrots, broccoli, and/or dark-green leafy; gravy | 0.644 | Pork, potatoes, and vegetables including carrots, broccoli, and/or dark-green leafy; gravy | 0.644 |
| 27320310 | Pork chow mein or chop suey with noodles | 20.556 | Pork chow mein or chop suey with noodles | 20.556 |
| 27320320 | Pork, rice, and vegetables including carrots, broccoli, and/or dark-green leafy; soy-based sauce | 15.284 | Pork, rice, and vegetables including carrots, broccoli, and/or dark-green leafy; soy-based sauce | 15.284 |
| 27320330 | Pork, rice, and vegetables excluding carrots, broccoli, and dark-green leafy; soy-based sauce | 14.766 | Pork, rice, and vegetables excluding carrots, broccoli, and dark-green leafy; soy-based sauce | 14.766 |
| 27320340 | Pork, rice, and vegetables including carrots, broccoli, and/or dark-green leafy; tomato-based sauce | 0.653 | Pork, rice, and vegetables including carrots, broccoli, and/or dark-green leafy; tomato-based sauce | 0.653 |
| 27320450 | Ham, potatoes, and vegetables including carrots, broccoli, and/or dark-green leafy; no sauce | 0.877 | Ham, potatoes, and vegetables including carrots, broccoli, and/or dark-green leafy; no sauce | 0.877 |
| 27320500 | Sweet and sour pork with rice | 8.328 | Sweet and sour pork with rice | 8.328 |
| 27330010 | Shepherd's pie with lamb | 3.449 |  |  |
| 27330060 | Lamb or mutton, rice, and vegetables including carrots, broccoli, and/or dark-green leafy; tomato-based sauce | 0.661 | Lamb or mutton, rice, and vegetables including carrots, broccoli, and/or dark-green leafy; tomato-based sauce | 0.661 |
| 27330080 | Lamb or mutton, rice, and vegetables including carrots, broccoli, and/or dark-green leafy; gravy | 0.663 | Lamb or mutton, rice, and vegetables including carrots, broccoli, and/or dark-green leafy; gravy | 0.663 |
| 27330170 | Stuffed grape leaves with lamb and rice | 8.908 | Stuffed grape leaves with lamb and rice | 8.908 |
| 27331150 | Veal fricassee, Puerto Rican style | 5.405 |  |  |
| 27332100 | Veal stew with potatoes and vegetables including carrots, broccoli, and/or dark-green leafy; tomato-based sauce | 8.474 |  |  |
| 27332110 | Veal stew with potatoes and vegetables excluding carrots, broccoli, and/or dark-green leafy; tomato-based sauce | 7.933 |  |  |
| 27335100 | Rabbit stew with potatoes and vegetables | 10.878 |  |  |
| 27335500 | Stewed rabbit, Puerto Rican style, | 4.739 | Stewed rabbit, Puerto Rican style, | 4.739 |
| 27336100 | Venison or deer stew with potatoes and vegetables including carrots, broccoli, and/or dark-green leafy; tomato-based sauce | 7.172 |  |  |
| 27336150 | Venison or deer stew with potatoes and vegetables excluding carrots, broccoli, and dark-green leafy; tomato-based sauce | 9.731 |  |  |
| 27336200 | Venison or deer, potatoes, and vegetables including carrots, broccoli, and/or dark-green leafy; gravy | 0.641 | Venison or deer, potatoes, and vegetables including carrots, broccoli, and/or dark-green leafy; gravy | 0.641 |
| 27336300 | Venison or deer, noodles, and vegetables including carrots, broccoli, and/or dark-green leafy; tomato-based sauce | 20.736 | Venison or deer, noodles, and vegetables including carrots, broccoli, and/or dark-green leafy; tomato-based sauce | 20.736 |
| 27336310 | Venison or deer, noodles, and vegetables excluding carrots, broccoli, and dark-green leafy; tomato-based sauce | 20.084 | Venison or deer, noodles, and vegetables excluding carrots, broccoli, and dark-green leafy; tomato-based sauce | 20.084 |
| 27341010 | Chicken or turkey, potatoes, and vegetables including carrots, broccoli, and/or dark-green leafy; no sauce | 0.871 | Chicken or turkey, potatoes, and vegetables including carrots, broccoli, and/or dark-green leafy; no sauce | 0.871 |
| 27341025 | Chicken or turkey, potatoes, and vegetables including carrots, broccoli, and/or dark-green leafy; gravy | 0.642 | Chicken or turkey, potatoes, and vegetables including carrots, broccoli, and/or dark-green leafy; gravy | 0.642 |
| 27341035 | Chicken or turkey, potatoes, and vegetables including carrots, broccoli, and/or dark-green leafy; cream sauce, white sauce, or mushroom sauce | 0.594 | Chicken or turkey, potatoes, and vegetables including carrots, broccoli, and/or dark-green leafy; cream sauce, white sauce, or mushroom sauce | 0.594 |
| 27341045 | Chicken or turkey, potatoes, and vegetables including carrots, broccoli, and/or dark-green leafy; cheese sauce | 0.670 | Chicken or turkey, potatoes, and vegetables including carrots, broccoli, and/or dark-green leafy; cheese sauce | 0.670 |
| 27341055 | Chicken or turkey, potatoes, and vegetables including carrots, broccoli, and/or dark-green leafy; tomato-based sauce | 0.637 | Chicken or turkey, potatoes, and vegetables including carrots, broccoli, and/or dark-green leafy; tomato-based sauce | 0.637 |
| 27341310 | Chicken or turkey stew with potatoes and vegetables including carrots, broccoli, and/or dark-green leafy; gravy | 11.646 |  |  |
| 27341320 | Chicken or turkey stew with potatoes and vegetables excluding carrots, broccoli, and dark-green leafy; gravy | 11.276 |  |  |
| 27341330 |  |  | Stew, chicken, with pasta | 11.842 |
| 27341510 | Chicken or turkey stew with potatoes and vegetables including carrots, broccoli, and/or dark-green leafy; tomato-based sauce | 11.601 |  |  |
| 27341520 | Chicken or turkey stew with potatoes and vegetables excluding carrots, broccoli, and dark-green leafy; tomato- based sauce | 11.234 | Stew, chicken | 11.234 |
| 27343010 | Chicken or turkey, noodles, and vegetables including carrots, broccoli, and/or dark-green leafy; no sauce | 28.127 | Chicken or turkey, noodles, and vegetables including carrots, broccoli, and/or dark-green leafy; no sauce | 28.127 |
| 27343020 | Chicken or turkey, noodles, and vegetables excluding carrots, broccoli, and dark-green leafy; no sauce | 27.251 | Chicken or turkey, noodles, and vegetables excluding carrots, broccoli, and dark-green leafy; no sauce | 27.251 |
| 27343410 | Chicken or turkey, noodles, and vegetables including carrots, broccoli, and/or dark-green leafy; gravy | 20.820 | Chicken or turkey, noodles, and vegetables including carrots, broccoli, and/or dark-green leafy; gravy | 20.820 |
| 27343420 | Chicken or turkey, noodles, and vegetables excluding carrots, broccoli, and dark-green leafy; gravy | 20.243 | Chicken or turkey, noodles, and vegetables excluding carrots, broccoli, and dark-green leafy; gravy | 20.243 |
| 27343470 | Chicken or turkey, noodles, and vegetables including carrots, broccoli, and/or dark-green leafy; cream sauce, white sauce, or mushroom sauce | 19.305 | Chicken or turkey, noodles, and vegetables including carrots, broccoli, and/or dark-green leafy; cream sauce, white sauce, or mushroom sauce | 19.305 |
| 27343480 | Chicken or turkey, noodles, and vegetables excluding carrots, broccoli, and/or dark-green leafy; cream sauce, white sauce, or mushroom sauce | 18.692 | Chicken or turkey, noodles, and vegetables excluding carrots, broccoli, and/or dark-green leafy; cream sauce, white sauce, or mushroom sauce | 18.692 |
| 27343510 | Chicken or turkey, noodles, and vegetables including carrots, broccoli, and/or dark-green leafy; tomato-based sauce | 20.650 | Chicken or turkey, noodles, and vegetables including carrots, broccoli, and/or dark-green leafy; tomato-based sauce | 20.650 |
| 27343520 | Chicken or turkey, noodles, and vegetables excluding carrots, broccoli, and dark-green leafy; tomato-based sauce | 20.000 | Chicken or turkey, noodles, and vegetables excluding carrots, broccoli, and dark-green leafy; tomato-based sauce | 20.000 |
| 27343910 | Chicken or turkey chow mein or chop suey with noodles | 20.512 | Chicken or turkey chow mein or chop suey with noodles | 20.512 |
| 27343950 | Chicken or turkey, noodles, and vegetables including carrots, broccoli, and/or dark-green leafy; cheese sauce | 21.466 | Chicken or turkey, noodles, and vegetables including carrots, broccoli, and/or dark-green leafy; cheese sauce | 21.466 |
| 27343960 | Chicken or turkey, noodles, and vegetables excluding carrots, broccoli, and dark-green leafy; cheese sauce | 20.790 | Chicken or turkey, noodles, and vegetables excluding carrots, broccoli, and dark-green leafy; cheese sauce | 20.790 |
| 27345010 | Chicken or turkey, rice, and vegetables including carrots, broccoli, and/or dark-green leafy; no sauce | 0.876 | Chicken or turkey, rice, and vegetables including carrots, broccoli, and/or dark-green leafy; no sauce | 0.876 |
| 27345210 | Chicken or turkey, rice, and vegetables including carrots, broccoli, and/or dark-green leafy; gravy | 0.650 | Chicken or turkey, rice, and vegetables including carrots, broccoli, and/or dark-green leafy; gravy | 0.650 |
| 27345310 | Chicken or turkey, rice, and vegetables including carrots, broccoli, and/or dark-green leafy; soy-based sauce | 15.284 | Chicken or turkey, rice, and vegetables including carrots, broccoli, and/or dark-green leafy; soy-based sauce | 15.284 |
| 27345320 | Chicken or turkey, rice, and vegetables excluding carrots, broccoli, and dark-green leafy; soy-based sauce | 14.766 | Chicken or turkey, rice, and vegetables excluding carrots, broccoli, and dark-green leafy; soy-based sauce | 14.766 |
| 27345410 | Chicken or turkey, rice, and vegetables including carrots, broccoli, and/or dark-green leafy; cream sauce, white sauce, or mushroom sauce | 0.603 | Chicken or turkey, rice, and vegetables including carrots, broccoli, and/or dark-green leafy; cream sauce, white sauce, or mushroom sauce | 0.603 |
| 27345440 | Chicken or turkey, rice, and vegetables including carrots, broccoli, and/or dark-green leafy; cheese sauce | 0.676 | Chicken or turkey, rice, and vegetables including carrots, broccoli, and/or dark-green leafy; cheese sauce | 0.676 |
| 27345510 | Chicken or turkey, rice, and vegetables including carrots, broccoli, and/or dark-green leafy; tomato-based sauce | 0.647 | Chicken or turkey, rice, and vegetables including carrots, broccoli, and/or dark-green leafy; tomato-based sauce | 0.647 |
| 27347100 | Chicken or turkey pot pie | 10.560 | Pot pie, chicken | 10.560 |
| 27347200 | Chicken or turkey, stuffing, and vegetables including carrots, broccoli, and/or dark-green leafy; no sauce | 29.153 | Chicken or turkey, stuffing, and vegetables including carrots, broccoli, and/or dark-green leafy; no sauce | 29.153 |
| 27347210 | Chicken or turkey,stuffing, and vegetables excluding carrots, broccoli, and dark green leafy; no sauce | 28.235 | Chicken or turkey,stuffing, and vegetables excluding carrots, broccoli, and dark green leafy; no sauce | 28.235 |
| 27347220 | Chicken or turkey, stuffing, and vegetables including carrots, broccoli, and/or dark-green leafy; gravy | 21.548 | Chicken or turkey, stuffing, and vegetables including carrots, broccoli, and/or dark-green leafy; gravy | 21.548 |
| 27347230 | Chicken or turkey, stuffing, and vegetables excluding carrots, broccoli, and dark-green leafy; gravy | 20.870 | Chicken or turkey, stuffing, and vegetables excluding carrots, broccoli, and dark-green leafy; gravy | 20.870 |
| 27347240 | Chicken or turkey, dumplings, and vegetables including carrots, broccoli, and/or dark green leafy; gravy | 0.655 | Chicken or turkey, dumplings, and vegetables including carrots, broccoli, and/or dark green leafy; gravy | 0.655 |
| 27348100 | Chicken fricassee, Puerto Rican style | 7.464 | Chicken fricassee, Puerto Rican style | 7.464 |
| 27350020 | Paella with seafood | 0.501 | Paella with seafood | 0.501 |
| 27350030 | Seafood stew with potatoes and vegetables excluding carrots, broccoli, and dark-green leafy; tomato-based sauce | 6.054 |  |  |
| 27350050 | Shrimp chow mein or chop suey with noodles | 20.071 | Shrimp chow mein or chop suey with noodles | 20.071 |
| 27350060 | Shrimp creole, with rice | 2.914 | Shrimp creole, with rice | 2.914 |
| 27350070 | Tuna pot pie | 7.564 |  |  |
| 27350080 | Tuna noodle casserole with vegetables, cream or white sauce | 16.812 | Tuna noodle casserole with vegetables, cream or white sauce | 16.812 |
| 27350090 | Fish, noodles, and vegetables including carrots, broccoli, and/or dark green leafy; cheese sauce | 21.466 | Fish, noodles, and vegetables including carrots, broccoli, and/or dark green leafy; cheese sauce | 21.466 |
| 27350100 | Fish, noodles, and vegetables excluding carrots, broccoli, and dark-green leafy; cheese sauce | 20.790 | Fish, noodles, and vegetables excluding carrots, broccoli, and dark-green leafy; cheese sauce | 20.790 |
| 27350110 | Bouillabaisse | 2.591 | Soup, bouillabaisse | 2.591 |
| 27350200 | Oyster pie | 9.150 |  |  |
| 27350310 | Seafood stew with potatoes and vegetables including carrots, broccoli, and/or dark-green leafy; tomato-based sauce | 6.160 |  |  |
| 27350410 | Tuna noodle casserole with vegetables and mushroom sauce | 16.199 | Tuna noodle casserole with vegetables and mushroom sauce | 16.199 |
| 27351010 | Codfish with starchy vegetables, Puerto Rican style | 5.237 | Codfish with starchy vegetables, Puerto Rican style | 5.237 |
| 27351030 | Stewed codfish, Puerto Rican style | 10.247 |  |  |
| 27351040 | Biscayne codfish, Puerto Rican style | 17.473 | Biscayne codfish, Puerto Rican style | 17.473 |
| 27360010 | Goulash, NFS | 24.490 |  |  |
| 27360050 | Meat pie, NFS | 10.560 |  |  |
| 27360080 | Chow mein or chop suey, NS as to type of meat, with noodles | 20.512 | Chow mein or chop suey, NS as to type of meat, with noodles | 20.512 |
| 27360090 | Paella, NFS | 0.491 | Paella, NFS | 0.491 |
| 27360100 | Brunswick stew | 3.538 |  |  |
| 27360120 | Chow mein or chop suey, various types of meat, with noodles | 20.542 | Chow mein or chop suey, various types of meat, with noodles | 20.542 |
| 27361010 | Stewed variety meats, mostly liver, Puerto Rican style | 13.991 |  |  |
| 27362000 | Stewed tripe, with potatoes, Puerto Rican style | 2.932 | Stewed tripe, with potatoes, Puerto Rican style | 2.932 |
| 27363000 | Gumbo with rice | 8.473 | Gumbo with rice | 8.473 |
| 27363100 | Jambalaya with meat and rice | 6.687 | Jambalaya with meat and rice | 6.687 |
| 27410210 | Beef and vegetables including carrots, broccoli, and/or dark-green leafy; no potatoes, no sauce | 1.702 | Beef and vegetables including carrots, broccoli, and/or dark-green leafy; no potatoes, no sauce | 1.702 |
| 27410250 | Beef shish kabob with vegetables, excluding potatoes | 29.019 | Beef shish kabob with vegetables, excluding potatoes | 29.019 |
| 27411100 | Beef with vegetables including carrots, broccoli, and/or dark-green leafy; no potatoes, tomato-based sauce | 1.145 | Beef with vegetables including carrots, broccoli, and/or dark-green leafy; no potatoes, tomato-based sauce | 1.145 |
| 27411120 | Swiss steak | 2.306 | Swiss steak | 2.306 |
| 27411150 | Beef rolls, stuffed with vegetables or meat mixture, tomato-based sauce | 2.736 | Beef rolls, stuffed with vegetables or meat mixture, tomato-based sauce | 2.736 |
| 27414100 | Beef with vegetables including carrots, broccoli, and/or dark-green leafy; no potatoes, mushroom sauce | 1.013 | Beef with vegetables including carrots, broccoli, and/or dark-green leafy; no potatoes, mushroom sauce | 1.013 |
| 27415100 | Beef and vegetables including carrots, broccoli, and/or dark-green leafy; no potatoes, soy-based sauce | 17.576 | Beef and vegetables including carrots, broccoli, and/or dark-green leafy; no potatoes, soy-based sauce | 17.576 |
| 27415110 | Beef and broccoli | 4.798 | Beef and broccoli | 4.798 |
| 27415120 | Beef, tofu, and vegetables including carrots, broccoli, and/or dark-green leafy; no potatoes, soy-based sauce | 16.968 | Beef, tofu, and vegetables including carrots, broccoli, and/or dark-green leafy; no potatoes, soy-based sauce | 16.968 |
| 27415150 | Beef chow mein or chop suey, no noodles | 16.725 | Beef chow mein or chop suey, no noodles | 16.725 |
| 27415170 | Kung Pao beef | 4.940 | Kung Pao beef | 4.940 |
| 27415200 | Beef and vegetables excluding carrots, broccoli, and dark-green leafy; no potatoes, soy-based sauce | 17.067 | Beef and vegetables excluding carrots, broccoli, and dark-green leafy; no potatoes, soy-based sauce | 17.067 |
| 27415220 | Beef, tofu, and vegetables excluding carrots, broccoli, and dark-green leafy; no potatoes, soy-based sauce | 16.448 | Beef, tofu, and vegetables excluding carrots, broccoli, and dark-green leafy; no potatoes, soy-based sauce | 16.448 |
| 27416150 | Pepper steak | 17.056 | Pepper steak | 17.056 |
| 27416200 | Beef, ground, with egg and onion | 7.575 | Beef, ground, with egg and onion | 7.575 |
| 27416300 | Beef taco filling: beef, cheese, tomato, taco sauce | 0.051 | Beef taco filling: beef, cheese, tomato, taco sauce | 0.051 |
| 27416400 | Stir fried beef and vegetables in soy sauce | 11.920 | Stir fried beef and vegetables in soy sauce | 11.920 |
| 27416450 | Beef and vegetables including carrots, broccoli, and/or dark-green leafy; no potatoes, gravy | 1.152 | Beef and vegetables including carrots, broccoli, and/or dark-green leafy; no potatoes, gravy | 1.152 |
| 27418110 | Seasoned shredded soup meat | 16.481 |  |  |
| 27418210 | Beef stew with vegetables excluding potatoes, Puerto Rican style | 7.662 |  |  |
| 27418310 | Corned beef with tomato sauce and onion, Puerto Rican style | 13.683 |  |  |
| 27418410 | Beef steak with onions, Puerto Rican style | 26.153 | Beef steak with onions, Puerto Rican style | 26.153 |
| 27420010 | Cabbage with ham hocks | 5.467 | Cabbage with ham hocks | 5.467 |
| 27420060 | Pork and vegetables including carrots, broccoli, and/or dark-green leafy; no potatoes, no sauce | 1.781 | Pork and vegetables including carrots, broccoli, and/or dark-green leafy; no potatoes, no sauce | 1.781 |
| 27420080 | Greens with ham or pork | 1.799 | Greens with ham or pork | 1.799 |
| 27420100 | Pork, tofu, and vegetables including carrots, broccoli, and/or dark-green leafy; no potatoes, soy-base sauce | 16.968 | Pork, tofu, and vegetables including carrots, broccoli, and/or dark-green leafy; no potatoes, soy-base sauce | 16.968 |
| 27420110 | Pork and vegetables, Hawaiian style | 16.847 | Pork and vegetables, Hawaiian style | 16.847 |
| 27420120 | Pork and watercress with soy-based sauce | 17.090 | Pork and watercress with soy-based sauce | 17.090 |
| 27420150 | Kung Pao pork | 4.940 | Kung Pao pork | 4.940 |
| 27420160 | Moo Shu pork, without Chinese pancake | 5.984 | Moo Shu pork, without Chinese pancake | 5.984 |
| 27420170 | Pork and onions with soy-based sauce | 39.736 | Pork and onions with soy-based sauce | 39.736 |
| 27420200 | Pork hash | 2.199 | Pork hash | 2.199 |
| 27420250 | Ham and vegetables including carrots broccoli, and/or dark- green leafy; no potatoes, no sauce | 1.468 | Ham and vegetables including carrots broccoli, and/or dark- green leafy; no potatoes, no sauce | 1.468 |
| 27420370 | Pork, tofu, and vegetables, excluding carrots, broccoli, and dark-green leafy; no potatoes, soy-based sauce | 16.454 | Pork, tofu, and vegetables, excluding carrots, broccoli, and dark-green leafy; no potatoes, soy-based sauce | 16.454 |
| 27420390 | Pork chow mein or chop suey, no noodles | 16.725 | Pork chow mein or chop suey, no noodles | 16.725 |
| 27420400 | Pork and vegetables including carrots, broccoli, and/or dark-green leafy; no potatoes, tomato-based sauce | 1.145 | Pork and vegetables including carrots, broccoli, and/or dark-green leafy; no potatoes, tomato-based sauce | 1.145 |
| 27420450 | Sausage and vegetables including carrots, broccoli, and/or dark-green leafy; no potatoes, tomato-based sauce | 1.175 | Sausage and vegetables including carrots, broccoli, and/or dark-green leafy; no potatoes, tomato-based sauce | 1.175 |
| 27420500 | Pork and vegetables including carrots, broccoli, and/or dark-green leafy; no potatoes, soy-based sauce | 17.576 | Pork and vegetables including carrots, broccoli, and/or dark-green leafy; no potatoes, soy-based sauce | 17.576 |
| 27420510 | Pork and vegetables excluding carrots, broccoli, and dark- green leafy; no potatoes, soy-based sauce | 17.067 | Pork and vegetables excluding carrots, broccoli, and dark- green leafy; no potatoes, soy-based sauce | 17.067 |
| 27420520 | Pork shish kabob with vegetables, excluding potatoes | 29.019 | Pork shish kabob with vegetables, excluding potatoes | 29.019 |
| 27422010 | Pork chop stewed with vegetables, Puerto Rican style | 6.219 |  |  |
| 27430500 | Veal goulash with vegetables excluding carrots, broccoli, and dark-green leafy; no potatoes, tomato-based sauce | 16.765 |  |  |
| 27430510 | Veal goulash with vegetables including carrots, broccoli, and/or dark-green leafy; no potatoes, tomato-based sauce | 6.103 |  |  |
| 27430580 | Veal with vegetables including carrots, broccoli, and/or dark-green leafy; no potatoes, cream or white sauce | 0.996 |  |  |
| 27430610 | Lamb shish kabob with vegetables, excluding potatoes | 29.019 | Lamb shish kabob with vegetables, excluding potatoes | 29.019 |
| 27440110 | Chicken or turkey and vegetables including carrots, broccoli, and/or dark-green leafy; no potatoes, no sauce | 1.632 | Chicken or turkey and vegetables including carrots, broccoli, and/or dark-green leafy; no potatoes, no sauce | 1.632 |
| 27440130 | Chicken or turkey shish kabob with vegetables, excluding potatoes | 29.019 | Chicken or turkey shish kabob with vegetables, excluding potatoes | 29.019 |
| 27441120 | Chicken or turkey creole, without rice | 5.061 | Chicken or turkey creole, without rice | 5.061 |
| 27442110 | Chicken or turkey and vegetables including carrots, broccoli, and/or dark-green leafy; no potatoes, gravy | 1.144 | Chicken or turkey and vegetables including carrots, broccoli, and/or dark-green leafy; no potatoes, gravy | 1.144 |
| 27443110 | Chicken or turkey a la king with vegetables including carrots, broccoli, and/or dark-green leafy; no potatoes, cream, white, or soup-based sauce | 1.898 | Chicken or turkey a la king with vegetables including carrots, broccoli, and/or dark-green leafy; no potatoes, cream, white, or soup-based sauce | 1.898 |
| 27443120 | Chicken or turkey a la king with vegetables excluding carrorts, broccoli, and dark-green leafy; no potatoes, cream, white, or soup-based sauce | 1.895 | Chicken or turkey a la king with vegetables excluding carrorts, broccoli, and dark-green leafy; no potatoes, cream, white, or soup-based sauce | 1.895 |
| 27443150 | Chicken or turkey divan | 3.335 | Chicken or turkey divan | 3.335 |
| 27445120 | Chicken or turkey and vegetables excluding carrots, broccoli, and dark-green leafy; no potatoes, soy-based sauce | 17.067 | Chicken or turkey and vegetables excluding carrots, broccoli, and dark-green leafy; no potatoes, soy-based sauce | 17.067 |
| 27445125 | Chicken or turkey and vegetables including carrots, broccoli, and/or dark-green leafy; no potatoes, tomato-based sauce | 1.129 | Chicken or turkey and vegetables including carrots, broccoli, and/or dark-green leafy; no potatoes, tomato-based sauce | 1.129 |
| 27445180 | Moo Goo Gai Pan | 1.911 | Moo Goo Gai Pan | 1.911 |
| 27445250 | Almond chicken | 4.961 | Almond chicken | 4.961 |
| 27446100 | Chicken or turkey chow mein or chop suey, no noodles | 16.684 | Chicken or turkey chow mein or chop suey, no noodles | 16.684 |
| 27446300 | Chicken or turkey garden salad, chicken and/or turkey, tomato and/or carrots, other vegetables, no dressing | 6.052 | Chicken or turkey garden salad, chicken and/or turkey, tomato and/or carrots, other vegetables, no dressing | 6.052 |
| 27446310 | Chicken or turkey garden salad, chicken and/or turkey, other vegetables excluding tomato and carrots, no dressing | 6.504 | Chicken or turkey garden salad, chicken and/or turkey, other vegetables excluding tomato and carrots, no dressing | 6.504 |
| 27446315 | Chicken or turkey garden salad with bacon and cheese, chicken and/or turkey, bacon, cheese, lettuce and/or greens, tomato and/or carrots, other vegetables, no dressing | 4.523 | Chicken or turkey garden salad with bacon and cheese, chicken and/or turkey, bacon, cheese, lettuce and/or greens, tomato and/or carrots, other vegetables, no dressing | 4.523 |
| 27446320 | Chicken or turkey, breaded, fried, garden salad with bacon and cheese, chicken and/or turkey, bacon, cheese, lettuce and/or greens, tomato and/or carrots, other vegetables, no dressing | 5.013 | Chicken or turkey, breaded, fried, garden salad with bacon and cheese, chicken and/or turkey, bacon, cheese, lettuce and/or greens, tomato and/or carrots, other vegetables, no dressing | 5.013 |
| 27446330 | Chicken or turkey garden salad with cheese, chicken and/or turkey, cheese, lettuce and/or greens, tomato and/or carrots, other vegetables, no dressing | 4.647 | Chicken or turkey garden salad with cheese, chicken and/or turkey, cheese, lettuce and/or greens, tomato and/or carrots, other vegetables, no dressing | 4.647 |
| 27446350 | Asian chicken or turkey garden salad, chicken and/or turkey, lettuce, fruit, nuts, no dressing | 3.174 | Asian chicken or turkey garden salad, chicken and/or turkey, lettuce, fruit, nuts, no dressing | 3.174 |
| 27446355 | Asian chicken or turkey garden salad with crispy noodles, chicken and/or turkey, lettuce, fruit, nuts, crispy noodles, no dressing | 1.945 | Asian chicken or turkey garden salad with crispy noodles, chicken and/or turkey, lettuce, fruit, nuts, crispy noodles, no dressing | 1.945 |
| 27446400 | Chicken or turkey and vegetables including carrots, broccoli, and/or dark-green leafy; no potatoes, cheese sauce | 0.973 | Chicken or turkey and vegetables including carrots, broccoli, and/or dark-green leafy; no potatoes, cheese sauce | 0.973 |
| 27448020 | Chicken or turkey fricassee, with sauce, no potatoes, potatoes reported separately, Puerto Rican style | 6.888 | Chicken or turkey fricassee, with sauce, no potatoes, potatoes reported separately, Puerto Rican style | 6.888 |
| 27450040 | Shrimp chow mein or chop suey, no noodles | 16.276 | Shrimp chow mein or chop suey, no noodles | 16.276 |
| 27450110 | Shrimp garden salad, shrimp, lettuce, eggs, tomato and/or carrots, other vegetables, no dressing | 5.257 | Shrimp garden salad, shrimp, lettuce, eggs, tomato and/or carrots, other vegetables, no dressing | 5.257 |
| 27450120 | Shrimp garden salad, shrimp, lettuce, eggs, vegetables excluding tomato and carrots, no dressing | 5.594 | Shrimp garden salad, shrimp, lettuce, eggs, vegetables excluding tomato and carrots, no dressing | 5.594 |
| 27450150 | Fish, tofu, and vegetables, tempura | 6.475 | Fish, tofu, and vegetables, tempura | 6.475 |
| 27450180 | Seafood garden salad with seafood, lettuce, vegetables excluding tomato and carrots, no dressing | 6.565 | Seafood garden salad with seafood, lettuce, vegetables excluding tomato and carrots, no dressing | 6.565 |
| 27450190 | Seafood garden salad with seafood, lettuce, tomato and/or carrots, other vegetables, no dressing | 6.105 | Seafood garden salad with seafood, lettuce, tomato and/or carrots, other vegetables, no dressing | 6.105 |
| 27450200 | Seafood garden salad with seafood, lettuce, eggs, vegetables excluding tomato and carrots, no dressing | 5.550 | Seafood garden salad with seafood, lettuce, eggs, vegetables excluding tomato and carrots, no dressing | 5.550 |
| 27450210 | Seafood garden salad with seafood, lettuce, eggs, tomato and/or carrots, other vegetables, no dressing | 5.218 | Seafood garden salad with seafood, lettuce, eggs, tomato and/or carrots, other vegetables, no dressing | 5.218 |
| 27450250 | Oysters Rockefeller | 8.581 | Oysters Rockefeller | 8.581 |
| 27450310 | Lomi salmon | 12.776 | Lomi salmon | 12.776 |
| 27450400 | Shrimp and vegetables including carrots, broccoli, and/or dark-green leafy; no potatoes, no sauce | 1.831 | Shrimp and vegetables including carrots, broccoli, and/or dark-green leafy; no potatoes, no sauce | 1.831 |
| 27450410 | Shrimp and vegetables including carrots, broccoli, and/or dark-green leafy; no potatoes, soy-based sauce | 17.543 | Shrimp and vegetables including carrots, broccoli, and/or dark-green leafy; no potatoes, soy-based sauce | 17.543 |
| 27450420 | Shrimp and vegetables excluding carrots, broccoli, and dark-green leafy; no potatoes, soy-based sauce | 17.034 | Shrimp and vegetables excluding carrots, broccoli, and dark-green leafy; no potatoes, soy-based sauce | 17.034 |
| 27450430 | Shrimp shish kabob with vegetables, excluding potatoes | 29.019 | Shrimp shish kabob with vegetables, excluding potatoes | 29.019 |
| 27450450 | Shrimp creole, no rice | 4.074 | Shrimp creole, no rice | 4.074 |
| 27450470 | Kung Pao shrimp | 4.940 | Kung Pao shrimp | 4.940 |
| 27450510 | Tuna casserole with vegetables and mushroom sauce, no noodles | 6.517 | Tuna casserole with vegetables and mushroom sauce, no noodles | 6.517 |
| 27450600 | Shellfish mixture and vegetables including carrots, broccoli, and/or dark-green leafy; no potatoes, soy-based sauce | 17.543 | Shellfish mixture and vegetables including carrots, broccoli, and/or dark-green leafy; no potatoes, soy-based sauce | 17.543 |
| 27450610 | Shellfish mixture and vegetables excluding carrots, broccoli, and dark-green leafy; no potatoes, soy-based sauce | 17.034 | Shellfish mixture and vegetables excluding carrots, broccoli, and dark-green leafy; no potatoes, soy-based sauce | 17.034 |
| 27450650 | Shellfish mixture and vegetables including carrots, broccoli, and/or dark-green leafy; no potatoes, mushroom sauce | 1.000 | Shellfish mixture and vegetables including carrots, broccoli, and/or dark-green leafy; no potatoes, mushroom sauce | 1.000 |
| 27450700 | Fish and vegetables including carrots, broccoli, and/or dark-green leafy; no potatoes, tomato-based sauce | 1.121 | Fish and vegetables including carrots, broccoli, and/or dark-green leafy; no potatoes, tomato-based sauce | 1.121 |
| 27450740 | Fish and vegetables including carrots, broccoli, and/or dark-green leafy; no potatoes, soy-based sauce | 17.543 | Fish and vegetables including carrots, broccoli, and/or dark-green leafy; no potatoes, soy-based sauce | 17.543 |
| 27450750 | Fish and vegetables excluding carrots, broccoli, and dark-green leafy; no potatoes, soy-based sauce | 17.034 | Fish and vegetables excluding carrots, broccoli, and dark-green leafy; no potatoes, soy-based sauce | 17.034 |
| 27450760 | Fish shish kabob with vegetables, excluding potatoes | 29.019 | Fish shish kabob with vegetables, excluding potatoes | 29.019 |
| 27451010 | Fried fish with sauce, Puerto Rican style | 19.003 | Fried fish with sauce, Puerto Rican style | 19.003 |
| 27451030 | Lobster with sauce, Puerto Rican style | 10.574 | Lobster with sauce, Puerto Rican style | 10.574 |
| 27451060 | Octopus salad, Puerto Rican style | 22.605 | Octopus salad, Puerto Rican style | 22.605 |
| 27451070 | Codfish salad, Puerto Rican style, Serenata | 16.491 | Codfish salad, Puerto Rican style, Serenata | 16.491 |
| 27460010 | Chow mein or chop suey, NS as to type of meat, no noodles | 16.684 | Chow mein or chop suey, NS as to type of meat, no noodles | 16.684 |
| 27460490 | Julienne salad, meat, cheese, eggs, vegetables, no dressing | 4.607 |  |  |
| 27460710 | Livers, chicken, chopped, with eggs and onion | 26.253 | Livers, chicken, chopped, with eggs and onion | 26.253 |
| 27460750 | Liver, beef or calves, and onions | 30.525 | Liver, beef or calves, and onions | 30.525 |
| 27462000 | Stewed chitterlings, Puerto Rican style | 11.375 | Stewed chitterlings, Puerto Rican style | 11.375 |
| 27463000 | Stewed gizzards, Puerto Rican style | 11.609 | Stewed gizzards, Puerto Rican style | 11.609 |
| 27464000 | Gumbo, no rice | 9.163 | Gumbo, no rice | 9.163 |
| 27500050 | Sandwich, NFS | 19.680 | Sandwich, NFS | 19.680 |
| 27500100 | Meat sandwich, NFS | 19.680 |  |  |
| 27500200 | Wrap sandwich, filled with meat, poultry, or fish, vegetables, and cheese | 11.204 |  |  |
| 27500300 | Wrap sandwich, filled with meat, poultry, or fish, and vegetables | 11.496 | Sandwich wrap, NFS | 11.496 |
| 27510000 | Beef sandwich, NFS | 19.680 |  |  |
| 27510105 |  |  | Sloppy joe sandwich, on wheat bun | 15.568 |
| 27510110 | Beef barbecue sandwich or Sloppy Joe, on bun | 15.017 |  |  |
| 27510130 | Beef barbecue submarine sandwich, on bun | 17.871 | Barbecue beef sandwich, on white bun | 17.871 |
| 27510135 |  |  | Barbecue beef sandwich, on wheat bun | 16.000 |
| 27510140 | Cheeseburger, 1 miniature patty, plain, on miniature bun, from fast food / restaurant | 23.172 | Cheeseburger slider, from fast food | 23.172 |
| 27510145 | Cheeseburger, 1 miniature patty, with condiments, on miniature bun, from fast food / restaurant | 18.162 |  |  |
| 27510150 | Cheeseburger, 1 miniature patty, on miniature bun, from school | 23.172 |  |  |
| 27510165 | Cheeseburger, 1 small patty, with condiments, on bun, from fast food / restaurant | 13.920 |  |  |
| 27510170 | Cheeseburger, 1 small patty, with condiments, on bun, from fast food / restaurant (Burger King Cheeseburger) | 13.920 | Cheeseburger (Burger King) | 13.920 |
| 27510171 | Cheeseburger, 1 small patty, with condiments, on bun, from fast food / restaurant (Burger King WHOPPER Jr. with cheese) | 14.620 | Whopper Jr with cheese (Burger King) | 14.620 |
| 27510172 | Cheeseburger, 1 small patty, with condiments, on bun, from fast food / restaurant (McDonald's Cheeseburger) | 13.920 | Cheeseburger (McDonalds) | 13.920 |
| 27510173 | Cheeseburger, 1 small patty, with condiments, on bun, from fast food / restaurant (Wendy's Kid's Cheeseburger) | 13.920 |  |  |
| 27510174 | Cheeseburger, 1 small patty, with condiments, on bun, from fast food / restaurant (Wendy's Jr. Cheeseburger) | 13.920 |  |  |
| 27510175 | Cheeseburger, 1 small patty, with condiments, on bun, from fast food / restaurant (Wendy's Jr. Cheeseburger Deluxe) | 14.620 |  |  |
| 27510190 | Cheeseburger on bun, from school | 24.471 | Cheeseburger, from school cafeteria | 24.471 |
| 27510195 | Cheeseburger, 1 small patty, plain, on white bun | 18.087 | Cheeseburger, on white bun, 1 small patty | 18.087 |
| 27510196 | Cheeseburger, 1 small patty, plain, on wheat bun | 18.087 | Cheeseburger, on wheat bun, 1 small patty | 18.087 |
| 27510205 | Cheeseburger, 1 small patty, with condiments, on white bun | 12.755 |  |  |
| 27510206 | Cheeseburger, 1 small patty, with condiments, on wheat bun | 12.755 |  |  |
| 27510207 | Cheeseburger, 1 small patty, with condiments, on whole wheat bun | 12.755 |  |  |
| 27510225 | Cheeseburger, 1 medium patty, with condiments, on bun, from fast food / restaurant | 13.920 |  |  |
| 27510231 | Cheeseburger, 1 medium patty, with condiments, on bun, from fast food / restaurant (Burger King WHOPPER with cheese) | 13.920 | Whopper with cheese (Burger King) | 13.920 |
| 27510232 | Cheeseburger, 1 medium patty, with condiments, on bun, from fast food / restaurant (McDonald's Quarter Pounder with cheese) | 13.920 | Quarter Pounder with cheese (McDonalds) | 13.920 |
| 27510233 | Cheeseburger, 1 medium patty, with condiments, on bun, from fast food / restaurant (Wendy's 1/4 lb Single with cheese) | 13.920 |  |  |
| 27510235 | Cheeseburger submarine sandwich with lettuce, tomato and spread | 19.200 |  |  |
| 27510241 | Cheeseburger, 1 medium patty, plain, on white bun | 17.349 | Cheeseburger, on white bun, 1 medium patty | 17.349 |
| 27510242 | Cheeseburger, 1 medium patty, plain, on wheat bun | 17.349 | Cheeseburger, on wheat bun, 1 medium patty | 17.349 |
| 27510243 | Cheeseburger, 1 medium patty, plain, on whole wheat bun | 17.349 |  |  |
| 27510246 |  |  | Cheeseburger, on wheat bun, 1 large patty | 13.873 |
| 27510251 | Cheeseburger, 1 medium patty, with condiments, on white bun | 12.875 |  |  |
| 27510252 | Cheeseburger, 1 medium patty, with condiments, on wheat bun | 12.875 |  |  |
| 27510253 | Cheeseburger, 1 medium patty, with condiments, on whole wheat bun | 12.875 |  |  |
| 27510255 |  |  | Double cheeseburger, on wheat bun, 2 small patties | 12.253 |
| 27510258 |  |  | Double cheeseburger, on wheat bun, 2 medium patties | 10.553 |
| 27510261 | Cheeseburger, 1 large patty, plain, on bun, from fast food / restaurant | 14.619 | Cheeseburger, from fast food, 1 large patty | 14.619 |
| 27510263 |  |  | Double cheeseburger, on wheat bun, 2 large patties | 8.643 |
| 27510266 | Cheeseburger, 1 large patty, with condiments, on bun, from fast food / restaurant | 9.227 |  |  |
| 27510276 | Bacon cheeseburger, 1 small patty, with condiments, on bun, from fast food / restaurant | 12.754 |  |  |
| 27510312 | Bacon cheeseburger, 1 medium patty, with condiments, on bun, from fast food / restaurant | 12.976 |  |  |
| 27510331 | Bacon cheeseburger, 1 medium patty, plain, on white bun | 15.824 |  |  |
| 27510341 | Bacon cheeseburger, 1 medium patty, with condiments, on white bun | 12.017 |  |  |
| 27510342 | Bacon cheeseburger, 1 medium patty, with condiments, on wheat bun | 12.017 |  |  |
| 27510343 | Bacon cheeseburger, 1 medium patty, with condiments, on whole wheat bun | 12.017 |  |  |
| 27510346 | Bacon cheeseburger, 1 large patty, with condiments, on bun, from fast food / restaurant | 8.570 |  |  |
| 27510376 | Double cheeseburger, 2 small patties, with condiments, on bun, from fast food / restaurant | 13.920 |  |  |
| 27510386 | Double cheeseburger, 2 small patties, with condiments, on bun, from fast food / restaurant (Burger King Double Cheeseburger) | 13.920 | Double cheeseburger (Burger King) | 13.920 |
| 27510387 | Double cheeseburger, 2 small patties, with condiments, on bun, from fast food / restaurant (McDonald's Double Cheeseburger) | 13.920 | Double cheeseburger (McDonalds) | 13.920 |
| 27510388 | Double cheeseburger, 2 small patties, with condiments, on bun, from fast food / restaurant (McDonald's McDouble) | 13.920 | McDouble (McDonalds) | 13.920 |
| 27510389 | Double cheeseburger, 2 small patties, with condiments, on bun, from fast food / restaurant (McDonald's Big Mac) | 13.920 | Big Mac (McDonalds) | 13.920 |
| 27510391 | Double cheeseburger, 2 small patties, with condiments, on bun, from fast food / restaurant (Wendy's Double Stack) | 13.920 |  |  |
| 27510406 | Double cheeseburger, 2 medium patties, with condiments, on bun, from fast food / restaurant | 13.920 |  |  |
| 27510412 | Double cheeseburger, 2 medium patties, with condiments, on bun, from fast food / restaurant (McDonald's Double Quarter Pounder with Cheese) | 13.920 |  |  |
| 27510413 | Double cheeseburger, 2 medium patties, with condiments, on bun, from fast food / restaurant (Wendy's 1/2 lb Double with cheese) | 13.920 |  |  |
| 27510431 | Double bacon cheeseburger, 2 small patties, with condiments, on bun, from fast food / restaurant (Burger King Bacon Double Cheeseburger) | 11.013 |  |  |
| 27510446 | Double bacon cheeseburger, 2 medium patties, plain, on bun, from fast food / restaurant | 10.360 |  |  |
| 27510451 | Double bacon cheeseburger, 2 medium patties, with condiments, on bun, from fast food / restaurant | 8.583 |  |  |
| 27510465 | Double bacon cheeseburger, 2 medium patties, with condiments, on bun, from fast food / restaurant (Wendy's Baconator) | 8.583 |  |  |
| 27510475 | Double bacon cheeseburger, 2 large patties, with condiments, on bun, from fast food / restaurant | 13.083 |  |  |
| 27510486 | Triple cheeseburger, 3 medium patties, with condiments, on bun, from fast food / restaurant | 11.269 |  |  |
| 27510501 | Hamburger, 1 miniature patty, plain, on miniature bun, from fast food / restaurant | 28.000 | Hamburger slider, from fast food | 28.000 |
| 27510506 | Hamburger, 1 miniature patty, with condiments, on miniature bun, from fast food / restaurant | 21.000 |  |  |
| 27510511 | Hamburger, 1 miniature patty, on miniature bun, from school | 28.000 |  |  |
| 27510521 | Hamburger, NFS | 19.680 | Hamburger, NFS | 19.680 |
| 27510536 | Hamburger, 1 small patty, with condiments, on bun, from fast food / restaurant | 23.040 |  |  |
| 27510551 | Hamburger, 1 small patty, with condiments, on bun, from fast food / restaurant (Burger King Hamburger) | 23.040 | Hamburger (Burger King) | 23.040 |
| 27510552 | Hamburger, 1 small patty, with condiments, on bun, from fast food / restaurant (Burger King WHOPPER Jr.) | 16.667 | Whopper Jr (Burger King) | 16.667 |
| 27510553 | Hamburger, 1 small patty, with condiments, on bun, from fast food / restaurant (McDonald's Hamburger) | 23.040 | Hamburger (McDonalds) | 23.040 |
| 27510555 | Hamburger, 1 small patty, with condiments, on bun, from fast food / restaurant (Wendy's Jr. Hamburger) | 23.040 |  |  |
| 27510565 | Hamburger, on bun, from school | 27.130 | Hamburger, from school cafeteria | 27.130 |
| 27510575 | Hamburger, 1 small patty, plain, on white bun | 21.333 | Hamburger, on white bun, 1 small patty | 21.333 |
| 27510576 | Hamburger, 1 small patty, plain, on wheat bun | 21.333 | Hamburger, on wheat bun, 1 small patty | 21.333 |
| 27510577 | Hamburger, 1 small patty, plain, on whole wheat bun | 21.333 |  |  |
| 27510585 | Hamburger, 1 small patty, with condiments, on white bun | 14.286 |  |  |
| 27510587 | Hamburger, 1 small patty, with condiments, on whole wheat bun | 14.286 |  |  |
| 27510606 | Hamburger, 1 medium patty, with condiments, on bun, from fast food / restaurant | 13.920 |  |  |
| 27510615 | Hamburger, 1 medium patty, with condiments, on bun, from fast food / restaurant (Burger King WHOPPER) | 13.920 | Whopper (Burger King) | 13.920 |
| 27510616 | Hamburger, 1 medium patty, with condiments, on bun, from fast food / restaurant (Wendy's 1/4 lb Single ) | 13.920 |  |  |
| 27510631 | Hamburger, 1 medium patty, plain, on white bun | 19.862 | Hamburger, on white bun, 1 medium patty | 19.862 |
| 27510632 | Hamburger, 1 medium patty, plain, on wheat bun | 19.862 | Hamburger, on wheat bun, 1 medium patty | 19.862 |
| 27510633 | Hamburger, 1 medium patty, plain, on whole wheat bun | 19.862 |  |  |
| 27510636 |  |  | Hamburger, on wheat bun, 1 large patty | 16.401 |
| 27510641 | Hamburger, 1 medium patty, with condiments, on white bun | 14.207 |  |  |
| 27510642 | Hamburger, 1 medium patty, with condiments, on wheat bun | 14.207 |  |  |
| 27510643 | Hamburger, 1 medium patty, with condiments, on whole wheat bun | 14.207 |  |  |
| 27510652 |  |  | Double hamburger, on wheat bun, 2 small patties | 13.662 |
| 27510657 |  |  | Double hamburger, on wheat bun, 2 medium patties | 12.473 |
| 27510659 |  |  | Double hamburger, on wheat bun, 2 large patties | 9.890 |
| 27510661 | Double hamburger, 2 small patties, plain, on bun, from fast food / restaurant | 18.838 | Double hamburger, from fast food, 2 small patties | 18.838 |
| 27510667 | Double hamburger, 2 small patties, with condiments, on bun, from fast food / restaurant | 13.158 |  |  |
| 27510671 | Double hamburger, 2 medium patties, plain, on bun, from fast food / restaurant | 13.061 | Double hamburger, from fast food, 2 medium patties | 13.061 |
| 27510700 | Meatball and spaghetti sauce submarine sandwich | 26.880 | Meatball sandwich or sub | 26.880 |
| 27510705 | Chiliburger, with or without cheese, on bun | 15.220 | Chiliburger, with or without cheese, on bun | 15.220 |
| 27510910 | Corned beef sandwich | 18.837 | Corned beef sandwich on white | 18.837 |
| 27510930 |  |  | Corned beef sandwich on wheat | 24.828 |
| 27510940 |  |  | Corned beef sandwich on wheat, with cheese | 21.022 |
| 27510950 | Reuben sandwich, corned beef sandwich with sauerkraut and cheese, with spread | 16.239 | Reuben sandwich | 16.239 |
| 27511010 | Pastrami sandwich | 18.837 |  |  |
| 27513010 | Roast beef sandwich | 19.680 | Roast beef sandwich on white | 19.680 |
| 27513020 | Roast beef sandwich, with gravy | 11.742 |  |  |
| 27513040 | Roast beef submarine sandwich, with lettuce, tomato and spread | 17.371 |  |  |
| 27513041 | Roast beef submarine sandwich, with cheese, lettuce, tomato and spread | 16.582 |  |  |
| 27513050 | Roast beef sandwich with cheese | 16.536 | Roast beef sandwich on white, with cheese | 16.536 |
| 27513055 |  |  | Roast beef sandwich on wheat | 24.828 |
| 27513060 | Roast beef sandwich with bacon and cheese sauce | 18.334 |  |  |
| 27513065 |  |  | Roast beef sandwich on wheat, with cheese | 21.022 |
| 27513070 | Roast beef submarine sandwich, on roll, au jus | 16.789 | French dip sandwich | 16.789 |
| 27514020 |  |  | Steak sandwich or sub on wheat | 24.828 |
| 27514040 |  |  | Cheese steak sandwich or sub on wheat | 20.000 |
| 27515000 | Steak submarine sandwich with lettuce and tomato | 18.894 |  |  |
| 27515010 | Steak sandwich, plain, on roll | 15.806 |  |  |
| 27515020 | Steak and cheese submarine sandwich, with lettuce and tomato | 19.200 |  |  |
| 27515030 | Steak and cheese sandwich, plain, on roll | 15.953 |  |  |
| 27515040 | Steak and cheese submarine sandwich, plain, on roll | 19.510 |  |  |
| 27515050 | Fajita-style beef sandwich with cheese, on pita bread, with lettuce and tomato | 27.037 |  |  |
| 27515070 | Steak and cheese submarine sandwich, with fried peppers and onions, on roll | 25.176 |  |  |
| 27516010 | Gyro sandwich (pita bread, beef, lamb, onion, condiments), with tomato and spread | 24.229 | Gyro sandwich | 24.229 |
| 27517000 | Wrap sandwich filled with beef patty, cheese and spread and/or sauce | 20.415 | Hamburger wrap sandwich, from fast food | 20.415 |
| 27520110 | Bacon sandwich, with spread | 29.033 |  |  |
| 27520120 | Bacon and cheese sandwich, with spread | 21.455 |  |  |
| 27520135 | Bacon, chicken, and tomato club sandwich, with cheese, lettuce and spread | 18.720 |  |  |
| 27520140 | Bacon and egg sandwich | 20.211 |  |  |
| 27520150 | Bacon, lettuce, and tomato sandwich with spread | 16.911 |  |  |
| 27520155 | Bacon, lettuce, and tomato submarine sandwich, with spread | 22.080 |  |  |
| 27520156 | Bacon, lettuce, tomato, and cheese submarine sandwich, with spread | 12.759 |  |  |
| 27520160 | Bacon, chicken, and tomato club sandwich, on multigrain roll with lettuce and spread | 11.308 |  |  |
| 27520165 | Bacon, breaded fried chicken fillet, and tomato club with lettuce and spread | 18.669 |  |  |
| 27520230 |  |  | Ham sandwich on wheat | 24.828 |
| 27520240 |  |  | Ham sandwich on wheat, with cheese | 21.022 |
| 27520250 | Ham on biscuit | 41.760 |  |  |
| 27520300 | Ham sandwich, with spread | 22.480 |  |  |
| 27520310 | Ham sandwich with lettuce and spread | 21.158 |  |  |
| 27520320 | Ham and cheese sandwich, with lettuce and spread | 17.304 |  |  |
| 27520330 | Ham and egg sandwich | 20.621 |  |  |
| 27520340 | Ham salad sandwich | 18.562 | Ham salad sandwich on white | 18.562 |
| 27520345 |  |  | Ham salad sandwich on wheat | 16.000 |
| 27520350 | Ham and cheese sandwich, with spread, grilled | 17.188 |  |  |
| 27520360 | Ham and cheese sandwich, on bun, with lettuce and spread | 13.031 |  |  |
| 27520370 | Hot ham and cheese sandwich, on bun | 12.511 |  |  |
| 27520380 | Ham and cheese on English muffin | 19.200 |  |  |
| 27520390 | Ham and cheese submarine sandwich, with lettuce, tomato and spread | 18.240 |  |  |
| 27520410 | Cuban sandwich, with spread | 21.550 | Cuban sandwich | 21.550 |
| 27520420 | Midnight sandwich, with spread | 17.114 |  |  |
| 27520500 | Pork sandwich, on white roll, with onions, dill pickles and barbecue sauce | 21.684 | Barbecue rib sandwich | 21.684 |
| 27520510 | Pork barbecue sandwich or Sloppy Joe, on bun | 12.232 | Barbecue pork sandwich, on white bun | 12.232 |
| 27520515 |  |  | Barbecue pork sandwich, on wheat bun | 16.000 |
| 27520520 | Pork sandwich | 23.746 | Pork sandwich | 23.746 |
| 27520530 | Pork sandwich, with gravy | 13.870 |  |  |
| 27520540 | Ham and tomato club sandwich, with lettuce and spread | 13.551 |  |  |
| 27520620 |  |  | Bacon, lettuce, tomato sandwich on wheat | 27.692 |
| 27540030 |  |  | Turkey sandwich on wheat | 24.828 |
| 27540040 |  |  | Turkey sandwich on wheat, with cheese | 21.022 |
| 27540110 | Chicken sandwich, with spread | 22.890 |  |  |
| 27540111 | Chicken sandwich, with cheese and spread | 19.417 |  |  |
| 27540120 | Chicken salad or chicken spread sandwich | 18.784 | Chicken salad sandwich on white | 18.784 |
| 27540121 |  |  | Chicken salad sandwich on wheat | 16.000 |
| 27540125 |  |  | Club sandwich on wheat | 21.818 |
| 27540126 |  |  | Club sandwich on wheat, with cheese | 18.824 |
| 27540130 | Chicken barbecue sandwich | 21.470 | Barbecue chicken sandwich, on white bun | 21.470 |
| 27540131 |  |  | Barbecue chicken sandwich, on wheat bun | 16.000 |
| 27540139 |  |  | Chicken fillet sandwich, from school cafeteria | 27.130 |
| 27540170 | Chicken patty sandwich, miniature, with spread | 20.325 |  |  |
| 27540185 |  |  | Chicken fillet sandwich, fried, on wheat bun | 18.462 |
| 27540186 |  |  | Chicken fillet sandwich, fried, on wheat bun, with cheese | 16.098 |
| 27540200 | Fajita-style chicken sandwich with cheese, on pita bread, with lettuce and tomato | 26.896 |  |  |
| 27540205 |  |  | Chicken fillet sandwich, grilled, on wheat bun | 18.857 |
| 27540206 |  |  | Chicken fillet sandwich, grilled, on wheat bun, with cheese | 16.398 |
| 27540230 | Chicken patty sandwich with cheese, on wheat bun, with lettuce, tomato and spread | 15.742 |  |  |
| 27540240 | Chicken fillet, broiled, sandwich, on whole wheat roll, with lettuce, tomato and spread | 17.896 |  |  |
| 27540250 | Chicken fillet, broiled, sandwich with cheese, on whole wheat roll, with lettuce, tomato and non-mayonnaise type spread | 15.679 |  |  |
| 27540260 | Chicken fillet, broiled, sandwich, on oat bran bun, with lettuce, tomato, spread | 16.131 |  |  |
| 27540280 | Chicken fillet, broiled, sandwich with cheese, on bun, with lettuce, tomato and spread | 17.760 |  |  |
| 27540285 | Chicken, bacon, and tomato club sandwich, with lettuce and spread | 18.720 |  |  |
| 27540290 | Chicken submarine sandwich, with lettuce, tomato and spread | 18.240 |  |  |
| 27540291 | Chicken submarine sandwich, with cheese, lettuce, tomato and spread | 18.240 |  |  |
| 27540295 | Buffalo chicken submarine sandwich | 15.844 |  |  |
| 27540296 | Buffalo chicken submarine sandwich with cheese | 14.195 |  |  |
| 27540310 | Turkey sandwich, with spread | 18.135 |  |  |
| 27540320 | Turkey salad or turkey spread sandwich | 18.538 |  |  |
| 27540330 | Turkey sandwich, with gravy | 9.295 |  |  |
| 27540350 | Turkey submarine sandwich, with cheese, lettuce, tomato and spread | 17.760 |  |  |
| 27540360 | Turkey and bacon submarine sandwich, with lettuce, tomato and spread | 15.282 |  |  |
| 27540361 | Turkey and bacon submarine sandwich, with cheese, lettuce, tomato and spread | 13.521 |  |  |
| 27541000 | Turkey, ham, and roast beef club sandwich, with lettuce, tomato and spread | 19.430 |  |  |
| 27541001 | Turkey, ham, and roast beef club sandwich with cheese, lettuce, tomato, and spread | 17.760 |  |  |
| 27545000 | Turkey or chicken burger, plain, on bun, from fast food / restaurant | 19.862 |  |  |
| 27545010 | Turkey or chicken burger, with condiments, on bun, from fast food / restaurant | 14.207 |  |  |
| 27545100 | Turkey or chicken burger, plain, on white bun | 19.862 | Turkey or chicken burger, on white bun | 19.862 |
| 27545110 | Turkey or chicken burger, plain, on wheat bun | 19.862 | Turkey or chicken burger, on wheat bun | 19.862 |
| 27545200 | Turkey or chicken burger, with condiments, on white bun | 14.207 |  |  |
| 27545210 | Turkey or chicken burger, with condiments, on wheat bun | 14.207 |  |  |
| 27545220 | Turkey or chicken burger, with condiments, on whole wheat bun | 14.207 |  |  |
| 27550110 | Crab cake sandwich, on bun | 19.200 | Crab cake sandwich | 19.200 |
| 27550200 |  |  | Fish sandwich, from school cafeteria | 26.667 |
| 27550410 |  |  | Fish sandwich, fried, on wheat bun | 20.122 |
| 27550415 |  |  | Fish sandwich, fried, on wheat bun, with cheese | 17.346 |
| 27550510 | Sardine sandwich, with lettuce and spread | 14.628 | Sardine sandwich | 14.628 |
| 27550710 | Tuna salad sandwich, with lettuce | 21.920 |  |  |
| 27550720 | Tuna salad sandwich | 23.248 | Tuna salad sandwich on white | 23.248 |
| 27550730 | Tuna melt sandwich | 12.632 | Tuna salad sandwich on white, with cheese | 12.632 |
| 27550735 |  |  | Tuna salad sandwich on wheat | 16.000 |
| 27550737 |  |  | Tuna salad sandwich on wheat, with cheese | 14.328 |
| 27560110 | Bologna sandwich, with spread | 30.225 |  |  |
| 27560120 | Bologna and cheese sandwich, with spread | 22.919 |  |  |
| 27560150 |  |  | Bologna sandwich on wheat | 24.828 |
| 27560160 |  |  | Bologna sandwich on wheat, with cheese | 21.022 |
| 27560350 | Pig in a blanket, frankfurter or hot dog wrapped in dough | 15.944 | Pig in a blanket | 15.944 |
| 27560410 | Puerto Rican sandwich | 28.462 |  |  |
| 27560500 | Pepperoni and salami submarine sandwich, with lettuce, tomato and spread | 21.402 |  |  |
| 27560510 | Salami sandwich, with spread | 30.344 |  |  |
| 27560540 |  |  | Salami sandwich on wheat | 24.828 |
| 27560550 |  |  | Salami sandwich on wheat, with cheese | 21.022 |
| 27560620 |  |  | Italian sandwich on wheat | 17.455 |
| 27560650 | Sausage on biscuit | 41.760 |  |  |
| 27560660 | Sausage griddle cake sandwich | 4.176 |  |  |
| 27560670 | Sausage and cheese on English muffin | 19.200 |  |  |
| 27560705 | Sausage balls, made with biscuit mix and cheese | 15.917 |  |  |
| 27560710 | Sausage sandwich | 23.851 | Italian sausage sandwich on white | 23.851 |
| 27560715 |  |  | Italian sausage sandwich on wheat | 21.333 |
| 27560720 | Sausage and spaghetti sauce sandwich | 15.340 |  |  |
| 27560910 | Cold cut sumarine sandwich, with cheese, lettuce, tomato and spread | 17.760 |  |  |
| 27563010 | Meat spread or potted meat sandwich | 23.851 |  |  |
| 27564000 | Frankfurter or hot dog sandwich, NFS, plain, on white bun | 21.176 | Hot dog sandwich, NFS, on white bun | 21.176 |
| 27564001 | Frankfurter or hot dog sandwich, NFS, plain, on wheat bun | 21.176 | Hot dog sandwich, NFS, on wheat bun | 21.176 |
| 27564002 | Frankfurter or hot dog sandwich, NFS, plain, on whole wheat bun | 21.176 |  |  |
| 27564003 | Frankfurter or hot dog sandwich, NFS, plain, on whole grain white bun | 21.176 |  |  |
| 27564004 | Frankfurter or hot dog sandwich, NFS, plain, on multigrain bun | 21.176 |  |  |
| 27564010 | Frankfurter or hot dog sandwich, NFS, plain, on white bread | 15.812 | Hot dog sandwich, NFS, on white bread | 15.812 |
| 27564020 | Frankfurter or hot dog sandwich, NFS, plain, on wheat bread | 15.812 | Hot dog sandwich, NFS, on wheat bread | 15.812 |
| 27564030 | Frankfurter or hot dog sandwich, NFS, plain, on whole wheat bread | 18.581 |  |  |
| 27564040 | Frankfurter or hot dog sandwich, NFS, plain, on whole grain white bread | 18.581 |  |  |
| 27564050 | Frankfurter or hot dog sandwich, NFS, plain, on multigrain bread | 18.581 |  |  |
| 27564060 | Frankfurter or hot dog sandwich, beef, plain, on white bun | 21.176 | Hot dog sandwich, beef, on white bun | 21.176 |
| 27564061 | Frankfurter or hot dog sandwich, beef, plain, on wheat bun | 21.176 | Hot dog sandwich, beef, on wheat bun | 21.176 |
| 27564062 | Frankfurter or hot dog sandwich, beef, plain, on whole wheat bun | 21.176 |  |  |
| 27564063 | Frankfurter or hot dog sandwich, beef, plain, on whole grain white bun | 21.176 |  |  |
| 27564064 | Frankfurter or hot dog sandwich, beef, plain, on multigrain bun | 21.176 |  |  |
| 27564070 | Frankfurter or hot dog sandwich, beef, plain, on white bread | 15.812 | Hot dog sandwich, beef, on white bread | 15.812 |
| 27564080 | Frankfurter or hot dog sandwich, beef, plain, on wheat bread | 15.812 | Hot dog sandwich, beef, on wheat bread | 15.812 |
| 27564090 | Frankfurter or hot dog sandwich, beef, plain, on whole wheat bread | 18.581 |  |  |
| 27564100 | Frankfurter or hot dog sandwich, beef, plain, on whole grain white bread | 18.581 |  |  |
| 27564110 | Frankfurter or hot dog sandwich, beef, plain, on multigrain bread | 18.581 |  |  |
| 27564120 | Frankfurter or hot dog sandwich, beef and pork, plain, on white bun | 21.176 |  |  |
| 27564121 | Frankfurter or hot dog sandwich, beef and pork, plain, on wheat bun | 21.176 |  |  |
| 27564122 | Frankfurter or hot dog sandwich, beef and pork, plain, on whole wheat bun | 21.176 |  |  |
| 27564123 | Frankfurter or hot dog sandwich, beef and pork, plain, on whole grain white bun | 21.176 |  |  |
| 27564124 | Frankfurter or hot dog sandwich, beef and pork, plain, on multigrain bun | 21.176 |  |  |
| 27564130 | Frankfurter or hot dog sandwich, beef and pork, plain, on white bread | 15.812 |  |  |
| 27564140 | Frankfurter or hot dog sandwich, beef and pork, plain, on wheat bread | 15.812 |  |  |
| 27564150 | Frankfurter or hot dog sandwich, beef and pork, plain, on whole wheat bread | 18.581 |  |  |
| 27564160 | Frankfurter or hot dog sandwich, beef and pork, plain, on whole grain white bread | 18.581 |  |  |
| 27564170 | Frankfurter or hot dog sandwich, beef and pork, plain, on multigrain bread | 18.581 |  |  |
| 27564180 | Frankfurter or hot dog sandwich, meat and poultry, plain, on white bun | 21.176 | Hot dog sandwich, meat and poultry, on white bun | 21.176 |
| 27564181 | Frankfurter or hot dog sandwich, meat and poultry, plain, on wheat bun | 21.176 | Hot dog sandwich, meat and poultry, on wheat bun | 21.176 |
| 27564182 | Frankfurter or hot dog sandwich, meat and poultry, plain, on whole wheat bun | 21.176 |  |  |
| 27564183 | Frankfurter or hot dog sandwich, meat and poultry, plain, on whole grain white bun | 21.176 |  |  |
| 27564184 | Frankfurter or hot dog sandwich, meat and poultry, plain, on multigrain bun | 21.176 |  |  |
| 27564190 | Frankfurter or hot dog sandwich, meat and poultry, plain, on white bread | 15.812 | Hot dog sandwich, meat and poultry, on white bread | 15.812 |
| 27564200 | Frankfurter or hot dog sandwich, meat and poultry, plain, on wheat bread | 15.812 | Hot dog sandwich, meat and poultry, on wheat bread | 15.812 |
| 27564210 | Frankfurter or hot dog sandwich, meat and poultry, plain, on whole wheat bread | 18.581 |  |  |
| 27564220 | Frankfurter or hot dog sandwich, meat and poultry, plain, on whole grain white bread | 18.581 |  |  |
| 27564230 | Frankfurter or hot dog sandwich, meat and poultry, plain, on multigrain bread | 18.581 |  |  |
| 27564240 | Frankfurter or hot dog sandwich, chicken and/or turkey, plain, on white bun | 21.176 | Hot dog sandwich, turkey, on white bun | 21.176 |
| 27564241 | Frankfurter or hot dog sandwich, chicken and/or turkey, plain, on wheat bun | 21.176 | Hot dog sandwich, turkey, on wheat bun | 21.176 |
| 27564242 | Frankfurter or hot dog sandwich, chicken and/or turkey, plain, on whole wheat bun | 21.176 |  |  |
| 27564243 | Frankfurter or hot dog sandwich, chicken and/or turkey, plain, on whole grain white bun | 21.176 |  |  |
| 27564244 | Frankfurter or hot dog sandwich, chicken and/or turkey, plain, on multigrain bun | 21.176 |  |  |
| 27564250 | Frankfurter or hot dog sandwich, chicken and/or turkey, plain, on white bread | 15.812 | Hot dog sandwich, turkey, on white bread | 15.812 |
| 27564260 | Frankfurter or hot dog sandwich, chicken and/or turkey, plain, on wheat bread | 15.812 | Hot dog sandwich, turkey, on wheat bread | 15.812 |
| 27564270 | Frankfurter or hot dog sandwich, chicken and/or turkey, plain, on whole wheat bread | 18.581 |  |  |
| 27564280 | Frankfurter or hot dog sandwich, chicken and/or turkey, plain, on whole grain white bread | 18.581 |  |  |
| 27564290 | Frankfurter or hot dog sandwich, chicken and/or turkey, plain, on multigrain bread | 18.581 |  |  |
| 27564300 | Frankfurter or hot dog sandwich, reduced fat or light, plain, on white bun | 21.176 | Hot dog sandwich, reduced fat, on white bun | 21.176 |
| 27564301 | Frankfurter or hot dog sandwich, reduced fat or light, plain, on wheat bun | 21.176 | Hot dog sandwich, reduced fat, on wheat bun | 21.176 |
| 27564302 | Frankfurter or hot dog sandwich, reduced fat or light, plain, on whole wheat bun | 21.176 |  |  |
| 27564303 | Frankfurter or hot dog sandwich, reduced fat or light, plain, on whole grain white bun | 21.176 |  |  |
| 27564304 | Frankfurter or hot dog sandwich, reduced fat or light, plain, on multigrain bun | 21.176 |  |  |
| 27564310 | Frankfurter or hot dog sandwich, reduced fat or light, plain, on white bread | 15.812 | Hot dog sandwich, reduced fat, on white bread | 15.812 |
| 27564320 | Frankfurter or hot dog sandwich, reduced fat or light, plain, on wheat bread | 15.812 | Hot dog sandwich, reduced fat, on wheat bread | 15.812 |
| 27564330 | Frankfurter or hot dog sandwich, reduced fat or light, plain, on whole wheat bread | 18.581 |  |  |
| 27564340 | Frankfurter or hot dog sandwich, reduced fat or light, plain, on whole grain white bread | 18.581 |  |  |
| 27564350 | Frankfurter or hot dog sandwich, reduced fat or light, plain, on multigrain bread | 18.581 |  |  |
| 27564360 | Frankfurter or hot dog sandwich, fat free, plain, on white bun | 21.176 |  |  |
| 27564361 | Frankfurter or hot dog sandwich, fat free, plain, on wheat bun | 21.176 |  |  |
| 27564362 | Frankfurter or hot dog sandwich, fat free, plain, on whole wheat bun | 21.176 |  |  |
| 27564363 | Frankfurter or hot dog sandwich, fat free, plain, on whole grain white bun | 21.176 |  |  |
| 27564364 | Frankfurter or hot dog sandwich, fat free, plain, on multigrain bun | 21.176 |  |  |
| 27564370 | Frankfurter or hot dog sandwich, fat free, plain, on white bread | 15.812 |  |  |
| 27564380 | Frankfurter or hot dog sandwich, fat free, plain, on wheat bread | 15.812 |  |  |
| 27564390 | Frankfurter or hot dog sandwich, fat free, plain, on whole wheat bread | 18.581 |  |  |
| 27564400 | Frankfurter or hot dog sandwich, fat free, plain, on whole grain white bread | 18.581 |  |  |
| 27564410 | Frankfurter or hot dog sandwich, fat free, plain, on multigrain bread | 18.581 |  |  |
| 27564420 | Frankfurter or hot dog sandwich, meatless, plain, on bun | 18.783 | Hot dog sandwich, vegetarian, on bun | 18.783 |
| 27564430 | Frankfurter or hot dog sandwich, meatless, plain, on bread | 13.714 | Hot dog sandwich, vegetarian, on bread | 13.714 |
| 27564440 | Frankfurter or hot dog sandwich, with chili, on white bun | 13.012 | Chili hot dog sandwich, on white bun | 13.012 |
| 27564441 | Frankfurter or hot dog sandwich, with chili, on wheat bun | 13.012 | Chili hot dog sandwich, on wheat bun | 13.012 |
| 27564442 | Frankfurter or hot dog sandwich, with chili, on whole wheat bun | 13.012 |  |  |
| 27564443 | Frankfurter or hot dog sandwich, with chili, on whole grain white bun | 13.012 |  |  |
| 27564444 | Frankfurter or hot dog sandwich, with chili, on multigrain bun | 13.012 |  |  |
| 27564450 | Frankfurter or hot dog sandwich, with chili, on white bread | 9.020 | Chili hot dog sandwich, on white bread | 9.020 |
| 27564460 | Frankfurter or hot dog sandwich, with chili, on wheat bread | 9.020 | Chili hot dog sandwich, on wheat bread | 9.020 |
| 27564470 | Frankfurter or hot dog sandwich, with chili, on whole wheat bread | 11.006 |  |  |
| 27564480 | Frankfurter or hot dog sandwich, with chili, on whole grain white bread | 11.006 |  |  |
| 27564490 | Frankfurter or hot dog sandwich, with chili, on multigrain bread | 11.006 |  |  |
| 27564500 | Frankfurter or hot dog sandwich, with meatless chili, on white bun | 13.012 |  |  |
| 27564501 | Frankfurter or hot dog sandwich, with meatless chili, on wheat bun | 13.012 |  |  |
| 27564502 | Frankfurter or hot dog sandwich, with meatless chili, on whole wheat bun | 13.012 |  |  |
| 27564503 | Frankfurter or hot dog sandwich, with meatless chili, on whole grain white bun | 13.012 |  |  |
| 27564504 | Frankfurter or hot dog sandwich, with meatless chili, on multigrain bun | 13.012 |  |  |
| 27564510 | Frankfurter or hot dog sandwich, with meatless chili, on white bread | 9.020 |  |  |
| 27564520 | Frankfurter or hot dog sandwich, with meatless chili, on wheat bread | 9.020 |  |  |
| 27564530 | Frankfurter or hot dog sandwich, with meatless chili, on whole wheat bread | 11.006 |  |  |
| 27564540 | Frankfurter or hot dog sandwich, with meatless chili, on whole grain white bread | 11.006 |  |  |
| 27564550 | Frankfurter or hot dog sandwich, with meatless chili, on multigrain bread | 11.006 |  |  |
| 27564560 | Frankfurter or hot dog sandwich, meatless, on bun, with meatless chili | 12.067 |  |  |
| 27564570 | Frankfurter or hot dog sandwich, meatless, on bread, with meatless chili | 8.296 |  |  |
| 27570310 | Hors d'oeuvres, with spread | 26.593 |  |  |
| 27580030 |  |  | Multiple meat sandwich on wheat | 20.000 |
| 27580040 |  |  | Multiple meat sandwich on wheat, with cheese | 17.455 |
| 27580130 |  |  | Turkey and ham sandwich on wheat | 20.000 |
| 27580140 |  |  | Turkey and ham sandwich on wheat, with cheese | 17.455 |
| 28110150 | Beef with vegetable, diet frozen meal | 0.279 | Beef with vegetable, diet frozen meal | 0.279 |
| 28110220 | Sirloin, chopped, with gravy, mashed potatoes, vegetable, frozen meal | 3.012 |  |  |
| 28110250 | Sirloin tips, with gravy, potatoes, vegetable, frozen meal | 2.741 |  |  |
| 28110270 | Sirloin beef, with gravy, potatoes, vegetable, frozen meal | 1.009 |  |  |
| 28110310 | Salisbury steak with gravy, potatoes, vegetable, frozen meal | 3.250 | Salisbury steak with gravy, potatoes, vegetable, frozen meal | 3.250 |
| 28110330 | Salisbury steak with gravy, whipped potatoes, vegetable, dessert, frozen meal | 3.953 | Salisbury steak with gravy, whipped potatoes, vegetable, dessert, frozen meal | 3.953 |
| 28110340 | Salisbury steak with gravy, potatoes, vegetable, soup or macaroni and cheese, dessert, frozen meal | 4.061 |  |  |
| 28110350 | Salisbury steak with gravy, potatoes, vegetable, dessert, frozen meal | 8.710 |  |  |
| 28110370 | Salisbury steak with gravy, macaroni and cheese, vegetable, frozen meal | 8.454 |  |  |
| 28110380 | Salisbury steak with gravy, macaroni and cheese, frozen meal | 17.011 | Salisbury steak with gravy, macaroni and cheese, frozen meal | 17.011 |
| 28110390 | Salisbury steak, potatoes, vegetable, dessert, diet frozen meal | 0.081 |  |  |
| 28110510 | Beef, sliced, with gravy, potatoes, vegetable, frozen meal | 1.606 | Beef, sliced, with gravy, potatoes, vegetable, frozen meal | 1.606 |
| 28110640 | Meatballs, Swedish, in sauce, with noodles, frozen meal | 18.558 |  |  |
| 28110660 | Meatballs, Swedish, in gravy, with noodles, diet frozen meal | 14.620 | Meatballs, Swedish, in gravy, with noodles, diet frozen meal | 14.620 |
| 28113110 | Salisbury steak, baked, with tomato sauce, vegetable, diet frozen meal | 2.938 |  |  |
| 28133110 | Veal, breaded, with spaghetti, in tomato sauce, frozen meal | 12.048 |  |  |
| 28133340 | Veal parmigiana with vegetable, fettuccine alfredo, dessert, frozen meal | 6.802 |  |  |
| 28140100 | Chicken dinner, NFS, frozen meal | 24.960 | Chicken dinner, NFS, frozen meal | 24.960 |
| 28140150 | Chicken divan, frozen meal | 2.549 |  |  |
| 28140250 | Chicken, boneless, with gravy, dressing, rice, vegetable, dessert, frozen meal | 1.889 |  |  |
| 28140320 | Chicken and noodles with vegetable, dessert, frozen meal | 16.588 |  |  |
| 28140720 | Chicken patty, or nuggets, boneless, breaded, potatoes, vegetable, frozen meal | 1.258 | Chicken patty, or nuggets, boneless, breaded, potatoes, vegetable, frozen meal | 1.258 |
| 28140730 | Chicken patty, breaded, with tomato sauce and cheese, fettuccine alfredo, vegetable, frozen meal | 7.920 |  |  |
| 28140740 | Chicken patty or nuggets, boneless, breaded, with pasta and tomato sauce, fruit, dessert, frozen meal | 11.838 |  |  |
| 28141010 | Chicken, fried, with potatoes, vegetable, dessert, frozen meal, large meat portion | 0.451 | Chicken, fried, with potatoes, vegetable, dessert, frozen meal, large meat portion | 0.451 |
| 28141050 | Chicken patty parmigiana, breaded, with vegetable, diet frozen meal | 4.379 |  |  |
| 28141250 | Chicken with rice and vegetable, diet frozen meal | 8.245 | Chicken with rice and vegetable, diet frozen meal | 8.245 |
| 28141610 | Chicken and vegetables in cream or white sauce, diet frozen meal | 0.338 |  |  |
| 28141650 | Chicken and vegetables au gratin with rice, diet frozen entree | 2.354 | Chicken and vegetables au gratin with rice, diet frozen entree | 2.354 |
| 28143020 | Chicken and vegetable entree with rice, diet frozen meal | 6.472 | Chicken and vegetable entree with rice, diet frozen meal | 6.472 |
| 28143040 | Chicken chow mein with rice, diet frozen meal | 5.127 |  |  |
| 28143080 | Chicken with noodles and cheese sauce, diet frozen meal | 17.520 | Chicken with noodles and cheese sauce, diet frozen meal | 17.520 |
| 28143110 | Chicken cacciatore with noodles, diet frozen meal | 16.351 |  |  |
| 28143130 | Chicken and vegetable entree with noodles, frozen meal | 18.977 |  |  |
| 28143150 | Chicken and vegetable entree with noodles, diet frozen meal | 13.352 | Chicken and vegetable entree with noodles, diet frozen meal | 13.352 |
| 28143170 | Chicken in cream sauce with noodles and vegetable, frozen meal | 21.606 |  |  |
| 28143190 | Chicken in mushroom sauce, white and wild rice, vegetable, frozen meal | 0.968 |  |  |
| 28143200 | Chicken in soy-based sauce, rice and vegetables, frozen meal | 1.729 | Chicken in soy-based sauce, rice and vegetables, frozen meal | 1.729 |
| 28143210 | Chicken in orange sauce with almond rice, diet frozen meal | 0.406 |  |  |
| 28144100 | Chicken and vegetable entree with noodles and cream sauce, frozen meal | 17.340 | Chicken and vegetable entree with noodles and cream sauce, frozen meal | 17.340 |
| 28145100 | Turkey with gravy, dressing, vegetable and fruit, diet frozen meal | 10.963 |  |  |
| 28145110 | Turkey with vegetable, stuffing, diet frozen meal | 4.827 | Turkey with vegetable, stuffing, diet frozen meal | 4.827 |
| 28145610 | Turkey with gravy, dressing, potatoes, vegetable, dessert, frozen meal | 3.870 |  |  |
| 28145710 | Turkey tetrazzini, frozen meal | 13.290 |  |  |
| 28150210 | Haddock with chopped spinach, diet frozen meal | 0.394 |  |  |
| 28150220 | Flounder with chopped broccoli, diet frozen meal | 2.527 |  |  |
| 28150510 | Fish in lemon-butter sauce with starch item, vegetable, frozen meal | 1.312 | Fish in lemon-butter sauce with starch item, vegetable, frozen meal | 1.312 |
| 28153010 | Shrimp and clams in tomato-based sauce, with noodles, frozen meal | 18.608 |  |  |
| 28154010 | Shrimp and vegetables in sauce with noodles, diet frozen meal | 16.759 | Shrimp and vegetables in sauce with noodles, diet frozen meal | 16.759 |
| 28160310 | Meat loaf with potatoes, vegetable, frozen meal | 7.804 | Meat loaf with potatoes, vegetable, frozen meal | 7.804 |
| 28160650 | Stuffed green pepper, frozen meal | 3.386 |  |  |
| 28160710 | Stuffed cabbage, with meat and tomato sauce, diet frozen meal | 8.342 |  |  |
| 28310150 | Oxtail soup | 2.581 |  |  |
| 28310160 | Beef broth, with tomato, home recipe | 5.297 |  |  |
| 28310230 | Meatball soup, home recipe, Mexican style | 6.046 | Soup, meatball | 6.046 |
| 28310320 | Beef noodle soup, Puerto Rican style | 3.090 |  |  |
| 28310330 | Pho | 1.966 | Soup, pho, with meat | 1.966 |
| 28310335 |  |  | Soup, pho, no meat | 3.979 |
| 28310420 | Beef and rice soup, Puerto Rican style | 2.808 |  |  |
| 28311010 | Pepperpot soup | 2.259 | Soup, pepperpot | 2.259 |
| 28311020 | Menudo soup, home recipe | 1.935 |  |  |
| 28311030 | Menudo soup, canned, prepared with water or ready-to-serve | 2.007 |  |  |
| 28315140 | Beef vegetable soup, home recipe, Mexican style | 3.441 | Soup, sopa or caldo de res | 3.441 |
| 28315160 | Italian Wedding Soup | 9.299 | Soup, Italian wedding | 9.299 |
| 28320130 | Ham, rice, and potato soup, Puerto Rican style | 3.952 |  |  |
| 28320140 | Ham, noodle, and vegetable soup, Puerto Rican style | 2.979 |  |  |
| 28320160 | Pork vegetable soup with potato, pasta, or rice, stew type, chunky style | 4.680 | Soup, pork or ham | 4.680 |
| 28320300 | Pork with vegetable excluding carrots, broccoli and/or dark-green leafy; soup, Asian Style | 3.876 |  |  |
| 28331110 | Lamb, pasta, and vegetable soup, Puerto Rican style | 2.816 |  |  |
| 28340130 | Chicken or turkey broth, with tomato, home recipe | 5.297 |  |  |
| 28340150 | Mexican style chicken broth soup stock | 4.084 |  |  |
| 28340210 | Chicken rice soup, Puerto Rican style | 3.665 |  |  |
| 28340220 | Chicken soup with noodles and potatoes, Puerto Rican style | 4.530 |  |  |
| 28340550 | Sweet and sour soup | 1.059 |  |  |
| 28340580 | Chicken or turkey soup with vegetables, broccoli, carrots, celery, potatoes and onions, Asian style | 2.551 |  |  |
| 28340590 | Chicken or turkey corn soup with noodles, home recipe | 6.398 |  |  |
| 28340660 | Chicken or turkey vegetable soup, home recipe | 4.699 | Soup, chicken | 4.699 |
| 28340670 | Chicken or turkey vegetable soup with rice, home recipe, Mexican style | 3.262 | Soup, sopa or caldo de pollo | 3.262 |
| 28345110 |  |  | Soup, cream of chicken | 1.427 |
| 28350050 | Fish chowder | 7.341 |  |  |
| 28350110 | Crab soup, NS as to tomato-base or cream style | 4.337 |  |  |
| 28350120 | Crab soup, tomato-base | 1.545 |  |  |
| 28350310 | Turtle and vegetable soup | 4.509 |  |  |
| 28351110 | Fish and vegetable soup, no potatoes, Mexican style | 4.989 |  |  |
| 28351120 | Fish soup with potatoes, Mexican style | 5.285 |  |  |
| 28351160 | Codfish, rice, and vegetable soup, Puerto Rican style | 4.243 |  |  |
| 28351170 | Codfish soup with noodles, Puerto Rican style | 13.750 |  |  |
| 28355110 |  |  | Soup, New England clam chowder | 1.427 |
| 28355210 | Crab soup, cream of, prepared with milk | 4.337 |  |  |
| 28355250 | Lobster bisque | 4.337 | Soup, bisque | 4.337 |
| 28355260 | Lobster gumbo | 7.660 | Lobster gumbo | 7.660 |
| 28355350 | Salmon soup, cream style | 4.337 |  |  |
| 28355440 | Shrimp gumbo | 7.660 | Shrimp gumbo | 7.660 |
| 28355450 | Seafood soup with potatoes and vegetables including carrots, broccoli, and/or dark-green leafy | 4.651 |  |  |
| 28355460 | Seafood soup with potatoes, and vegetables excluding carrots, broccoli, and dark-green leafy | 4.766 |  |  |
| 28355470 | Seafood soup with vegetables including carrots, broccoli, and/or dark-green leafy; no potatoes | 4.946 |  |  |
| 28355480 | Seafood soup with vegetables excluding carrots, broccoli, and dark-green leafy; no potatoes | 5.077 | Soup, fish or shrimp | 5.077 |
| 28360210 | Spanish vegetable soup, Puerto Rican style | 5.938 |  |  |
| 28500070 | Gravy, beef or meat, home recipe | 1.368 |  |  |
| 28500080 | Gravy, poultry, home recipe | 1.368 |  |  |
| 28510010 | Gravy or sauce, poultry-based from Puerto Rican-style chicken fricasse | 13.020 |  |  |
| 28510030 | Gravy, meat-based, from Puerto-Rican style beef stew | 3.113 |  |  |
| 28522000 | Mole poblano sauce | 14.844 | Mole sauce | 14.844 |
| 28522050 | Mole verde sauce | 6.457 |  |  |
| 32101500 | Egg, Benedict | 7.919 | Egg, Benedict | 7.919 |
| 32101530 | Egg curry | 5.888 |  |  |
| 32105190 | Egg casserole with bread, cheese, milk and meat | 2.184 | Egg casserole with bread, cheese, milk and meat | 2.184 |
| 32105210 | Chicken egg foo yung | 7.400 | Chicken egg foo yung | 7.400 |
| 32105220 | Pork egg foo yung | 7.409 | Pork egg foo yung | 7.409 |
| 32105230 | Shrimp egg foo yung | 7.436 | Shrimp egg foo yung | 7.436 |
| 32105240 | Beef egg foo yung | 7.400 | Beef egg foo yung | 7.400 |
| 32105330 | Scrambled eggs with jerked beef, Puerto Rican style | 11.560 |  |  |
| 32110150 | Shrimp-egg patty | 3.374 |  |  |
| 32130430 | Egg omelet or scrambled egg, with dark-green vegetables, fat added in cooking | 1.032 | Egg omelet or scrambled egg, with dark-green vegetables, fat added | 1.032 |
| 32130440 | Egg omelet or scrambled egg, with dark-green vegetables, fat not added in cooking | 1.076 | Egg omelet or scrambled egg, with dark-green vegetables, no added fat | 1.076 |
| 32130450 | Egg omelet or scrambled egg, with dark-green vegetables, NS as to fat added in cooking | 1.032 | Egg omelet or scrambled egg, with dark-green vegetables, NS as to fat | 1.032 |
| 32130460 | Egg omelet or scrambled egg, with tomatoes and dark-green vegetables, fat added in cooking | 0.612 | Egg omelet or scrambled egg, with tomatoes and dark-green vegetables, fat added | 0.612 |
| 32130470 | Egg omelet or scrambled egg, with tomatoes and dark-green vegetables, fat not added in cooking | 0.635 | Egg omelet or scrambled egg, with tomatoes and dark-green vegetables, no fat added | 0.635 |
| 32130480 | Egg omelet or scrambled egg, with tomatoes and dark-green vegetables, NS as to fat added in cooking | 0.612 | Egg omelet or scrambled egg, with tomatoes and dark-green vegetables, NS as to fat | 0.612 |
| 32130490 | Egg omelet or scrambled egg, with vegetables other than dark green and/or tomatoes, fat added in cooking | 13.143 | Egg omelet or scrambled egg, with vegetables other than dark green and/or tomatoes, fat added | 13.143 |
| 32130500 | Egg omelet or scrambled egg, with vegetables other than dark green and/or tomatoes, fat not added in cooking | 13.926 | Egg omelet or scrambled egg, with vegetables other than dark green and/or tomatoes, no added fat | 13.926 |
| 32130510 | Egg omelet or scrambled egg, with vegetables other than dark green and/or tomatoes, NS as to fat added in cooking | 13.143 | Egg omelet or scrambled egg, with vegetables other than dark green and/or tomatoes, NS as to fat | 13.143 |
| 32130630 | Egg omelet or scrambled egg, with cheese and dark-green vegetables, fat added in cooking | 0.933 | Egg omelet or scrambled egg, with cheese and dark-green vegetables, fat added | 0.933 |
| 32130640 | Egg omelet or scrambled egg, with cheese and dark-green vegetables, fat not added in cooking | 0.969 | Egg omelet or scrambled egg, with cheese and dark-green vegetables, no added fat | 0.969 |
| 32130650 | Egg omelet or scrambled egg, with cheese and dark-green vegetables, NS as to fat added in cooking | 0.933 | Egg omelet or scrambled egg, with cheese and dark-green vegetables, NS as to fat | 0.933 |
| 32130660 | Egg omelet or scrambled egg, with cheese, tomatoes, and dark-green vegetables, fat added in cooking | 0.560 | Egg omelet or scrambled egg, with cheese, tomatoes, and dark-green vegetables, fat added | 0.560 |
| 32130670 | Egg omelet or scrambled egg, with cheese, tomatoes, and dark-green vegetables, fat not added in cooking | 0.579 | Egg omelet or scrambled egg, with cheese, tomatoes, and dark-green vegetables, no added fat | 0.579 |
| 32130680 | Egg omelet or scrambled egg, with cheese, tomatoes, and dark-green vegetables, NS as to fat added in cooking | 0.560 | Egg omelet or scrambled egg, with cheese, tomatoes, and dark-green vegetables, NS as to fat | 0.560 |
| 32130690 | Egg omelet or scrambled egg, with cheese and vegetables other than dark green and/or tomatoes, fat added in cooking | 12.085 | Egg omelet or scrambled egg, with cheese and vegetables other than dark green and/or tomatoes, fat added | 12.085 |
| 32130700 | Egg omelet or scrambled egg, with cheese and vegetables other than dark green and/or tomatoes, fat not added in cooking | 12.745 | Egg omelet or scrambled egg, with cheese and vegetables other than dark green and/or tomatoes, no added fat | 12.745 |
| 32130710 | Egg omelet or scrambled egg, with cheese and vegetables other than dark green and/or tomatoes, NS as to fat added in cooking | 12.085 | Egg omelet or scrambled egg, with cheese and vegetables other than dark green and/or tomatoes, NS as to fat | 12.085 |
| 32130830 | Egg omelet or scrambled egg, with meat and dark-green vegetables, fat added in cooking | 0.607 | Egg omelet or scrambled egg, with meat and dark-green vegetables, fat added | 0.607 |
| 32130840 | Egg omelet or scrambled egg, with meat and dark-green vegetables, fat not added in cooking | 0.630 | Egg omelet or scrambled egg, with meat and dark-green vegetables, no added fat | 0.630 |
| 32130850 | Egg omelet or scrambled egg, with meat and dark-green vegetables, NS as to fat added in cooking | 0.607 | Egg omelet or scrambled egg, with meat and dark-green vegetables, NS as to fat | 0.607 |
| 32130860 | Egg omelet or scrambled egg, with meat, tomatoes, and dark-green vegetables, fat added in cooking | 0.215 | Egg omelet or scrambled egg, with meat, tomatoes, and dark-green vegetables, fat added | 0.215 |
| 32130870 | Egg omelet or scrambled egg, with meat, tomatoes, and dark-green vegetables, fat not added in cooking | 0.223 | Egg omelet or scrambled egg, with meat, tomatoes, and dark-green vegetables, no added fat | 0.223 |
| 32130880 | Egg omelet or scrambled egg, with meat, tomatoes, and dark-green vegetables, NS as to fat added in cooking | 0.215 | Egg omelet or scrambled egg, with meat, tomatoes, and dark-green vegetables, NS as to fat | 0.215 |
| 32130890 | Egg omelet or scrambled egg, with meat and vegetables other than dark-green and/or tomatoes, fat added in cooking | 5.137 | Egg omelet or scrambled egg, with meat and vegetables other than dark-green and/or tomatoes, fat added | 5.137 |
| 32130900 | Egg omelet or scrambled egg, with meat and vegetables other than dark-green and/or tomatoes, fat not added in cooking | 5.436 | Egg omelet or scrambled egg, with meat and vegetables other than dark-green and/or tomatoes, no added fat | 5.436 |
| 32130910 | Egg omelet or scrambled egg, with meat and vegetables other than dark-green and/or tomatoes, NS as to fat added in cooking | 5.137 | Egg omelet or scrambled egg, with meat and vegetables other than dark-green and/or tomatoes, NS as to fat | 5.137 |
| 32131030 | Egg omelet or scrambled egg, with cheese, meat, and dark-green vegetables, fat added in cooking | 0.211 | Egg omelet or scrambled egg, with cheese, meat, and dark-green vegetables, fat added | 0.211 |
| 32131040 | Egg omelet or scrambled egg, with cheese, meat, and dark-green vegetables, fat not added in cooking | 0.219 | Egg omelet or scrambled egg, with cheese, meat, and dark-green vegetables, no added fat | 0.219 |
| 32131050 | Egg omelet or scrambled egg, with cheese, meat, and dark-green vegetables, NS as to fat added in cooking | 0.211 | Egg omelet or scrambled egg, with cheese, meat, and dark-green vegetables, NS as to fat | 0.211 |
| 32131060 | Egg omelet or scrambled egg, with cheese, meat, tomatoes, and dark-green vegetables, fat added in cooking | 0.200 | Egg omelet or scrambled egg, with cheese, meat, tomatoes, and dark-green vegetables, fat added | 0.200 |
| 32131070 | Egg omelet or scrambled egg, with cheese, meat, tomatoes, and dark-green vegetables, fat not added in cooking | 0.207 | Egg omelet or scrambled egg, with cheese, meat, tomatoes, and dark-green vegetables, no added fat | 0.207 |
| 32131080 | Egg omelet or scrambled egg, with cheese, meat, tomatoes, and dark-green vegetables, NS as to fat added in cooking | 0.200 | Egg omelet or scrambled egg, with cheese, meat, tomatoes, and dark-green vegetables, NS as to fat | 0.200 |
| 32131090 | Egg omelet or scrambled egg, with cheese, meat, and vegetables other than dark-green and/or tomatoes, fat added in cooking | 4.732 | Egg omelet or scrambled egg, with cheese, meat, and vegetables other than dark-green and/or tomatoes, fat added | 4.732 |
| 32131100 | Egg omelet or scrambled egg, with cheese, meat, and vegetables other than dark-green and/or tomatoes, fat not added in cooking | 4.985 | Egg omelet or scrambled egg, with cheese, meat, and vegetables other than dark-green and/or tomatoes, no added fat | 4.985 |
| 32131110 | Egg omelet or scrambled egg, with cheese, meat, and vegetables other than dark-green and/or tomatoes, NS as to fat added in cooking | 4.732 | Egg omelet or scrambled egg, with cheese, meat, and vegetables other than dark-green and/or tomatoes, NS as to fat | 4.732 |
| 32131200 | Egg omelet or scrambled egg, with potatoes and/or onions, fat added in cooking | 9.597 | Egg omelet or scrambled egg, with potatoes and/or onions, fat added | 9.597 |
| 32131210 | Egg omelet or scrambled egg, with potatoes and/or onions, fat not added in cooking | 10.117 | Egg omelet or scrambled egg, with potatoes and/or onions, no added fat | 10.117 |
| 32131220 | Egg omelet or scrambled egg, with potatoes and/or onions, NS as to fat added in cooking | 9.597 | Egg omelet or scrambled egg, with potatoes and/or onions, NS as to fat | 9.597 |
| 32201000 | Fried egg sandwich | 26.353 |  |  |
| 32202000 | Egg, cheese, ham, and bacon on bun | 15.698 |  |  |
| 32202010 | Egg, cheese, and ham on English muffin | 19.200 |  |  |
| 32202020 | Egg, cheese, and ham on biscuit | 15.292 |  |  |
| 32202025 | Egg, cheese and ham on bagel | 16.118 |  |  |
| 32202030 | Egg, cheese, and sausage on English muffin | 19.200 |  |  |
| 32202034 | Egg, cheese, and sausage on bun | 14.824 |  |  |
| 32202035 | Egg, extra cheese, and extra sausage, on bun | 11.556 |  |  |
| 32202040 | Egg, cheese, and beef on English Muffin | 18.549 |  |  |
| 32202045 | Egg, cheese, and steak on bagel | 18.240 |  |  |
| 32202050 | Egg, cheese, and sausage on biscuit | 27.360 |  |  |
| 32202055 | Egg, cheese, and sausage griddle cake sandwich | 27.360 |  |  |
| 32202060 | Egg and sausage on biscuit | 17.280 |  |  |
| 32202070 | Egg, cheese, and bacon on biscuit | 27.360 |  |  |
| 32202075 | Egg, cheese, and bacon griddle cake sandwich | 27.360 |  |  |
| 32202080 | Egg, cheese, and bacon on English muffin | 19.200 |  |  |
| 32202085 | Egg, cheese and bacon on bagel | 18.119 |  |  |
| 32202090 | Egg and bacon on biscuit | 17.280 |  |  |
| 32202110 | Egg and ham on biscuit | 17.280 |  |  |
| 32202120 | Egg, cheese and sausage on bagel | 18.240 |  |  |
| 32203010 | Egg salad sandwich | 16.451 | Egg salad sandwich on white | 16.451 |
| 32203020 |  |  | Egg salad sandwich on wheat | 16.000 |
| 32204010 | Scrambled egg sandwich | 22.974 |  |  |
| 32301100 | Garlic egg soup, Puerto Rican style | 3.730 |  |  |
| 32400300 | Egg white, omelet, scrambled, or fried, with vegetables, fat added in cooking | 15.482 | Egg white, omelet, scrambled, or fried, with vegetables | 15.482 |
| 32400310 | Egg white, omelet, scrambled, or fried, with vegetables, fat not added in cooking | 16.581 |  |  |
| 32400320 | Egg white, omelet, scrambled, or fried, with vegetables, NS as to fat added in cooking | 15.482 |  |  |
| 32400500 | Egg white, omelet, scrambled, or fried, with cheese and vegetables, fat added in cooking | 14.036 | Egg white, omelet, scrambled, or fried, with cheese and vegetables | 14.036 |
| 32400510 | Egg white, omelet, scrambled, or fried, with cheese and vegetables, fat not added in cooking | 14.933 |  |  |
| 32400520 | Egg white, omelet, scrambled, or fried, with cheese and vegetables, NS as to fat added in cooking | 14.036 |  |  |
| 32400600 | Egg white, omelet, scrambled, or fried, with meat and vegetables, fat added in cooking | 6.027 | Egg white, omelet, scrambled, or fried, with meat and vegetables | 6.027 |
| 32400610 | Egg white, omelet, scrambled, or fried, with meat and vegetables, fat not added in cooking | 6.442 |  |  |
| 32400620 | Egg white, omelet, scrambled, or fried, with meat and vegetables, NS as to fat added in cooking | 6.027 |  |  |
| 32400700 | Egg white, omelet, scrambled, or fried, with cheese, meat, and vegetables, fat added in cooking | 5.478 | Egg white, omelet, scrambled, or fried, with cheese, meat, and vegetables | 5.478 |
| 32400710 | Egg white, omelet, scrambled, or fried, with cheese, meat, and vegetables, fat not added in cooking | 5.819 |  |  |
| 32400720 | Egg white, omelet, scrambled, or fried, with cheese, meat, and vegetables, NS as to fat added in cooking | 5.478 |  |  |
| 33401200 | Egg substitute, omelet, scrambled, or fried, with vegetables, fat added in cooking | 15.482 | Egg substitute, omelet, scrambled, or fried, with vegetables | 15.482 |
| 33401210 | Egg substitute, omelet, scrambled, or fried, with vegetables, fat not added in cooking | 16.581 |  |  |
| 33401220 | Egg substitute, omelet, scrambled, or fried, with vegetables, NS as to fat added in cooking | 15.482 |  |  |
| 33401400 | Egg substitute, omelet, scrambled, or fried, with cheese and vegetables, fat added in cooking | 14.036 | Egg substitute, omelet, scrambled, or fried, with cheese and vegetables | 14.036 |
| 33401410 | Egg substitute, omelet, scrambled, or fried, with cheese and vegetables, fat not added in cooking | 14.933 |  |  |
| 33401420 | Egg substitute, omelet, scrambled, or fried, with cheese and vegetables, NS as to fat added in cooking | 14.036 |  |  |
| 33401500 | Egg substitute, omelet, scrambled, or fried, with meat and vegetables, fat added in cooking | 6.027 | Egg substitute, omelet, scrambled, or fried, with meat and vegetables | 6.027 |
| 33401510 | Egg substitute, omelet, scrambled, or fried, with meat and vegetables, fat not added in cooking | 6.442 |  |  |
| 33401520 | Egg substitute, omelet, scrambled, or fried, with meat and vegetables, NS as to fat added in cooking | 6.027 |  |  |
| 33401600 | Egg substitute, omelet, scrambled, or fried, with cheese, meat, and vegetables, fat added in cooking | 5.478 | Egg substitute, omelet, scrambled, or fried, with cheese, meat, and vegetables | 5.478 |
| 33401610 | Egg substitute, omelet, scrambled, or fried, with cheese, meat, and vegetables, fat not added in cooking | 5.819 |  |  |
| 33401620 | Egg substitute, omelet, scrambled, or fried, with cheese, meat, and vegetables, NS as to fat added in cooking | 5.478 |  |  |
| 34001200 |  |  | Egg sandwich on wheat bread | 25.043 |
| 34001210 |  |  | Egg sandwich on wheat bread, with cheese | 21.176 |
| 34001220 |  |  | Egg sandwich on wheat bread, with meat | 18.344 |
| 34001230 |  |  | Egg sandwich on wheat bread, with meat and cheese | 16.180 |
| 41102990 |  |  | Lima beans, NFS | 13.000 |
| 41103000 | Lima beans, dry, cooked, NS as to fat added in cooking | 12.433 |  |  |
| 41103010 | Lima beans, dry, cooked, fat added in cooking, NS as to type of fat | 12.433 | Lima beans, from dried | 12.433 |
| 41103011 | Lima beans, dry, cooked, made with oil | 12.433 |  |  |
| 41103012 | Lima beans, dry, cooked, made with animal fat or meat drippings | 12.483 |  |  |
| 41103013 | Lima beans, dry, cooked, made with margarine | 12.391 |  |  |
| 41103020 | Lima beans, dry, cooked, fat not added in cooking | 13.329 |  |  |
| 41105990 |  |  | Kidney beans, NFS | 19.500 |
| 41106000 | Red kidney beans, dry, cooked, NS as to fat added in cooking | 18.012 |  |  |
| 41106010 | Red kidney beans, dry, cooked, fat added in cooking, NS as to type of fat | 18.012 | Kidney beans, from dried, fat added | 18.012 |
| 41106011 | Red kidney beans, dry, cooked, made with oil | 18.012 |  |  |
| 41106012 | Red kidney beans, dry, cooked, made with animal fat or meat drippings | 18.088 |  |  |
| 41106013 | Red kidney beans, dry, cooked, made with margarine | 17.947 |  |  |
| 41106020 | Red kidney beans, dry, cooked, fat not added in cooking | 19.390 | Kidney beans, from dried, no added fat | 19.390 |
| 41106040 | Red kidney beans, canned, drained, fat added in cooking, NS as to type of fat | 18.130 | Kidney beans, from canned, fat added | 18.130 |
| 41106050 | Red kidney beans, canned, drained, made with oil | 18.130 |  |  |
| 41106080 | Red kidney beans, canned, drained, fat not added in cooking | 19.500 | Kidney beans, from canned, no added fat | 19.500 |
| 41106100 | Red kidney beans, canned, drained, low sodium, NS as to fat added in cooking | 18.128 |  |  |
| 41106110 | Red kidney beans, canned, drained, low sodium, fat added in cooking | 18.128 | Kidney beans, from canned, reduced sodium | 18.128 |
| 41106120 | Red kidney beans, canned, drained, low sodium, fat not added in cooking | 19.500 |  |  |
| 41106150 |  |  | Kidney beans, from fast food / restaurant | 17.568 |
| 41106170 |  |  | Kidney beans with meat | 16.770 |
| 41107000 | Soybeans, dry, cooked, fat not added in cooking | 7.854 |  |  |
| 41107010 | Soybeans, dry, cooked, fat added in cooking | 7.281 | Soybeans, cooked | 7.281 |
| 41107020 | Soybeans, dry, cooked, NS as to fat added in cooking | 7.281 |  |  |
| 41203030 | Black bean salad | 11.931 | Black bean salad | 11.931 |
| 41205010 | Refried beans, fat added in cooking, NS as to type of fat | 6.495 | Refried beans | 6.495 |
| 41205011 | Refried beans, made with oil | 6.495 |  |  |
| 41205012 | Refried beans, made with animal fat or meat drippings | 6.511 |  |  |
| 41205013 | Refried beans, made with margarine | 6.481 |  |  |
| 41205016 | Refried beans, NS as to fat added in cooking | 6.495 |  |  |
| 41205030 | Refried beans with meat | 6.209 | Refried beans with meat | 6.209 |
| 41205070 | Hummus, plain | 8.971 | Hummus, plain | 8.971 |
| 41205075 | Hummus, flavored | 8.799 | Hummus, flavored | 8.799 |
| 41205100 | Black bean sauce | 28.882 | Black bean sauce | 28.882 |
| 41209000 | Falafel | 18.753 | Falafel | 18.753 |
| 41210000 | Bean cake | 18.350 | Bean cake | 18.350 |
| 41210090 | Stewed beans with pork, tomatoes, and chili peppers, Mexican style | 1.687 |  |  |
| 41210100 | Stewed red beans, Puerto Rican style | 0.291 |  |  |
| 41210150 | Stewed pink beans with white potatoes and ham, Puerto Rican style | 0.292 |  |  |
| 41210160 | Stewed pink beans with pig's feet, Puerto Rican style | 0.698 |  |  |
| 41210170 | Stewed red beans with pig's feet, Puerto Rican style | 0.698 |  |  |
| 41210190 | Stewed red beans with pig's feet and potatoes, Puerto Rican style | 0.697 |  |  |
| 41210200 | Black beans, Cuban style | 5.362 |  |  |
| 41221020 | Chili with beans, without meat | 15.516 | Chili, vegetarian | 15.516 |
| 41300990 |  |  | Blackeyed peas, NFS | 50.400 |
| 41301000 | Cowpeas, dry, cooked, NS as to fat added in cooking | 46.727 |  |  |
| 41301010 | Cowpeas, dry, cooked, fat added in cooking | 46.727 | Blackeyed peas, from dried | 46.727 |
| 41301020 | Cowpeas, dry, cooked, fat not added in cooking | 50.407 |  |  |
| 41301990 |  |  | Chickpeas, NFS | 12.500 |
| 41302000 | Chickpeas, dry, cooked, NS as to fat added in cooking | 11.477 |  |  |
| 41302010 | Chickpeas, dry, cooked, fat added in cooking, NS as to type of fat | 11.477 | Chickpeas, from dried, fat added | 11.477 |
| 41302011 | Chickpeas, dry, cooked, made with oil | 11.477 |  |  |
| 41302012 | Chickpeas, dry, cooked, made with animal fat or meat drippings | 11.529 |  |  |
| 41302013 | Chickpeas, dry, cooked, made with margarine | 11.433 |  |  |
| 41302020 | Chickpeas, dry, cooked, fat not added in cooking | 12.424 | Chickpeas, from dried, no added fat | 12.424 |
| 41302030 | Chickpeas, canned, drained, NS as to fat added in cooking | 11.622 |  |  |
| 41302040 | Chickpeas, canned, drained, fat added in cooking, NS as to type of fat | 11.622 | Chickpeas, from canned, fat added | 11.622 |
| 41302050 | Chickpeas, canned, drained, made with oil | 11.622 |  |  |
| 41302080 | Chickpeas, canned, drained, fat not added in cooking | 12.479 | Chickpeas, from canned, no added fat | 12.479 |
| 41302100 | Chickpeas, canned, drained, low sodium, NS as to fat added in cooking | 11.620 |  |  |
| 41302110 | Chickpeas, canned, drained, low sodium, fat added in cooking | 11.620 | Chickpeas, from canned, reduced sodium | 11.620 |
| 41302120 | Chickpeas, canned, drained, low sodium, fat not added in cooking | 12.500 |  |  |
| 41303000 | Green or yellow split peas, dry, cooked, fat not added in cooking | 18.705 | Split peas, from dried, no added fat | 18.705 |
| 41303010 | Green or yellow split peas, dry, cooked, fat added in cooking, NS as to type of fat | 17.495 | Split peas, from dried, fat added | 17.495 |
| 41303011 | Green or yellow split peas, dry, cooked, made with oil | 17.495 |  |  |
| 41303012 | Green or yellow split peas, dry, cooked, made with animal fat or meat drippings | 17.562 |  |  |
| 41303013 | Green or yellow split peas, dry, cooked, made with margarine | 17.438 |  |  |
| 41303020 | Green or yellow split peas, dry, cooked, NS as to fat added in cooking | 17.495 |  |  |
| 41304030 | Peas, dry, cooked with pork | 14.556 |  |  |
| 41304130 | Cowpeas, dry, cooked with pork | 38.055 |  |  |
| 41306000 | Loaf, lentil | 11.888 |  |  |
| 41310150 | Stewed chickpeas, Puerto Rican style | 0.309 |  |  |
| 41310160 | Stewed chickpeas, with potatoes, Puerto Rican style | 0.647 |  |  |
| 41310900 |  |  | Bean chips | 4.191 |
| 41311020 | Sambar, vegetable stew | 3.968 | Sambar, vegetable stew | 3.968 |
| 41311030 |  |  | Lentil curry | 1.827 |
| 41410010 | Soy nuts | 7.900 | Soy nuts | 7.900 |
| 41420380 |  |  | Yogurt, soy | 0.948 |
| 41421020 | Soybean curd, breaded, fried | 4.571 | Soybean curd, breaded, fried | 4.571 |
| 41601040 | Lima bean soup, home recipe, canned or ready-to-serve | 9.820 |  |  |
| 41601070 |  |  | Soup, miso or tofu | 10.610 |
| 41601080 | Pinto bean soup, home recipe, canned or ready-to-serve | 5.036 |  |  |
| 41601090 | Bean soup, with macaroni, home recipe, canned, or ready-to-serve | 11.156 |  |  |
| 41601100 | Portuguese bean soup, home recipe, canned or ready-to-serve | 3.423 |  |  |
| 41601130 | Bean soup, mixed beans, home recipe, canned or ready-to-serve | 6.119 |  |  |
| 41601140 | Bean soup, home recipe | 5.170 |  |  |
| 41601180 | Bean and ham soup, home recipe | 4.406 | Soup, bean, with meat | 4.406 |
| 41602020 | Garbanzo bean or chickpea soup, home recipe, canned or ready-to-serve | 9.360 |  |  |
| 41602030 |  |  | Soup, split pea, with meat | 6.474 |
| 41602050 |  |  | Soup, split pea | 7.399 |
| 41603010 | Lentil soup, home recipe, canned, or ready-to-serve | 5.211 | Soup, lentil | 5.211 |
| 41811950 | Swiss steak, with gravy, meatless | 3.723 | Swiss steak, with gravy, meatless | 3.723 |
| 41812400 | Vegetarian pot pie | 7.946 | Pot pie, no meat | 7.946 |
| 41812450 | Vegetarian chili, made with meat substitute | 16.550 |  |  |
| 41812500 | Tofu and vegetables including carrots, broccoli, and/or dark-green leafy; no potatoes, with soy-based sauce | 15.868 | Tofu and vegetables including carrots, broccoli, and/or dark-green leafy; no potatoes, with soy-based sauce | 15.868 |
| 41812510 | Tofu and vegetables excluding carrots, broccoli, and dark-green leafy; no potatoes, with soy-based sauce | 15.350 | Tofu and vegetables excluding carrots, broccoli, and dark-green leafy; no potatoes, with soy-based sauce | 15.350 |
| 41812800 | Vegetarian stew | 2.143 | Vegetarian stew | 2.143 |
| 41812850 | Vegetarian stroganoff | 7.948 | Vegetarian stroganoff | 7.948 |
| 41901020 | Soyburger, meatless, with cheese on bun | 14.049 | Veggie burger, on bun, with cheese | 14.049 |
| 41901030 |  |  | Falafel sandwich | 7.472 |
| 42203200 |  |  | Soy nut butter | 4.716 |
| 42204100 | Brown nut gravy, meatless | 9.333 | Gravy, vegetarian | 9.333 |
| 42301010 | Peanut butter sandwich, NFS | 31.304 | Peanut butter sandwich, NFS | 31.304 |
| 42301015 | Peanut butter sandwich, with regular peanut butter, on white bread | 31.304 | Peanut butter sandwich, with regular peanut butter, on white bread | 31.304 |
| 42301020 | Peanut butter sandwich, with regular peanut butter, on wheat bread | 31.304 | Peanut butter sandwich, with regular peanut butter, on wheat bread | 31.304 |
| 42301025 | Peanut butter sandwich, with regular peanut butter, on whole wheat bread | 31.304 |  |  |
| 42301115 | Peanut butter sandwich, with reduced fat peanut butter, on white bread | 31.304 | Peanut butter sandwich, with reduced fat peanut butter, on white bread | 31.304 |
| 42301120 | Peanut butter sandwich, with reduced fat peanut butter, on wheat bread | 31.304 | Peanut butter sandwich, with reduced fat peanut butter, on wheat bread | 31.304 |
| 42301125 | Peanut butter sandwich, with reduced fat peanut butter, on whole wheat bread | 31.304 |  |  |
| 42302010 | Peanut butter and jelly sandwich, NFS | 25.714 | Peanut butter and jelly sandwich, NFS | 25.714 |
| 42302015 | Peanut butter and jelly sandwich, with regular peanut butter, regular jelly, on white bread | 25.714 | Peanut butter and jelly sandwich, with regular peanut butter, regular jelly, on white bread | 25.714 |
| 42302020 | Peanut butter and jelly sandwich, with regular peanut butter, regular jelly, on wheat bread | 25.714 | Peanut butter and jelly sandwich, with regular peanut butter, regular jelly, on wheat bread | 25.714 |
| 42302025 | Peanut butter and jelly sandwich, with regular peanut butter, regular jelly, on whole wheat bread | 25.714 |  |  |
| 42302055 | Peanut butter and jelly sandwich, with reduced fat peanut butter, regular jelly, on white bread | 25.714 | Peanut butter and jelly sandwich, with reduced fat peanut butter, on white bread | 25.714 |
| 42302060 | Peanut butter and jelly sandwich, with reduced fat peanut butter, regular jelly, on wheat bread | 25.714 | Peanut butter and jelly sandwich, with reduced fat peanut butter, on wheat bread | 25.714 |
| 42302065 | Peanut butter and jelly sandwich, with reduced fat peanut butter, regular jelly, on whole wheat bread | 25.714 |  |  |
| 42302105 | Peanut butter and jelly sandwich, with regular peanut butter, reduced sugar jelly, on white bread | 25.714 | Peanut butter and jelly sandwich, with regular peanut butter, reduced sugar jelly, on white bread | 25.714 |
| 42302110 | Peanut butter and jelly sandwich, with regular peanut butter, reduced sugar jelly, on wheat bread | 25.714 | Peanut butter and jelly sandwich, with regular peanut butter, reduced sugar jelly, on wheat bread | 25.714 |
| 42302115 | Peanut butter and jelly sandwich, with regular peanut butter, reduced sugar jelly, on whole wheat bread | 25.714 |  |  |
| 42302155 | Peanut butter and jelly sandwich, with reduced fat peanut butter, reduced sugar jelly, on white bread | 25.714 |  |  |
| 42302160 | Peanut butter and jelly sandwich, with reduced fat peanut butter, reduced sugar jelly, on wheat bread | 25.714 |  |  |
| 42302165 | Peanut butter and jelly sandwich, with reduced fat peanut butter, reduced sugar jelly, on whole wheat bread | 25.714 |  |  |
| 42303100 | Peanut butter and jelly sandwich, frozen commercial product without crusts | 25.714 | Peanut butter and jelly sandwich, frozen commercial product without crusts | 25.714 |
| 42304020 |  |  | Almond butter sandwich, on wheat bread | 31.304 |
| 42304040 |  |  | Almond butter and jelly sandwich, on wheat bread | 25.714 |
| 42305020 |  |  | Nutella sandwich on wheat bread | 31.304 |
| 51000100 | Bread, NS as to major flour | 48.000 | Bread, NS as to major flour | 48.000 |
| 51000110 | Bread, NS as to major flour, toasted | 48.000 | Bread, NS as to major flour, toasted | 48.000 |
| 51000180 | Bread, made from home recipe or purchased at a bakery, NS as to major flour | 48.000 | Bread, made from home recipe or purchased at a bakery, NS as to major flour | 48.000 |
| 51000190 | Bread, made from home recipe or purchased at a bakery, toasted, NS as to major flour | 48.000 | Bread, made from home recipe or purchased at a bakery, toasted, NS as to major flour | 48.000 |
| 51000200 | Roll, NS as to major flour | 48.000 | Roll, NS as to major flour | 48.000 |
| 51000300 | Roll, hard, NS as to major flour | 48.000 | Roll, hard, NS as to major flour | 48.000 |
| 51000400 | Roll, bran, NS as to type of bran | 48.000 | Roll, bran, NS as to type of bran | 48.000 |
| 51101000 | Bread, white | 48.000 | Bread, white | 48.000 |
| 51101010 | Bread, white, toasted | 48.000 | Bread, white, toasted | 48.000 |
| 51101050 | Bread, white, made from home recipe or purchased at a bakery | 28.591 | Bread, white, made from home recipe or purchased at a bakery | 28.591 |
| 51101060 | Bread, white, made from home recipe or purchased at a bakery, toasted | 48.000 | Bread, white, made from home recipe or purchased at a bakery, toasted | 48.000 |
| 51102010 | Bread, white with whole wheat swirl | 48.000 | Bread, white with whole wheat swirl | 48.000 |
| 51102020 | Bread, white with whole wheat swirl, toasted | 48.000 | Bread, white with whole wheat swirl, toasted | 48.000 |
| 51105010 | Bread, Cuban | 48.000 | Bread, Cuban | 48.000 |
| 51105040 | Bread, Cuban, toasted | 48.000 | Bread, Cuban, toasted | 48.000 |
| 51106010 | Bread, native, water, Puerto Rican style | 28.194 | Bread, native, water, Puerto Rican style | 28.194 |
| 51106020 | Bread, native, water, toasted, Puerto Rican style | 48.000 | Bread, native, water, toasted, Puerto Rican style | 48.000 |
| 51106200 | Bread, lard, Puerto Rican style | 27.264 | Bread, lard, Puerto Rican style | 27.264 |
| 51106210 | Bread, lard, toasted, Puerto Rican style | 27.264 | Bread, lard, toasted, Puerto Rican style | 27.264 |
| 51106300 | Bread, caressed, Puerto Rican style | 29.895 |  |  |
| 51106310 | Bread, caressed, toasted, Puerto Rican style | 48.000 |  |  |
| 51107010 | Bread, French or Vienna | 48.000 | Bread, French or Vienna | 48.000 |
| 51107040 | Bread, French or Vienna, toasted | 48.000 | Bread, French or Vienna, toasted | 48.000 |
| 51108010 | Focaccia, Italian flatbread, plain | 48.000 | Focaccia, Italian, plain | 48.000 |
| 51108100 | Naan, Indian flatbread | 48.000 | Bread, naan | 48.000 |
| 51109010 | Bread, Italian, Grecian, Armenian | 48.000 | Bread, Italian, Grecian, Armenian | 48.000 |
| 51109040 | Bread, Italian, Grecian, Armenian, toasted | 48.000 | Bread, Italian, Grecian, Armenian, toasted | 48.000 |
| 51109100 | Bread, pita | 48.000 | Bread, pita | 48.000 |
| 51109110 | Bread, pita, toasted | 48.000 |  |  |
| 51109150 | Bread, pita with fruit | 40.873 | Bread, pita with fruit | 40.873 |
| 51109200 | Bread, pita with fruit, toasted | 40.800 |  |  |
| 51111010 | Bread, cheese | 48.000 | Bread, cheese | 48.000 |
| 51111040 | Bread, cheese, toasted | 48.000 | Bread, cheese, toasted | 48.000 |
| 51113010 | Bread, cinnamon | 48.000 | Bread, cinnamon | 48.000 |
| 51113100 | Bread, cinnamon, toasted | 48.000 | Bread, cinnamon, toasted | 48.000 |
| 51115010 | Bread, cornmeal and molasses | 22.219 | Bread, cornmeal and molasses | 22.219 |
| 51119010 | Bread, egg, Challah | 48.000 | Bread, egg, Challah | 48.000 |
| 51119040 | Bread, egg, Challah, toasted | 48.000 | Bread, egg, Challah, toasted | 48.000 |
| 51121015 | Garlic bread, NFS | 48.000 | Garlic bread, NFS | 48.000 |
| 51121025 | Garlic bread, from fast food / restaurant | 47.904 | Garlic bread, from fast food / restaurant | 47.904 |
| 51121035 | Garlic bread, from frozen | 48.000 | Garlic bread, from frozen | 48.000 |
| 51121045 | Garlic bread, with parmesan cheese, from fast food / restaurant | 46.967 | Garlic bread, with parmesan cheese, from fast food / restaurant | 46.967 |
| 51121055 | Garlic bread, with parmesan cheese, from frozen | 47.059 | Garlic bread, with parmesan cheese, from frozen | 47.059 |
| 51121065 | Garlic bread, with melted cheese, from fast food / restaurant | 38.339 | Garlic bread, with melted cheese, from fast food / restaurant | 38.339 |
| 51121075 | Garlic bread, with melted cheese, from frozen | 41.739 | Garlic bread, with melted cheese, from frozen | 41.739 |
| 51121110 | Bread, onion | 40.074 | Bread, onion | 40.074 |
| 51121120 | Bread, onion, toasted | 48.000 | Bread, onion, toasted | 48.000 |
| 51122000 | Bread, reduced calorie and/or high fiber, white or NFS | 48.000 | Bread, reduced calorie and/or high fiber, white or NFS | 48.000 |
| 51122010 | Bread, reduced calorie and/or high fiber, white or NFS, toasted | 48.000 | Bread, reduced calorie and/or high fiber, white or NFS, toasted | 48.000 |
| 51122100 | Bread, reduced calorie and/or high fiber, white or NFS, with fruit and/or nuts | 40.873 | Bread, reduced calorie and/or high fiber, white or NFS, with fruit and/or nuts | 40.873 |
| 51122110 | Bread, reduced calorie and/or high fiber, white or NFS, with fruit and/or nuts, toasted | 48.000 | Bread, reduced calorie and/or high fiber, white or NFS, with fruit and/or nuts, toasted | 48.000 |
| 51122300 | Bread, white, special formula, added fiber | 48.000 | Bread, white, special formula, added fiber | 48.000 |
| 51122310 | Bread, white, special formula, added fiber, toasted | 48.000 | Bread, white, special formula, added fiber, toasted | 48.000 |
| 51123010 | Bread, high protein | 48.000 |  |  |
| 51123020 | Bread, high protein, toasted | 48.000 | Bread, high protein, toasted | 48.000 |
| 51127010 | Bread, potato | 48.000 | Bread, potato | 48.000 |
| 51127020 | Bread, potato, toasted | 48.000 | Bread, potato, toasted | 48.000 |
| 51129010 | Bread, raisin | 43.200 | Bread, raisin | 43.200 |
| 51129020 | Bread, raisin, toasted | 43.200 | Bread, raisin, toasted | 43.200 |
| 51130510 | Bread, white, low sodium or no salt | 48.000 |  |  |
| 51130520 | Bread, white, low sodium or no salt, toasted | 48.000 |  |  |
| 51133010 | Bread, sour dough | 48.000 | Bread, sour dough | 48.000 |
| 51133020 | Bread, sour dough, toasted | 48.000 | Bread, sour dough, toasted | 48.000 |
| 51134000 | Bread, sweet potato | 23.486 | Bread, sweet potato | 23.486 |
| 51135000 | Bread, vegetable | 33.840 | Bread, vegetable | 33.840 |
| 51135010 | Bread, vegetable, toasted | 48.000 | Bread, vegetable, toasted | 48.000 |
| 51136000 | Bruschetta | 18.531 | Bruschetta | 18.531 |
| 51140100 | Bread, dough, fried | 24.569 | Bread, dough, fried | 24.569 |
| 51150000 | Roll, white, soft | 48.000 | Roll, white, soft | 48.000 |
| 51153000 | Roll, white, hard | 48.000 | Roll, white, hard | 48.000 |
| 51154010 | Roll, white, hot dog bun | 48.000 | Roll, white, hot dog bun | 48.000 |
| 51154100 | Roll, white, hamburger bun | 48.000 | Roll, white, hamburger bun | 48.000 |
| 51154510 | Roll, diet | 48.000 | Roll, diet | 48.000 |
| 51154550 | Roll, egg bread | 48.000 | Roll, egg bread | 48.000 |
| 51154600 | Roll, cheese | 45.600 | Roll, cheese | 45.600 |
| 51155000 | Roll, French or Vienna | 48.000 | Roll, French or Vienna | 48.000 |
| 51156500 | Roll, garlic | 48.719 | Roll, garlic | 48.719 |
| 51157000 | Roll, white, hoagie, submarine | 48.000 | Roll, white, hoagie, submarine | 48.000 |
| 51158100 | Roll, Mexican, bolillo | 48.000 | Roll, Mexican, bolillo | 48.000 |
| 51159000 | Roll, sour dough | 48.000 | Roll, sour dough | 48.000 |
| 51160000 | Roll, sweet, no frosting | 48.000 | Roll, sweet, no frosting | 48.000 |
| 51160100 | Roll, sweet, cinnamon bun, no frosting | 48.000 | Roll, sweet, cinnamon bun, no frosting | 48.000 |
| 51160110 | Roll, sweet, cinnamon bun, frosted | 48.000 | Roll, sweet, cinnamon bun, frosted | 48.000 |
| 51161000 | Pan Dulce, with fruit, no frosting | 48.000 | Pan Dulce, with fruit, no frosting | 48.000 |
| 51161020 | Roll, sweet, with fruit, frosted | 40.500 | Roll, sweet, with fruit, frosted | 40.500 |
| 51161030 | Roll, sweet, with fruit, frosted, diet | 48.000 | Roll, sweet, with fruit, frosted, diet | 48.000 |
| 51161050 | Roll, sweet, frosted | 37.816 | Roll, sweet, frosted | 37.816 |
| 51161250 | Pan Dulce, no topping | 48.000 | Pan Dulce, no topping | 48.000 |
| 51161270 | Pan Dulce, with sugar topping | 44.378 | Pan Dulce, with sugar topping | 44.378 |
| 51161280 | Pan Dulce, with raisins and icing | 41.364 | Pan Dulce, with raisins and icing | 41.364 |
| 51165000 | Coffee cake, yeast type | 48.000 | Coffee cake, yeast type | 48.000 |
| 51167000 | Brioche | 17.723 | Brioche | 17.723 |
| 51168000 | Bread, Spanish coffee | 26.966 | Bread, Spanish coffee | 26.966 |
| 51180010 | Bagel | 48.000 | Bagel | 48.000 |
| 51180030 | Bagel, with raisins | 43.200 | Bagel, with raisins | 43.200 |
| 51180080 | Bagel, with fruit other than raisins | 43.700 | Bagel, with fruit other than raisins | 43.700 |
| 51182010 | Bread stuffing | 3.545 | Bread stuffing | 3.545 |
| 51182020 | Bread stuffing made with egg | 45.120 | Bread stuffing made with egg | 45.120 |
| 51183990 | Breadsticks, NFS | 48.000 | Breadsticks, NFS | 48.000 |
| 51184000 | Breadsticks, hard, NFS | 48.000 | Breadsticks, hard, NFS | 48.000 |
| 51184100 | Breadsticks, hard, reduced sodium | 42.672 | Breadsticks, hard, reduced sodium | 42.672 |
| 51184200 | Breadsticks, soft, NFS | 48.000 | Breadsticks, soft, NFS | 48.000 |
| 51184210 | Breadsticks, soft, from fast food / restaurant | 47.904 | Breadsticks, soft, fast food / restaurant | 47.904 |
| 51184220 | Breadsticks, soft, from frozen | 48.000 | Breadsticks, soft, frozen | 48.000 |
| 51184230 | Breadsticks, soft, with parmesan cheese, from fast food / restaurant | 46.967 | Breadsticks, soft, with parmesan cheese, fast food / restaurant | 46.967 |
| 51184240 | Breadsticks, soft, with parmesan cheese, from frozen | 48.000 |  |  |
| 51184250 | Breadsticks, soft, topped with melted cheese | 38.339 |  |  |
| 51184260 | Breadsticks, soft, stuffed with melted cheese | 38.339 | Breadsticks, soft, stuffed or topped with melted cheese | 38.339 |
| 51185000 | Croutons | 48.000 | Croutons | 48.000 |
| 51186010 | Muffin, English | 48.000 | Muffin, English | 48.000 |
| 51186100 | Muffin, English, with raisins | 43.200 | Muffin, English, with raisins | 43.200 |
| 51186130 | Muffin, English, cheese | 45.600 | Muffin, English, cheese | 45.600 |
| 51186160 | Muffin, English, with fruit other than raisins | 43.700 | Muffin, English, with fruit other than raisins | 43.700 |
| 51187000 | Melba toast | 48.000 | Melba toast | 48.000 |
| 51188100 | Pannetone | 20.724 | Pannetone | 20.724 |
| 51300050 | Bread, whole grain white | 48.000 | Bread, whole grain white | 48.000 |
| 51300060 | Bread, whole grain white, toasted | 48.000 | Bread, whole grain white, toasted | 48.000 |
| 51300100 | Bagel, whole grain white | 48.000 | Bagel, whole grain white | 48.000 |
| 51300110 | Bread, whole wheat | 48.000 | Bread, whole wheat | 48.000 |
| 51300120 | Bread, whole wheat, toasted | 48.000 | Bread, whole wheat, toasted | 48.000 |
| 51300140 | Bread, whole wheat, made from home recipe or purchased at bakery | 26.895 | Bread, whole wheat, made from home recipe or purchased at bakery | 26.895 |
| 51300150 | Bread, whole wheat, made from home recipe or purchased at bakery, toasted | 48.000 | Bread, whole wheat, made from home recipe or purchased at bakery, toasted | 48.000 |
| 51300175 | Bread, chappatti or roti, wheat | 48.000 | Bread, chappatti or roti | 48.000 |
| 51300180 | Bread, puri, wheat | 22.859 | Bread, puri | 22.859 |
| 51300185 | Bread, paratha, wheat | 48.000 | Bread, paratha | 48.000 |
| 51300210 | Bread, whole wheat, with raisins | 21.173 | Bread, whole wheat, with raisins | 21.173 |
| 51300220 | Bread, whole wheat, with raisins, toasted | 43.200 | Bread, whole wheat, with raisins, toasted | 43.200 |
| 51300300 | Bread, sprouted wheat | 48.000 | Bread, sprouted wheat | 48.000 |
| 51300310 | Bread, sprouted wheat, toasted | 48.000 | Bread, sprouted wheat, toasted | 48.000 |
| 51301010 | Bread, wheat or cracked wheat | 48.000 | Bread, wheat or cracked wheat | 48.000 |
| 51301020 | Bread, wheat or cracked wheat, toasted | 48.000 | Bread, wheat or cracked wheat, toasted | 48.000 |
| 51301040 | Bread, wheat or cracked wheat, made from home recipe or purchased at bakery | 27.956 | Bread, wheat or cracked wheat, made from home recipe or purchased at bakery | 27.956 |
| 51301050 | Bread, wheat or cracked wheat, made from home recipe or purchased at bakery, toasted | 48.000 | Bread, wheat or cracked wheat, made from home recipe or purchased at bakery, toasted | 48.000 |
| 51301120 | Bread, wheat or cracked wheat, with raisins | 22.246 | Bread, wheat or cracked wheat, with raisins | 22.246 |
| 51301130 | Bread, wheat or cracked wheat, with raisins, toasted | 43.200 | Bread, wheat or cracked wheat, with raisins, toasted | 43.200 |
| 51301510 | Bread, wheat or cracked wheat, reduced calorie and/or high fiber | 48.000 | Bread, wheat or cracked wheat, reduced calorie and/or high fiber | 48.000 |
| 51301520 | Bread, wheat or cracked wheat, reduced calorie and/or high fiber, toasted | 48.000 | Bread, wheat or cracked wheat, reduced calorie and/or high fiber, toasted | 48.000 |
| 51301540 |  |  | Bread, French or Vienna, whole wheat | 48.000 |
| 51301550 |  |  | Bread, French or Vienna, whole wheat, toasted | 52.747 |
| 51301600 | Bread, pita, whole wheat | 48.000 | Bread, pita, whole wheat | 48.000 |
| 51301610 | Bread, pita, whole wheat, toasted | 48.000 |  |  |
| 51301620 | Bread, pita, wheat or cracked wheat | 48.000 | Bread, pita, wheat or cracked wheat | 48.000 |
| 51301630 | Bread, pita, wheat or cracked wheat, toasted | 48.000 |  |  |
| 51301700 | Bagel, wheat | 48.000 | Bagel, wheat | 48.000 |
| 51301750 | Bagel, whole wheat | 48.000 | Bagel, whole wheat | 48.000 |
| 51301800 | Bagel, wheat, with raisins | 43.200 | Bagel, wheat, with raisins | 43.200 |
| 51301805 | Bagel, whole wheat, with raisins | 43.200 | Bagel, whole wheat, with raisins | 43.200 |
| 51301820 | Bagel, wheat, with fruit and nuts | 42.240 | Bagel, wheat, with fruit and nuts | 42.240 |
| 51301900 | Bagel, wheat bran | 48.000 | Bagel, wheat bran | 48.000 |
| 51302500 | Muffin, English, wheat bran | 48.000 | Muffin, English, wheat bran | 48.000 |
| 51302520 | Muffin, English, wheat bran, with raisins | 43.200 | Muffin, English, wheat bran, with raisins | 43.200 |
| 51303010 | Muffin, English, wheat or cracked wheat | 48.000 | Muffin, English, wheat or cracked wheat | 48.000 |
| 51303030 | Muffin, English, whole wheat | 48.000 | Muffin, English, whole wheat | 48.000 |
| 51303050 | Muffin, English, wheat or cracked wheat, with raisins | 43.636 | Muffin, English, wheat or cracked wheat, with raisins | 43.636 |
| 51303070 | Muffin, English, whole wheat, with raisins | 43.200 | Muffin, English, whole wheat, with raisins | 43.200 |
| 51303100 | Muffin, English, whole grain white | 48.000 | Muffin, English, whole grain white | 48.000 |
| 51306000 | Breadsticks, hard, whole wheat | 28.800 | Breadsticks, hard, whole wheat | 28.800 |
| 51320010 | Roll, wheat or cracked wheat | 48.000 | Roll, wheat or cracked wheat | 48.000 |
| 51320060 | Roll, wheat or cracked wheat, hot dog bun | 48.000 | Roll, wheat or cracked wheat, hot dog bun | 48.000 |
| 51320070 | Roll, wheat or cracked wheat, hamburger bun | 48.000 | Roll, wheat or cracked wheat, hamburger bun | 48.000 |
| 51320500 | Roll, whole wheat | 48.000 | Roll, whole wheat | 48.000 |
| 51320550 | Roll, whole wheat, hot dog bun | 48.000 | Roll, whole wheat, hot dog bun | 48.000 |
| 51320560 | Roll, whole wheat, hamburger bun | 48.000 | Roll, whole wheat, hamburger bun | 48.000 |
| 51320700 | Roll, whole grain white | 48.000 | Roll, whole grain white | 48.000 |
| 51320710 | Roll, whole grain white, hot dog bun | 48.000 | Roll, whole grain white, hot dog bun | 48.000 |
| 51320720 | Roll, whole grain white, hamburger bun | 48.000 | Roll, whole grain white, hamburger bun | 48.000 |
| 51401010 | Bread, rye | 48.000 | Bread, rye | 48.000 |
| 51401020 | Bread, rye, toasted | 48.000 | Bread, rye, toasted | 48.000 |
| 51401030 | Bread, marble rye and pumpernickel | 48.000 | Bread, marble rye and pumpernickel | 48.000 |
| 51401040 | Bread, marble rye and pumpernickel, toasted | 48.000 | Bread, marble rye and pumpernickel, toasted | 48.000 |
| 51401060 | Bread, rye, reduced calorie and/or high fiber | 48.000 |  |  |
| 51401070 | Bread, rye, reduced calorie and/or high fiber, toasted | 48.000 |  |  |
| 51401200 | Muffin, English, rye | 48.000 | Muffin, English, rye | 48.000 |
| 51404010 | Bread, pumpernickel | 48.000 | Bread, pumpernickel | 48.000 |
| 51404020 | Bread, pumpernickel, toasted | 48.000 | Bread, pumpernickel, toasted | 48.000 |
| 51404500 | Bagel, pumpernickel | 48.000 | Bagel, pumpernickel | 48.000 |
| 51404550 | Muffin, English, pumpernickel | 48.000 | Muffin, English, pumpernickel | 48.000 |
| 51407010 | Bread, black | 48.000 | Bread, black | 48.000 |
| 51407020 | Bread, black, toasted | 48.000 | Bread, black, toasted | 48.000 |
| 51420000 | Roll, rye | 48.000 | Roll, rye | 48.000 |
| 51421000 | Roll, pumpernickel | 48.000 | Roll, pumpernickel | 48.000 |
| 51501010 | Bread, oatmeal | 48.000 | Bread, oatmeal | 48.000 |
| 51501020 | Bread, oatmeal, toasted | 48.000 | Bread, oatmeal, toasted | 48.000 |
| 51501040 | Bread, oat bran | 48.000 | Bread, oat bran | 48.000 |
| 51501050 | Bread, oat bran, toasted | 48.000 | Bread, oat bran, toasted | 48.000 |
| 51501060 | Bread, oat bran, reduced calorie and/or high fiber | 48.000 |  |  |
| 51501070 | Bread, oat bran, reduced calorie and/or high fiber, toasted | 48.000 |  |  |
| 51501080 | Bagel, oat bran | 48.000 | Bagel, oat bran | 48.000 |
| 51502010 | Roll, oatmeal | 48.000 | Roll, oatmeal | 48.000 |
| 51503000 | Muffin, English, oat bran | 48.000 | Muffin, English, oat bran | 48.000 |
| 51503040 | Muffin, English, oat bran, with raisins | 43.200 | Muffin, English, oat bran, with raisins | 43.200 |
| 51601010 | Bread, multigrain, toasted | 48.000 | Bread, multigrain, toasted | 48.000 |
| 51601020 | Bread, multigrain | 48.000 | Bread, multigrain | 48.000 |
| 51601210 | Bread, multigrain, with raisins | 43.200 | Bread, multigrain, with raisins | 43.200 |
| 51601220 | Bread, multigrain, with raisins, toasted | 48.000 | Bread, multigrain, with raisins, toasted | 48.000 |
| 51602010 | Bread, multigrain, reduced calorie and/or high fiber | 48.000 | Bread, multigrain, reduced calorie and/or high fiber | 48.000 |
| 51602020 | Bread, multigrain, reduced calorie and/or high fiber, toasted | 48.000 | Bread, multigrain, reduced calorie and/or high fiber, toasted | 48.000 |
| 51620000 | Roll, multigrain | 48.000 | Roll, multigrain | 48.000 |
| 51620020 | Roll, multigrain, hot dog bun | 48.000 | Roll, multigrain, hot dog bun | 48.000 |
| 51620030 | Roll, multigrain, hamburger bun | 48.000 | Roll, multigrain, hamburger bun | 48.000 |
| 51630000 | Bagel, multigrain | 48.000 | Bagel, multigrain | 48.000 |
| 51630100 | Bagel, multigrain, with raisins | 43.200 | Bagel, multigrain, with raisins | 43.200 |
| 51630200 | Muffin, English, multigrain | 48.000 | Muffin, English, multigrain | 48.000 |
| 51801010 | Bread, barley | 48.000 | Bread, barley | 48.000 |
| 51801020 | Bread, barley, toasted | 48.000 | Bread, barley, toasted | 48.000 |
| 51804010 | Bread, soy | 19.882 | Bread, soy | 19.882 |
| 51805010 | Bread, sunflower meal | 25.890 | Bread, sunflower meal | 25.890 |
| 51807000 | Injera, Ethiopian bread | 0.034 | Injera, Ethiopian bread | 0.034 |
| 52101040 | Crumpet | 15.365 |  |  |
| 52101100 | Biscuit, baking powder or buttermilk type, made from mix | 28.621 |  |  |
| 52102040 | Biscuit, baking powder or buttermilk type, made from refrigerated dough | 48.000 | Biscuit, from refrigerated dough | 48.000 |
| 52103000 | Biscuit, baking powder or buttermilk type, commercially baked | 48.000 | Biscuit, from fast food / restaurant | 48.000 |
| 52104010 | Biscuit, baking powder or buttermilk type, made from home recipe | 23.170 | Biscuit, home recipe | 23.170 |
| 52104040 | Biscuit, whole wheat | 24.053 | Biscuit, wheat | 24.053 |
| 52104200 | Biscuit, cinnamon-raisin | 17.228 | Biscuit with fruit | 17.228 |
| 52105100 | Scone | 22.493 | Scone | 22.493 |
| 52105200 | Scone, with fruit | 20.114 | Scone, with fruit | 20.114 |
| 52202060 | Cornbread, made from home recipe | 9.380 | Cornbread, made from home recipe | 9.380 |
| 52206060 | Cornbread muffin, stick, round, made from home recipe | 9.380 | Cornbread muffin, stick, round, made from home recipe | 9.380 |
| 52208760 | Gordita/sope shell, plain, no filling | 3.391 | Gordita/sope shell, plain, no filling | 3.391 |
| 52215000 | Tortilla, NFS | 24.000 | Tortilla, NFS | 24.000 |
| 52215200 | Tortilla, flour | 48.000 | Tortilla, flour | 48.000 |
| 52215260 | Tortilla, whole wheat | 48.000 | Tortilla, whole wheat | 48.000 |
| 52215350 | Taco shell, flour | 40.336 | Taco shell, flour | 40.336 |
| 52301000 | Muffin, NFS | 11.040 | Muffin, NFS | 11.040 |
| 52302010 | Muffin, fruit | 11.040 | Muffin, fruit | 11.040 |
| 52302020 | Muffin, fruit, low fat | 11.040 | Muffin, fruit, low fat | 11.040 |
| 52302500 | Muffin, chocolate chip | 10.855 | Muffin, chocolate chip | 10.855 |
| 52302600 | Muffin, chocolate | 12.070 | Muffin, chocolate | 12.070 |
| 52303010 | Muffin, whole wheat | 13.663 | Muffin, whole wheat | 13.663 |
| 52303500 | Muffin, wheat | 13.933 | Muffin, wheat | 13.933 |
| 52304000 | Muffin, whole grain | 13.933 | Muffin, whole grain | 13.933 |
| 52304010 | Muffin, wheat bran | 10.289 | Muffin, wheat bran | 10.289 |
| 52304040 | Muffin, bran with fruit, lowfat | 8.702 | Muffin, bran with fruit, lowfat | 8.702 |
| 52304100 | Muffin, oatmeal | 9.145 | Muffin, oatmeal | 9.145 |
| 52304150 |  |  | Muffin, oat bran | 48.000 |
| 52306010 | Muffin, plain | 12.800 | Muffin, plain | 12.800 |
| 52306300 | Muffin, cheese | 14.915 | Muffin, cheese | 14.915 |
| 52306500 | Muffin, pumpkin | 10.148 | Muffin, pumpkin | 10.148 |
| 52306550 | Muffin, zucchini | 10.314 | Muffin, zucchini | 10.314 |
| 52306700 | Muffin, carrot | 10.367 | Muffin, carrot | 10.367 |
| 52311010 | Popover | 12.379 | Popover | 12.379 |
| 52401000 | Bread, Boston Brown | 48.000 | Bread, Boston Brown | 48.000 |
| 52403000 | Bread, nut | 12.030 | Bread, nut | 12.030 |
| 52404060 | Bread, pumpkin | 10.148 | Bread, pumpkin | 10.148 |
| 52405010 | Bread, fruit | 11.825 | Bread, fruit | 11.825 |
| 52407000 | Bread, zucchini | 10.314 | Bread, zucchini | 10.314 |
| 52408000 | Bread, Irish soda | 15.103 | Bread, Irish soda | 15.103 |
| 53100070 | Cake batter, raw, not chocolate | 12.105 |  |  |
| 53100100 | Cake or cupcake, NS as to type | 24.000 | Cake or cupcake, NFS | 24.000 |
| 53101100 | Cake, angel food, without icing or filling | 24.000 | Cake, angel food | 24.000 |
| 53101200 | Cake, angel food, with icing or filling | 14.733 |  |  |
| 53101250 | Cake, angel food, with fruit and icing or filling | 13.688 |  |  |
| 53102100 | Cake or cupcake, applesauce, without icing or filling | 8.347 |  |  |
| 53102200 | Cake or cupcake, applesauce, with icing or filling | 5.446 | Cake or cupcake, apple | 5.446 |
| 53102600 | Cake or cupcake, banana, without icing or filling | 11.040 |  |  |
| 53102700 | Cake or cupcake, banana, with icing or filling | 7.123 | Cake or cupcake, banana | 7.123 |
| 53102800 | Cake or cupcake, Black Forest | 24.000 | Cake or cupcake, Black Forest | 24.000 |
| 53103000 | Cake, Boston cream pie | 24.000 | Cake, Boston cream pie | 24.000 |
| 53104100 | Cake or cupcake, carrot, without icing or filling | 8.663 |  |  |
| 53104260 | Cake or cupcake, carrot, with icing or filling | 5.579 | Cake or cupcake, carrot | 5.579 |
| 53104300 | Cake, carrot, diet | 24.000 |  |  |
| 53104400 | Cake or cupcake, coconut, with icing or filling | 24.000 | Cake or cupcake, coconut | 24.000 |
| 53104500 |  |  | Cheesecake, plain | 7.074 |
| 53105270 | Cake or cupcake, chocolate, devil's food or fudge, with icing or filling | 24.000 | Cake or cupcake, chocolate with chocolate icing, bakery | 24.000 |
| 53105300 | Cake or cupcake, German chocolate, with icing or filling | 22.072 | Cake or cupcake, German chocolate | 22.072 |
| 53105500 | Cake, chocolate, with icing, diet | 24.000 |  |  |
| 53106500 | Cake, cream, without icing or topping | 21.000 | Cake, cream | 21.000 |
| 53108200 | Snack cake, chocolate, with icing or filling | 24.000 | Snack cake, chocolate | 24.000 |
| 53108220 | Snack cake, chocolate, with icing or filling, reduced fat and calories | 24.000 |  |  |
| 53109200 | Snack cake, not chocolate, with icing or filling | 24.000 | Snack cake, white | 24.000 |
| 53109220 | Snack cake, not chocolate, with icing or filling, reduced fat and calories | 24.000 |  |  |
| 53109300 | Cake, Dobos Torte | 15.584 |  |  |
| 53110000 | Cake, fruit cake, light or dark, holiday type cake | 24.000 | Cake, fruit cake | 24.000 |
| 53111000 | Cake or cupcake, gingerbread | 13.027 | Cake or cupcake, gingerbread | 13.027 |
| 53112000 | Cake, ice cream and cake roll, chocolate | 14.008 |  |  |
| 53112100 | Cake, ice cream and cake roll, not chocolate | 14.008 | Ice cream cake | 14.008 |
| 53113000 | Cake, jelly roll | 18.667 | Cake, jelly roll | 18.667 |
| 53114100 | Cake or cupcake, lemon, with icing or filling | 16.594 | Cake or cupcake, lemon | 16.594 |
| 53115100 | Cake or cupcake, marble, without icing or filling | 24.000 |  |  |
| 53115200 | Cake or cupcake, marble, with icing or filling | 24.000 | Cake or cupcake, marble | 24.000 |
| 53115310 | Cake or cupcake, nut, without icing or filling | 21.000 |  |  |
| 53115320 | Cake or cupcake, nut, with icing or filling | 14.894 |  |  |
| 53115410 | Cake or cupcake, oatmeal | 7.486 |  |  |
| 53115450 | Cake or cupcake, peanut butter | 13.281 | Cake or cupcake, peanut butter | 13.281 |
| 53116000 | Cake, pound, without icing or filling | 24.000 | Cake, pound | 24.000 |
| 53116020 | Cake, pound, with icing or filling | 16.011 |  |  |
| 53116270 | Cake, pound, chocolate | 20.256 |  |  |
| 53116350 | Cake, pound, Puerto Rican style | 9.333 |  |  |
| 53116390 | Cake, pound, reduced fat, cholesterol free | 24.000 |  |  |
| 53116500 | Cake or cupcake, pumpkin, without icing or filling | 8.102 |  |  |
| 53116510 | Cake or cupcake, pumpkin, with icing or filling | 5.340 | Cake or cupcake, pumpkin | 5.340 |
| 53116550 | Cake or cupcake, raisin-nut | 17.920 |  |  |
| 53116570 | Cake, Ravani | 8.468 |  |  |
| 53116650 | Cake, Quezadilla, El Salvadorian style | 48.000 |  |  |
| 53117100 | Cake or cupcake, spice, without icing or filling | 8.347 |  |  |
| 53117200 | Cake or cupcake, spice, with icing or filling | 5.446 | Cake or cupcake, spice | 5.446 |
| 53118100 | Cake, sponge, without icing or filling | 24.000 | Cake, sponge | 24.000 |
| 53118200 | Cake, sponge, with icing or filling | 15.246 |  |  |
| 53118300 | Cake, sponge, chocolate | 15.246 |  |  |
| 53118410 | Rum cake, without icing | 24.000 |  |  |
| 53118500 | Cake, torte | 15.640 | Cake, torte | 15.640 |
| 53118550 | Cake, tres leche | 5.351 | Cake, tres leche | 5.351 |
| 53119000 | Cake, pineapple, upside down | 5.195 | Cake, pineapple, upside down | 5.195 |
| 53120270 | Cake or cupcake, white, with icing or filling | 24.000 | Cake or cupcake, white with white icing, bakery | 24.000 |
| 53121270 | Cake or cupcake, yellow, with icing or filling | 24.000 | Cake or cupcake, white with chocolate icing, bakery | 24.000 |
| 53122070 | Cake, shortcake, biscuit type, with whipped cream and fruit | 11.188 |  |  |
| 53122080 | Cake, shortcake, biscuit type, with fruit | 12.829 |  |  |
| 53123070 | Cake, shortcake, sponge type, with whipped cream and fruit | 15.303 | Cake, strawberry shortcake | 15.303 |
| 53123080 | Cake, shortcake, sponge type, with fruit | 16.944 |  |  |
| 53123500 | Cake, shortcake, with whipped topping and fruit, diet | 12.061 |  |  |
| 53124110 | Cake or cupcake, zucchini | 5.555 | Cake or cupcake, zucchini | 5.555 |
| 53200100 | Cookie, batter or dough, raw | 15.407 | Cookie, batter or dough, raw | 15.407 |
| 53201000 | Cookie, NFS | 15.360 | Cookie, NFS | 15.360 |
| 53202000 | Cookie, almond | 15.360 | Cookie, almond | 15.360 |
| 53203000 | Cookie, applesauce | 15.360 | Cookie, applesauce | 15.360 |
| 53203500 | Cookie, biscotti | 22.537 | Cookie, biscotti | 22.537 |
| 53204000 | Cookie, brownie, NS as to icing | 15.360 | Cookie, brownie, NS as to icing | 15.360 |
| 53204010 | Cookie, brownie, without icing | 15.360 | Cookie, brownie, without icing | 15.360 |
| 53204100 | Cookie, brownie, with icing or filling | 7.632 | Cookie, brownie, with icing or filling | 7.632 |
| 53204840 | Cookie, brownie, reduced fat, NS as to icing | 15.360 | Cookie, brownie, reduced fat, NS as to icing | 15.360 |
| 53204860 | Cookie, brownie, fat free, NS as to icing | 15.360 |  |  |
| 53205250 | Cookie, butterscotch, brownie | 15.360 | Cookie, butterscotch, brownie | 15.360 |
| 53205260 | Cookie, bar, with chocolate | 15.360 | Cookie, bar, with chocolate | 15.360 |
| 53206000 | Cookie, chocolate chip | 15.360 | Cookie, chocolate chip | 15.360 |
| 53206020 | Cookie, chocolate chip, made from home recipe or purchased at a bakery | 8.627 | Cookie, chocolate chip, made from home recipe or purchased at a bakery | 8.627 |
| 53206030 | Cookie, chocolate chip, reduced fat | 15.360 | Cookie, chocolate chip, reduced fat | 15.360 |
| 53206100 | Cookie, chocolate chip sandwich | 15.360 | Cookie, chocolate chip sandwich | 15.360 |
| 53206550 | Cookie, chocolate, made with oatmeal and coconut, no bake | 15.360 | Cookie, chocolate, made with oatmeal and coconut, no bake | 15.360 |
| 53207000 | Cookie, chocolate or fudge | 15.360 | Cookie, chocolate or fudge | 15.360 |
| 53207020 | Cookie, chocolate or fudge, reduced fat | 15.360 |  |  |
| 53207050 | Cookie, chocolate, with chocolate filling or coating, fat free | 15.360 |  |  |
| 53209005 | Cookie, chocolate, with icing or coating | 15.360 | Cookie, chocolate, with icing or coating | 15.360 |
| 53209010 | Cookie, sugar wafer, chocolate-covered | 15.360 | Cookie, sugar wafer, chocolate-covered | 15.360 |
| 53209015 | Cookie, chocolate sandwich | 15.360 | Cookie, chocolate sandwich | 15.360 |
| 53209020 | Cookie, chocolate sandwich, reduced fat | 15.360 | Cookie, chocolate sandwich, reduced fat | 15.360 |
| 53209100 | Cookie, chocolate, sandwich, with extra filling | 15.360 | Cookie, chocolate, sandwich, with extra filling | 15.360 |
| 53209500 | Cookie, chocolate and vanilla sandwich | 15.360 | Cookie, chocolate and vanilla sandwich | 15.360 |
| 53210000 | Cookie, chocolate wafer | 15.360 | Cookie, chocolate wafer | 15.360 |
| 53210900 | Cookie, graham cracker with chocolate and marshmallow | 4.978 | Cookie, graham cracker with chocolate and marshmallow | 4.978 |
| 53211000 | Cookie bar, with chocolate, nuts, and graham crackers | 15.360 | Cookie bar, with chocolate, nuts, and graham crackers | 15.360 |
| 53220000 | Cookie, fruit-filled bar | 15.360 | Cookie, fruit-filled bar | 15.360 |
| 53220010 | Cookie, fruit-filled bar, fat free | 15.360 |  |  |
| 53220030 | Cookie, fig bar | 15.360 | Cookie, fig bar | 15.360 |
| 53220040 | Cookie, fig bar, fat free | 15.360 |  |  |
| 53222010 | Cookie, fortune | 15.360 | Cookie, fortune | 15.360 |
| 53223000 | Cookie, gingersnaps | 15.360 | Cookie, gingersnaps | 15.360 |
| 53224000 | Cookie, ladyfinger | 15.360 | Cookie, ladyfinger | 15.360 |
| 53224250 | Cookie, lemon bar | 10.839 | Cookie, lemon bar | 10.839 |
| 53230000 | Cookie, molasses | 15.360 | Cookie, molasses | 15.360 |
| 53231000 | Cookie, Lebkuchen | 15.360 | Cookie, Lebkuchen | 15.360 |
| 53233000 | Cookie, oatmeal | 15.360 | Cookie, oatmeal | 15.360 |
| 53233010 | Cookie, oatmeal, with raisins | 15.360 | Cookie, oatmeal, with raisins | 15.360 |
| 53233040 | Cookie, oatmeal, reduced fat, NS as to raisins | 15.360 | Cookie, oatmeal, reduced fat, NS as to raisins | 15.360 |
| 53233050 | Cookie, oatmeal sandwich, with creme filling | 15.360 | Cookie, oatmeal sandwich, with creme filling | 15.360 |
| 53233060 | Cookie, oatmeal, with chocolate chips | 11.715 | Cookie, oatmeal, with chocolate chips | 11.715 |
| 53233080 | Cookie, oatmeal sandwich, with peanut butter and jelly filling | 15.360 | Cookie, oatmeal sandwich, with peanut butter and jelly filling | 15.360 |
| 53233100 | Cookie, oatmeal, with chocolate and peanut butter, no bake | 15.360 | Cookie, oatmeal, with chocolate and peanut butter, no bake | 15.360 |
| 53234000 | Cookie, peanut butter | 15.360 | Cookie, peanut butter | 15.360 |
| 53234100 | Cookie, peanut butter, with chocolate | 11.715 | Cookie, peanut butter, with chocolate | 11.715 |
| 53235000 | Cookie, peanut butter sandwich | 15.360 | Cookie, peanut butter sandwich | 15.360 |
| 53235500 | Cookie, with peanut butter filling, chocolate-coated | 15.360 | Cookie, with peanut butter filling, chocolate-coated | 15.360 |
| 53235600 | Cookie, Pfeffernusse | 15.360 | Cookie, Pfeffernusse | 15.360 |
| 53236100 | Cookie, pumpkin | 15.360 | Cookie, pumpkin | 15.360 |
| 53237000 | Cookie, raisin | 15.360 | Cookie, raisin | 15.360 |
| 53237010 | Cookie, raisin sandwich, cream-filled | 15.360 | Cookie, raisin sandwich, cream-filled | 15.360 |
| 53237500 | Cookie, rum ball, no bake | 15.360 | Cookie, rum ball, no bake | 15.360 |
| 53238000 | Cookie, sandwich-type, not chocolate or vanilla | 15.360 | Cookie, sandwich-type, not chocolate or vanilla | 15.360 |
| 53239000 | Cookie, shortbread | 15.360 | Cookie, shortbread | 15.360 |
| 53239010 | Cookie, shortbread, reduced fat | 15.360 | Cookie, shortbread, reduced fat | 15.360 |
| 53239050 | Cookie, shortbread, with icing or filling | 12.219 | Cookie, shortbread, with icing or filling | 12.219 |
| 53239100 | Pocky | 15.360 | Pocky | 15.360 |
| 53240000 | Cookie, animal | 15.360 | Cookie, animal | 15.360 |
| 53240010 | Cookie, animal, with frosting or icing | 15.360 | Cookie, animal, with frosting or icing | 15.360 |
| 53241500 | Cookie, butter or sugar | 15.360 | Cookie, butter or sugar | 15.360 |
| 53241510 | Marie biscuit | 15.360 | Marie biscuit | 15.360 |
| 53241600 | Cookie, butter or sugar, with fruit and/or nuts | 12.334 | Cookie, butter or sugar, with fruit and/or nuts | 12.334 |
| 53242000 | Cookie, sugar wafer | 15.360 | Cookie, sugar wafer | 15.360 |
| 53242500 | Cookie, toffee bar | 15.360 | Cookie, toffee bar | 15.360 |
| 53243000 | Cookie, vanilla sandwich | 15.360 | Cookie, vanilla sandwich | 15.360 |
| 53243010 | Cookie, vanilla sandwich, extra filling | 15.360 | Cookie, vanilla sandwich, extra filling | 15.360 |
| 53243050 | Cookie, vanilla sandwich, reduced fat | 15.360 | Cookie, vanilla sandwich, reduced fat | 15.360 |
| 53244020 | Cookie, butter or sugar, with icing or filling other than chocolate | 8.203 | Cookie, butter or sugar, with icing or filling other than chocolate | 8.203 |
| 53246000 | Cookie, tea, Japanese | 15.360 | Cookie, tea, Japanese | 15.360 |
| 53247000 | Cookie, vanilla wafer | 15.360 | Cookie, vanilla wafer | 15.360 |
| 53247050 | Cookie, vanilla wafer, reduced fat | 15.360 | Cookie, vanilla wafer, reduced fat | 15.360 |
| 53247500 | Cookie, vanilla with caramel, coconut, and chocolate coating | 15.360 | Cookie, vanilla with caramel, coconut, and chocolate coating | 15.360 |
| 53251100 | Cookie, rugelach | 15.360 | Cookie, rugelach | 15.360 |
| 53260030 | Cookie, chocolate chip, sugar free | 15.360 | Cookie, chocolate chip, reduced sugar | 15.360 |
| 53260200 | Cookie, oatmeal, sugar free | 15.360 | Cookie, oatmeal, reduced sugar | 15.360 |
| 53260300 | Cookie, sandwich, sugar free | 15.360 | Cookie, sandwich, reduced sugar | 15.360 |
| 53260400 | Cookie, sugar or plain, sugar free | 15.360 | Cookie, sugar or plain, sugar free | 15.360 |
| 53260500 | Cookie, sugar wafer, sugar free | 15.360 | Cookie, sugar wafer, sugar free | 15.360 |
| 53260600 | Cookie, peanut butter, sugar free | 15.360 | Cookie, peanut butter, sugar free | 15.360 |
| 53270100 | Cookies, Puerto Rican style | 26.345 | Cookies, Puerto Rican style | 26.345 |
| 53300100 | Pie, NFS | 10.560 | Pie, NFS | 10.560 |
| 53300170 | Pie, individual size or tart, NFS | 10.560 |  |  |
| 53300180 | Pie, fried, NFS | 10.560 |  |  |
| 53301000 | Pie, apple, two crust | 10.560 | Pie, apple | 10.560 |
| 53301070 | Pie, apple, individual size or tart | 10.560 | Pie, apple, fast food | 10.560 |
| 53301080 | Pie, apple, fried pie | 10.560 |  |  |
| 53301500 | Pie, apple, one crust | 6.927 |  |  |
| 53301750 | Pie, apple, diet | 8.430 |  |  |
| 53302000 | Pie, apricot, two crust | 10.426 |  |  |
| 53302070 | Pie, apricot, individual size or tart | 10.560 |  |  |
| 53302080 | Pie, apricot, fried pie | 10.560 |  |  |
| 53303000 | Pie, blackberry, two crust | 9.191 |  |  |
| 53303070 | Pie, blackberry, individual size or tart | 10.690 |  |  |
| 53303500 | Pie, berry, not blackberry, blueberry, boysenberry, huckleberry, raspberry, or strawberry; two crust | 10.260 | Pie, berry | 10.260 |
| 53303510 | Pie, berry, not blackberry, blueberry, boysenberry, huckleberry, raspberry, or strawberry; one crust | 6.145 |  |  |
| 53303570 | Pie, berry, not blackberry, blueberry, boysenberry, huckleberry, raspberry, or strawberry, individual size or tart | 11.838 |  |  |
| 53304000 | Pie, blueberry, two crust | 10.560 | Pie, blueberry | 10.560 |
| 53304050 | Pie, blueberry, one crust | 10.360 |  |  |
| 53304070 | Pie, blueberry, individual size or tart | 12.655 |  |  |
| 53305000 | Pie, cherry, two crust | 10.560 | Pie, cherry | 10.560 |
| 53305010 | Pie, cherry, one crust | 6.246 |  |  |
| 53305070 | Pie, cherry, individual size or tart | 10.560 |  |  |
| 53305080 | Pie, cherry, fried pie | 10.560 |  |  |
| 53305700 | Pie, lemon, not cream or meringue | 11.358 | Pie, lemon | 11.358 |
| 53305720 | Pie, lemon, not cream or meringue, individual size or tart | 13.034 |  |  |
| 53305750 | Pie, lemon, fried pie | 10.560 |  |  |
| 53306000 | Pie, mince, two crust | 10.560 |  |  |
| 53306070 | Pie, mince, individual size or tart | 12.740 |  |  |
| 53307000 | Pie, peach, two crust | 10.560 | Pie, peach | 10.560 |
| 53307050 | Pie, peach, one crust | 6.481 |  |  |
| 53307070 | Pie, peach, individual size or tart | 11.912 |  |  |
| 53307080 | Pie, peach, fried pie | 10.560 |  |  |
| 53307500 | Pie, pear, two crust | 12.123 |  |  |
| 53307570 | Pie, pear, individual size or tart | 13.487 |  |  |
| 53308000 | Pie, pineapple, two crust | 10.162 |  |  |
| 53308070 | Pie, pineapple, individual size or tart | 10.560 |  |  |
| 53308300 | Pie, plum, two crust | 10.350 |  |  |
| 53308500 | Pie, prune, one crust | 8.288 |  |  |
| 53309000 | Pie, raisin, two crust | 9.813 |  |  |
| 53309070 | Pie, raisin, individual size or tart | 11.386 |  |  |
| 53310000 | Pie, raspberry, one crust | 8.510 |  |  |
| 53310050 | Pie, raspberry, two crust | 11.877 |  |  |
| 53311000 | Pie, rhubarb, two crust | 12.939 |  |  |
| 53311050 | Pie, rhubarb, one crust | 8.496 |  |  |
| 53311070 | Pie, rhubarb, individual size or tart | 14.419 |  |  |
| 53312000 | Pie, strawberry, one crust | 7.681 | Pie, strawberry | 7.681 |
| 53313000 | Pie, strawberry-rhubarb, two crust | 12.013 |  |  |
| 53314000 | Pie, strawberry, individual size or tart | 10.089 |  |  |
| 53340000 | Pie, apple-sour cream | 5.439 |  |  |
| 53340500 | Pie, cherry, made with cream cheese and sour cream | 3.307 |  |  |
| 53341000 | Pie, banana cream | 10.560 | Pie, banana cream | 10.560 |
| 53341070 | Pie, banana cream, individual size or tart | 9.356 |  |  |
| 53341500 | Pie, buttermilk | 6.218 |  |  |
| 53341750 | Pie, chess | 11.347 |  |  |
| 53342000 | Pie, chocolate cream | 10.560 | Pie, chocolate cream | 10.560 |
| 53342070 | Pie, chocolate cream, individual size or tart | 9.416 |  |  |
| 53343000 | Pie, coconut cream | 10.560 | Pie, coconut cream | 10.560 |
| 53343070 | Pie, coconut cream, individual size or tart | 7.273 |  |  |
| 53344000 | Pie, custard | 10.560 | Pie, custard | 10.560 |
| 53344070 | Pie, custard, individual size or tart | 7.677 |  |  |
| 53344200 | Mixed fruit tart filled with custard or cream cheese | 6.824 |  |  |
| 53344300 | Dessert pizza | 12.899 | Dessert pizza | 12.899 |
| 53345000 | Pie, lemon cream | 4.714 |  |  |
| 53345070 | Pie, lemon cream, individual size or tart | 6.505 |  |  |
| 53346000 | Pie, peanut butter cream | 7.000 | Pie, peanut butter cream | 7.000 |
| 53346500 | Pie, pineapple cream | 6.231 | Pie, pineapple cream | 6.231 |
| 53347000 | Pie, pumpkin | 10.560 | Pie, pumpkin | 10.560 |
| 53347070 | Pie, pumpkin, individual size or tart | 10.560 |  |  |
| 53347100 | Pie, raspberry cream | 7.756 |  |  |
| 53347500 | Pie, sour cream, raisin | 9.552 |  |  |
| 53347600 | Pie, squash | 4.925 |  |  |
| 53348000 | Pie, strawberry cream | 3.792 |  |  |
| 53348070 | Pie, strawberry cream, individual size or tart | 6.663 |  |  |
| 53360000 | Pie, sweet potato | 6.254 | Pie, sweet potato | 6.254 |
| 53365000 | Pie, vanilla cream | 10.560 | Pie, vanilla cream | 10.560 |
| 53366000 | Pie, yogurt, frozen | 1.895 |  |  |
| 53370000 | Pie, chiffon, not chocolate | 7.027 |  |  |
| 53371000 | Pie, chiffon, chocolate | 6.790 |  |  |
| 53373000 | Pie, black bottom | 3.511 |  |  |
| 53381000 | Pie, lemon meringue | 10.560 | Pie, lemon meringue | 10.560 |
| 53381070 | Pie, lemon meringue, individual size or tart | 10.560 |  |  |
| 53382000 | Pie, chocolate-marshmallow | 2.173 |  |  |
| 53385000 | Pie, pecan | 10.560 | Pie, pecan | 10.560 |
| 53385070 | Pie, pecan, individual size or tart | 10.560 |  |  |
| 53385500 | Pie, oatmeal | 6.403 | Pie, oatmeal | 6.403 |
| 53386000 | Pie, pudding, flavors other than chocolate | 7.864 |  |  |
| 53386050 | Pie, pudding, flavors other than chocolate, individual size or tart | 26.857 |  |  |
| 53386250 | Pie, pudding, chocolate, with chocolate coating, individual size | 22.560 |  |  |
| 53386500 | Pie, pudding, flavors other than chocolate, with chocolate coating, individual size | 22.560 |  |  |
| 53387000 | Pie, Toll house chocolate chip | 8.409 |  |  |
| 53390000 | Pie, shoo-fly | 12.095 |  |  |
| 53390100 | Pie, tofu with fruit | 2.235 |  |  |
| 53391000 | Pie shell | 48.000 | Pie shell | 48.000 |
| 53391100 | Pie shell, graham cracker | 48.000 | Pie shell, graham cracker | 48.000 |
| 53391150 | Pie shell, chocolate wafer | 48.000 |  |  |
| 53391200 | Vanilla wafer dessert base | 48.000 |  |  |
| 53400200 | Blintz, cheese-filled | 5.226 |  |  |
| 53400300 | Blintz, fruit-filled | 3.436 |  |  |
| 53410100 | Cobbler, apple | 5.986 | Cobbler, apple | 5.986 |
| 53410200 | Cobbler, apricot | 5.381 |  |  |
| 53410300 | Cobbler, berry | 10.066 | Cobbler, berry | 10.066 |
| 53410500 | Cobbler, cherry | 6.016 | Cobbler, cherry | 6.016 |
| 53410800 | Cobbler, peach | 6.170 | Cobbler, peach | 6.170 |
| 53410850 | Cobbler, pear | 9.331 |  |  |
| 53410860 | Cobbler, pineapple | 5.923 |  |  |
| 53410880 | Cobbler, plum | 5.937 |  |  |
| 53410900 | Cobbler, rhubarb | 6.449 |  |  |
| 53415100 | Crisp, apple, apple dessert | 8.636 | Crisp, apple | 8.636 |
| 53415110 |  |  | Fritter, plain | 15.785 |
| 53415120 | Fritter, apple | 7.998 | Fritter, fruit | 7.998 |
| 53415200 | Fritter, banana | 10.357 |  |  |
| 53415220 | Fritter, berry | 8.922 |  |  |
| 53415300 | Crisp, blueberry | 8.688 | Crisp, berry | 8.688 |
| 53415400 | Crisp, cherry | 8.575 | Crisp, cherry | 8.575 |
| 53415500 | Crisp, peach | 4.407 | Crisp, peach | 4.407 |
| 53415600 | Crisp, rhubarb | 4.203 |  |  |
| 53420000 | Cream puff, eclair, custard or cream filled, NS as to icing | 5.133 | Cream puff, eclair, custard or cream filled, NS as to icing | 5.133 |
| 53420100 | Cream puff, eclair, custard or cream filled, not iced | 6.452 | Cream puff, eclair, custard or cream filled, not iced | 6.452 |
| 53420210 | Cream puff, eclair, custard or cream filled, iced, reduced fat | 4.486 | Cream puff, eclair, custard or cream filled, iced, reduced fat | 4.486 |
| 53420300 | Air filled fritter or fried puff, without syrup, Puerto Rican style | 6.598 |  |  |
| 53420310 | Wheat flour fritter, without syrup | 7.792 |  |  |
| 53420400 | Sopaipilla, without syrup or honey | 22.431 | Sopaipilla, without syrup or honey | 22.431 |
| 53420410 | Sopaipilla with syrup or honey | 18.632 | Sopaipilla with syrup or honey | 18.632 |
| 53430000 | Crepe, NS as to filling | 6.393 |  |  |
| 53430100 | Crepe, chocolate filled | 4.027 | Crepe, chocolate filled | 4.027 |
| 53430200 | Crepe, fruit filled | 6.397 | Crepe, fruit filled | 6.397 |
| 53430750 | Tamale, sweet, with fruit | 1.640 |  |  |
| 53440300 | Strudel, berry | 11.859 | Strudel, berry | 11.859 |
| 53440500 | Strudel, cherry | 8.000 | Strudel, cherry | 8.000 |
| 53440600 | Strudel, cheese | 8.319 | Strudel, cheese | 8.319 |
| 53440700 | Strudel, peach | 7.589 | Strudel, peach | 7.589 |
| 53440750 | Strudel, pineapple | 7.751 |  |  |
| 53440800 | Strudel, cheese and fruit | 4.587 |  |  |
| 53441110 | Baklava | 17.753 | Baklava | 17.753 |
| 53441210 | Basbousa | 7.052 | Basbousa | 7.052 |
| 53450000 | Turnover or dumpling, apple | 14.353 | Turnover, fruit | 14.353 |
| 53450300 | Turnover or dumpling, berry | 15.811 |  |  |
| 53450500 | Turnover or dumpling, cherry | 11.604 |  |  |
| 53450800 | Turnover or dumpling, lemon | 11.628 |  |  |
| 53451000 | Turnover or dumpling, peach | 14.374 |  |  |
| 53451500 | Turnover, guava | 10.932 |  |  |
| 53451750 | Turnover, pumpkin | 8.371 |  |  |
| 53452100 | Pastry, fruit-filled | 10.327 | Pastry, fruit-filled | 10.327 |
| 53452120 | Pastry, made with bean or lotus seed paste filling, baked | 15.731 | Pastry, made with bean or lotus seed paste filling, baked | 15.731 |
| 53452130 | Pastry, made with bean paste and salted egg yolk filling, baked | 13.122 | Pastry, made with bean paste and salted egg yolk filling, baked | 13.122 |
| 53452170 | Pastry, cookie type, fried | 11.144 | Pastry, cookie type, fried | 11.144 |
| 53452200 | Pastry, Italian, with cheese | 9.912 | Pastry, Italian, with cheese | 9.912 |
| 53452400 | Pastry, puff | 7.200 | Pastry, puff | 7.200 |
| 53452420 | Pastry, puff, custard or cream filled, iced or not iced | 4.032 | Pastry, puff, custard or cream filled, iced or not iced | 4.032 |
| 53452450 | Cheese pastry puffs | 6.978 | Cheese pastry puffs | 6.978 |
| 53452500 | Pastry, mainly flour and water, fried | 38.413 | Pastry, mainly flour and water, fried | 38.413 |
| 53453150 | Empanada, Mexican turnover, fruit-filled | 19.041 | Empanada, fruit | 19.041 |
| 53453170 | Empanada, Mexican turnover, pumpkin | 18.587 |  |  |
| 53500100 | Breakfast pastry, NFS | 7.200 | Breakfast pastry, NFS | 7.200 |
| 53510000 | Danish pastry, plain or spice | 7.200 | Danish pastry, plain or spice | 7.200 |
| 53510100 | Danish pastry, with fruit | 7.200 | Danish pastry, with fruit | 7.200 |
| 53511000 | Danish pastry, with cheese | 7.200 | Danish pastry, with cheese | 7.200 |
| 53520000 | Doughnut, NS as to cake or yeast | 48.000 | Doughnut, NFS | 48.000 |
| 53520110 | Doughnut, cake type | 48.000 |  |  |
| 53520120 | Doughnut, chocolate, cake type | 48.000 | Doughnut, chocolate | 48.000 |
| 53520140 | Doughnut, cake type, chocolate covered | 48.000 | Doughnut, cake type, chocolate icing | 48.000 |
| 53520150 | Doughnut, cake type, chocolate covered, dipped in peanuts | 36.443 |  |  |
| 53520160 | Doughnut, chocolate, cake type, with chocolate icing | 48.000 | Doughnut, chocolate, with chocolate icing | 48.000 |
| 53520200 | Churros | 9.723 | Churros | 9.723 |
| 53520500 | Doughnut, Asian | 48.000 |  |  |
| 53520510 |  |  | Beignet | 42.667 |
| 53520600 | Cruller, NFS | 48.000 |  |  |
| 53520700 | French cruller | 48.000 |  |  |
| 53521100 | Doughnut, chocolate, raised or yeast, with chocolate icing | 33.423 |  |  |
| 53521110 | Doughnut, raised or yeast | 48.000 | Doughnut, yeast type | 48.000 |
| 53521120 | Doughnut, chocolate, raised or yeast | 45.851 |  |  |
| 53521130 | Doughnut, raised or yeast, chocolate covered | 32.716 | Doughnut, yeast type, with chocolate icing | 32.716 |
| 53521140 | Doughnut, jelly | 48.000 | Doughnut, jelly | 48.000 |
| 53521210 | Doughnut, custard-filled | 48.000 | Doughnut, custard-filled | 48.000 |
| 53521220 | Doughnut, chocolate cream-filled | 37.354 |  |  |
| 53521230 | Doughnut, custard-filled, with icing | 28.478 | Doughnut, custard-filled, with icing | 28.478 |
| 53530000 |  |  | Breakfast tart | 5.000 |
| 53610100 | Coffee cake, crumb or quick-bread type | 13.145 | Coffee cake, crumb or quick-bread type | 13.145 |
| 53610170 | Coffee cake, crumb or quick-bread type, with fruit | 8.357 | Coffee cake, crumb or quick-bread type, with fruit | 8.357 |
| 53610200 | Coffee cake, crumb or quick-bread type, cheese-filled | 11.275 | Coffee cake, crumb or quick-bread type, cheese-filled | 11.275 |
| 53710700 |  |  | Cereal or granola bar (Kellogg's Special K bar) | 48.000 |
| 53710810 |  |  | Cereal or granola bar (KIND Fruit and Nut Bar) | 1.124 |
| 53713010 |  |  | Cereal or granola bar, fruit and nut | 1.124 |
| 53714510 | Breakfast bar, date, with yogurt coating | 15.360 | Breakfast bar, date, with yogurt coating | 15.360 |
| 54001000 | Crackers, NFS | 48.000 | Crackers, NFS | 48.000 |
| 54102010 | Graham crackers | 15.360 | Graham crackers | 15.360 |
| 54102015 | Graham crackers (Teddy Grahams) | 15.360 | Graham crackers (Teddy Grahams) | 15.360 |
| 54102020 | Graham crackers, chocolate covered | 15.360 | Graham crackers, chocolate covered | 15.360 |
| 54102050 | Crackers, oatmeal | 48.000 | Crackers, oatmeal | 48.000 |
| 54102060 | Crackers, Cuban | 15.360 | Crackers, Cuban | 15.360 |
| 54102100 | Graham crackers, reduced fat | 15.360 | Graham crackers, reduced fat | 15.360 |
| 54102200 | Graham crackers, sandwich, with filling | 15.360 | Graham crackers, sandwich, with filling | 15.360 |
| 54103000 | Crackers, breakfast biscuit | 25.248 | Crackers, breakfast biscuit | 25.248 |
| 54200100 | Crackers, butter, reduced sodium | 48.000 | Crackers, butter, reduced sodium | 48.000 |
| 54201010 | Crackers, matzo, reduced sodium | 48.000 | Crackers, matzo, reduced sodium | 48.000 |
| 54202020 | Crackers, saltine, reduced sodium | 47.442 | Crackers, saltine, reduced sodium | 47.442 |
| 54204020 | Crackers, wheat, reduced sodium | 48.000 | Crackers, wheat, reduced sodium | 48.000 |
| 54204030 | Crackers, woven wheat, reduced sodium | 48.000 | Crackers, woven wheat, reduced sodium | 48.000 |
| 54301010 | Crackers, butter, plain | 48.000 | Crackers, butter, plain | 48.000 |
| 54301020 | Crackers, butter, flavored | 48.000 | Crackers, butter, flavored | 48.000 |
| 54301030 | Crackers, butter (Ritz) | 48.000 | Crackers, butter (Ritz) | 48.000 |
| 54301100 | Crackers, butter, reduced fat | 48.000 | Crackers, butter, reduced fat | 48.000 |
| 54304000 | Crackers, cheese | 48.000 | Crackers, cheese | 48.000 |
| 54304005 | Crackers, cheese (Cheez-It) | 48.000 | Crackers, cheese (Cheez-It) | 48.000 |
| 54304020 | Crackers, cheese (Goldfish) | 48.000 | Crackers, cheese (Goldfish) | 48.000 |
| 54304100 | Crackers, cheese, reduced fat | 48.000 | Crackers, cheese, reduced fat | 48.000 |
| 54304150 | Crackers, cheese, whole grain | 48.000 | Crackers, cheese, whole grain | 48.000 |
| 54305010 | Crackers, crispbread | 14.000 | Crackers, crispbread | 14.000 |
| 54305020 | Crackers, flatbread | 7.000 | Crackers, flatbread | 7.000 |
| 54307000 | Crackers, matzo | 48.000 | Crackers, matzo | 48.000 |
| 54308000 | Crackers, milk | 48.000 | Crackers, milk | 48.000 |
| 54313000 | Crackers, oyster | 48.000 | Crackers, oyster | 48.000 |
| 54325000 | Crackers, saltine | 48.000 | Crackers, saltine | 48.000 |
| 54325010 | Crackers, saltine, reduced fat | 48.000 | Crackers, saltine, reduced fat | 48.000 |
| 54325060 | Crackers, saltine, multigrain | 48.000 | Crackers, saltine, multigrain | 48.000 |
| 54326000 | Crackers, multigrain | 48.000 | Crackers, multigrain | 48.000 |
| 54336000 | Crackers, water | 48.000 | Crackers, water | 48.000 |
| 54336100 | Crackers, wonton | 48.000 | Crackers, wonton | 48.000 |
| 54337010 | Crackers, woven wheat | 48.000 | Crackers, woven wheat | 48.000 |
| 54337020 | Crackers, woven wheat, plain (Triscuit) | 48.000 | Crackers, woven wheat, plain (Triscuit) | 48.000 |
| 54337030 | Crackers, woven wheat, flavored (Triscuit) | 48.000 | Crackers, woven wheat, flavored (Triscuit) | 48.000 |
| 54337060 | Crackers, woven wheat, reduced fat | 48.000 | Crackers, woven wheat, reduced fat | 48.000 |
| 54338000 | Crackers, wheat | 48.000 | Crackers, wheat | 48.000 |
| 54338010 | Crackers, wheat, plain (Wheat Thins) | 48.000 | Crackers, wheat, plain (Wheat Thins) | 48.000 |
| 54338020 | Crackers, wheat, flavored (Wheat Thins) | 48.000 | Crackers, wheat, flavored (Wheat Thins) | 48.000 |
| 54338100 | Crackers, wheat, reduced fat | 48.000 | Crackers, wheat, reduced fat | 48.000 |
| 54339000 | Crackers, corn | 48.000 |  |  |
| 54350000 | Crackers, baby food | 48.000 | Baby Toddler crackers | 48.000 |
| 54406010 |  |  | Onion flavored rings | 106.100 |
| 54406200 | Shrimp chips | 48.000 | Shrimp chips | 48.000 |
| 54408070 |  |  | Pretzels, hard, multigrain | 0.941 |
| 54408475 |  |  | Pretzels, soft, from school lunch | 2.286 |
| 54408480 |  |  | Pretzels, soft, multigrain | 2.271 |
| 55100005 | Pancakes, NFS | 15.360 | Pancakes, NFS | 15.360 |
| 55100010 | Pancakes, plain, from frozen | 15.360 | Pancakes, plain, frozen | 15.360 |
| 55100015 | Pancakes, plain, reduced fat, from fozen | 15.360 |  |  |
| 55100020 | Pancakes, with fruit, from frozen | 14.842 | Pancakes, fruit, frozen | 14.842 |
| 55100025 | Pancakes, with chocolate, from frozen | 14.592 | Pancakes, chocolate, frozen | 14.592 |
| 55100030 | Pancakes, whole grain, from frozen | 14.899 | Pancakes, whole grain, frozen | 14.899 |
| 55100035 | Pancakes, whole grain, reduced fat, from frozen | 15.360 |  |  |
| 55100050 | Pancakes, plain, from fast food / restaurant | 4.984 | Pancakes, plain, fast food / restaurant | 4.984 |
| 55100055 | Pancakes, with fruit, from fast food / restaurant | 4.987 | Pancakes, fruit, fast food / restaurant | 4.987 |
| 55100060 | Pancakes, with chocolate, from fast food / restaurant | 4.163 | Pancakes, chocolate, fast food / restaurant | 4.163 |
| 55100065 | Pancakes, whole grain, from fast food / restaurant | 5.138 | Pancakes, whole grain, fast food / restaurant | 5.138 |
| 55100070 | Pancakes, whole grain and nuts, from fast food / restaurant | 4.590 |  |  |
| 55100080 | Pancakes, from school, NFS | 13.824 | Pancakes, school | 13.824 |
| 55101000 | Pancakes, plain | 5.157 | Pancakes, plain | 5.157 |
| 55101015 | Pancakes, plain, reduced fat | 5.271 | Pancakes, plain, reduced fat | 5.271 |
| 55103000 | Pancakes, with fruit | 5.122 | Pancakes, fruit | 5.122 |
| 55103020 | Pancakes, pumpkin | 4.284 | Pancakes, pumpkin | 4.284 |
| 55103100 | Pancakes, with chocolate | 4.284 | Pancakes, chocolate | 4.284 |
| 55105100 | Pancakes, cornmeal | 4.509 |  |  |
| 55105200 | Pancakes, whole grain | 5.323 | Pancakes, whole grain | 5.323 |
| 55105205 | Pancakes, whole grain, reduced fat | 5.444 | Pancakes, whole grain, reduced fat | 5.444 |
| 55200010 | Waffle, NFS | 15.360 | Waffle, NFS | 15.360 |
| 55200020 | Waffle, plain, from frozen | 15.360 | Waffle, plain, frozen | 15.360 |
| 55200030 | Waffle, plain, reduced fat, from frozen | 18.771 | Waffle, plain, reduced fat | 18.771 |
| 55200040 | Waffle, fruit, from frozen | 14.842 | Waffle, fruit, frozen | 14.842 |
| 55200050 | Waffle, chocolate, from frozen | 14.592 | Waffle, chocolate, frozen | 14.592 |
| 55200060 |  |  | Waffle, whole grain, frozen | 45.600 |
| 55200070 |  |  | Waffle, whole grain, reduced fat | 48.000 |
| 55200080 | Waffle, whole grain, fruit, from frozen | 0.238 | Waffle, whole grain, fruit, frozen | 0.238 |
| 55200100 | Waffle, plain, from fast food / restaurant | 4.680 | Waffle, plain, fast food / restaurant | 4.680 |
| 55200110 | Waffle, chocolate, from fast food / restaurant | 3.949 | Waffle, chocolate, fast food / restaurant | 3.949 |
| 55200120 | Waffle, fruit, from fast food / restaurant | 4.746 | Waffle, fruit, fast food / restaurant | 4.746 |
| 55200130 | Waffle, whole grain, from fast food / restaurant | 4.816 | Waffle, whole grain, fast food / restaurant | 4.816 |
| 55200200 |  |  | Waffle, school | 48.000 |
| 55201000 | Waffle, plain | 4.989 | Waffle, plain | 4.989 |
| 55203000 | Waffle, fruit | 4.992 | Waffle, fruit | 4.992 |
| 55203600 | Waffle, chocolate | 4.167 | Waffle, chocolate | 4.167 |
| 55203700 | Waffle, cinnamon | 4.978 | Waffle, cinnamon | 4.978 |
| 55204000 | Waffle, cornmeal | 6.322 |  |  |
| 55205000 | Waffle, whole grain | 5.144 | Waffle, whole grain | 5.144 |
| 55211050 | Waffle, plain, reduced fat | 5.157 |  |  |
| 55212000 | Waffle, whole grain, reduced fat | 5.323 |  |  |
| 55300020 | French toast, plain, from frozen | 22.737 | French toast, frozen | 22.737 |
| 55300030 | French toast, whole grain, from frozen | 22.737 |  |  |
| 55300050 | French toast, plain, from fast food / restaurant | 23.607 | French toast, fast food / restaurant | 23.607 |
| 55300055 | French toast, whole grain, from fast food / restaurant | 23.607 |  |  |
| 55300060 | French toast, from school, NFS | 23.564 | French toast, school | 23.564 |
| 55301000 | French toast, plain | 20.426 | French toast, plain | 20.426 |
| 55301010 | French toast, plain, reduced fat | 21.493 |  |  |
| 55301015 | French toast, whole grain | 20.426 | French toast, whole grain | 20.426 |
| 55301020 | French toast, whole grain, reduced fat | 21.493 |  |  |
| 55301031 | French toast sticks, plain, from frozen | 27.613 | French toast sticks | 27.613 |
| 55301040 | French toast sticks, plain, from fast food / restaurant | 48.000 | French toast sticks, fast food / restaurant | 48.000 |
| 55301048 | French toast sticks, from school, NFS | 23.564 | French toast sticks, school | 23.564 |
| 55301050 | French toast sticks, plain | 48.000 |  |  |
| 55310100 | Fried bread, Puerto Rican style | 8.111 |  |  |
| 55401000 | Crepe, plain | 8.622 | Crepe, plain | 8.622 |
| 55502000 | Flour and water gravy | 9.992 |  |  |
| 55610200 | Dumpling, fried, Puerto Rican style | 18.113 |  |  |
| 55610300 | Dumpling, plain | 11.699 | Dumpling, no meat | 11.699 |
| 55702000 | Cake or pancake made with rice flour and/or dried beans | 9.905 | Idli | 9.905 |
| 55801000 | Funnel cake with sugar | 11.660 | Funnel cake with sugar | 11.660 |
| 55801010 | Funnel cake with sugar and fruit | 8.552 | Funnel cake with sugar and fruit | 8.552 |
| 56112000 | Noodles, cooked | 47.714 | Noodles, cooked | 47.714 |
| 56113000 |  |  | Noodles, whole grain, cooked | 47.744 |
| 56113990 | Noodles, vegetable, cooked | 47.714 |  |  |
| 56116000 | Noodles, chow mein | 48.000 | Noodles, chow mein | 48.000 |
| 56130000 | Pasta, cooked | 47.714 | Pasta, cooked | 47.714 |
| 56132990 |  |  | Pasta, whole grain, cooked | 47.714 |
| 56201550 | Cornmeal dumpling | 3.632 |  |  |
| 56205070 | Rice, sweet, cooked with honey | 0.467 | Rice, sweet, cooked with honey | 0.467 |
| 56206990 | Cream of wheat, NS as to regular, quick, or instant, NS as to fat added in cooking | 5.537 | Cream of wheat, NFS | 5.537 |
| 56207000 | Cream of wheat, NS as to regular, quick, or instant, fat not added in cooking | 5.639 |  |  |
| 56207005 | Cream of wheat, NS as to regular, quick, or instant, fat added in cooking | 5.537 |  |  |
| 56207015 | Cream of wheat, regular or quick, made with water, NS as to fat added in cooking | 5.537 |  |  |
| 56207016 | Cream of wheat, regular or quick, made with water, fat not added in cooking | 5.639 | Cream of wheat, regular or quick, made with water, no added fat | 5.639 |
| 56207017 | Cream of wheat, regular or quick, made with water, fat added in cooking | 5.537 | Cream of wheat, regular or quick, made with water, fat added | 5.537 |
| 56207021 | Cream of wheat, regular or quick, made with milk, NS as to fat added in cooking | 5.537 |  |  |
| 56207022 | Cream of wheat, regular or quick, made with milk, fat not added in cooking | 5.639 |  |  |
| 56207023 | Cream of wheat, regular or quick, made with milk, fat added in cooking | 5.537 |  |  |
| 56207025 | Cream of wheat, regular or quick, made with non-dairy milk, NS as to fat added in cooking | 5.537 |  |  |
| 56207026 | Cream of wheat, regular or quick, made with non-dairy milk, fat not added in cooking | 5.639 |  |  |
| 56207027 | Cream of wheat, regular or quick, made with non-dairy milk, fat added in cooking | 5.537 |  |  |
| 56207030 | Cream of wheat, instant, made with water, fat not added in cooking | 6.892 | Cream of wheat, instant, made with water, no added fat | 6.892 |
| 56207050 | Wheat, cream of, cooked, made with milk and sugar, Puerto Rican style | 4.656 |  |  |
| 56207060 | Cream of wheat, instant, made with water, fat added in cooking | 6.720 | Cream of wheat, instant, made with water, fat added | 6.720 |
| 56207070 | Cream of wheat, instant, made with water, NS as to fat added in cooking | 6.720 |  |  |
| 56207094 | Cream of wheat, instant, made with milk, fat added in cooking | 6.720 |  |  |
| 56207095 | Cream of wheat, instant, made with milk, fat not added in cooking | 6.892 |  |  |
| 56207096 | Cream of wheat, instant, made with milk, NS as to fat added in cooking | 6.720 |  |  |
| 56207101 | Cream of wheat, instant, made with non-dairy milk, NS as to fat added in cooking | 6.720 |  |  |
| 56207102 | Cream of wheat, instant, made with non-dairy milk, fat not added in cooking | 6.892 |  |  |
| 56207103 | Cream of wheat, instant, made with non-dairy milk, fat added in cooking | 6.720 |  |  |
| 56207110 |  |  | Bulgur, no added fat | 47.735 |
| 56207120 |  |  | Bulgur, fat added | 44.664 |
| 56207130 |  |  | Bulgur, NS as to fat | 47.735 |
| 56207160 |  |  | Couscous, plain, cooked | 47.772 |
| 56207190 | Whole wheat cereal, cooked, NS as to fat added in cooking | 5.537 |  |  |
| 56207200 | Whole wheat cereal, cooked, fat not added in cooking | 5.639 | Whole wheat cereal, cooked | 5.639 |
| 56207210 | Whole wheat cereal, cooked, fat added in cooking | 5.537 |  |  |
| 56207370 | Wheat cereal, chocolate flavored, cooked | 5.491 | Wheat cereal, chocolate flavored, cooked | 5.491 |
| 56209000 | Cream of rye | 1.645 |  |  |
| 57100100 |  |  | Cereal, ready-to-eat, NFS | 12.000 |
| 57101000 | Cereal (Kellogg's All-Bran) | 50.000 |  |  |
| 57125000 |  |  | Cereal, cinnamon toast | 16.643 |
| 57207000 |  |  | Cereal, bran flakes, plain | 38.608 |
| 57214000 |  |  | Cereal, shredded wheat, flavored | 38.074 |
| 57221700 |  |  | Cereal, fruit rings | 4.766 |
| 57237100 |  |  | Cereal, oat bunches | 11.956 |
| 57240100 |  |  | Cereal, corn squares, flavored | 5.278 |
| 57301600 |  |  | Cereal, multigrain | 14.172 |
| 57329000 |  |  | Cereal, bran flakes, flavored | 30.592 |
| 57344000 |  |  | Cereal, K's, plain | 17.147 |
| 57344010 |  |  | Cereal, K's, flavored | 16.399 |
| 57347000 |  |  | Cereal, flavored puffs | 12.808 |
| 57401100 | Cereal, toasted oat | 48.000 | Cereal, O's, NFS | 48.000 |
| 57411000 |  |  | Cereal, wheat squares | 41.922 |
| 57412000 |  |  | Wheat germ | 48.000 |
| 57416000 | Cereal, puffed wheat, plain | 48.000 | Cereal, plain puffs | 48.000 |
| 57417000 | Cereal (Post Shredded Wheat) | 48.000 | Cereal, shredded wheat, plain | 48.000 |
| 57418000 |  |  | Cereal, wheat flakes | 40.226 |
| 57420100 |  |  | Cereal, other, NFS | 12.000 |
| 57420150 |  |  | Cereal, other, honey | 24.000 |
| 57420160 |  |  | Cereal, reduced sugar | 12.530 |
| 57601100 |  |  | Wheat bran | 48.000 |
| 58100000 | Burrito, taco, or quesadilla with egg | 17.728 |  |  |
| 58100005 | Burrito, taco, or quesadilla with egg and potato | 14.907 |  |  |
| 58100010 | Burrito, taco, or quesadilla with egg and breakfast meat | 15.335 |  |  |
| 58100015 | Burrito, taco, or quesadilla with egg, potato, and breakfast meat | 13.178 |  |  |
| 58100017 | Burrito, taco, or quesadilla with egg, potato, and breakfast meat, from fast food | 13.969 |  |  |
| 58100020 | Burrito, taco, or quesadilla with egg, beans, and breakfast meat | 12.637 |  |  |
| 58100100 | Burrito with meat | 12.630 |  |  |
| 58100120 | Burrito with meat and beans | 12.278 |  |  |
| 58100135 | Burrito with meat and sour cream | 10.890 |  |  |
| 58100140 | Burrito with meat, beans, and sour cream | 10.627 |  |  |
| 58100160 | Burrito with meat, beans, and rice | 11.462 |  |  |
| 58100165 | Burrito with meat, beans, rice, and sour cream | 10.010 |  |  |
| 58100200 | Burrito with chicken | 15.058 |  |  |
| 58100220 | Burrito with chicken and beans | 13.322 |  |  |
| 58100235 | Burrito with chicken and sour cream | 12.648 |  |  |
| 58100245 | Burrito with chicken, beans, and sour cream | 11.400 |  |  |
| 58100255 | Burrito with chicken, beans, and rice | 12.367 |  |  |
| 58100260 | Burrito with chicken, beans, rice, and sour cream | 10.693 |  |  |
| 58100300 | Burrito with beans and rice, meatless | 11.171 |  |  |
| 58100320 | Burrito with beans, meatless | 11.944 |  |  |
| 58100330 | Burrito with beans, rice, and sour cream, meatless | 9.787 |  |  |
| 58100360 | Chilaquiles, tortilla casserole with salsa, cheese, and egg | 11.481 | Chilaquiles | 11.481 |
| 58100370 | Chilaquiles, tortilla casserole with salsa and cheese, no egg | 13.541 |  |  |
| 58100520 | Enchilada with meat and beans, red-chile or enchilada sauce | 5.540 |  |  |
| 58100525 | Enchilada with meat and beans, green-chile or enchilada sauce | 13.307 |  |  |
| 58100530 | Enchilada with meat, red-chile or enchilada sauce | 5.961 |  |  |
| 58100535 | Enchilada with meat, green-chile or enchilada sauce | 14.393 |  |  |
| 58100620 | Enchilada with chicken and beans, red-chile or enchilada sauce | 5.536 |  |  |
| 58100625 | Enchilada with chicken and beans, green-chile or enchilada sauce | 13.298 |  |  |
| 58100630 | Enchilada with chicken, red-chile or enchilada sauce | 5.953 |  |  |
| 58100635 | Enchilada with chicken, green-chile or enchilada sauce | 14.373 |  |  |
| 58100720 | Enchilada with beans, meatless, red-chile or enchilada sauce | 5.174 |  |  |
| 58100725 | Enchilada with beans, green-chile or enchilada sauce | 12.373 |  |  |
| 58100800 | Enchilada, just cheese, meatless, no beans, red-chile or enchilada sauce | 6.125 |  |  |
| 58100805 | Enchilada, just cheese, meatless, no beans, green-chile or enchilada sauce | 14.820 |  |  |
| 58101320 | Taco or tostada with meat | 9.066 |  |  |
| 58101325 | Taco or tostada with meat and sour cream | 7.885 |  |  |
| 58101345 | Soft taco with meat | 22.901 |  |  |
| 58101350 | Soft taco with meat and sour cream | 20.227 |  |  |
| 58101450 | Soft taco with chicken | 19.564 |  |  |
| 58101460 | Soft taco with chicken and sour cream | 16.779 |  |  |
| 58101520 | Taco or tostada with chicken | 1.007 |  |  |
| 58101525 | Taco or tostada with chicken and sour cream | 0.843 |  |  |
| 58101540 | Taco or tostada with fish | 1.002 |  |  |
| 58101555 | Soft taco with fish | 19.473 |  |  |
| 58101610 | Soft taco with beans | 13.082 |  |  |
| 58101615 | Soft taco with beans and sour cream | 11.723 |  |  |
| 58101620 | Soft taco with meat and beans | 17.665 |  |  |
| 58101625 | Soft taco with chicken and beans | 15.748 |  |  |
| 58101630 | Soft taco with meat, beans, and sour cream | 15.723 |  |  |
| 58101635 | Soft taco with chicken, beans, and sour cream | 13.856 |  |  |
| 58101720 | Taco or tostada with beans | 0.020 |  |  |
| 58101725 | Taco or tostada with beans and sour cream | 0.017 |  |  |
| 58101730 | Taco or tostada with meat and beans | 4.208 |  |  |
| 58101735 | Taco or tostada with chicken and beans | 0.414 |  |  |
| 58101745 | Taco or tostada with meat, beans, and sour cream | 3.696 |  |  |
| 58101750 | Taco or tostada with chicken, beans, and sour cream | 0.358 |  |  |
| 58101800 | Ground beef with tomato sauce and taco seasonings on a cornbread crust | 8.327 |  |  |
| 58101820 | Mexican casserole made with ground beef, beans, tomato sauce, cheese, taco seasonings, and corn chips | 11.558 |  |  |
| 58101830 | Mexican casserole made with ground beef, tomato sauce, cheese, taco seasonings, and corn chips | 8.650 | Frito pie | 8.650 |
| 58101930 | Taco or tostada salad with meat | 14.425 | Taco or tostada salad with meat | 14.425 |
| 58101935 | Taco or tostada salad with chicken | 14.255 | Taco or tostada salad with chicken | 14.255 |
| 58101940 | Taco or tostada salad, meatless | 14.480 | Taco or tostada salad, meatless | 14.480 |
| 58101945 | Taco or tostada salad with meat and sour cream | 12.688 | Taco or tostada salad with meat and sour cream | 12.688 |
| 58101950 | Taco or tostada salad with chicken and sour cream | 12.556 | Taco or tostada salad with chicken and sour cream | 12.556 |
| 58101955 | Taco or tostada salad, meatless with sour cream | 12.717 | Taco or tostada salad, meatless with sour cream | 12.717 |
| 58103130 | Tamale with chicken | 1.082 | Tamale, chicken | 1.082 |
| 58103200 | Tamale, plain, meatless, no sauce, Puerto Rican style or Carribean Style | 2.013 |  |  |
| 58103210 | Tamale, meatless, with sauce, Puerto Rican or Caribbean style | 1.226 |  |  |
| 58103310 | Tamale casserole with meat | 7.064 | Tamale casserole with meat | 7.064 |
| 58104260 | Gordita, sope, or chalupa with beans | 11.017 | Gordita, with beans | 11.017 |
| 58104270 | Gordita, sope, or chalupa with beans and sour cream | 10.037 |  |  |
| 58104280 | Gordita, sope, or chalupa with meat and sour cream | 17.014 |  |  |
| 58104290 | Gordita, sope, or chalupa with meat | 18.867 | Gordita, meat | 18.867 |
| 58104320 | Gordita, sope, or chalupa with chicken and sour cream | 13.639 |  |  |
| 58104340 | Gordita, sope, or chalupa with chicken | 15.423 |  |  |
| 58104500 | Chimichanga with meat | 21.743 | Chimichanga, meat | 21.743 |
| 58104520 | Chimichanga, meatless | 17.812 | Chimichanga, with beans | 17.812 |
| 58104530 | Chimichanga with chicken | 20.769 | Chimichanga, chicken | 20.769 |
| 58104535 | Chimichanga with meat and sour cream | 24.915 |  |  |
| 58104540 | Chimichanga, meatless, with sour cream | 16.190 |  |  |
| 58104550 | Chimichanga with chicken and sour cream | 18.612 |  |  |
| 58104710 | Quesadilla, just cheese, meatless | 30.698 | Quesadilla, cheese only | 30.698 |
| 58104720 | Quesadilla, just cheese, from fast food | 27.840 |  |  |
| 58104730 | Quesadilla with meat | 27.433 | Quesadilla, beef or pork | 27.433 |
| 58104740 | Quesadilla with chicken | 27.411 | Quesadilla, chicken | 27.411 |
| 58104750 | Quesadilla with vegetables | 27.810 | Quesadilla, with vegetables | 27.810 |
| 58104760 | Quesadilla with vegetables and meat | 27.622 | Quesadilla, beef or pork, with vegetables | 27.622 |
| 58104770 | Quesadilla with vegetables and chicken | 27.611 | Quesadilla, chicken, with vegetables | 27.611 |
| 58104900 | Taquito or flauta with egg | 25.412 | Taquito, egg | 25.412 |
| 58104905 | Taquito or flauta with egg and breakfast meat | 24.000 |  |  |
| 58105000 | Fajita with chicken and vegetables | 24.066 | Fajita, chicken | 24.066 |
| 58105050 | Fajita with meat and vegetables | 24.066 | Fajita, beef or pork | 24.066 |
| 58105060 |  |  | Fajita, shrimp | 8.468 |
| 58105075 | Fajita with vegetables | 23.261 | Fajita, vegetable | 23.261 |
| 58106300 | Pizza, cheese, with vegetables, from frozen, thin crust | 4.934 | Pizza, cheese, with vegetables, from frozen, thin crust | 4.934 |
| 58106305 | Pizza, cheese with vegetables, from frozen, thick crust | 2.654 | Pizza, cheese with vegetables, from frozen, thick crust | 2.654 |
| 58106320 | Pizza, cheese, with vegetables, from restaurant or fast food, thin crust | 5.656 | Pizza, cheese, with vegetables, from restaurant or fast food, thin crust | 5.656 |
| 58106325 | Pizza, cheese, with vegetables, from restaurant or fast food, medium crust | 4.260 | Pizza, cheese, with vegetables, from restaurant or fast food, medium crust | 4.260 |
| 58106330 | Pizza, cheese, with vegetables, from restaurant or fast food, thick crust | 4.490 | Pizza, cheese, with vegetables, from restaurant or fast food, thick crust | 4.490 |
| 58106345 | Pizza with cheese and extra vegetables, thin crust | 5.666 | Pizza with cheese and extra vegetables, thin crust | 5.666 |
| 58106347 | Pizza with cheese and extra vegetables, medium crust | 4.395 | Pizza with cheese and extra vegetables, medium crust | 4.395 |
| 58106350 | Pizza with cheese and extra vegetables, thick crust | 2.887 | Pizza with cheese and extra vegetables, thick crust | 2.887 |
| 58106720 | Pizza with meat and vegetables, from restaurant or fast food, thin crust | 4.760 | Pizza with meat and vegetables, from restaurant or fast food, thin crust | 4.760 |
| 58106725 |  |  | Pizza with meat and vegetables, from restaurant or fast food, medium crust | 3.497 |
| 58106730 | Pizza with meat and vegetables, from restaurant or fast food, thick crust | 2.185 | Pizza with meat and vegetables, from restaurant or fast food, thick crust | 2.185 |
| 58106736 | Pizza with extra meat and extra vegetables, thin crust | 3.843 | Pizza with extra meat and extra vegetables, thin crust | 3.843 |
| 58106737 | Pizza with extra meat and extra vegetables, thick crust | 2.699 | Pizza with extra meat and extra vegetables, thick crust | 2.699 |
| 58106738 | Pizza with extra meat and extra vegetables, medium crust | 2.924 | Pizza with extra meat and extra vegetables, medium crust | 2.924 |
| 58107050 | Pizza, no cheese, thin crust | 19.267 | Pizza, no cheese, thin crust | 19.267 |
| 58107100 | Pizza, no cheese, thick crust | 20.921 | Pizza, no cheese, thick crust | 20.921 |
| 58107205 | White pizza, cheese, thin crust | 27.006 | White pizza, cheese, thin crust | 27.006 |
| 58107207 | White pizza, cheese, thick crust | 31.344 | White pizza, cheese, thick crust | 31.344 |
| 58107212 | White pizza, cheese, with vegetables, thin crust | 27.012 | White pizza, cheese, with vegetables, thin crust | 27.012 |
| 58107214 | White pizza, cheese, with vegetables, thick crust | 30.630 | White pizza, cheese, with vegetables, thick crust | 30.630 |
| 58107222 | White pizza, cheese, with meat, thin crust | 21.971 | White pizza, cheese, with meat, thin crust | 21.971 |
| 58107224 | White pizza, cheese, with meat, thick crust | 26.522 | White pizza, cheese, with meat, thick crust | 26.522 |
| 58107232 | White pizza, cheese, with meat and vegetables, thin crust | 24.190 | White pizza, cheese, with meat and vegetables, thin crust | 24.190 |
| 58107234 | White pizza, cheese, with meat and vegetables, thick crust | 27.933 | White pizza, cheese, with meat and vegetables, thick crust | 27.933 |
| 58108000 | Calzone, with cheese, meatless | 12.591 | Calzone, with cheese, meatless | 12.591 |
| 58108010 | Calzone, with meat and cheese | 14.291 | Calzone, with meat and cheese | 14.291 |
| 58109015 | Pizza, cheese, whole wheat thin crust | 22.737 | Pizza, cheese, whole wheat thin crust | 22.737 |
| 58109020 | Pizza, cheese, whole wheat thick crust | 27.200 | Pizza, cheese, whole wheat thick crust | 27.200 |
| 58109030 | Pizza, with meat, whole wheat thin crust | 18.462 | Pizza, with meat, whole wheat thin crust | 18.462 |
| 58109040 | Pizza, with meat, whole wheat thick crust | 22.986 | Pizza, with meat, whole wheat thick crust | 22.986 |
| 58109050 | Pizza, cheese and vegetables, whole wheat thin crust | 23.603 | Pizza, cheese and vegetables, whole wheat thin crust | 23.603 |
| 58109060 | Pizza, cheese and vegetables, whole wheat thick crust | 27.172 | Pizza, cheese and vegetables, whole wheat thick crust | 27.172 |
| 58109100 |  |  | Pizza, cheese, gluten-free thin crust | 7.579 |
| 58109110 |  |  | Pizza, cheese, gluten-free thick crust | 6.000 |
| 58109120 |  |  | Pizza, with meat, gluten-free thin crust | 6.154 |
| 58109130 |  |  | Pizza, with meat, gluten-free thick crust | 5.070 |
| 58109140 | Pizza, cheese and vegetables, gluten-free thin crust | 5.452 | Pizza, cheese and vegetables, gluten-free thin crust | 5.452 |
| 58109150 | Pizza, cheese and vegetables, gluten-free thick crust | 4.506 | Pizza, cheese and vegetables, gluten-free thick crust | 4.506 |
| 58109210 | Breakfast pizza with egg | 24.242 | Breakfast pizza with egg | 24.242 |
| 58110200 | Roll with meat and/or shrimp, vegetables and rice paper, not fried | 4.211 | Roll with meat and/or shrimp, vegetables and rice paper, not fried | 4.211 |
| 58111200 | Puffs, fried, crab meat and cream cheese filled | 15.489 | Puffs, fried, crab meat and cream cheese filled | 15.489 |
| 58115110 | Tamale casserole, Puerto Rican style | 10.174 |  |  |
| 58115150 | Tamal in a leaf, Puerto Rican style | 8.220 |  |  |
| 58115210 | Taco with crab meat, Puerto Rican style | 4.298 |  |  |
| 58116110 | Meat turnover, Puerto Rican style | 17.457 |  |  |
| 58116115 | Empanada, Mexican turnover, filled with cheese and vegetables | 18.300 | Empanada, no meat | 18.300 |
| 58116130 | Empanada, Mexican turnover, filled with chicken and vegetables | 17.829 | Empanada, chicken, with vegetables | 17.829 |
| 58116210 | Meat pie, Puerto Rican style | 12.952 |  |  |
| 58116310 | Cheese turnover, Puerto Rican style | 20.188 |  |  |
| 58117310 | Kibby, Puerto Rican style | 14.864 | Kibby, Puerto Rican style | 14.864 |
| 58117410 | Codfish fritter, Puerto Rican style | 16.123 | Bacalaitos fritos | 16.123 |
| 58117510 | Hayacas, Puerto Rican style | 20.657 | Hayacas, Puerto Rican style | 20.657 |
| 58120110 | Crepe, filled with meat, poultry, or seafood, with sauce | 2.319 | Crepe, with meat | 2.319 |
| 58120120 | Crepe, filled with meat, poultry, or seafood, no sauce | 3.109 |  |  |
| 58121510 | Dumpling, meat-filled | 14.826 |  |  |
| 58121620 | Dumpling, vegetable | 19.768 |  |  |
| 58122210 | Gnocchi, cheese | 4.089 | Gnocchi, cheese | 4.089 |
| 58122220 | Gnocchi, potato | 9.112 | Gnocchi, potato | 9.112 |
| 58122250 | Kishke, stuffed derma | 45.278 |  |  |
| 58122310 | Knish, potato | 19.592 |  |  |
| 58122320 | Knish, cheese | 16.821 | Knish | 16.821 |
| 58122330 | Knish, meat | 26.858 |  |  |
| 58123110 | Sweet bread dough, filled with meat, steamed | 19.215 | Bao bun | 19.215 |
| 58123120 | Sweet bread dough, filled with bean paste, meatless, steamed | 20.392 | Bao bun, no meat | 20.392 |
| 58124210 | Pastry, cheese-filled | 9.763 | Pastry, cheese-filled | 9.763 |
| 58124220 | Pastry, egg and cheese filled | 23.071 | Pastry, egg and cheese filled | 23.071 |
| 58124230 | Pastry, meat / poultry-filled | 16.066 | Pastry, meat / poultry-filled | 16.066 |
| 58124250 | Spanakopitta | 7.593 | Spanakopita | 7.593 |
| 58124500 | Pastry, filled with potatoes and peas, fried | 15.712 | Samosa | 15.712 |
| 58125110 | Quiche with meat, poultry or fish | 11.786 | Quiche with meat, poultry or fish | 11.786 |
| 58125120 | Spinach quiche, meatless | 10.909 | Spinach quiche, meatless | 10.909 |
| 58125180 | Cheese quiche, meatless | 13.656 | Cheese quiche, meatless | 13.656 |
| 58126000 | Turnover filled with ground beef and cabbage | 20.543 |  |  |
| 58126110 | Turnover, meat-filled, no gravy | 15.379 |  |  |
| 58126120 | Turnover, meat-filled, with gravy | 9.629 |  |  |
| 58126130 | Turnover, meat- and cheese-filled, no gravy | 14.400 | Turnover or hot pocket, ham | 14.400 |
| 58126140 | Turnover, meat- and bean-filled, no gravy | 15.799 |  |  |
| 58126150 | Turnover, meat- and cheese-filled, tomato-based sauce | 14.400 | Turnover or hot pocket, pizza pocket, meat | 14.400 |
| 58126160 | Turnover, cheese-filled, tomato-based sauce | 14.400 | Turnover or hot pocket, pizza pocket | 14.400 |
| 58126170 | Turnover filled with meat and vegetable, no potatoes, no gravy | 16.378 |  |  |
| 58126180 | Turnover, meat-, potato-, and vegetable-filled, no gravy | 19.710 |  |  |
| 58126270 | Turnover, chicken- or turkey-, and cheese-filled, no gravy | 14.400 | Turnover or hot pocket, chicken | 14.400 |
| 58126280 | Turnover, chicken- or turkey-, and vegetable-filled, lower in fat | 14.400 |  |  |
| 58126290 | Turnover, meat- and cheese-filled, lower in fat | 14.400 | Turnover or lean pocket | 14.400 |
| 58126300 | Turnover, meat- and cheese-filled, tomato-based sauce, lower in fat | 14.400 |  |  |
| 58126310 | Turnover, chicken, with gravy | 8.963 |  |  |
| 58126400 | Turnover, filled with egg, meat and cheese | 14.400 | Turnover or breakfast pocket, egg | 14.400 |
| 58126410 | Turnover, filled with egg, meat, and cheese, lower in fat | 14.400 |  |  |
| 58127110 | Vegetables in pastry | 4.434 |  |  |
| 58127150 | Vegetables and cheese in pastry | 6.624 |  |  |
| 58127200 | Croissant sandwich, filled with broccoli and cheese | 13.192 |  |  |
| 58127210 | Croissant sandwich, filled with ham and cheese | 12.001 |  |  |
| 58127220 | Croissant sandwich, filled with chicken, broccoli, and cheese sauce | 11.094 |  |  |
| 58127500 | Vegetable submarine sandwich, with fat free spread | 25.514 |  |  |
| 58127510 | Vegetable submarine sandwich, with spread | 24.762 |  |  |
| 58128000 | Biscuit with gravy | 0.960 | Biscuit with gravy | 0.960 |
| 58128120 | Cornmeal dressing with chicken or turkey and vegetables | 13.882 | Cornmeal dressing with chicken or turkey and vegetables | 13.882 |
| 58128210 | Dressing with oysters | 26.770 | Dressing with oysters | 26.770 |
| 58128220 | Dressing with chicken or turkey and vegetables | 31.407 | Dressing with chicken or turkey and vegetables | 31.407 |
| 58128250 | Dressing with meat and vegetables | 26.461 | Dressing with meat and vegetables | 26.461 |
| 58130011 | Lasagna with meat | 12.480 | Lasagna with meat | 12.480 |
| 58130014 | Lasagna with meat, from restaurant | 12.480 | Lasagna with meat, from restaurant | 12.480 |
| 58130015 | Lasagna with meat, home recipe | 15.713 | Lasagna with meat, home recipe | 15.713 |
| 58130016 | Lasagna with meat, frozen | 12.480 | Lasagna with meat, frozen | 12.480 |
| 58130020 | Lasagna with meat and spinach | 15.074 | Lasagna with meat and spinach | 15.074 |
| 58130140 | Lasagna with chicken or turkey | 15.435 | Lasagna with chicken or turkey | 15.435 |
| 58130150 | Lasagna, with chicken or turkey, and spinach | 15.074 | Lasagna, with chicken or turkey, and spinach | 15.074 |
| 58130310 | Lasagna, meatless | 12.480 | Lasagna, meatless | 12.480 |
| 58130320 | Lasagna, meatless, with vegetables | 15.494 | Lasagna, meatless, with vegetables | 15.494 |
| 58130610 | Lasagna with meat, whole wheat noodles | 3.134 |  |  |
| 58130810 | Lasagna, meatless, whole wheat noodles | 3.104 |  |  |
| 58130910 | Lasagna with meat, spinach noodles | 15.460 |  |  |
| 58130950 | Lasagna, meatless, spinach noodles | 15.494 |  |  |
| 58131100 | Ravioli, NS as to filling, no sauce | 16.656 | Ravioli, NS as to filling, no sauce | 16.656 |
| 58131110 | Ravioli, NS as to filling, with tomato sauce | 12.175 | Ravioli, NS as to filling, with tomato sauce | 12.175 |
| 58131120 | Ravioli, NS as to filling, with cream sauce | 9.977 | Ravioli, NS as to filling, with cream sauce | 9.977 |
| 58131310 | Ravioli, meat-filled, no sauce | 16.447 | Ravioli, meat-filled, no sauce | 16.447 |
| 58131320 | Ravioli, meat-filled, with tomato sauce or meat sauce | 12.238 | Ravioli, meat-filled, with tomato sauce or meat sauce | 12.238 |
| 58131330 | Ravioli, meat-filled, with cream sauce | 9.939 | Ravioli, meat-filled, with cream sauce | 9.939 |
| 58131510 | Ravioli, cheese-filled, no sauce | 16.376 | Ravioli, cheese-filled, no sauce | 16.376 |
| 58131530 | Ravioli, cheese-filled, with meat sauce | 10.957 | Ravioli, cheese-filled, with meat sauce | 10.957 |
| 58131535 | Ravioli, cheese-filled, with cream sauce | 9.623 | Ravioli, cheese-filled, with cream sauce | 9.623 |
| 58131590 | Ravioli, cheese and spinach-filled, no sauce | 16.110 | Ravioli, cheese and spinach-filled, no sauce | 16.110 |
| 58131600 | Ravioli, cheese and spinach-filled, with cream sauce | 9.533 | Ravioli, cheese and spinach-filled, with cream sauce | 9.533 |
| 58131610 | Ravioli, cheese and spinach filled, with tomato sauce | 10.229 | Ravioli, cheese and spinach filled, with tomato sauce | 10.229 |
| 58133110 | Manicotti, cheese-filled, no sauce | 10.915 | Manicotti, cheese-filled, no sauce | 10.915 |
| 58133120 | Manicotti, cheese-filled, with tomato sauce, meatless | 9.464 | Manicotti, cheese-filled, with tomato sauce, meatless | 9.464 |
| 58133130 | Manicotti, cheese-filled, with meat sauce | 8.831 | Manicotti, cheese-filled, with meat sauce | 8.831 |
| 58133140 | Manicotti, vegetable- and cheese-filled, with tomato sauce, meatless | 9.467 | Manicotti, vegetable- and cheese-filled, with tomato sauce, meatless | 9.467 |
| 58134110 | Stuffed shells, cheese-filled, no sauce | 12.436 | Stuffed shells, cheese-filled, no sauce | 12.436 |
| 58134120 | Stuffed shells, cheese-filled, with tomato sauce, meatless | 10.269 | Stuffed shells, cheese-filled, with tomato sauce, meatless | 10.269 |
| 58134130 | Stuffed shells, cheese-filled, with meat sauce | 9.528 | Stuffed shells, cheese-filled, with meat sauce | 9.528 |
| 58134160 | Stuffed shells, cheese- and spinach- filled, no sauce | 13.179 | Stuffed shells, cheese- and spinach- filled, no sauce | 13.179 |
| 58134210 | Stuffed shells, with chicken, with tomato sauce | 10.583 | Stuffed shells, with chicken, with tomato sauce | 10.583 |
| 58134310 | Stuffed shells, with fish and/or shellfish, with tomato sauce | 10.501 | Stuffed shells, with fish and/or shellfish, with tomato sauce | 10.501 |
| 58134610 | Tortellini, meat-filled, with tomato sauce | 28.433 | Tortellini, meat-filled, with tomato sauce | 28.433 |
| 58134613 | Tortellini, meat-filled, with tomato sauce, canned | 25.440 | Tortellini, meat-filled, with tomato sauce, canned | 25.440 |
| 58134620 |  |  | Tortellini, cheese-filled, meatless, with tomato sauce | 22.468 |
| 58134623 | Tortellini, cheese-filled, meatless, with tomato sauce, canned | 10.865 | Tortellini, cheese-filled, meatless, with tomato sauce, canned | 10.865 |
| 58134630 | Tortellini, cheese-filled, meatless, with vegetables and vinaigrette dressing | 19.128 |  |  |
| 58134640 | Tortellini, cheese-filled, meatless, with vinaigrette dressing | 15.358 | Tortellini, cheese-filled, meatless, with vinaigrette dressing | 15.358 |
| 58134650 | Tortellini, meat-filled, no sauce | 13.767 | Tortellini, meat-filled, no sauce | 13.767 |
| 58134660 | Tortellini, cheese-filled, with cream sauce | 11.353 | Tortellini, cheese-filled, with cream sauce | 11.353 |
| 58134680 |  |  | Tortellini, cheese-filled, no sauce | 36.923 |
| 58134710 | Tortellini, spinach-filled, with tomato sauce | 28.433 | Tortellini, spinach-filled, with tomato sauce | 28.433 |
| 58134720 | Tortellini, spinach-filled, no sauce | 16.133 | Tortellini, spinach-filled, no sauce | 16.133 |
| 58134810 | Cannelloni, cheese- and spinach-filled, no sauce | 22.616 | Cannelloni, cheese- and spinach-filled, no sauce | 22.616 |
| 58135110 | Chow fun noodles with meat and vegetables | 10.372 | Chow fun noodles with meat and vegetables | 10.372 |
| 58135120 | Chow fun noodles with vegetables, meatless | 13.040 | Chow fun noodles with vegetables, meatless | 13.040 |
| 58137220 | Pad Thai, meatless | 4.221 | Pad Thai, meatless | 4.221 |
| 58137230 | Pad Thai with chicken | 3.967 | Pad Thai with chicken | 3.967 |
| 58137240 | Pad Thai with seafood | 4.095 | Pad Thai with seafood | 4.095 |
| 58137250 | Pad Thai with meat | 4.070 | Pad Thai with meat | 4.070 |
| 58137300 | Adobo, with noodles | 0.806 | Adobo, with noodles | 0.806 |
| 58140110 | Spaghetti with corned beef, Puerto Rican style | 30.105 |  |  |
| 58140310 | Macaroni with tuna, Puerto Rican style | 21.332 | Macaroni with tuna, Puerto Rican style | 21.332 |
| 58145110 | Macaroni or noodles with cheese | 24.486 | Macaroni or noodles with cheese | 24.486 |
| 58145117 | Macaroni or noodles with cheese, Easy Mac type | 6.948 | Macaroni or noodles with cheese, Easy Mac type | 6.948 |
| 58145119 | Macaroni or noodles with cheese, made from reduced fat packaged mix | 7.888 | Macaroni or noodles with cheese, made from reduced fat packaged mix | 7.888 |
| 58145120 | Macaroni or noodles with cheese and tuna | 20.096 | Macaroni or noodles with cheese and tuna | 20.096 |
| 58145135 | Macaroni or noodles with cheese and meat | 16.705 | Macaroni or noodles with cheese and meat | 16.705 |
| 58145136 | Macaroni or noodles with cheese and meat, prepared from Hamburger Helper mix | 2.927 | Macaroni or noodles with cheese and meat, prepared from Hamburger Helper mix | 2.927 |
| 58145140 | Macaroni or noodles with cheese and tomato | 19.750 | Macaroni or noodles with cheese and tomato | 19.750 |
| 58145160 | Macaroni or noodles with cheese and frankfurters or hot dogs | 20.003 | Macaroni or noodles with cheese and frankfurters or hot dogs | 20.003 |
| 58145170 | Macaroni or noodles with cheese and egg | 20.800 | Macaroni or noodles with cheese and egg | 20.800 |
| 58145190 | Macaroni or noodles with cheese and chicken or turkey | 16.356 | Macaroni or noodles with cheese and chicken or turkey | 16.356 |
| 58145300 |  |  | Macaroni or noodles with cheese, whole grain | 23.653 |
| 58146120 | Pasta with tomato-based sauce, cheese and meat | 10.229 | Pasta with tomato-based sauce, cheese and meat | 10.229 |
| 58146150 | Pasta with tomato-based sauce and cheese | 15.511 | Pasta with tomato-based sauce and cheese | 15.511 |
| 58146160 | Pasta with vegetables, no sauce or dressing | 31.796 | Pasta with vegetables, no sauce or dressing | 31.796 |
| 58146200 | Pasta, meat-filled, with gravy, canned | 11.516 |  |  |
| 58146210 | Pasta with sauce, NFS | 24.960 | Pasta with sauce, NFS | 24.960 |
| 58146215 |  |  | Pasta with sauce, meatless, school lunch | 47.911 |
| 58146221 | Pasta with tomato-based sauce, restaurant | 23.053 | Pasta with tomato-based sauce, restaurant | 23.053 |
| 58146222 | Pasta with tomato-based sauce, home recipe | 24.889 | Pasta with tomato-based sauce, home recipe | 24.889 |
| 58146223 | Pasta with tomato-based sauce, ready-to-heat | 24.348 | Pasta with tomato-based sauce, ready-to-heat | 24.348 |
| 58146301 | Pasta with tomato-based sauce, and added vegetables, restaurant | 23.053 | Pasta with tomato-based sauce, and added vegetables, restaurant | 23.053 |
| 58146302 | Pasta with tomato-based sauce, and added vegetables, home recipe | 24.889 | Pasta with tomato-based sauce, and added vegetables, home recipe | 24.889 |
| 58146303 | Pasta with tomato-based sauce, and added vegetables, ready-to-heat | 24.348 | Pasta with tomato-based sauce, and added vegetables, ready-to-heat | 24.348 |
| 58146315 |  |  | Pasta with sauce and meat, from school lunch | 43.128 |
| 58146321 | Pasta with tomato-based sauce and meat, restaurant | 23.053 | Pasta with tomato-based sauce and meat, restaurant | 23.053 |
| 58146322 | Pasta with tomato-based sauce and meat, home recipe | 24.889 | Pasta with tomato-based sauce and meat, home recipe | 24.889 |
| 58146323 | Pasta with tomato-based sauce and meat, ready-to-heat | 24.348 | Pasta with tomato-based sauce and meat, ready-to-heat | 24.348 |
| 58146331 | Pasta with tomato-based sauce, meat, and added vegetables, restaurant | 23.053 | Pasta with tomato-based sauce, meat, and added vegetables, restaurant | 23.053 |
| 58146332 | Pasta with tomato-based sauce, meat, and added vegetables, home recipe | 24.889 | Pasta with tomato-based sauce, meat, and added vegetables, home recipe | 24.889 |
| 58146333 | Pasta with tomato-based sauce, meat, and added vegetables, ready-to-heat | 24.348 | Pasta with tomato-based sauce, meat, and added vegetables, ready-to-heat | 24.348 |
| 58146341 | Pasta with tomato-based sauce and poultry, restaurant | 23.053 | Pasta with tomato-based sauce and poultry, restaurant | 23.053 |
| 58146342 | Pasta with tomato-based sauce and poultry, home recipe | 24.889 | Pasta with tomato-based sauce and poultry, home recipe | 24.889 |
| 58146343 | Pasta with tomato-based sauce and poultry, ready-to-heat | 24.348 | Pasta with tomato-based sauce and poultry, ready-to-heat | 24.348 |
| 58146351 | Pasta with tomato-based sauce, poultry, and added vegetables, restaurant | 23.053 | Pasta with tomato-based sauce, poultry, and added vegetables, restaurant | 23.053 |
| 58146352 | Pasta with tomato-based sauce, poultry, and added vegetables, home recipe | 24.889 | Pasta with tomato-based sauce, poultry, and added vegetables, home recipe | 24.889 |
| 58146353 | Pasta with tomato-based sauce, poultry, and added vegetables, ready-to-heat | 24.348 | Pasta with tomato-based sauce, poultry, and added vegetables, ready-to-heat | 24.348 |
| 58146361 | Pasta with tomato-based sauce and seafood, restaurant | 23.053 | Pasta with tomato-based sauce and seafood, restaurant | 23.053 |
| 58146362 | Pasta with tomato-based sauce and seafood, home recipe | 24.889 | Pasta with tomato-based sauce and seafood, home recipe | 24.889 |
| 58146363 | Pasta with tomato-based sauce and seafood, ready-to-heat | 24.348 | Pasta with tomato-based sauce and seafood, ready-to-heat | 24.348 |
| 58146371 | Pasta with tomato-based sauce, seafood, and added vegetables, restaurant | 23.053 | Pasta with tomato-based sauce, seafood, and added vegetables, restaurant | 23.053 |
| 58146372 | Pasta with tomato-based sauce, seafood, and added vegetables, home recipe | 24.889 | Pasta with tomato-based sauce, seafood, and added vegetables, home recipe | 24.889 |
| 58146373 | Pasta with tomato-based sauce, seafood, and added vegetables, ready-to-heat | 24.348 | Pasta with tomato-based sauce, seafood, and added vegetables, ready-to-heat | 24.348 |
| 58146381 | Pasta with cream sauce, restaurant | 22.974 | Pasta with cream sauce, restaurant | 22.974 |
| 58146382 | Pasta with cream sauce, home recipe | 24.889 | Pasta with cream sauce, home recipe | 24.889 |
| 58146383 | Pasta with cream sauce, ready-to-heat | 24.348 | Pasta with cream sauce, ready-to-heat | 24.348 |
| 58146391 | Pasta with cream sauce and added vegetables, restaurant | 22.974 | Pasta with cream sauce and added vegetables, restaurant | 22.974 |
| 58146392 | Pasta with cream sauce and added vegetables, from home recipe | 24.889 | Pasta with cream sauce and added vegetables, from home recipe | 24.889 |
| 58146393 | Pasta with cream sauce and added vegetables, ready-to-heat | 24.348 | Pasta with cream sauce and added vegetables, ready-to-heat | 24.348 |
| 58146401 | Pasta with cream sauce and meat, restaurant | 22.974 | Pasta with cream sauce and meat, restaurant | 22.974 |
| 58146402 | Pasta with cream sauce and meat, home recipe | 24.889 | Pasta with cream sauce and meat, home recipe | 24.889 |
| 58146403 | Pasta with cream sauce and meat, ready-to-heat | 24.348 | Pasta with cream sauce and meat, ready-to-heat | 24.348 |
| 58146411 | Pasta with cream sauce, meat, and added vegetables, restaurant | 22.974 | Pasta with cream sauce, meat, and added vegetables, restaurant | 22.974 |
| 58146412 | Pasta with cream sauce, meat, and added vegetables, home recipe | 24.889 | Pasta with cream sauce, meat, and added vegetables, home recipe | 24.889 |
| 58146413 | Pasta with cream sauce, meat, and added vegetables, ready-to-heat | 24.348 | Pasta with cream sauce, meat, and added vegetables, ready-to-heat | 24.348 |
| 58146421 | Pasta with cream sauce and poultry, restaurant | 22.974 | Pasta with cream sauce and poultry, restaurant | 22.974 |
| 58146422 | Pasta with cream sauce and poultry, home recipe | 24.889 | Pasta with cream sauce and poultry, home recipe | 24.889 |
| 58146423 | Pasta with cream sauce and poultry, ready-to-heat | 24.348 | Pasta with cream sauce and poultry, ready-to-heat | 24.348 |
| 58146431 | Pasta with cream sauce, poultry, and added vegetables, restaurant | 22.974 | Pasta with cream sauce, poultry, and added vegetables, restaurant | 22.974 |
| 58146432 | Pasta with cream sauce, poultry, and added vegetables, home recipe | 24.889 | Pasta with cream sauce, poultry, and added vegetables, home recipe | 24.889 |
| 58146433 | Pasta with cream sauce, poultry, and added vegetables, ready-to-heat | 24.348 | Pasta with cream sauce, poultry, and added vegetables, ready-to-heat | 24.348 |
| 58146441 | Pasta with cream sauce and seafood, restaurant | 22.974 | Pasta with cream sauce and seafood, restaurant | 22.974 |
| 58146442 | Pasta with cream sauce and seafood, home recipe | 24.889 | Pasta with cream sauce and seafood, home recipe | 24.889 |
| 58146443 | Pasta with cream sauce and seafood, ready-to-heat | 24.348 | Pasta with cream sauce and seafood, ready-to-heat | 24.348 |
| 58146451 | Pasta with cream sauce, seafood, and added vegetables, restaurant | 22.974 | Pasta with cream sauce, seafood, and added vegetables, restaurant | 22.974 |
| 58146452 | Pasta with cream sauce, seafood, and added vegetables, home recipe | 24.889 | Pasta with cream sauce, seafood, and added vegetables, home recipe | 24.889 |
| 58146453 | Pasta with cream sauce, seafood, and added vegetables, ready-to-heat | 24.348 | Pasta with cream sauce, seafood, and added vegetables, ready-to-heat | 24.348 |
| 58146601 |  |  | Pasta, whole grain, with tomato-based sauce, restaurant | 23.053 |
| 58146602 |  |  | Pasta, whole grain, with tomato-based sauce, home recipe | 24.889 |
| 58146603 |  |  | Pasta, whole grain, with tomato-based sauce, ready-to-heat | 24.348 |
| 58146611 |  |  | Pasta, whole grain, with tomato-based sauce and added vegetables, restaurant | 23.053 |
| 58146612 |  |  | Pasta, whole grain, with tomato-based sauce and added vegetables, home recipe | 24.889 |
| 58146613 |  |  | Pasta, whole grain, with tomato-based sauce and added vegetables, ready-to-heat | 24.348 |
| 58146621 |  |  | Pasta, whole grain, with tomato-based sauce and meat, restaurant | 23.053 |
| 58146622 |  |  | Pasta, whole grain, with tomato-based sauce and meat, home recipe | 24.889 |
| 58146623 |  |  | Pasta, whole grain, with tomato-based sauce and meat, ready-to-heat | 24.348 |
| 58146631 |  |  | Pasta, whole grain, with tomato-based sauce, meat, and added vegetables, restaurant | 23.053 |
| 58146632 |  |  | Pasta, whole grain, with tomato-based sauce, meat, and added vegetables, home recipe | 24.889 |
| 58146633 |  |  | Pasta, whole grain, with tomato-based sauce, meat, and added vegetables, ready-to-heat | 24.348 |
| 58146641 |  |  | Pasta, whole grain, with tomato-based sauce and poultry, restaurant | 23.053 |
| 58146642 |  |  | Pasta, whole grain, with tomato-based sauce and poultry, home recipe | 24.889 |
| 58146643 |  |  | Pasta, whole grain, with tomato-based sauce and poultry, ready-to-heat | 24.348 |
| 58146651 |  |  | Pasta, whole grain, with tomato-based sauce, poultry, and added vegetables, restaurant | 23.053 |
| 58146652 |  |  | Pasta, whole grain, with tomato-based sauce, poultry, and added vegetables, home recipe | 24.889 |
| 58146653 |  |  | Pasta, whole grain, with tomato-based sauce, poultry, and added vegetables, ready-to-heat | 24.348 |
| 58146661 |  |  | Pasta, whole grain, with tomato-based sauce and seafood, restaurant | 23.053 |
| 58146662 |  |  | Pasta, whole grain, with tomato-based sauce and seafood, home recipe | 24.889 |
| 58146663 |  |  | Pasta, whole grain, with tomato-based sauce and seafood, ready-to-heat | 24.348 |
| 58146671 |  |  | Pasta, whole grain, with tomato-based sauce, seafood, and added vegetables, restaurant | 23.053 |
| 58146672 |  |  | Pasta, whole grain, with tomato-based sauce, seafood, and added vegetables, home recipe | 24.889 |
| 58146673 |  |  | Pasta, whole grain, with tomato-based sauce, seafood, and added vegetables, ready-to-heat | 24.348 |
| 58146681 |  |  | Pasta, whole grain, with cream sauce, restaurant | 22.974 |
| 58146682 |  |  | Pasta, whole grain, with cream sauce, home recipe | 24.889 |
| 58146683 |  |  | Pasta, whole grain, with cream sauce, ready-to-heat | 24.348 |
| 58146691 |  |  | Pasta, whole grain, with cream sauce, and added vegetables, restaurant | 22.974 |
| 58146692 |  |  | Pasta, whole grain, with cream sauce, and added vegetables, home recipe | 24.889 |
| 58146693 |  |  | Pasta, whole grain, with cream sauce, and added vegetables, ready-to-heat | 24.348 |
| 58146701 |  |  | Pasta, whole grain, with cream sauce and meat, restaurant | 22.974 |
| 58146702 |  |  | Pasta, whole grain, with cream sauce and meat, home recipe | 24.889 |
| 58146703 |  |  | Pasta, whole grain, with cream sauce and meat, ready-to-heat | 24.348 |
| 58146711 |  |  | Pasta, whole grain, with cream sauce, meat, and added vegetables, restaurant | 22.974 |
| 58146712 |  |  | Pasta, whole grain, with cream sauce, meat, and added vegetables, home recipe | 24.889 |
| 58146713 |  |  | Pasta, whole grain, with cream sauce, meat, and added vegetables, ready-to-heat | 24.348 |
| 58146721 |  |  | Pasta, whole grain, with cream sauce and poultry, restaurant | 22.974 |
| 58146722 |  |  | Pasta, whole grain, with cream sauce and poultry, home recipe | 24.889 |
| 58146723 |  |  | Pasta, whole grain, with cream sauce and poultry, ready-to-heat | 24.348 |
| 58146731 |  |  | Pasta, whole grain, with cream sauce, poultry, and added vegetables, restaurant | 22.974 |
| 58146732 |  |  | Pasta, whole grain, with cream sauce, poultry, and added vegetables, home recipe | 24.889 |
| 58146733 |  |  | Pasta, whole grain, with cream sauce, poultry, and added vegetables, ready-to-heat | 24.348 |
| 58146741 |  |  | Pasta, whole grain, with cream sauce and seafood, restaurant | 22.974 |
| 58146742 |  |  | Pasta, whole grain, with cream sauce and seafood, home recipe | 24.889 |
| 58146743 |  |  | Pasta, whole grain, with cream sauce and seafood, ready-to-heat | 24.348 |
| 58146751 |  |  | Pasta, whole grain, with cream sauce, seafood, and added vegetables, restaurant | 22.974 |
| 58146752 |  |  | Pasta, whole grain, with cream sauce, seafood, and added vegetables, home recipe | 24.889 |
| 58146753 |  |  | Pasta, whole grain, with cream sauce, seafood, and added vegetables, ready-to-heat | 24.348 |
| 58147110 | Pasta with tomato-based sauce and beans or lentils | 18.348 | Pasta with tomato-based sauce and beans or lentils | 18.348 |
| 58147330 | Macaroni or noodles, creamed, with cheese | 32.713 | Macaroni or noodles, creamed, with cheese | 32.713 |
| 58147340 | Macaroni or noodles, creamed, with cheese and tuna | 25.739 | Macaroni or noodles, creamed, with cheese and tuna | 25.739 |
| 58147510 | Flavored pasta | 4.987 | Flavored pasta | 4.987 |
| 58147520 | Yat Ga Mein with meat, fish, or poultry | 20.542 | Yat Ga Mein with meat, fish, or poultry | 20.542 |
| 58148110 | Macaroni or pasta salad, made with mayonnaise | 36.500 | Macaroni or pasta salad, made with mayonnaise | 36.500 |
| 58148111 | Macaroni or pasta salad, made with light mayonnaise | 36.027 | Macaroni or pasta salad, made with light mayonnaise | 36.027 |
| 58148112 | Macaroni or pasta salad, made with mayonnaise-type salad dressing | 36.144 | Macaroni or pasta salad, made with mayonnaise-type salad dressing | 36.144 |
| 58148113 | Macaroni or pasta salad, made with light mayonnaise-type salad dressing | 36.027 | Macaroni or pasta salad, made with light mayonnaise-type salad dressing | 36.027 |
| 58148114 | Macaroni or pasta salad, made with Italian dressing | 27.703 | Macaroni or pasta salad, made with Italian dressing | 27.703 |
| 58148115 | Macaroni or pasta salad, made with light Italian dressing | 27.632 | Macaroni or pasta salad, made with light Italian dressing | 27.632 |
| 58148116 | Macaroni or pasta salad, made with creamy dressing | 27.632 | Macaroni or pasta salad, made with creamy dressing | 27.632 |
| 58148117 | Macaroni or pasta salad, made with light creamy dressing | 27.562 | Macaroni or pasta salad, made with light creamy dressing | 27.562 |
| 58148118 | Macaroni or pasta salad, made with any type of fat free dressing | 35.657 | Macaroni or pasta salad, made with any type of fat free dressing | 35.657 |
| 58148120 | Macaroni or pasta salad with egg | 33.229 | Macaroni or pasta salad with egg | 33.229 |
| 58148130 | Macaroni or pasta salad with tuna | 30.902 | Macaroni or pasta salad with tuna | 30.902 |
| 58148140 | Macaroni or pasta salad with crab meat | 31.006 | Macaroni or pasta salad with crab meat | 31.006 |
| 58148150 | Macaroni or pasta salad with shrimp | 31.250 | Macaroni or pasta salad with shrimp | 31.250 |
| 58148160 | Macaroni or pasta salad with tuna and egg | 28.525 | Macaroni or pasta salad with tuna and egg | 28.525 |
| 58148170 | Macaroni or pasta salad with chicken | 31.006 | Macaroni or pasta salad with chicken | 31.006 |
| 58148180 | Macaroni or pasta salad with cheese | 31.786 | Macaroni or pasta salad with cheese | 31.786 |
| 58148550 | Macaroni or pasta salad with meat | 30.834 | Macaroni or pasta salad with meat | 30.834 |
| 58149110 | Noodle pudding | 30.991 | Noodle pudding | 30.991 |
| 58149160 | Noodle pudding, with milk | 8.534 |  |  |
| 58149210 | Somen salad with noodles, lettuce, egg, fish, and pork | 12.645 |  |  |
| 58150520 | Dukboki or Tteokbokki, Korean | 1.327 | Dukboki or Tteokbokki, Korean | 1.327 |
| 58150530 | Adobo, with rice | 0.834 | Adobo, with rice | 0.834 |
| 58155110 | Rice with chicken, Puerto Rican style | 0.693 |  |  |
| 58155310 | Paella with meat, Valenciana style | 6.745 |  |  |
| 58155320 | Seafood paella, Puerto Rican style | 4.035 | Seafood paella, Puerto Rican style | 4.035 |
| 58155410 | Soupy rice with chicken, Puerto Rican style | 0.245 | Soupy rice with chicken, Puerto Rican style | 0.245 |
| 58155510 | Soupy rice mixture with chicken and potatoes, Puerto Rican style | 2.356 | Soupy rice mixture with chicken and potatoes, Puerto Rican style | 2.356 |
| 58155610 | Rice meal fritter, Puerto Rican style | 2.509 |  |  |
| 58155910 | Rice with squid, Puerto Rican style | 0.704 | Rice with squid, Puerto Rican style | 0.704 |
| 58156110 | Fried rice, Puerto Rican style | 10.025 |  |  |
| 58156210 | Rice with vienna sausage, Puerto Rican style | 2.727 | Rice with vienna sausage, Puerto Rican style | 2.727 |
| 58156310 | Rice with Spanish sausage, Puerto Rican style | 0.777 | Rice with Spanish sausage, Puerto Rican style | 0.777 |
| 58156410 | Rice with onions, Puerto Rican style | 9.768 |  |  |
| 58157110 | Spicy rice pudding, Puerto Rican style | 1.210 |  |  |
| 58157320 |  |  | Congee, with vegetables | 5.273 |
| 58160000 | Biryani with vegetables | 10.072 | Biryani with vegetables | 10.072 |
| 58160102 |  |  | Kidney beans and rice, from fast food / restaurant | 11.337 |
| 58160110 | Rice with beans | 7.996 | Beans and white rice | 7.996 |
| 58160120 | Rice with beans and tomatoes | 6.152 | Beans and rice, with tomatoes | 6.152 |
| 58160130 | Rice with beans and chicken | 6.739 |  |  |
| 58160135 | Rice with beans and beef | 6.726 |  |  |
| 58160140 | Rice with beans and pork | 6.726 |  |  |
| 58160150 | Red beans and rice | 12.670 | Kidney beans and white rice | 12.670 |
| 58160160 | Hopping John | 27.666 |  |  |
| 58160550 | Rice, white, with dark green vegetables, NS as to fat added in cooking | 1.882 | Rice, white, with dark green vegetables, NS as to fat | 1.882 |
| 58160560 | Rice, white, with dark green vegetables, fat not added in cooking | 1.923 | Rice, white, with dark green vegetables, no added fat | 1.923 |
| 58160570 | Rice, white, with dark green vegetables, fat added in cooking | 1.882 | Rice, white, with dark green vegetables, fat added | 1.882 |
| 58160610 | Rice, white, with dark green vegetables and tomatoes and/or tomato-based sauce, NS as to fat added in cooking | 0.882 | Rice, white, with dark green vegetables and tomatoes and/or tomato-based sauce, NS as to fat | 0.882 |
| 58160620 | Rice, white, with dark green vegetables and tomatoes and/or tomato-based sauce, fat not added in cooking | 0.900 | Rice, white, with dark green vegetables and tomatoes and/or tomato-based sauce, no added fat | 0.900 |
| 58160630 | Rice, white, with dark green vegetables and tomatoes and/or tomato-based sauce, fat added in cooking | 0.882 | Rice, white, with dark green vegetables and tomatoes and/or tomato-based sauce, fat added | 0.882 |
| 58160640 | Rice, white, with carrots and dark green vegetables, NS as to fat added in cooking | 0.940 | Rice, white, with carrots and dark green vegetables, NS as to fat | 0.940 |
| 58160650 | Rice, white, with carrots and dark green vegetables, fat not added in cooking | 0.960 | Rice, white, with carrots and dark green vegetables, no added fat | 0.960 |
| 58160660 | Rice, white, with carrots and dark green vegetables, fat added in cooking | 0.940 | Rice, white, with carrots and dark green vegetables, fat added | 0.940 |
| 58160670 | Rice, white, with carrots, dark green vegetables, and tomatoes and/or tomato-based sauce, NS as to fat added in cooking | 0.601 | Rice, white, with carrots, dark green vegetables, and tomatoes and/or tomato-based sauce, NS as to fat | 0.601 |
| 58160680 | Rice, white, with carrots, dark green vegetables, and tomatoes and/or tomato-based sauce, fat not added in cooking | 0.613 | Rice, white, with carrots, dark green vegetables, and tomatoes and/or tomato-based sauce, no added fat | 0.613 |
| 58160690 | Rice, white, with carrots, dark green vegetables, and tomatoes and/or tomato-based sauce, fat added in cooking | 0.601 | Rice, white, with carrots, dark green vegetables, and tomatoes and/or tomato-based sauce, fat added | 0.601 |
| 58160700 | Rice, white, with other vegetables, NS as to fat added in cooking | 8.759 | Rice, white, with other vegetables, NS as to fat | 8.759 |
| 58160710 | Rice, white, with other vegetables, fat not added in cooking | 8.947 | Rice, white, with other vegetables, no added fat | 8.947 |
| 58160720 | Rice, white, with other vegetables, fat added in cooking | 8.759 | Rice, white, with other vegetables, fat added | 8.759 |
| 58160800 | Rice, white, with lentils, NS as to fat added in cooking | 7.744 | Rice, white, with lentils, NS as to fat | 7.744 |
| 58160805 | Rice, white, with lentils, fat added in cooking | 7.744 | Rice, white, with lentils, fat added | 7.744 |
| 58160810 | Rice, white, with lentils, fat not added in cooking | 8.100 | Rice, white, with lentils, no added fat | 8.100 |
| 58161320 | Rice, brown, with beans | 7.320 | Beans and brown rice | 7.320 |
| 58161321 |  |  | Kidney beans and brown rice | 11.700 |
| 58161325 | Rice, brown, with beans and tomatoes | 5.744 |  |  |
| 58161470 | Rice, brown, with dark green vegetables, NS as to fat added in cooking | 1.607 | Rice, brown, with dark green vegetables, NS as to fat | 1.607 |
| 58161472 | Rice, brown, with dark green vegetables, fat not added in cooking | 1.636 | Rice, brown, with dark green vegetables, no added fat | 1.636 |
| 58161474 | Rice, brown, with dark green vegetables, fat added in cooking | 1.607 | Rice, brown, with dark green vegetables, fat added | 1.607 |
| 58161490 | Rice, brown, with dark green vegetables and tomatoes and/or tomato-based sauce, NS as to fat added in cooking | 0.760 | Rice, brown, with dark green vegetables and tomatoes and/or tomato-based sauce, NS as to fat | 0.760 |
| 58161492 | Rice, brown, with dark green vegetables and tomatoes and/or tomato-based sauce, fat not added in cooking | 0.773 | Rice, brown, with dark green vegetables and tomatoes and/or tomato-based sauce, no added fat | 0.773 |
| 58161494 | Rice, brown, with dark green vegetables and tomatoes and/or tomato-based sauce, fat added in cooking | 0.760 | Rice, brown, with dark green vegetables and tomatoes and/or tomato-based sauce, fat added | 0.760 |
| 58161500 | Rice, brown, with carrots and dark green vegetables, NS as to fat added in cooking | 0.802 | Rice, brown, with carrots and dark green vegetables, NS as to fat | 0.802 |
| 58161502 | Rice, brown, with carrots and dark green vegetables, fat not added in cooking | 0.817 | Rice, brown, with carrots and dark green vegetables, no added fat | 0.817 |
| 58161504 | Rice, brown, with carrots and dark green vegetables, fat added in cooking | 0.802 | Rice, brown, with carrots and dark green vegetables, fat added | 0.802 |
| 58161510 | Grape leaves stuffed with rice | 38.211 | Grape leaves stuffed with rice | 38.211 |
| 58161520 | Rice, brown, with carrots, dark green vegetables, and tomatoes and/or tomato-based sauce, NS as to fat added in cooking | 0.516 | Rice, brown, with carrots, dark green vegetables, and tomatoes and/or tomato-based sauce, NS as to fat | 0.516 |
| 58161522 | Rice, brown, with carrots, dark green vegetables, and tomatoes and/or tomato-based sauce, fat not added in cooking | 0.525 | Rice, brown, with carrots, dark green vegetables, and tomatoes and/or tomato-based sauce, no added fat | 0.525 |
| 58161524 | Rice, brown, with carrots, dark green vegetables, and tomatoes and/or tomato-based sauce, fat added in cooking | 0.516 | Rice, brown, with carrots, dark green vegetables, and tomatoes and/or tomato-based sauce, fat added | 0.516 |
| 58161530 | Rice, brown, with other vegetables, NS as to fat added in cooking | 7.477 | Rice, brown, with other vegetables, NS as to fat | 7.477 |
| 58161532 | Rice, brown, with other vegetables, fat not added in cooking | 7.614 | Rice, brown, with other vegetables, no added fat | 7.614 |
| 58161534 | Rice, brown, with other vegetables, fat added in cooking | 7.477 | Rice, brown, with other vegetables, fat added | 7.477 |
| 58161710 | Rice croquette | 3.487 | Rice croquette | 3.487 |
| 58162090 | Stuffed pepper, with meat | 0.966 | Stuffed pepper, with meat | 0.966 |
| 58162120 | Stuffed pepper, with rice, meatless | 0.909 | Stuffed pepper, with rice, meatless | 0.909 |
| 58162130 | Stuffed tomato, with rice and meat | 4.215 | Stuffed tomato, with rice and meat | 4.215 |
| 58162140 | Stuffed tomato, with rice, meatless | 7.890 | Stuffed tomato, with rice, meatless | 7.890 |
| 58163130 | Dirty rice | 14.469 | Dirty rice | 14.469 |
| 58163310 | Flavored rice mixture | 2.287 | Flavored rice mixture | 2.287 |
| 58163360 | Flavored rice, brown and wild | 0.068 | Flavored rice, brown and wild | 0.068 |
| 58163400 | Flavored rice and pasta mixture, reduced sodium | 6.473 | Flavored rice and pasta mixture, reduced sodium | 6.473 |
| 58163410 | Spanish rice, fat added in cooking | 8.761 | Spanish rice, fat added | 8.761 |
| 58163420 | Spanish rice, fat not added in cooking | 8.879 | Spanish rice, no added fat | 8.879 |
| 58163430 | Spanish rice, NS as to fat added in cooking | 8.762 | Spanish rice, NS as to fat | 8.762 |
| 58163450 | Spanish rice with ground beef | 6.595 | Spanish rice with ground beef | 6.595 |
| 58163510 | Rice dressing | 7.077 | Rice dressing | 7.077 |
| 58174000 | Upma, Indian breakfast dish | 25.602 | Upma | 25.602 |
| 58175110 | Tabbouleh | 8.334 | Tabbouleh | 8.334 |
| 58200100 | Wrap sandwich, filled with meat, poultry, or fish, vegetables, and rice | 5.895 |  |  |
| 58200200 | Wrap sandwich, filled with vegetables and rice | 6.071 |  |  |
| 58200210 |  |  | Vegetable sandwich on white | 9.764 |
| 58200220 |  |  | Vegetable sandwich on white, with cheese | 8.649 |
| 58200230 |  |  | Vegetable sandwich on wheat | 27.433 |
| 58200240 |  |  | Vegetable sandwich on wheat, with cheese | 24.302 |
| 58200250 | Wrap sandwich, filled with vegetables | 10.101 | Vegetable sandwich wrap | 10.101 |
| 58200300 | Wrap sandwich, filled with meat, poultry, or fish, vegetables, rice, and cheese | 5.942 |  |  |
| 58201000 | Jelly sandwich, regular jelly, NFS | 28.800 | Jelly sandwich, NFS | 28.800 |
| 58201005 | Jelly sandwich, regular jelly, on white bread | 28.800 | Jelly sandwich, on white bread | 28.800 |
| 58201015 | Jelly sandwich, regular jelly, on wheat bread | 28.800 | Jelly sandwich, on wheat bread | 28.800 |
| 58201025 | Jelly sandwich, regular jelly, on whole wheat bread | 28.800 |  |  |
| 58201035 | Jelly sandwich, reduced sugar jelly, on white bread | 28.800 |  |  |
| 58201045 | Jelly sandwich, reduced sugar jelly, on wheat bread | 28.800 |  |  |
| 58201055 | Jelly sandwich, reduced sugar jelly, on whole wheat bread | 28.800 |  |  |
| 58301020 | Lasagna with cheese and sauce, diet frozen meal | 19.623 |  |  |
| 58301030 | Veal lasagna, diet frozen meal | 13.992 |  |  |
| 58301050 | Lasagna with cheese and meat sauce, diet frozen meal | 12.691 |  |  |
| 58301110 | Vegetable lasagna, frozen meal | 16.856 |  |  |
| 58301150 | Zucchini lasagna, diet frozen meal | 16.011 |  |  |
| 58302000 | Macaroni and cheese, diet frozen meal | 19.700 |  |  |
| 58302050 | Beef and noodles with meat sauce and cheese, diet frozen meal | 15.571 |  |  |
| 58302080 | Noodles with vegetables in tomato-based sauce, diet frozen meal | 9.719 | Noodles with vegetables in tomato-based sauce, diet frozen meal | 9.719 |
| 58303100 | Rice, with broccoli, cheese sauce, frozen side dish | 4.417 |  |  |
| 58304010 | Spaghetti and meatballs dinner, NFS, frozen meal | 17.247 | Spaghetti and meatballs dinner, NFS, frozen meal | 17.247 |
| 58304020 | Spaghetti and meatballs with tomato sauce, sliced apples, bread, frozen meal | 17.676 |  |  |
| 58304050 | Spaghetti with meat and mushroom sauce, diet frozen meal | 25.691 |  |  |
| 58304200 | Ravioli, cheese-filled, with tomato sauce, diet frozen meal | 11.794 |  |  |
| 58304220 | Rigatoni with meat sauce and cheese, diet frozen meal | 7.604 |  |  |
| 58304250 | Manicotti, cheese-filled, with tomato sauce, diet frozen meal | 12.615 |  |  |
| 58304300 | Cannelloni, cheese-filled, with tomato sauce, diet frozen meal | 11.263 |  |  |
| 58304400 | Linguini with vegetables and seafood in white wine sauce, diet frozen meal | 13.832 |  |  |
| 58305250 | Pasta with vegetable and cheese sauce, diet frozen meal | 15.024 |  |  |
| 58306020 | Beef enchilada, chili gravy, rice, refried beans, frozen meal | 9.196 |  |  |
| 58306100 | Chicken enchilada, diet frozen meal | 3.880 |  |  |
| 58306200 | Chicken fajitas, diet frozen meal | 26.079 |  |  |
| 58306500 | Chicken burritos, diet frozen meal | 24.201 |  |  |
| 58310210 | Sausage and french toast, frozen meal | 12.754 |  |  |
| 58310310 | Pancakes and sausage, frozen meal | 11.341 | Pancakes and sausage, frozen meal | 11.341 |
| 58402100 | Beef noodle soup, home recipe | 7.750 |  |  |
| 58403010 |  |  | Soup, chicken noodle, canned | 7.091 |
| 58403040 | Chicken or turkey noodle soup, home recipe | 7.742 | Soup, chicken noodle | 7.742 |
| 58403100 | Noodle and potato soup, Puerto Rican style | 3.627 |  |  |
| 58404030 | Chicken or turkey rice soup, home recipe | 5.717 |  |  |
| 58404500 | Matzo ball soup | 9.148 | Soup, Matzo ball | 9.148 |
| 58407038 |  |  | Ramen bowl with beef | 28.224 |
| 58407042 |  |  | Ramen bowl with chicken | 28.224 |
| 58407046 |  |  | Ramen bowl with fish | 28.224 |
| 58407052 |  |  | Ramen bowl, vegetarian | 28.224 |
| 58408500 | Noodle soup with vegetables, Asian style | 3.098 |  |  |
| 58409000 | Noodle soup, with fish ball, shrimp, and dark green leafy vegetable | 1.846 |  |  |
| 58421000 | Sopa seca, Mexican style, NFS | 18.641 |  |  |
| 58421010 | Sopa Seca de Fideo, Mexican style, made with dry noodles, home recipe | 24.186 |  |  |
| 58421020 | Sopa de Fideo Aguada, Mexican style noodle soup, home recipe | 4.054 | Soup, sopa de fideo aguada | 4.054 |
| 58421060 | Sopa seca de arroz, home recipe, Mexican style | 14.741 |  |  |
| 58421080 | Sopa de tortilla, Mexican style tortilla soup, home recipe | 5.985 | Soup, tortilla | 5.985 |
| 59003000 | Meat substitute, cereal- and vegetable protein-based, fried | 38.400 | Meat substitute, cereal- and vegetable protein-based, fried | 38.400 |
| 61113500 | Lemon pie filling | 2.774 | Lemon pie filling | 2.774 |
| 62101050 | Fruit mixture, dried | 0.840 | Dried, fruit, NFS | 0.840 |
| 62105000 | Blueberries, dried | 5.000 | Blueberries, dried | 5.000 |
| 62107100 | Banana flakes, dehydrated | 29.000 |  |  |
| 62107200 | Banana chips | 29.000 | Banana chips | 29.000 |
| 62119100 | Pear, dried, uncooked | 5.600 | Pear, dried | 5.600 |
| 62119200 | Pear, dried, cooked, NS as to sweetened or unsweetened; sweetened, NS as to type of sweetener | 5.000 |  |  |
| 62119220 | Pear, dried, cooked, unsweetened | 5.600 |  |  |
| 62119230 | Pear, dried, cooked, with sugar | 5.000 |  |  |
| 63100100 | Fruit, NS as to type | 4.000 | Fruit, NFS | 4.000 |
| 63107010 | Banana, raw | 10.000 | Banana, raw | 10.000 |
| 63107050 | Banana, white, Guineo blanco maduro | 10.000 |  |  |
| 63107070 | Banana, Chinese, raw | 10.000 |  |  |
| 63107080 | Banana, red, Guineo morado | 10.000 |  |  |
| 63107090 | Banana, red, fried | 9.454 |  |  |
| 63107110 | Banana, baked | 9.031 |  |  |
| 63107210 | Banana, ripe, fried | 9.454 |  |  |
| 63107310 | Banana, ripe, boiled | 10.000 |  |  |
| 63107410 | Banana, batter-dipped, fried | 15.398 | Banana, baked | 15.398 |
| 63109015 |  |  | Melon, frozen | 2.700 |
| 63126010 | Juneberry, raw | 5.000 |  |  |
| 63137010 | Pear, raw | 5.600 | Pear, raw | 5.600 |
| 63137050 | Pear, Japanese, raw | 5.600 | Pear, Asian, raw | 5.600 |
| 63137110 | Pear, cooked or canned, NS as to sweetened or unsweetened; sweetened, NS as to type of sweetener | 5.600 | Pear, canned, NFS | 5.600 |
| 63137120 | Pear, cooked or canned, unsweetened, water pack | 5.600 |  |  |
| 63137130 | Pear, cooked or canned, in heavy syrup | 5.600 |  |  |
| 63137140 | Pear, cooked or canned, in light syrup | 5.600 | Pear, canned, in syrup | 5.600 |
| 63137150 | Pear, cooked or canned, drained solids | 5.600 |  |  |
| 63137170 | Pear, cooked or canned, juice pack | 5.600 | Pear, canned, juice pack | 5.600 |
| 63149010 | Watermelon, raw | 8.100 | Watermelon, raw | 8.100 |
| 63200100 |  |  | Berries, NFS | 1.500 |
| 63200110 |  |  | Berries, frozen | 2.525 |
| 63200200 | Berries, frozen, NFS | 5.000 |  |  |
| 63203010 | Blueberries, raw | 5.000 | Blueberries, raw | 5.000 |
| 63203110 | Blueberries, cooked or canned, NS as to sweetened or unsweetened; sweetened, NS as to type of sweetener | 5.000 | Bluberries, canned | 5.000 |
| 63203120 | Blueberries, cooked or canned, unsweetened, water pack | 3.350 |  |  |
| 63203125 | Blueberries, cooked or canned, in light syrup | 5.000 |  |  |
| 63203130 | Blueberries, cooked or canned, in heavy syrup | 5.000 |  |  |
| 63203550 | Blueberries, frozen, sweetened | 5.000 |  |  |
| 63203570 | Blueberries, frozen, NS as to sweetened or unsweetened | 5.000 |  |  |
| 63203600 | Blueberries, frozen, unsweetened | 5.000 | Blueberries, frozen | 5.000 |
| 63203700 | Blueberry pie filling | 5.000 | Blueberry pie filling | 5.000 |
| 63214000 | Huckleberries, raw | 5.000 |  |  |
| 63219000 | Raspberries, raw, NS as to color | 5.100 | Raspberries, raw | 5.100 |
| 63219010 | Raspberries, black, raw | 5.100 |  |  |
| 63219020 | Raspberries, red, raw | 5.100 |  |  |
| 63219110 | Raspberries, cooked or canned, NS as to sweetened or unsweetened; sweetened, NS as to type of sweetener | 5.100 |  |  |
| 63219120 | Raspberries, cooked or canned, unsweetened, water pack | 3.417 |  |  |
| 63219130 | Raspberries, cooked or canned, in heavy syrup | 5.100 |  |  |
| 63219600 | Raspberries, frozen, NS as to added sweetener | 5.100 |  |  |
| 63219610 | Raspberries, frozen, unsweetened | 5.100 | Raspberries, frozen | 5.100 |
| 63219620 | Raspberries, frozen, with sugar | 5.100 |  |  |
| 63301010 | Ambrosia | 2.129 | Ambrosia | 2.129 |
| 63307100 | Cranberry-raspberry Sauce | 1.020 |  |  |
| 63311000 | Fruit salad, fresh or raw, excluding citrus fruits, no dressing | 3.144 | Fruit salad, fresh or raw, excluding citrus fruits, no dressing | 3.144 |
| 63311050 | Fruit salad, fresh or raw, including citrus fruits, no dressing | 2.480 | Fruit salad, fresh or raw, including citrus fruits, no dressing | 2.480 |
| 63311080 | Fruit cocktail or mix, frozen | 0.510 |  |  |
| 63311140 |  |  | Fruit cocktail, canned, in syrup | 5.152 |
| 63311170 |  |  | Fruit cocktail, canned, juice pack | 4.928 |
| 63320100 | Fruit salad, Puerto Rican style | 2.931 |  |  |
| 63401990 | Banana, chocolate-covered with nuts | 7.062 |  |  |
| 63402010 | Banana whip | 5.248 |  |  |
| 63402045 | Fried dwarf banana, Puerto Rican style | 9.177 |  |  |
| 63402050 | Fried dwarf banana with cheese, Puerto Rican style | 8.462 |  |  |
| 63402950 | Fruit salad, excluding citrus fruits, with salad dressing or mayonnaise | 2.031 | Fruit salad, excluding citrus fruits, with salad dressing or mayonnaise | 2.031 |
| 63402960 | Fruit salad, excluding citrus fruits, with whipped cream | 2.233 | Fruit salad, excluding citrus fruits, with whipped cream | 2.233 |
| 63402970 | Fruit salad, excluding citrus fruits, with nondairy whipped topping | 2.336 | Fruit salad, excluding citrus fruits, with nondairy whipped topping | 2.336 |
| 63402980 | Fruit salad, excluding citrus fruits, with marshmallows | 1.912 | Fruit salad, excluding citrus fruits, with marshmallows | 1.912 |
| 63402990 | Fruit salad, including citrus fruits, with pudding | 1.921 | Fruit salad, including citrus fruits, with pudding | 1.921 |
| 63403000 | Fruit salad, excluding citrus fruits, with pudding | 1.958 | Fruit salad, excluding citrus fruits, with pudding | 1.958 |
| 63403010 | Fruit salad, including citrus fruits, with salad dressing or mayonnaise | 1.990 | Fruit salad, including citrus fruits, with salad dressing or mayonnaise | 1.990 |
| 63403020 | Fruit salad, including citrus fruit, with whipped cream | 2.184 | Fruit salad, including citrus fruit, with whipped cream | 2.184 |
| 63403030 | Fruit salad, including citrus fruits, with nondairy whipped topping | 2.283 | Fruit salad, including citrus fruits, with nondairy whipped topping | 2.283 |
| 63403040 | Fruit salad, including citrus fruits, with marshmallows | 1.876 | Fruit salad, including citrus fruits, with marshmallows | 1.876 |
| 63409010 | Guacamole, NFS | 1.283 | Guacamole, NFS | 1.283 |
| 63409015 | Guacamole with tomatoes | 1.183 | Guacamole with tomatoes | 1.183 |
| 63409020 | Chutney | 4.219 | Chutney | 4.219 |
| 63413010 | Pineapple salad with dressing | 0.014 | Pineapple salad with dressing | 0.014 |
| 63415100 |  |  | Soup, fruit | 1.620 |
| 64104610 |  |  | Blueberry juice | 3.250 |
| 64133100 | Watermelon juice, 100% | 8.100 | Watermelon juice, 100% | 8.100 |
| 64134015 | Fruit smoothie, with whole fruit, no dairy | 2.918 | Fruit smoothie, with whole fruit, no dairy | 2.918 |
| 64134020 | Fruit smoothie, with whole fruit, no dairy, added protein | 2.708 | Fruit smoothie, with whole fruit, no dairy, added protein | 2.708 |
| 64134025 |  |  | Fruit smoothie, with whole fruit, non-dairy | 2.174 |
| 64134030 | Fruit smoothie juice drink, no dairy | 2.061 | Fruit smoothie juice drink, no dairy | 2.061 |
| 64134100 | Fruit smoothie, light | 2.125 | Fruit smoothie, light | 2.125 |
| 64134200 |  |  | Fruit smoothie, bottled | 10.000 |
| 64201500 | Banana nectar | 3.500 | Banana nectar | 3.500 |
| 64215010 |  |  | Pear nectar | 5.600 |
| 67109000 | Pears, baby food, NS as to strained or junior | 5.600 |  |  |
| 67109010 | Pears, baby food, strained | 5.600 | Baby Toddler pears, Stage 1 | 5.600 |
| 67109020 | Pears, baby food, junior | 5.600 |  |  |
| 67109030 | Pears, baby food, toddler | 5.600 |  |  |
| 67113000 | Apples and pears, baby food, NS as to strained or junior | 2.800 |  |  |
| 67113010 | Apples and pears, baby food, strained | 2.800 |  |  |
| 67113020 | Apples and pears, baby food, junior | 2.800 |  |  |
| 71305020 | Potato, scalloped, from fast food or restaurant | 1.070 | Potato, scalloped, from fast food or restaurant | 1.070 |
| 71305030 | Potato, scalloped, from fresh | 0.710 | Potato, scalloped, from fresh | 0.710 |
| 71305040 | Potato, scalloped, from fresh, with meat | 0.674 | Potato, scalloped, from fresh, with meat | 0.674 |
| 71305050 | Potato, scalloped, from dry mix | 1.104 | Potato, scalloped, from dry mix | 1.104 |
| 71305060 | Potato, scalloped, from dry mix, with meat | 1.065 | Potato, scalloped, from dry mix, with meat | 1.065 |
| 71305070 | Potato, scalloped, ready-to-heat | 1.085 | Potato, scalloped, ready-to-heat | 1.085 |
| 71305080 | Potato, scalloped, ready-to-heat, with meat | 1.033 | Potato, scalloped, ready-to-heat, with meat | 1.033 |
| 71403500 | Potato, home fries, with vegetables | 10.301 | Potato, home fries, with vegetables | 10.301 |
| 71503010 | Potato patty | 9.146 | Potato patty | 9.146 |
| 71507035 | Potato, baked, peel not eaten, with vegetables | 0.447 | Potato, baked, peel not eaten, with vegetables | 0.447 |
| 71508035 | Potato, baked, peel eaten, with vegetables | 0.447 | Potato, baked, peel eaten, with vegetables | 0.447 |
| 71600950 | Potato salad with egg, from restaurant | 1.716 | Potato salad with egg, from restaurant | 1.716 |
| 71601015 | Potato salad with egg, made with light mayonnaise | 1.648 | Potato salad with egg, made with light mayonnaise | 1.648 |
| 71601020 | Potato salad with egg, made with mayonnaise-type salad dressing | 1.653 | Potato salad with egg, made with mayonnaise-type salad dressing | 1.653 |
| 71601025 | Potato salad with egg, made with light mayonnaise-type salad dressing | 1.648 | Potato salad with egg, made with light mayonnaise-type salad dressing | 1.648 |
| 71601030 | Potato salad with egg, made with creamy dressing | 1.730 | Potato salad with egg, made with creamy dressing | 1.730 |
| 71601035 | Potato salad with egg, made with light creamy dressing | 1.725 | Potato salad with egg, made with light creamy dressing | 1.725 |
| 71601040 | Potato salad with egg, made with Italian dressing | 1.735 | Potato salad with egg, made with Italian dressing | 1.735 |
| 71601045 | Potato salad with egg, made with light Italian dressing | 1.730 | Potato salad with egg, made with light Italian dressing | 1.730 |
| 71601050 | Potato salad with egg, made with any type of fat free dressing | 1.633 | Potato salad with egg, made with any type of fat free dressing | 1.633 |
| 71602010 | Potato salad, German style | 7.126 | Potato salad, German style | 7.126 |
| 71602950 | Potato salad, from restaurant | 1.953 | Potato salad, from restaurant | 1.953 |
| 71603010 | Potato salad, made with mayonnaise | 1.891 | Potato salad, made with mayonnaise | 1.891 |
| 71603015 | Potato salad, made with light mayonnaise | 1.866 | Potato salad, made with light mayonnaise | 1.866 |
| 71603020 | Potato salad, made with mayonnaise-type salad dressing | 1.872 | Potato salad, made with mayonnaise-type salad dressing | 1.872 |
| 71603025 | Potato salad, made with light mayonnaise-type salad dressing | 1.866 | Potato salad, made with light mayonnaise-type salad dressing | 1.866 |
| 71603030 | Potato salad, made with creamy dressing | 1.971 | Potato salad, made with creamy dressing | 1.971 |
| 71603035 | Potato salad, made with light creamy dressing | 1.964 | Potato salad, made with light creamy dressing | 1.964 |
| 71603040 | Potato salad, made with Italian dressing | 1.978 | Potato salad, made with Italian dressing | 1.978 |
| 71603045 | Potato salad, made with light Italian dressing | 1.971 | Potato salad, made with light Italian dressing | 1.971 |
| 71603050 | Potato salad, made with any type of fat free dressing | 1.846 | Potato salad, made with any type of fat free dressing | 1.846 |
| 71701000 | Potato pancake | 6.516 | Potato pancake | 6.516 |
| 71701500 | Lefse (Norwegian) | 11.925 | Lefse | 11.925 |
| 71703990 | Stewed potatoes | 6.312 | Stewed potatoes | 6.312 |
| 71704000 | Stewed potatoes with tomatoes | 6.485 | Stewed potatoes with tomatoes | 6.485 |
| 71801000 |  |  | Soup, potato | 1.427 |
| 71801005 |  |  | Soup, potato with meat | 1.427 |
| 71801100 | Potato and cheese soup | 4.744 |  |  |
| 71803010 | Potato chowder | 3.537 |  |  |
| 71905120 | Plantain, ripe, rolled in flour, fried | 2.383 |  |  |
| 71910110 | Green banana, cooked in salt water | 9.940 |  |  |
| 71910210 | Green banana, fried | 9.454 |  |  |
| 71910310 | Pickled green bananas, Puerto Rican style | 26.717 |  |  |
| 71931010 | Cassava with creole sauce, Puerto Rican style | 23.367 |  |  |
| 71945020 | Yam buns; Puerto Rican style | 3.279 |  |  |
| 72113100 | Dandelion greens, raw | 243.000 | Dandelion greens, raw | 243.000 |
| 72113200 | Dandelion greens, cooked, NS as to fat added in cooking | 155.515 |  |  |
| 72113210 | Dandelion greens, cooked, fat not added in cooking | 162.453 |  |  |
| 72113220 | Dandelion greens, cooked, fat added in cooking, NS as to type of fat | 155.515 | Dandelion greens, cooked | 155.515 |
| 72113230 | Dandelion greens, cooked, made with oil | 155.778 |  |  |
| 72113240 | Dandelion greens, cooked, made with butter | 155.515 |  |  |
| 72113250 | Dandelion greens, cooked, made with margarine | 155.456 |  |  |
| 72116000 | Romaine lettuce, raw | 0.167 | Romaine lettuce, raw | 0.167 |
| 72116150 | Caesar salad, with romaine, no dressing | 2.595 | Caesar salad, with romaine, no dressing | 2.595 |
| 72118200 | Greens, cooked, NS as to form, NS as to fat added in cooking | 1.378 |  |  |
| 72118201 | Greens, cooked, from fresh, NS as to fat added in cooking | 1.378 |  |  |
| 72118202 | Greens, cooked, from frozen, NS as to fat added in cooking | 1.378 |  |  |
| 72118203 | Greens, cooked, from canned, NS as to fat added in cooking | 1.378 |  |  |
| 72118210 | Greens, cooked, NS as to form, fat not added in cooking | 1.420 |  |  |
| 72118211 | Greens, cooked, from fresh, fat not added in cooking | 1.420 | Greens, fresh, cooked, no added fat | 1.420 |
| 72118212 | Greens, cooked, from frozen, fat not added in cooking | 1.420 | Greens, frozen, cooked, no added fat | 1.420 |
| 72118213 | Greens, cooked, from canned, fat not added in cooking | 1.420 |  |  |
| 72118220 | Greens, cooked, NS as to form, fat added in cooking, NS as to type of fat | 1.378 | Greens, NS as to form, cooked | 1.378 |
| 72118221 | Greens, cooked, from fresh, fat added in cooking, NS as to type of fat | 1.378 | Greens, fresh, cooked, fat added | 1.378 |
| 72118222 | Greens, cooked, from frozen, fat added in cooking, NS as to type of fat | 1.378 | Greens, frozen, cooked, fat added | 1.378 |
| 72118223 | Greens, cooked, from canned, fat added in cooking, NS as to type of fat | 1.378 | Greens, canned, cooked | 1.378 |
| 72118224 | Greens, cooked, NS as to form, made with oil | 1.379 |  |  |
| 72118225 | Greens, cooked, NS as to form, made with butter | 1.378 |  |  |
| 72118226 | Greens, cooked, NS as to form, made with margarine | 1.377 |  |  |
| 72118227 | Greens, cooked, from fresh, made with oil | 1.379 |  |  |
| 72118228 | Greens, cooked, from fresh, made with butter | 1.378 |  |  |
| 72118229 | Greens, cooked, from fresh, made with margarine | 1.377 |  |  |
| 72118230 | Greens, cooked, from frozen, made with oil | 1.379 |  |  |
| 72118231 | Greens, cooked, from frozen, made with butter | 1.378 |  |  |
| 72118232 | Greens, cooked, from frozen, made with margarine | 1.323 |  |  |
| 72118233 | Greens, cooked, from canned, made with oil | 1.379 |  |  |
| 72118234 | Greens, cooked, from canned, made with butter | 1.378 |  |  |
| 72118235 | Greens, cooked, from canned, made with margarine | 1.377 |  |  |
| 72118300 | Chamnamul, cooked, NS as to fat added in cooking | 1.017 |  |  |
| 72118305 | Chamnamul, cooked, fat not added in cooking | 1.068 |  |  |
| 72118310 | Chamnamul, cooked, fat added in cooking | 1.017 |  |  |
| 72119190 | Kale, raw | 5.100 | Kale, raw | 5.100 |
| 72119200 | Kale, cooked, NS as to form, NS as to fat added in cooking | 4.885 |  |  |
| 72119201 | Kale, cooked, from fresh, NS as to fat added in cooking | 4.885 |  |  |
| 72119202 | Kale, cooked, from frozen, NS as to fat added in cooking | 4.885 |  |  |
| 72119203 | Kale, cooked, from canned, NS as to fat added in cooking | 4.885 |  |  |
| 72119210 | Kale, cooked, NS as to form, fat not added in cooking | 5.061 |  |  |
| 72119211 | Kale, cooked, from fresh, fat not added in cooking | 5.061 | Kale, fresh, cooked, no added fat | 5.061 |
| 72119212 | Kale, cooked, from frozen, fat not added in cooking | 5.061 | Kale, frozen, cooked, no added fat | 5.061 |
| 72119213 | Kale, cooked, from canned, fat not added in cooking | 5.061 |  |  |
| 72119220 | Kale, cooked, NS as to form, fat added in cooking, NS as to type of fat | 4.885 | Kale, NS as to form, cooked | 4.885 |
| 72119221 | Kale, cooked, from fresh, fat added in cooking, NS as to type of fat | 4.885 | Kale, fresh, cooked, fat added | 4.885 |
| 72119222 | Kale, cooked, from frozen, fat added in cooking, NS as to type of fat | 4.885 | Kale, frozen, cooked, fat added | 4.885 |
| 72119223 | Kale, cooked, from canned, fat added in cooking, NS as to type of fat | 4.885 |  |  |
| 72119224 | Kale, cooked, NS as to form, made with oil | 4.891 |  |  |
| 72119225 | Kale, cooked, NS as to form, made with butter | 4.885 |  |  |
| 72119226 | Kale, cooked, NS as to form, made with margarine | 4.883 |  |  |
| 72119227 | Kale, cooked, from fresh, made with oil | 4.891 |  |  |
| 72119228 | Kale, cooked, from fresh, made with butter | 4.885 |  |  |
| 72119229 | Kale, cooked, from fresh, made with margarine | 4.883 |  |  |
| 72119230 | Kale, cooked, from frozen, made with oil | 4.891 |  |  |
| 72119231 | Kale, cooked, from frozen, made with butter | 4.885 |  |  |
| 72119232 | Kale, cooked, from frozen, made with margarine | 4.883 |  |  |
| 72119233 | Kale, cooked, from canned, made with oil | 4.891 |  |  |
| 72119234 | Kale, cooked, from canned, made with butter | 4.885 |  |  |
| 72119235 | Kale, cooked, from canned, made with margarine | 4.883 |  |  |
| 72121209 | Mustard Cabbage, cooked, NS as to fat added in cooking | 7.933 |  |  |
| 72121210 | Mustard cabbage, cooked, fat not added in cooking | 8.152 |  |  |
| 72121211 | Mustard Cabbage, cooked, fat added in cooking | 7.933 |  |  |
| 72125240 | Spinach souffle | 3.407 | Spinach souffle | 3.407 |
| 72125260 | Spinach and cheese casserole | 3.627 | Spinach and cheese casserole | 3.627 |
| 72125310 | Palak Paneer | 11.973 | Palak Paneer | 11.973 |
| 72125500 | Channa Saag | 16.748 | Channa Saag | 16.748 |
| 72201100 | Broccoli, raw | 7.800 | Broccoli, raw | 7.800 |
| 72201200 | Broccoli, cooked, NS as to form, NS as to fat added in cooking | 7.564 |  |  |
| 72201201 | Broccoli, cooked, from fresh, NS as to fat added in cooking | 7.524 |  |  |
| 72201202 | Broccoli, cooked, from frozen, NS as to fat added in cooking | 7.564 |  |  |
| 72201210 | Broccoli, cooked, NS as to form, fat not added in cooking | 7.758 |  |  |
| 72201211 | Broccoli, cooked, from fresh, fat not added in cooking | 7.750 | Broccoli, fresh, cooked, no added fat | 7.750 |
| 72201212 | Broccoli, cooked, from frozen, fat not added in cooking | 7.758 | Broccoli, frozen, cooked, no added fat | 7.758 |
| 72201213 | Broccoli, cooked, NS as to form, made with oil | 7.572 |  |  |
| 72201214 | Broccoli, cooked, NS as to form, made with butter | 7.564 |  |  |
| 72201215 | Broccoli, cooked, NS as to form, made with margarine | 7.563 |  |  |
| 72201220 | Broccoli, cooked, NS as to form, fat added in cooking, NS as to type of fat | 7.564 | Broccoli, NS as to form, cooked | 7.564 |
| 72201221 | Broccoli, cooked, from fresh, fat added in cooking, NS as to type of fat | 7.524 | Broccoli, fresh, cooked, fat added, NS as to fat type | 7.524 |
| 72201222 | Broccoli, cooked, from frozen, fat added in cooking, NS as to type of fat | 7.564 | Broccoli, frozen, cooked, fat added, NS as to fat type | 7.564 |
| 72201223 | Broccoli, cooked, from fresh, made with oil | 7.532 | Broccoli, fresh, cooked with oil | 7.532 |
| 72201224 | Broccoli, cooked, from fresh, made with butter | 7.524 | Broccoli, fresh, cooked with butter or margarine | 7.524 |
| 72201225 | Broccoli, cooked, from fresh, made with margarine | 7.522 |  |  |
| 72201226 | Broccoli, cooked, from frozen, made with oil | 7.572 | Broccoli, frozen, cooked with oil | 7.572 |
| 72201227 | Broccoli, cooked, from frozen, made with butter | 7.564 | Broccoli, frozen, cooked with butter or margarine | 7.564 |
| 72201228 | Broccoli, cooked, from frozen, made with margarine | 7.563 |  |  |
| 72201230 | Broccoli, cooked, NS as to form, with cheese sauce | 5.409 |  |  |
| 72201231 | Broccoli, cooked, from fresh, with cheese sauce | 5.126 |  |  |
| 72201232 | Broccoli, cooked, from frozen, with cheese sauce | 5.408 |  |  |
| 72201240 | Broccoli, cooked, NS as to form, with mushroom sauce | 4.807 |  |  |
| 72201241 | Broccoli, cooked, from fresh, with mushroom sauce | 4.497 |  |  |
| 72201242 | Broccoli, cooked, from frozen, with mushroom sauce | 4.807 |  |  |
| 72201250 | Broccoli, cooked, NS as to form, with cream sauce | 5.361 |  |  |
| 72201251 | Broccoli, cooked, from fresh, with cream sauce | 4.324 |  |  |
| 72201252 | Broccoli, cooked, from frozen, with cream sauce | 4.639 |  |  |
| 72202010 | Broccoli casserole with noodles | 20.888 | Broccoli casserole with noodles | 20.888 |
| 72202020 | Broccoli casserole with rice | 6.166 | Broccoli casserole with rice | 6.166 |
| 72202030 | Broccoli, batter-dipped and fried | 8.824 | Fried broccoli | 8.824 |
| 72302100 |  |  | Soup, broccoli cheese | 1.427 |
| 72306000 | Watercress broth with shrimp | 2.886 |  |  |
| 72308000 | Dark-green leafy vegetable soup with meat, Asian style | 2.664 |  |  |
| 72308500 | Dark-green leafy vegetable soup, meatless, Asian style | 3.144 |  |  |
| 73210110 | Pumpkin fritters, Puerto Rican style | 5.496 |  |  |
| 73211110 | Sweet potato and pumpkin casserole, Puerto Rican style | 0.544 |  |  |
| 73304010 | Squash fritter or cake | 11.772 |  |  |
| 73305010 | Squash, winter, baked with cheese | 4.829 |  |  |
| 73305020 | Squash, winter, souffle | 1.453 | Squash, winter, souffle | 1.453 |
| 73502000 | Squash, winter type, soup, home recipe, canned, or ready-to-serve | 0.550 | Soup, pumpkin | 0.550 |
| 74202050 | Tomatoes, red, NS as to form, fried | 7.091 |  |  |
| 74202051 | Tomatoes, red, from fresh, fried | 4.097 |  |  |
| 74203010 | Tomatoes, NS as to form, scalloped | 19.113 | Tomatoes, scalloped | 19.113 |
| 74203011 | Tomatoes, from fresh, scalloped | 5.201 |  |  |
| 74204011 | Tomatoes, from fresh, stewed | 3.059 |  |  |
| 74205010 | Tomatoes, green, cooked, NS as to form | 7.091 | Fried green tomatoes | 7.091 |
| 74205011 | Tomatoes, green, cooked, from fresh | 4.913 |  |  |
| 74205020 | Tomato, green, pickled | 11.191 | Tomato, green, pickled | 11.191 |
| 74402110 | Salsa, pico de gallo | 16.002 | Salsa, pico de gallo | 16.002 |
| 74402200 | Salsa, red, homemade | 8.222 | Salsa, red, homemade | 8.222 |
| 74402260 | Enchilada sauce, green | 14.378 | Enchilada sauce, green | 14.378 |
| 74404010 |  |  | Spaghetti sauce | 48.000 |
| 74404020 |  |  | Spaghetti sauce with added vegetables | 36.706 |
| 74404050 |  |  | Spaghetti sauce, reduced sodium | 48.000 |
| 74404090 | Vodka sauce with tomatoes and cream | 0.327 | Vodka sauce with tomatoes and cream | 0.327 |
| 74405010 | Tomato relish | 22.372 |  |  |
| 74410110 | Puerto Rican seasoning with ham | 30.992 | Puerto Rican seasoning with ham | 30.992 |
| 74415110 | Puerto Rican seasoning with ham and tomato sauce | 18.405 |  |  |
| 74420110 | Puerto Rican seasoning without ham and tomato sauce | 46.438 | Puerto Rican seasoning without ham and tomato sauce | 46.438 |
| 74504100 | Tomato and onion, cooked, NS as to fat added in cooking | 17.873 |  |  |
| 74504110 | Tomato and onion, cooked, fat not added in cooking | 18.234 |  |  |
| 74504120 | Tomato and onion, cooked, fat added in cooking, NS as to type of fat | 17.873 |  |  |
| 74504121 | Tomato and onion, cooked, made with oil | 17.887 |  |  |
| 74504122 | Tomato and onion, cooked, made with butter | 17.873 |  |  |
| 74504123 | Tomato and onion, cooked, made with margarine | 17.869 |  |  |
| 74506000 | Tomato and cucumber salad made with tomato, cucumber, oil, and vinegar | 10.505 | Tomato and cucumber salad made with tomato, cucumber, oil, and vinegar | 10.505 |
| 74601010 |  |  | Soup, cream of tomato | 1.427 |
| 74602010 |  |  | Soup, tomato, canned | 3.773 |
| 74602200 |  |  | Soup, tomato, canned / carton, reduced sodium | 3.780 |
| 74604500 | Tomato noodle soup, canned, prepared with water or ready-to-serve | 11.852 |  |  |
| 74604600 | Tomato noodle soup, canned, prepared with milk | 6.720 |  |  |
| 74701000 | Tomato sandwich | 21.207 | Tomato sandwich on white | 21.207 |
| 74701010 |  |  | Tomato sandwich on wheat | 23.743 |
| 75100750 | Artichoke, Jerusalem, raw | 209.700 | Artichoke | 209.700 |
| 75100800 | Asparagus, raw | 50.000 | Asparagus, raw | 50.000 |
| 75102500 | Beets, raw | 6.000 | Beets, raw | 6.000 |
| 75102750 | Brussels sprouts, raw | 8.200 | Brussels sprouts, raw | 8.200 |
| 75103000 | Cabbage, green, raw | 8.200 | Cabbage, green, raw | 8.200 |
| 75104000 | Cabbage, Chinese, raw | 8.200 | Cabbage, Chinese, raw | 8.200 |
| 75105000 | Cabbage, red, raw | 6.100 | Cabbage, red, raw | 6.100 |
| 75109010 | Fennel bulb, raw | 6.100 | Fennel bulb, raw | 6.100 |
| 75111500 | Garlic, raw | 192.600 | Garlic, raw | 192.600 |
| 75112500 | Leek, raw | 127.700 |  |  |
| 75113000 | Lettuce, raw | 0.500 | Lettuce, raw | 0.500 |
| 75113060 | Lettuce, Boston, raw | 0.500 | Lettuce, Boston, raw | 0.500 |
| 75113070 | Lettuce, manoa | 0.500 |  |  |
| 75114000 | Mixed salad greens, raw | 0.200 | Mixed salad greens, raw | 0.200 |
| 75117010 | Onions, young green, raw | 41.000 | Onions, green, raw | 41.000 |
| 75117020 | Onions, mature, raw | 106.100 | Onions, raw | 106.100 |
| 75127750 | Snowpeas, raw | 6.400 | Snowpeas, raw | 6.400 |
| 75140500 | Broccoli salad with cauliflower, cheese, bacon bits, and dressing | 10.336 | Broccoli salad with cauliflower, cheese, bacon bits, and dressing | 10.336 |
| 75140510 | Broccoli slaw salad | 9.505 | Broccoli slaw salad | 9.505 |
| 75141000 | Cabbage salad or coleslaw, made with coleslaw dressing | 9.750 | Coleslaw | 9.750 |
| 75141005 | Cabbage salad or coleslaw, made with light coleslaw dressing | 9.585 |  |  |
| 75141020 | Cabbage salad or coleslaw, made with Italian dressing | 9.884 |  |  |
| 75141025 | Cabbage salad or coleslaw, made with light Italian dressing | 9.839 |  |  |
| 75141030 | Cabbage salad or coleslaw, made with creamy dressing | 9.839 |  |  |
| 75141035 | Cabbage salad or coleslaw, made with light creamy dressing | 9.628 |  |  |
| 75141040 | Cabbage salad or coleslaw, made with any type of fat free dressing | 9.697 | Cabbage salad, NFS | 9.697 |
| 75141100 | Cabbage salad or coleslaw with apples and/or raisins, with dressing | 7.720 | Coleslaw, with fruit | 7.720 |
| 75141200 | Cabbage salad or coleslaw with pineapple, with dressing | 8.392 |  |  |
| 75141300 | Cabbage, Chinese, salad, with dressing | 3.821 |  |  |
| 75142550 | Cucumber salad, made with Italian dressing | 13.093 | Cucumber salad, made with Italian dressing | 13.093 |
| 75142600 | Cucumber salad made with cucumber and vinegar | 11.724 | Cucumber salad made with cucumber and vinegar | 11.724 |
| 75143000 | Lettuce, salad with assorted vegetables including tomatoes and/or carrots, no dressing | 9.099 | Lettuce, salad with assorted vegetables including tomatoes and/or carrots, no dressing | 9.099 |
| 75143050 | Lettuce, salad with assorted vegetables excluding tomatoes and carrots, no dressing | 10.159 | Lettuce, salad with assorted vegetables excluding tomatoes and carrots, no dressing | 10.159 |
| 75143100 | Lettuce, salad with avocado, tomato, and/or carrots, with or without other vegetables, no dressing | 7.377 | Lettuce, salad with avocado, tomato, and/or carrots, with or without other vegetables, no dressing | 7.377 |
| 75143200 | Lettuce, salad with cheese, tomato and/or carrots, with or without other vegetables, no dressing | 7.516 | Lettuce, salad with cheese, tomato and/or carrots, with or without other vegetables, no dressing | 7.516 |
| 75143300 | Lettuce, salad with egg, tomato, and/or carrots, with or without other vegetables, no dressing | 7.258 | Lettuce, salad with egg, tomato, and/or carrots, with or without other vegetables, no dressing | 7.258 |
| 75145000 | Seven-layer salad, lettuce salad made with a combination of onion, celery, green pepper, peas, mayonnaise, cheese, eggs, and/or bacon | 8.759 | Seven-layer salad, lettuce salad made with a combination of onion, celery, green pepper, peas, mayonnaise, cheese, eggs, and/or bacon | 8.759 |
| 75146000 | Greek Salad, no dressing | 5.826 | Greek Salad, no dressing | 5.826 |
| 75148010 | Cobb salad, no dressing | 0.047 | Cobb salad, no dressing | 0.047 |
| 75202000 | Asparagus, cooked, NS as to form, NS as to fat added in cooking | 32.951 |  |  |
| 75202001 | Asparagus, cooked, from fresh, NS as to fat added in cooking | 32.951 |  |  |
| 75202002 | Asparagus, cooked, from frozen, NS as to fat added in cooking | 32.451 |  |  |
| 75202003 | Asparagus, cooked, from canned, NS as to fat added in cooking | 33.214 |  |  |
| 75202010 | Asparagus, cooked, NS as to form, fat not added in cooking | 33.812 |  |  |
| 75202011 | Asparagus, cooked, from fresh, fat not added in cooking | 33.812 | Asparagus, fresh, cooked, no added fat | 33.812 |
| 75202012 | Asparagus, cooked, from frozen, fat not added in cooking | 33.812 | Asparagus, frozen, cooked, no added fat | 33.812 |
| 75202013 | Asparagus, cooked, from canned, fat not added in cooking | 33.860 | Asparagus, canned, cooked, no added fat | 33.860 |
| 75202020 | Asparagus, cooked, NS as to form, fat added in cooking, NS as to type of fat | 32.951 | Asparagus, NS as to form, cooked | 32.951 |
| 75202021 | Asparagus, cooked, from fresh, fat added in cooking, NS as to type of fat | 32.951 | Asparagus, fresh, cooked, fat added, NS as to fat type | 32.951 |
| 75202022 | Asparagus, cooked, from frozen, fat added in cooking, NS as to type of fat | 32.951 | Asparagus, frozen, cooked, fat added, NS as to fat type | 32.951 |
| 75202023 | Asparagus, cooked, from canned, fat added in cooking, NS as to type of fat | 33.214 | Asparagus, canned, cooked, fat added, NS as to fat type | 33.214 |
| 75202024 | Asparagus, cooked, NS as to form, made with oil | 32.984 |  |  |
| 75202025 | Asparagus, cooked, NS as to form, made with butter | 32.951 |  |  |
| 75202026 | Asparagus, cooked, NS as to form, made with margarine | 32.944 |  |  |
| 75202027 | Asparagus, cooked, from fresh, made with oil | 32.984 | Asparagus, fresh, cooked with oil | 32.984 |
| 75202028 | Asparagus, cooked, from fresh, made with butter | 32.951 | Asparagus, fresh, cooked with butter or margarine | 32.951 |
| 75202029 | Asparagus, cooked, from fresh, made with margarine | 32.944 |  |  |
| 75202031 | Asparagus, cooked, from frozen, made with oil | 32.984 | Asparagus, frozen, cooked with oil | 32.984 |
| 75202032 | Asparagus, cooked, from frozen, made with butter | 32.951 | Asparagus, frozen, cooked with butter or margarine | 32.951 |
| 75202033 | Asparagus, cooked, from frozen, made with margarine | 32.944 |  |  |
| 75202034 | Asparagus, cooked, from canned, made with oil | 33.239 | Asparagus, canned, cooked with oil | 33.239 |
| 75202035 | Asparagus, cooked, from canned, made with butter | 33.214 | Asparagus, canned, cooked with butter or margarine | 33.214 |
| 75202036 | Asparagus, cooked, from canned, made with margarine | 33.208 |  |  |
| 75204000 | Beans, lima, immature, cooked, NS as to form, NS as to fat added in cooking | 12.963 |  |  |
| 75204001 | Beans, lima, immature, cooked, from fresh, NS as to fat added in cooking | 12.963 |  |  |
| 75204002 | Beans, lima, immature, cooked, from frozen, NS as to fat added in cooking | 12.963 |  |  |
| 75204003 | Beans, lima, immature, cooked, from canned, NS as to fat added in cooking | 12.963 |  |  |
| 75204010 | Beans, lima, immature, cooked, NS as to form, fat not added in cooking | 13.322 |  |  |
| 75204011 | Beans, lima, immature, cooked, from fresh, fat not added in cooking | 13.322 |  |  |
| 75204012 | Beans, lima, immature, cooked, from frozen, fat not added in cooking | 13.322 | Lima beans, from frozen, no added fat | 13.322 |
| 75204013 | Beans, lima, immature, cooked, from canned, fat not added in cooking | 13.322 |  |  |
| 75204020 | Beans, lima, immature, cooked, NS as to form, fat added in cooking, NS as to type of fat | 12.963 |  |  |
| 75204021 | Beans, lima, immature, cooked, from fresh, fat added in cooking, NS as to type of fat | 12.963 |  |  |
| 75204022 | Beans, lima, immature, cooked, from frozen, fat added in cooking, NS as to type of fat | 12.966 | Lima beans, from frozen, fat added | 12.966 |
| 75204023 | Beans, lima, immature, cooked, from canned, fat added in cooking, NS as to type of fat | 12.963 | Lima beans, from canned | 12.963 |
| 75204024 | Beans, lima, immature, cooked, NS as to form, made with oil | 12.977 |  |  |
| 75204025 | Beans, lima, immature, cooked, NS as to form, made with butter | 12.963 |  |  |
| 75204026 | Beans, lima, immature, cooked, NS as to form, made with margarine | 12.960 |  |  |
| 75204027 | Beans, lima, immature, cooked, from fresh, made with oil | 12.977 |  |  |
| 75204028 | Beans, lima, immature, cooked, from fresh, made with butter | 12.963 |  |  |
| 75204029 | Beans, lima, immature, cooked, from fresh, made with margarine | 12.960 |  |  |
| 75204030 | Beans, lima, immature, cooked, from frozen, made with oil | 12.977 |  |  |
| 75204031 | Beans, lima, immature, cooked, from frozen, made with butter | 12.963 |  |  |
| 75204032 | Beans, lima, immature, cooked, from frozen, made with margarine | 12.960 |  |  |
| 75204033 | Beans, lima, immature, cooked, from canned, made with oil | 12.977 |  |  |
| 75204034 | Beans, lima, immature, cooked, from canned, made with butter | 12.963 |  |  |
| 75204035 | Beans, lima, immature, cooked, from canned, made with margarine | 12.960 |  |  |
| 75207000 | Bean sprouts, cooked, NS as to form, NS as to fat added in cooking | 3.242 |  |  |
| 75207001 | Bean sprouts, cooked, from fresh, NS as to fat added in cooking | 3.242 |  |  |
| 75207010 | Bean sprouts, cooked, NS as to form, fat not added in cooking | 3.375 |  |  |
| 75207011 | Bean sprouts, cooked, from fresh, fat not added in cooking | 3.375 |  |  |
| 75207020 | Bean sprouts, cooked, NS as to form, fat added in cooking | 3.242 |  |  |
| 75207021 | Bean sprouts, cooked, from fresh, fat added in cooking | 3.242 | Bean sprouts, cooked | 3.242 |
| 75208000 | Beets, cooked, NS as to form, NS as to fat added in cooking | 5.804 |  |  |
| 75208001 | Beets, cooked, from fresh, NS as to fat added in cooking | 5.804 |  |  |
| 75208002 | Beets, cooked, from frozen, NS as to fat added in cooking | 5.804 |  |  |
| 75208003 | Beets, cooked, from canned, NS as to fat added in cooking | 5.804 |  |  |
| 75208010 | Beets, cooked, NS as to form, fat not added in cooking | 5.965 |  |  |
| 75208011 | Beets, cooked, from fresh, fat not added in cooking | 5.965 | Beets, fresh, cooked, no added fat | 5.965 |
| 75208012 | Beets, cooked, from frozen, fat not added in cooking | 5.965 |  |  |
| 75208013 | Beets, cooked, from canned, fat not added in cooking | 5.965 | Beets, canned, cooked, no added fat | 5.965 |
| 75208020 | Beets, cooked, NS as to form, fat added in cooking, NS as to type of fat | 5.804 | Beets, NS as to form, cooked | 5.804 |
| 75208021 | Beets, cooked, from fresh, fat added in cooking, NS as to type of fat | 5.804 | Beets, fresh, cooked, fat added | 5.804 |
| 75208022 | Beets, cooked, from frozen, fat added in cooking, NS as to type of fat | 5.804 |  |  |
| 75208023 | Beets, cooked, from canned, fat added in cooking, NS as to type of fat | 5.804 | Beets, canned, cooked, fat added | 5.804 |
| 75208121 | Beets, cooked, NS as to form, made with oil | 5.811 |  |  |
| 75208122 | Beets, cooked, NS as to form, made with butter | 5.804 |  |  |
| 75208123 | Beets, cooked, NS as to form, made with margarine | 5.803 |  |  |
| 75208124 | Beets, cooked, from fresh, made with oil | 5.811 |  |  |
| 75208125 | Beets, cooked, from fresh, made with butter | 5.804 |  |  |
| 75208126 | Beets, cooked, from fresh, made with margarine | 5.803 |  |  |
| 75208127 | Beets, cooked, from frozen, made with oil | 5.811 |  |  |
| 75208128 | Beets, cooked, from frozen, made with butter | 5.804 |  |  |
| 75208129 | Beets, cooked, from frozen, made with margarine | 5.803 |  |  |
| 75208130 | Beets, cooked, from canned, made with oil | 5.811 |  |  |
| 75208131 | Beets, cooked, from canned, made with butter | 5.804 |  |  |
| 75208132 | Beets, cooked, from canned, made with margarine | 5.803 |  |  |
| 75209000 | Brussels sprouts, cooked, NS as to form, NS as to fat added in cooking | 7.908 |  |  |
| 75209001 | Brussels sprouts, cooked, from fresh, NS as to fat added in cooking | 7.910 |  |  |
| 75209002 | Brussels sprouts, cooked, from frozen, NS as to fat added in cooking | 7.908 |  |  |
| 75209010 | Brussels sprouts, cooked, NS as to form, fat not added in cooking | 8.147 |  |  |
| 75209011 | Brussels sprouts, cooked, from fresh, fat not added in cooking | 8.148 | Brussels sprouts, fresh, cooked, no added fat | 8.148 |
| 75209012 | Brussels sprouts, cooked, from frozen, fat not added in cooking | 8.147 | Brussels sprouts, frozen, cooked, no added fat | 8.147 |
| 75209020 | Brussels sprouts, cooked, NS as to form, fat added in cooking, NS as to type of fat | 7.908 | Brussels sprouts, NS as to form, cooked | 7.908 |
| 75209021 | Brussels sprouts, cooked, from fresh, fat added in cooking, NS as to type of fat | 7.911 | Brussels sprouts, fresh, cooked, fat added | 7.911 |
| 75209022 | Brussels sprouts, cooked, from frozen, fat added in cooking, NS as to type of fat | 7.908 | Brussels sprouts, frozen, cooked, fat added | 7.908 |
| 75209030 | Brussels sprouts, cooked, NS as to form, made with oil | 7.917 |  |  |
| 75209031 | Brussels sprouts, cooked, NS as to form, made with butter | 7.908 |  |  |
| 75209032 | Brussels sprouts, cooked, NS as to form, made with margarine | 7.906 |  |  |
| 75209040 | Brussels sprouts, cooked, from fresh, made with oil | 7.919 |  |  |
| 75209041 | Brussels sprouts, cooked, from fresh, made with butter | 7.910 |  |  |
| 75209042 | Brussels sprouts, cooked, from fresh, made with margarine | 7.907 |  |  |
| 75209050 | Brussels sprouts, cooked, from frozen, made with oil | 7.917 |  |  |
| 75209051 | Brussels sprouts, cooked, from frozen, made with butter | 7.908 |  |  |
| 75209052 | Brussels sprouts, cooked, from frozen, made with margarine | 7.906 |  |  |
| 75210000 | Cabbage, Chinese, cooked, NS as to fat added in cooking | 7.941 |  |  |
| 75210010 | Cabbage, Chinese, cooked, fat not added in cooking | 8.152 | Cabbage, Chinese, cooked, no added fat | 8.152 |
| 75210020 | Cabbage, Chinese, cooked, fat added in cooking, NS as to type of fat | 7.941 | Cabbage, Chinese, cooked, fat added | 7.941 |
| 75210021 | Cabbage, Chinese, cooked, made with oil | 7.941 |  |  |
| 75210022 | Cabbage, Chinese, cooked, made with butter | 7.933 |  |  |
| 75210023 | Cabbage, Chinese, cooked, made with margarine | 7.931 |  |  |
| 75211010 | Cabbage, green, cooked, NS as to fat added in cooking | 7.898 |  |  |
| 75211020 | Cabbage, green, cooked, fat not added in cooking | 8.146 | Cabbage, green, cooked, no added fat | 8.146 |
| 75211030 | Cabbage, green, cooked, fat added in cooking, NS as to type of fat | 7.898 | Cabbage, green, cooked, fat added, NS as to fat type | 7.898 |
| 75211031 | Cabbage, green, cooked, made with oil | 7.908 | Cabbage, green, cooked with oil | 7.908 |
| 75211032 | Cabbage, green, cooked, made with butter | 7.898 | Cabbage, green, cooked with butter or margarine | 7.898 |
| 75211033 | Cabbage, green, cooked, made with margarine | 7.896 |  |  |
| 75212000 | Cabbage, red, cooked, NS as to fat added in cooking | 5.876 |  |  |
| 75212010 | Cabbage, red, cooked, fat not added in cooking | 6.060 |  |  |
| 75212020 | Cabbage, red, cooked, fat added in cooking, NS as to type of fat | 5.876 | Cabbage, red, cooked | 5.876 |
| 75212021 | Cabbage, red, cooked, made with oil | 5.883 |  |  |
| 75212022 | Cabbage, red, cooked, made with butter | 5.876 |  |  |
| 75212023 | Cabbage, red, cooked, made with margarine | 5.874 |  |  |
| 75213000 | Cabbage, savoy, cooked, NS as to fat added in cooking | 7.900 |  |  |
| 75213010 | Cabbage, savoy, cooked, fat not added in cooking | 8.151 |  |  |
| 75213020 | Cabbage, savoy, cooked, fat added in cooking, NS as to type of fat | 7.900 | Cabbage, savoy, cooked | 7.900 |
| 75213021 | Cabbage, savoy, cooked, made with oil | 7.900 |  |  |
| 75213022 | Cabbage, savoy, cooked, made with butter | 7.900 |  |  |
| 75213023 | Cabbage, savoy, cooked, made with margarine | 7.900 |  |  |
| 75215100 | Fennel bulb, cooked, NS as to fat added in cooking | 5.877 |  |  |
| 75215110 | Fennel bulb, cooked, fat not added in cooking | 6.064 |  |  |
| 75215120 | Fennel bulb, cooked, fat added in cooking, NS as to type of fat | 5.877 | Fennel bulb, cooked | 5.877 |
| 75215121 | Fennel bulb, cooked, made with oil | 5.877 |  |  |
| 75215122 | Fennel bulb, cooked, made with butter | 5.877 |  |  |
| 75215123 | Fennel bulb, cooked, made with margarine | 5.877 |  |  |
| 75217400 | Garlic, cooked | 191.451 | Garlic, cooked | 191.451 |
| 75218400 | Leek, cooked, NS as to fat added in cooking | 123.025 | Leeks | 123.025 |
| 75220049 | Lettuce, cooked, NS as to fat added in cooking | 0.482 |  |  |
| 75220050 | Lettuce, cooked, fat not added in cooking | 0.497 |  |  |
| 75220051 | Lettuce, cooked, fat added in cooking | 0.482 | Lettuce, cooked | 0.482 |
| 75221000 | Onions, cooked, NS as to form, NS as to fat added in cooking | 79.209 |  |  |
| 75221001 | Onions, cooked, from fresh, NS as to fat added in cooking | 78.946 |  |  |
| 75221002 | Onions, cooked, from frozen, NS as to fat added in cooking | 79.209 |  |  |
| 75221010 | Onions, cooked, NS as to form, fat not added in cooking | 80.763 |  |  |
| 75221011 | Onions, cooked, from fresh, fat not added in cooking | 80.716 | Onions, cooked, no added fat | 80.716 |
| 75221012 | Onions, cooked, from frozen, fat not added in cooking | 80.931 |  |  |
| 75221014 | Onions, cooked, NS as to form, made with oil | 79.270 |  |  |
| 75221015 | Onions, cooked, NS as to form, made with butter | 79.209 |  |  |
| 75221016 | Onions, cooked, NS as to form, made with margarine | 79.196 |  |  |
| 75221017 | Onions, cooked, from fresh, made with oil | 79.015 |  |  |
| 75221018 | Onions, cooked, from fresh, made with butter | 78.946 |  |  |
| 75221019 | Onions, cooked, from fresh, made with margarine | 78.931 |  |  |
| 75221020 | Onions, cooked, NS as to form, fat added in cooking, NS as to type of fat | 79.209 |  |  |
| 75221021 | Onions, cooked, from fresh, fat added in cooking, NS as to type of fat | 78.946 | Onions, cooked, fat added | 78.946 |
| 75221022 | Onions, cooked, from frozen, fat added in cooking, NS as to type of fat | 79.209 |  |  |
| 75221023 | Onions, cooked, from frozen, made with oil | 79.015 |  |  |
| 75221024 | Onions, cooked, from frozen, made with butter | 78.946 |  |  |
| 75221025 | Onions, cooked, from frozen, made with margarine | 78.931 |  |  |
| 75221030 | Onions, pearl, cooked, NS as to form | 80.716 | Onions, pearl, cooked | 80.716 |
| 75221031 | Onions, pearl, cooked, from fresh | 80.716 |  |  |
| 75221032 | Onions, pearl, cooked, from frozen | 80.716 |  |  |
| 75221033 | Onions, pearl, cooked, from canned | 80.716 |  |  |
| 75221040 | Onions, green, cooked, NS as to form, NS as to fat added in cooking | 39.499 |  |  |
| 75221041 | Onions, green, cooked, from fresh, NS as to fat added in cooking | 39.499 |  |  |
| 75221050 | Onions, green, cooked, NS as to form, fat not added in cooking | 40.755 |  |  |
| 75221051 | Onions, green, cooked, from fresh, fat not added in cooking | 40.594 |  |  |
| 75221060 | Onions, green, cooked, NS as to form, fat added in cooking | 39.499 |  |  |
| 75221061 | Onions, green, cooked, from fresh, fat added in cooking | 39.499 | Onions, green, cooked | 39.499 |
| 75223000 | Peas, cowpeas, field peas, or blackeye peas, not dried, cooked, NS as to form, NS as to fat added in cooking | 49.047 |  |  |
| 75223001 | Peas, cowpeas, field peas, or blackeye peas, not dried, cooked, from fresh, NS as to fat added in cooking | 49.047 |  |  |
| 75223002 | Peas, cowpeas, field peas, or blackeye peas, not dried, cooked, from frozen, NS as to fat added in cooking | 49.047 |  |  |
| 75223003 | Peas, cowpeas, field peas, or blackeye peas, not dried, cooked, from canned, NS as to fat added in cooking | 48.866 |  |  |
| 75223010 | Peas, cowpeas, field peas, or blackeye peas, not dried, cooked, NS as to form, fat not added in cooking | 50.404 |  |  |
| 75223011 | Peas, cowpeas, field peas, or blackeye peas, not dried, cooked, from fresh, fat not added in cooking | 50.395 |  |  |
| 75223012 | Peas, cowpeas, field peas, or blackeye peas, not dried, cooked, from frozen, fat not added in cooking | 50.404 |  |  |
| 75223013 | Peas, cowpeas, field peas, or blackeye peas, not dried, cooked, from canned, fat not added in cooking | 50.213 |  |  |
| 75223020 | Peas, cowpeas, field peas, or blackeye peas, not dried, cooked, NS as to form, fat added in cooking, NS as to type of fat | 49.047 |  |  |
| 75223021 | Peas, cowpeas, field peas, or blackeye peas, not dried, cooked, from fresh, fat added in cooking, NS as to type of fat | 48.999 |  |  |
| 75223022 | Peas, cowpeas, field peas, or blackeye peas, not dried, cooked, from frozen, fat added in cooking, NS as to type of fat | 49.047 | Blackeyed peas, from frozen | 49.047 |
| 75223023 | Peas, cowpeas, field peas, or blackeye peas, not dried, cooked, from canned, fat added in cooking, NS as to type of fat | 48.866 | Blackeyed peas, from canned | 48.866 |
| 75223024 | Peas, cowpeas, field peas, or blackeye peas, not dried, cooked, NS as to form, made with oil | 49.099 |  |  |
| 75223025 | Peas, cowpeas, field peas, or blackeye peas, not dried, cooked, NS as to form, made with butter | 49.047 |  |  |
| 75223026 | Peas, cowpeas, field peas, or blackeye peas, not dried, cooked, NS as to form, made with margarine | 49.035 |  |  |
| 75223027 | Peas, cowpeas, field peas, or blackeye peas, not dried, cooked, from fresh, made with oil | 49.052 |  |  |
| 75223028 | Peas, cowpeas, field peas, or blackeye peas, not dried, cooked, from fresh, made with butter | 48.999 |  |  |
| 75223029 | Peas, cowpeas, field peas, or blackeye peas, not dried, cooked, from fresh, made with margarine | 48.987 |  |  |
| 75223030 | Peas, cowpeas, field peas, or blackeye peas, not dried, cooked, from frozen, made with oil | 49.099 |  |  |
| 75223031 | Peas, cowpeas, field peas, or blackeye peas, not dried, cooked, from frozen, made with butter | 49.047 |  |  |
| 75223032 | Peas, cowpeas, field peas, or blackeye peas, not dried, cooked, from frozen, made with margarine | 50.170 |  |  |
| 75223033 | Peas, cowpeas, field peas, or blackeye peas, not dried, cooked, from canned, made with oil | 48.918 |  |  |
| 75223034 | Peas, cowpeas, field peas, or blackeye peas, not dried, cooked, from canned, made with butter | 48.866 |  |  |
| 75223035 | Peas, cowpeas, field peas, or blackeye peas, not dried, cooked, from canned, made with margarine | 48.855 |  |  |
| 75231000 | Snowpea, cooked, NS as to form, NS as to fat added in cooking | 6.186 |  |  |
| 75231001 | Snowpea, cooked, from fresh, NS as to fat added in cooking | 6.186 |  |  |
| 75231002 | Snowpea, cooked, from frozen, NS as to fat added in cooking | 6.186 |  |  |
| 75231010 | Snowpea, cooked, NS as to form, fat not added in cooking | 6.360 |  |  |
| 75231011 | Snowpea, cooked, from fresh, fat not added in cooking | 6.360 | Snowpeas, fresh, cooked, no added fat | 6.360 |
| 75231012 | Snowpea, cooked, from frozen, fat not added in cooking | 6.360 | Snowpeas, frozen, cooked, no added fat | 6.360 |
| 75231020 | Snowpea, cooked, NS as to form, fat added in cooking, NS as to type of fat | 6.186 | Snowpeas, NS as to form, cooked | 6.186 |
| 75231021 | Snowpea, cooked, from fresh, fat added in cooking, NS as to type of fat | 6.186 | Snowpeas, fresh, cooked, fat added | 6.186 |
| 75231022 | Snowpea, cooked, from frozen, fat added in cooking, NS as to type of fat | 6.186 | Snowpeas, frozen, cooked, fat added | 6.186 |
| 75231023 | Snowpea, cooked, NS as to form, made with oil | 6.186 |  |  |
| 75231024 | Snowpea, cooked, NS as to form, made with butter | 6.179 |  |  |
| 75231025 | Snowpea, cooked, NS as to form, made with margarine | 6.177 |  |  |
| 75231026 | Snowpea, cooked, from fresh, made with oil | 6.186 |  |  |
| 75231027 | Snowpea, cooked, from fresh, made with butter | 6.179 |  |  |
| 75231028 | Snowpea, cooked, from fresh, made with margarine | 6.177 |  |  |
| 75231029 | Snowpea, cooked, from frozen, made with oil | 6.186 |  |  |
| 75231030 | Snowpea, cooked, from frozen, made with butter | 6.179 |  |  |
| 75231031 | Snowpea, cooked, from frozen, made with margarine | 6.177 |  |  |
| 75301110 |  |  | Lima beans and corn, cooked, no added fat | 6.481 |
| 75301120 |  |  | Lima beans and corn, cooked, fat added | 6.292 |
| 75302029 | Beans, string, green, with chickpeas, cooked, NS as to fat added in cooking | 3.648 |  |  |
| 75302030 | Beans, string, green, with chickpeas, cooked, fat not added in cooking | 3.775 |  |  |
| 75302031 | Beans, string, green, with chickpeas, cooked, fat added in cooking | 3.648 |  |  |
| 75302069 | Beans, string, green, with spaetzel, cooked, NS as to fat added in cooking | 13.766 |  |  |
| 75302070 | Beans, string, green, with spaetzel, cooked, fat not added in cooking | 14.249 |  |  |
| 75302071 | Beans, string, green, with spaetzel, cooked, fat added in cooking | 13.766 |  |  |
| 75302080 | Bean salad, yellow and/or green string beans | 14.665 | Bean salad, yellow and/or green string beans | 14.665 |
| 75302200 | Beans, string, green, with onions, NS as to fat added in cooking | 28.015 |  |  |
| 75302205 | Beans, string, green, with onions, cooked, fat not added in cooking | 28.915 |  |  |
| 75302210 | Beans, string, green, with onions, cooked, fat added in cooking, NS as to type of fat | 28.015 |  |  |
| 75302211 | Beans, string, green, with onions, cooked, made with oil | 28.050 |  |  |
| 75302212 | Beans, string, green, with onions, cooked, made with butter | 28.015 |  |  |
| 75302213 | Beans, string, green, with onions, cooked, made with margarine | 28.008 |  |  |
| 75306998 | Green peppers and onions, cooked, fat not added in cooking | 48.940 | Peppers and onions, cooked, no added fat | 48.940 |
| 75306999 | Green peppers and onions, cooked, NS as to fat added in cooking | 47.645 |  |  |
| 75307000 | Green peppers and onions, cooked, fat added in cooking, NS as to type of fat | 47.645 | Peppers and onions, cooked, fat added | 47.645 |
| 75307001 | Green peppers and onions, cooked, made with oil | 47.695 |  |  |
| 75307002 | Green peppers and onions, cooked, made with butter | 47.645 |  |  |
| 75307003 | Green peppers and onions, cooked, made with margarine | 47.634 |  |  |
| 75315249 | Cowpeas with snap beans, cooked, NS as to fat added in cooking | 39.081 |  |  |
| 75315250 | Cowpeas with snap beans, cooked, fat not added in cooking | 40.219 |  |  |
| 75315251 | Cowpeas with snap beans, cooked, fat added in cooking | 39.035 |  |  |
| 75315999 | Squash, summer, yellow or green, and onions, cooked, NS as to fat added in cooking | 22.035 |  |  |
| 75316000 | Squash, summer, yellow or green, and onions, cooked, fat not added in cooking | 22.588 |  |  |
| 75316020 | Squash, summer, yellow or green, and onions, cooked, fat added in cooking | 22.035 |  |  |
| 75316022 | Squash, summer, yellow or green, and onions, cooked, made with oil | 22.056 |  |  |
| 75316023 | Squash, summer, yellow or green, and onions, cooked, made with butter | 22.035 |  |  |
| 75316024 | Squash, summer, yellow or green, and onions, cooked, made with margarine | 22.030 |  |  |
| 75316050 | Ratatouille | 14.626 | Ratatouille | 14.626 |
| 75317000 | Vegetables, stew type, cooked, NS as to fat added in cooking | 7.813 |  |  |
| 75317010 | Vegetables, stew type, cooked, fat added in cooking, NS as to type of fat | 7.813 | Vegetables, stew type, cooked, fat added | 7.813 |
| 75317011 | Vegetables, stew type, cooked, made with oil | 7.813 |  |  |
| 75317012 | Vegetables, stew type, cooked, made with butter | 7.813 |  |  |
| 75317013 | Vegetables, stew type, cooked, made with margarine | 7.813 |  |  |
| 75317020 | Vegetables, stew type, cooked, fat not added in cooking | 8.062 | Vegetables, stew type, cooked, no added fat | 8.062 |
| 75330100 | Vegetable combination, including carrots, broccoli, and/or dark-green leafy; cooked, no sauce, NS as to fat added in cooking | 2.048 |  |  |
| 75330110 | Vegetable combination, including carrots, broccoli, and/or dark-green leafy; cooked, no sauce, fat not added in cooking | 2.115 |  |  |
| 75330120 | Vegetable combination, including carrots, broccoli, and/or dark-green leafy; cooked, no sauce, fat added in cooking, NS as to type of fat | 2.045 |  |  |
| 75330121 | Vegetable combination, including carrots, broccoli, and/or dark-green leafy; cooked, no sauce, made with oil | 2.048 |  |  |
| 75330122 | Vegetable combination, including carrots, broccoli, and/or dark-green leafy; cooked, no sauce, made with butter | 2.045 |  |  |
| 75330123 | Vegetable combination, including carrots, broccoli, and/or dark-green leafy; cooked, no sauce, made with margarine | 2.045 |  |  |
| 75330130 | Vegetable combination, excluding carrots, broccoli, and dark-green leafy; cooked, no sauce, NS as to fat added in cooking | 13.002 |  |  |
| 75330140 | Vegetable combination, excluding carrots, broccoli, and dark-green leafy; cooked, no sauce, fat not added in cooking | 13.389 |  |  |
| 75330150 | Vegetable combination, excluding carrots, broccoli, and dark-green leafy; cooked, no sauce, fat added in cooking, NS as to type of fat | 13.002 |  |  |
| 75330151 | Vegetable combination, excluding carrots, broccoli, and dark-green leafy; cooked, no sauce, made with oil | 13.017 |  |  |
| 75330152 | Vegetable combination, excluding carrots, broccoli, and dark-green leafy; cooked, no sauce, made with butter | 13.002 |  |  |
| 75330153 | Vegetable combination, excluding carrots, broccoli, and dark-green leafy; cooked, no sauce, made with margarine | 12.999 |  |  |
| 75340000 | Vegetable combinations, Asian style, broccoli, green pepper, water chestnut, etc., cooked, NS as to fat added in cooking | 3.579 |  |  |
| 75340010 | Vegetable combinations, Asian style, broccoli, green pepper, water chestnut, etc., cooked, fat not added in cooking | 3.085 | Asian stir fry vegetables, cooked, no added fat | 3.085 |
| 75340020 | Vegetable combinations, Asian style, broccoli, green pepper, water chestnut, etc., cooked, fat added in cooking, NS as to type of fat | 3.579 | Asian stir fry vegetables, cooked, fat added | 3.579 |
| 75340021 | Vegetable combinations, Asian style, broccoli, green pepper, water chestnut, etc., cooked, made with oil | 3.583 |  |  |
| 75340022 | Vegetable combinations, Asian style, broccoli, green pepper, water chestnut, etc., cooked, made with butter | 3.579 |  |  |
| 75340023 | Vegetable combinations, Asian style, broccoli, green pepper, water chestnut, etc., cooked, made with margarine | 2.867 |  |  |
| 75340160 | Vegetable and pasta combinations with cream or cheese sauce, broccoli, pasta, carrots, corn, zucchini, peppers, cauliflower, peas, etc., cooked | 9.018 |  |  |
| 75340300 | Pinacbet | 10.959 |  |  |
| 75365000 | Vegetable mixture, dried | 7.288 | Vegetable mixture, dried | 7.288 |
| 75400500 | Artichokes, stuffed | 17.558 | Artichokes, stuffed | 17.558 |
| 75401010 | Asparagus, NS as to form, creamed or with cheese sauce | 23.294 |  |  |
| 75401011 | Asparagus, from fresh, creamed or with cheese sauce | 23.307 |  |  |
| 75401012 | Asparagus, from frozen, creamed or with cheese sauce | 23.307 |  |  |
| 75401013 | Asparagus, from canned, creamed or with cheese sauce | 25.349 |  |  |
| 75402010 | Beans, lima, immature, NS as to form, creamed or with cheese sauce | 9.014 |  |  |
| 75402011 | Beans, lima, immature, from fresh, creamed or with cheese sauce | 9.019 |  |  |
| 75402012 | Beans, lima, immature, from frozen, creamed or with cheese sauce | 9.019 |  |  |
| 75402013 | Beans, lima, immature, from canned, creamed or with cheese sauce | 9.019 |  |  |
| 75402020 | Beans, lima, immature, cooked, NS as to form, with mushroom sauce | 8.005 |  |  |
| 75402021 | Beans, lima, immature, cooked, from fresh, with mushroom sauce | 8.005 |  |  |
| 75402022 | Beans, lima, immature, cooked, from frozen, with mushroom sauce | 8.005 |  |  |
| 75402023 | Beans, lima, immature, cooked, from canned, with mushroom sauce | 8.005 |  |  |
| 75403200 | Beans, string, green, cooked, Szechuan-style | 8.132 | Green beans, cooked, Szechuan-style | 8.132 |
| 75405010 | Beets with Harvard sauce | 4.073 |  |  |
| 75406010 | Brussels sprouts, NS as to form, creamed | 4.533 |  |  |
| 75406011 | Brussels sprouts, from fresh, creamed | 4.546 |  |  |
| 75406012 | Brussels sprouts, from frozen, creamed | 4.533 |  |  |
| 75407010 | Cabbage, creamed | 4.466 |  |  |
| 75409020 | Cauliflower, batter-dipped, fried | 5.725 | Fried cauliflower | 5.725 |
| 75410500 | Chiles rellenos, cheese-filled | 1.473 | Chiles rellenos, cheese-filled | 1.473 |
| 75410530 | Chiles rellenos, filled with meat and cheese | 4.807 | Chiles rellenos, filled with meat and cheese | 4.807 |
| 75410550 | Jalapeno pepper, stuffed with cheese, breaded or battered, fried | 6.204 | Stuffed jalapeno pepper | 6.204 |
| 75411020 | Corn fritter | 12.885 | Fritter, corn | 12.885 |
| 75412010 | Eggplant, batter-dipped, fried | 3.209 | Fried eggplant | 3.209 |
| 75412030 | Eggplant dip | 2.891 | Eggplant dip | 2.891 |
| 75412060 | Eggplant parmesan casserole, regular | 1.837 | Eggplant parmesan casserole, regular | 1.837 |
| 75414020 | Mushrooms, stuffed | 12.160 | Mushrooms, stuffed | 12.160 |
| 75414030 | Mushrooms, batter-dipped, fried | 8.537 | Fried mushrooms | 8.537 |
| 75414500 | Okra, batter-dipped, fried | 3.645 | Fried okra | 3.645 |
| 75415010 | Onions, NS as to form, creamed | 50.778 |  |  |
| 75415011 | Onions, from fresh, creamed | 50.778 |  |  |
| 75415021 | Onion rings, from fresh, batter-dipped, baked or fried | 58.097 |  |  |
| 75415022 |  |  | Fried onion rings | 58.000 |
| 75418000 | Squash, summer, yellow or green, breaded or battered, baked | 6.931 |  |  |
| 75418010 | Squash, summer, yellow or green, breaded or battered, fried | 6.115 | Fried summer squash, yellow or green | 6.115 |
| 75418020 | Squash, summer, casserole with tomato and cheese | 3.266 | Squash, summer, casserole with tomato and cheese | 3.266 |
| 75418030 | Squash, summer, casserole, with rice and tomato sauce | 2.461 | Squash, summer, casserole, with rice and tomato sauce | 2.461 |
| 75418040 | Squash, summer, casserole, with cheese sauce | 3.550 | Squash, summer, casserole, with cheese sauce | 3.550 |
| 75418060 | Squash, summer, souffle | 1.775 | Squash, summer, souffle | 1.775 |
| 75418220 | Creamed christophine, Puerto Rican style | 1.236 |  |  |
| 75439010 | Vegetable stew without meat | 10.351 | Stew, vegetable | 10.351 |
| 75439500 | Chow mein or chop suey, meatless, no noodles | 24.960 | Chow mein or chop suey, meatless, no noodles | 24.960 |
| 75440100 | Vegetable combination, including carrots, broccoli, and/or dark-green leafy; cooked, with soy-based sauce | 25.956 |  |  |
| 75440110 | Vegetable combination, excluding carrots, broccoli, and dark-green leafy; cooked, with soy-based sauce | 25.685 |  |  |
| 75440200 | Vegetable tempura | 14.267 | Vegetable tempura | 14.267 |
| 75440300 | Vegetable combinations, including carrots, broccoli, and/or dark-green leafy; cooked, with tomato sauce | 1.328 |  |  |
| 75440310 | Vegetable combinations, excluding carrots, broccoli, and dark-green leafy; cooked, with tomato sauce | 8.723 |  |  |
| 75440400 | Vegetables, dipped in chick-pea flour batter, fried, Pakora | 5.092 | Pakora | 5.092 |
| 75440500 | Vegetable combinations, including carrots, broccoli, and/or dark-green leafy; cooked, with cheese sauce | 1.356 |  |  |
| 75440510 | Vegetable combinations, excluding carrots, broccoli, and dark-green leafy; cooked, with cheese sauce | 8.893 |  |  |
| 75440600 | Vegetable curry | 6.143 | Vegetable curry | 6.143 |
| 75450500 | Vegetable combination, including carrots, broccoli, and/or dark-green leafy; cooked, with cream sauce | 1.343 |  |  |
| 75450510 | Vegetable combination, excluding carrots, broccoli, and dark-green leafy; cooked, with cream sauce | 8.807 |  |  |
| 75450600 | Vegetable combination, including carrots, broccoli, and/or dark-green leafy; cooked, with butter sauce | 2.054 |  |  |
| 75460700 | Vegetable combinations, including carrots, broccoli, and/or dark-green leafy; cooked, with pasta | 13.033 |  |  |
| 75460710 | Vegetable combinations, excluding carrots, broccoli, and dark-green leafy; cooked, with pasta | 20.764 |  |  |
| 75460800 | Vegetable combinations, including carrots, broccoli, and/or dark-green leafy; cooked, with butter sauce and pasta | 15.475 |  |  |
| 75460810 | Vegetable combinations, excluding carrots, broccoli, and dark-green leafy; cooked, with butter sauce and pasta | 20.824 |  |  |
| 75460900 | Chow mein or chop suey, meatless, with noodles | 28.932 | Chow mein or chop suey, meatless, with noodles | 28.932 |
| 75501010 | Corn relish | 3.993 | Relish, corn | 3.993 |
| 75502010 | Cauliflower, pickled | 16.293 | Cauliflower, pickled | 16.293 |
| 75502500 | Cabbage, fresh, pickled, Japanese style | 8.200 | Cabbage, green, pickled | 8.200 |
| 75502510 | Cabbage, red, pickled | 2.899 | Cabbage, red, pickled | 2.899 |
| 75502550 | Cabbage, mustard, salted | 8.200 |  |  |
| 75511020 | Peppers, pickled | 13.969 | Peppers, sweet, pickled | 13.969 |
| 75515000 | Vegetables, pickled, Hawaiian style | 2.535 |  |  |
| 75515010 | Vegetable relish | 6.839 |  |  |
| 75534030 | Turnip, pickled | 14.407 | Turnip, pickled | 14.407 |
| 75534500 | Tsukemono, Japanese pickles | 4.100 |  |  |
| 75535000 | Zucchini, pickled | 16.860 | Zucchini, pickled | 16.860 |
| 75601100 | Borscht | 13.160 | Soup, borscht | 13.160 |
| 75601200 | Cabbage soup, home recipe, canned or ready-to-serve | 4.346 |  |  |
| 75601210 | Cabbage with meat soup, home recipe, canned or ready-to-serve | 4.776 |  |  |
| 75604020 | Corn soup, cream of, prepared with water | 0.486 |  |  |
| 75604600 | Gazpacho | 4.515 | Soup, gazpacho | 4.515 |
| 75607060 |  |  | Soup, cream of mushroom | 1.427 |
| 75608100 | Onion soup, French | 37.656 | Soup, French onion | 37.656 |
| 75611010 |  |  | Soup, cream of vegetable | 1.427 |
| 75647000 | Seaweed soup | 1.843 | Soup, seaweed | 1.843 |
| 75649110 | Vegetable soup, home recipe | 4.248 | Soup, vegetable | 4.248 |
| 75649150 | Vegetable noodle soup, home recipe | 6.853 |  |  |
| 75651000 | Minestrone soup, home recipe | 6.285 | Soup, minestrone | 6.285 |
| 75651140 | Vegetable soup with chicken broth, home recipe, Mexican style | 3.691 |  |  |
| 75652010 | Vegetable beef soup, home recipe | 4.703 | Soup, beef | 4.703 |
| 75652040 | Vegetable beef soup with noodles or pasta, home recipe | 6.519 |  |  |
| 75652050 | Vegetable beef soup with rice, home recipe | 4.816 |  |  |
| 75656010 | Vegetable soup, Spanish style, stew type | 3.316 |  |  |
| 76102030 | Broccoli, carrots and cheese, baby food, junior | 2.337 |  |  |
| 76403010 | Beets, baby food, strained | 6.000 | Baby Toddler beets | 6.000 |
| 77121010 | Fried stuffed potatoes, Puerto Rican style | 0.779 | Fried stuffed potatoes, Puerto Rican style | 0.779 |
| 77141010 | Potato chicken pie, Puerto Rican style | 12.221 |  |  |
| 77205110 | Ripe plantain fritters, Puerto Rican style | 2.259 |  |  |
| 77205610 | Ripe plantain meat pie, Puerto Rican style | 2.776 | Ripe plantain meat pie, Puerto Rican style | 2.776 |
| 77230210 | Cassava Pasteles, Puerto Rican style | 5.740 |  |  |
| 77230510 | Cassava fritter stuffed with crab meat, Puerto Rican style | 3.565 |  |  |
| 77250110 | Stuffed tannier fritters, Puerto Rican style | 3.205 |  |  |
| 77272010 | Puerto Rican pasteles | 3.981 | Puerto Rican pasteles | 3.981 |
| 77316010 | Stuffed cabbage, with meat, Puerto Rican style | 8.341 | Stuffed cabbage, with meat, Puerto Rican style | 8.341 |
| 77316510 | Stuffed cabbage, with meat and rice, Syrian dish, Puerto Rican style | 4.355 | Stuffed cabbage, with meat and rice, Syrian dish, Puerto Rican style | 4.355 |
| 77316600 | Eggplant and meat casserole | 5.459 | Eggplant and meat casserole | 5.459 |
| 77513010 | Spanish stew | 3.316 |  |  |
| 77563010 | Puerto Rican stew | 0.515 |  |  |
| 78101100 | Fruit and vegetable smoothie | 3.786 | Fruit and vegetable smoothie, with dairy | 3.786 |
| 78101110 | Fruit and vegetable smoothie, added protein | 3.495 | Fruit and vegetable smoothie, added protein | 3.495 |
| 78101115 |  |  | Fruit and vegetable smoothie, non-dairy | 3.786 |
| 78101118 |  |  | Fruit and vegetable smoothie, non-dairy, added protein | 3.495 |
| 78101125 |  |  | Fruit and vegetable smoothie, no dairy | 3.782 |
| 81301000 | Garlic sauce | 4.622 | Garlic sauce | 4.622 |
| 81302070 | Pesto sauce | 4.419 | Pesto sauce | 4.419 |
| 81322000 | Honey butter | 4.492 | Honey butter | 4.492 |
| 83108000 |  |  | Vegan mayonnaise | 7.900 |
| 83208500 | Korean dressing or marinade | 3.541 | Korean dressing or marinade | 3.541 |
| 89902040 |  |  | Onions, for use on a sandwich | 106.100 |
| 91302010 | Honey | 7.500 | Honey | 7.500 |
| 91361070 | Plum sauce, Asian style | 2.889 |  |  |
| 91407150 | Bean paste, sweetened | 13.220 | Bean paste, sweetened | 13.220 |
| 91501020 | Gelatin dessert with fruit | 1.744 | Gelatin dessert with fruit | 1.744 |
| 91501040 | Gelatin dessert with fruit and whipped cream | 1.532 |  |  |
| 91501070 | Gelatin dessert with fruit and sour cream | 1.190 |  |  |
| 91501100 | Gelatin salad with vegetables | 0.513 | Gelatin salad with vegetables | 0.513 |
| 91501110 | Gelatin dessert with fruit and whipped topping | 1.554 |  |  |
| 91511020 | Gelatin dessert, dietetic, with fruit, sweetened with low calorie sweetener | 1.951 | Gelatin dessert, sugar free, with fruit | 1.951 |
| 91511070 | Gelatin dessert, dietetic, with fruit and sour cream, sweetened with low calorie sweetener | 1.286 |  |  |
| 91511080 | Gelatin dessert, dietetic, with fruit and cream cheese, sweetened with low calorie sweetener | 2.377 |  |  |
| 91511100 | Gelatin salad, dietetic, with vegetables, sweetened with low calorie sweetener | 0.577 |  |  |
| 91511110 | Gelatin dessert, dietetic, with fruit and whipped topping, sweetened with low calorie sweetener | 1.618 |  |  |
| 91520100 | Yokan | 8.462 | Yokan | 8.462 |
| 91718300 |  |  | Ladoo, round ball | 4.095 |
| 93406000 | Glug | 0.343 | Glug | 0.343 |
| 99995000 | Breading or batter as ingredient in food | 25.087 | Breading or batter as ingredient in food | 25.087 |
| 99995130 | Wheat bread as ingredient in sandwiches | 48.000 | Wheat bread as ingredient in sandwiches | 48.000 |
| 99995135 |  |  | Wheat bun as ingredient in sandwiches | 43.200 |
| 99997510 |  |  | Onions, cooked, as ingredient | 124.824 |
| 99997800 |  |  | Dark green vegetables as ingredient in omelet | 8.070 |
| 99997802 |  |  | Tomatoes as ingredient in omelet | 8.070 |
| 99997804 |  |  | Other vegetables as ingredient in omelet | 8.070 |
| 99997805 |  |  | Mirepoix, cooked, as ingredient | 40.350 |
| 99997810 |  |  | Vegetables as ingredient in curry | 20.175 |
| 99997820 |  |  | Vegetables as ingredient in stews | 8.070 |

Abbreviations: FNDDS, Food and Nutrient Database for Dietary Studies; NHANES, National Health and Nutrition Examination Survey; ISAPP, International Scientific Association for Probiotics and Prebiotics. This table presents food-code-level estimated prebiotic values used for the NHANES analyses. Only FNDDS food codes with positive assigned estimated ISAPP-recognized prebiotic values in at least one survey period are shown. Values are expressed as mg/g food. Estimated values were harmonized across survey periods when the same FNDDS food code was available in both periods. Blank cells indicate that the food code was not present in that survey period or was not assigned positive estimated prebiotic content. Food codes not listed were assigned 0 mg/g or were not assigned positive estimated prebiotic content.
