# Supplementary material for: Estimated prebiotic intake and depression in US adults: repeated cross-sectional analyses of NHANES 2015–2018 and 2021–2023
Source: J Nutr Sci. 2026 Jul 31;15:e70. doi: 10.1017/jns.2026.10136 (PMC13439025; doi:10.1017/jns.2026.10136)
Supplement: Supplementary file 4 [file S2048679026101360sup004.docx]

**Supplementary Table 4**
**Comparison of adults excluded from and included in the NHANES 2021-2023 restricted regression sample**

| **Characteristic** | **Excluded from restricted regression sample (n = 2,419)** | **Restricted regression sample (n = 1,390)** |
| --- | --- | --- |
| **Age, years** | 47.04 (0.69) | 50.31 (0.95) |
| **Sex, n (weighted %)** |  |  |
| Male | 957 (45.5) | 724 (55.8) |
| Female | 1,462 (54.5) | 666 (44.2) |
| **Race/ethnicity, n (weighted %)** |  |  |
| Mexican American | 159 (7.3) | 66 (5.6) |
| Other Hispanic | 235 (9.7) | 111 (7.8) |
| Non-Hispanic White | 1,548 (63.4) | 898 (63.2) |
| Non-Hispanic Black | 277 (11.4) | 121 (8.2) |
| Non-Hispanic Asian | 59 (3.0) | 104 (10.1) |
| Other race/multiracial | 141 (5.1) | 90 (5.1) |
| **Education, n (weighted %)** |  |  |
| Did not graduate high school | 226 (8.3) | 122 (6.2) |
| High school/GED | 492 (23.9) | 239 (22.0) |
| Some college/AA | 841 (34.5) | 340 (24.0) |
| College graduate or above | 860 (33.4) | 689 (47.8) |
| Poverty ratio | 3.09 (0.10) | 3.30 (0.13) |
| BMI, kg/m² | 32.32 (0.29) | 25.22 (0.17) |
| **BMI category, n (weighted %)** |  |  |
| Underweight | 36 (1.4) | 9 (0.6) |
| Normal weight | 351 (14.9) | 591 (42.6) |
| Overweight | 459 (21.0) | 779 (56.2) |
| Obese | 1,573 (62.7) | 11 (0.6) |
| **Total energy intake, kcal/day** | 2,039 (20) | 2,157 (42) |
| **Estimated prebiotic intake, g/day** | 5.71 (0.15) | 6.79 (0.42) |
| **Estimated prebiotic intake, g/1000 kcal** | 2.81 (0.04) | 3.15 (0.11) |
| **PHQ-9 score** | 4.11 (0.16) | 3.42 (0.15) |
| **Depression status, n (weighted %)** |  |  |
| No depression | 2,103 (87.9) | 1,258 (89.9) |
| Depression | 316 (12.1) | 132 (10.1) |
| **Sleep adequacy, n (weighted %)** |  |  |
| Inadequate sleep | 513 (22.6) | 249 (17.0) |
| Adequate sleep | 1,879 (77.4) | 1,141 (83.0) |
| **Employment status, n (weighted %)** |  |  |
| Working | 1,367 (66.8) | 695 (61.0) |
| Not working | 1,051 (33.2) | 695 (39.0) |
| **Excessive alcohol intake, n (weighted %)** |  |  |
| No | 978 (37.2) | 1,390 (100.0) |
| Yes | 1,441 (62.8) | 0 (0.0) |

Abbreviations: NHANES, National Health and Nutrition Examination Survey; SE, standard error; BMI, body mass index; GED, general educational development; AA, associate degree. Values are presented as weighted mean (SE) for continuous variables and unweighted n with weighted column percentage for categorical variables. The excluded group includes participants in the descriptive analytic sample who were not included in the restricted regression sample. The restricted regression sample excluded participants with BMI outside 18-30 kg/m², excessive alcohol intake, missing total energy intake, missing sleep adequacy, or incomplete covariate data required for the regression model. Analyses accounted for the complex NHANES survey design, including dietary sample weights, strata, and primary sampling units. P values are not shown because this table is intended to describe sample restriction rather than test primary study hypotheses.
