# Supplementary material for: Estimated prebiotic intake and depression in US adults: repeated cross-sectional analyses of NHANES 2015–2018 and 2021–2023
Source: J Nutr Sci. 2026 Jul 31;15:e70. doi: 10.1017/jns.2026.10136 (PMC13439025; doi:10.1017/jns.2026.10136)
Supplement: Supplementary file 5 [file S2048679026101360sup005.docx]

**Supplementary Table 5**
**Estimated ISAPP-recognized prebiotic intake by demographic subgroup, NHANES 2015-2018 and 2021-2023**

|  | **NHANES 2015-2018** | | | | | | | **NHANES 2021-2023** | | | | | | |
| --- | --- | --- | --- | --- | --- | --- | --- | --- | --- | --- | --- | --- | --- | --- |
| **Characteristic** | **n** | **Estimated prebiotic intake, g/day** | **SE** | **95% CI** | **Energy-adjusted intake, g/1000 kcal/day** | **SE** | **95% CI** | **n** | **Estimated prebiotic intake, g/day** | **SE** | **95% CI** | **Energy-adjusted intake, g/1000 kcal/day** | **SE** | **95% CI** |
| **Overall** | 7,408 | 6.44 | 0.08 | 6.28-6.60 | 3.14 | 0.03 | 3.07-3.20 | 3,809 | 6.09 | 0.17 | 5.71-6.46 | 2.93 | 0.04 | 2.85-3.02 |
| **Sex** |  |  |  |  |  |  |  |  |  |  |  |  |  |  |
| Male | 3,558 | 7.34 | 0.10 | 7.12-7.55 | 3.10 | 0.05 | 3.01-3.20 | 1,681 | 6.71 | 0.18 | 6.33-7.09 | 2.84 | 0.06 | 2.71-2.98 |
| Female | 3,850 | 5.59 | 0.09 | 5.41-5.77 | 3.17 | 0.03 | 3.10-3.24 | 2,128 | 5.49 | 0.21 | 5.03-5.94 | 3.02 | 0.05 | 2.91-3.13 |
| **Age group** |  |  |  |  |  |  |  |  |  |  |  |  |  |  |
| 18-29 years | 1,417 | 6.39 | 0.19 | 6.01-6.77 | 3.10 | 0.06 | 2.98-3.23 | 370 | 6.26 | 0.65 | 4.87-7.65 | 2.76 | 0.16 | 2.42-3.09 |
| 30-39 years | 1,148 | 7.02 | 0.18 | 6.66-7.38 | 3.21 | 0.07 | 3.08-3.36 | 512 | 6.31 | 0.32 | 5.64-6.99 | 3.00 | 0.11 | 2.78-3.23 |
| 40-49 years | 1,135 | 6.24 | 0.19 | 5.85-6.62 | 3.02 | 0.08 | 2.85-3.19 | 472 | 5.96 | 0.34 | 5.24-6.68 | 2.71 | 0.12 | 2.46-2.95 |
| 50-59 years | 1,185 | 6.46 | 0.17 | 6.10-6.81 | 3.11 | 0.09 | 2.93-3.29 | 598 | 6.03 | 0.16 | 5.68-6.37 | 2.98 | 0.06 | 2.85-3.11 |
| 60-69 years | 1,357 | 6.36 | 0.27 | 5.80-6.91 | 3.13 | 0.10 | 2.94-3.33 | 1,047 | 5.86 | 0.14 | 5.56-6.16 | 2.96 | 0.06 | 2.83-3.09 |
| 70-79 years | 780 | 6.20 | 0.23 | 5.73-6.67 | 3.31 | 0.09 | 3.12-3.49 | 644 | 6.11 | 0.19 | 5.69-6.52 | 3.23 | 0.11 | 2.99-3.46 |
| 80 years and older | 386 | 5.61 | 0.19 | 5.23-5.98 | 3.20 | 0.11 | 2.98-3.42 | 166 | 5.91 | 0.36 | 5.14-6.69 | 3.32 | 0.21 | 2.87-3.77 |
| **Race/ethnicity** |  |  |  |  |  |  |  |  |  |  |  |  |  |  |
| Mexican American | 1,093 | 7.00 | 0.24 | 6.50-7.50 | 3.26 | 0.07 | 3.11-3.41 | 225 | 5.76 | 0.33 | 5.05-6.46 | 2.80 | 0.13 | 2.52-3.09 |
| Other Hispanic | 770 | 6.36 | 0.26 | 5.83-6.88 | 3.06 | 0.10 | 2.86-3.26 | 346 | 5.42 | 0.34 | 4.69-6.15 | 2.85 | 0.15 | 2.54-3.16 |
| Non-Hispanic White | 2,783 | 6.50 | 0.10 | 6.29-6.71 | 3.14 | 0.04 | 3.06-3.23 | 2,446 | 6.12 | 0.14 | 5.81-6.42 | 2.91 | 0.05 | 2.80-3.02 |
| Non-Hispanic Black | 1,636 | 5.51 | 0.14 | 5.23-5.80 | 2.87 | 0.07 | 2.74-3.01 | 398 | 5.10 | 0.31 | 4.44-5.75 | 2.71 | 0.19 | 2.32-3.11 |
| Non-Hispanic Asian | 778 | 6.56 | 0.18 | 6.18-6.94 | 3.46 | 0.10 | 3.25-3.67 | 163 | 8.66 | 1.28 | 5.94-11.38 | 3.77 | 0.19 | 3.37-4.18 |
| Other race / multiracial | 348 | 6.53 | 0.27 | 5.97-7.08 | 3.13 | 0.16 | 2.80-3.45 | 231 | 6.55 | 0.86 | 4.72-8.37 | 3.04 | 0.19 | 2.64-3.45 |
| **BMI category** |  |  |  |  |  |  |  |  |  |  |  |  |  |  |
| Underweight | 106 | 5.46 | 0.42 | 4.61-6.31 | 3.01 | 0.28 | 2.42-3.59 | 45 | 6.00 | 0.85 | 4.19-7.81 | 2.85 | 0.34 | 2.14-3.57 |
| Normal weight | 1,865 | 6.49 | 0.18 | 6.13-6.86 | 3.18 | 0.05 | 3.07-3.28 | 942 | 6.70 | 0.50 | 5.62-7.77 | 3.07 | 0.12 | 2.81-3.33 |
| Overweight | 2,284 | 6.51 | 0.15 | 6.21-6.80 | 3.13 | 0.05 | 3.04-3.22 | 1,238 | 6.22 | 0.22 | 5.76-6.68 | 3.00 | 0.06 | 2.87-3.12 |
| Obese | 3,153 | 6.39 | 0.14 | 6.10-6.68 | 3.12 | 0.05 | 3.01-3.23 | 1,584 | 5.61 | 0.16 | 5.28-5.95 | 2.80 | 0.05 | 2.70-2.90 |
| **Education** |  |  |  |  |  |  |  |  |  |  |  |  |  |  |
| Did not graduate high school | 1,347 | 6.19 | 0.26 | 5.67-6.72 | 3.11 | 0.07 | 2.97-3.26 | 348 | 5.38 | 0.48 | 4.34-6.41 | 2.80 | 0.09 | 2.60-3.00 |
| High school / GED | 1,818 | 6.25 | 0.17 | 5.91-6.60 | 3.13 | 0.06 | 3.00-3.26 | 731 | 5.77 | 0.41 | 4.90-6.63 | 2.79 | 0.08 | 2.61-2.96 |
| Some college / AA | 2,378 | 6.31 | 0.12 | 6.06-6.56 | 3.04 | 0.04 | 2.96-3.13 | 1,181 | 5.87 | 0.18 | 5.50-6.25 | 2.84 | 0.07 | 2.68-2.99 |
| College graduate or above | 1,865 | 6.80 | 0.18 | 6.43-7.17 | 3.24 | 0.07 | 3.09-3.40 | 1,549 | 6.59 | 0.18 | 6.21-6.97 | 3.12 | 0.05 | 3.02-3.23 |
| **Poverty ratio** |  |  |  |  |  |  |  |  |  |  |  |  |  |  |
| 0.00-1.00 | 1,512 | 5.97 | 0.14 | 5.68-6.25 | 3.03 | 0.06 | 2.91-3.15 | 508 | 5.38 | 0.37 | 4.60-6.17 | 2.84 | 0.16 | 2.50-3.18 |
| 1.01-2.00 | 1,993 | 6.35 | 0.20 | 5.94-6.76 | 3.14 | 0.08 | 2.97-3.30 | 719 | 5.57 | 0.30 | 4.92-6.21 | 2.87 | 0.11 | 2.62-3.11 |
| 2.01-3.00 | 1,261 | 6.51 | 0.15 | 6.19-6.82 | 3.10 | 0.06 | 2.98-3.22 | 622 | 6.03 | 0.39 | 5.20-6.87 | 2.87 | 0.08 | 2.69-3.04 |
| 3.01-4.00 | 775 | 6.27 | 0.20 | 5.85-6.69 | 3.07 | 0.10 | 2.87-3.27 | 486 | 5.88 | 0.36 | 5.13-6.64 | 2.76 | 0.12 | 2.50-3.02 |
| 4.01-5.00 | 1,867 | 6.69 | 0.16 | 6.37-7.01 | 3.21 | 0.06 | 3.10-3.33 | 1,474 | 6.63 | 0.31 | 5.97-7.29 | 3.08 | 0.06 | 2.94-3.22 |

Abbreviations: NHANES, National Health and Nutrition Examination Survey; ISAPP, International Scientific Association for Probiotics and Prebiotics; SE, standard error; CI, confidence interval; BMI, body mass index. Values are presented for descriptive analytic samples. n values are unweighted counts. Means, SEs, and 95% CIs are survey-weighted estimates accounting for the complex NHANES survey design, including dietary sample weights, strata, and primary sampling units. Estimated prebiotic intake reflects the sum of estimated ISAPP-recognized prebiotics from foods and beverages reported in one or two reliable 24-hour dietary recalls. Energy-adjusted estimated prebiotic intake was calculated as estimated prebiotic intake divided by total energy intake and multiplied by 1000. NHANES 2015-2018 and NHANES 2021-2023 were analyzed as separate survey periods; no formal cross-period statistical tests are shown.
