# Supplementary material for: Estimated prebiotic intake and depression in US adults: repeated cross-sectional analyses of NHANES 2015–2018 and 2021–2023
Source: J Nutr Sci. 2026 Jul 31;15:e70. doi: 10.1017/jns.2026.10136 (PMC13439025; doi:10.1017/jns.2026.10136)
Supplement: Supplementary file 6 [file S2048679026101360sup006.docx]

**Supplementary Table 6**
**Sensitivity analyses for survey-weighted logistic regression models, NHANES 2015-2018 and 2021-2023**

| **Model** | **n** | **Exposure unit** | **Adjusted OR for depression** | **95% CI** | **P value** | **Covariates / notes** |
| --- | --- | --- | --- | --- | --- | --- |
| **NHANES 2015-2018** | | | | | | |
| **Simplified covariate model** |  |  |  |  |  |  |
| Simplified overall | 3,601 | Per 5 g/day | 0.70 | 0.50-0.99 | 0.043 | age, sex, race/ethnicity, poverty ratio, total energy |
| Simplified male | 1,843 | Per 5 g/day | 0.71 | 0.45-1.12 | 0.138 | age, race/ethnicity, poverty ratio, total energy |
| Simplified female | 1,758 | Per 5 g/day | 0.65 | 0.48-0.88 | 0.007 | age, race/ethnicity, poverty ratio, total energy |
| **Two-day-only sensitivity model** |  |  |  |  |  |  |
| Two-day-only overall | 3,601 | Per 5 g/day | 0.69 | 0.49-0.98 | 0.038 | age, sex, race/ethnicity, education, poverty ratio, employment status, sleep adequacy, total energy |
| Two-day-only male | 1,843 | Per 5 g/day | 0.69 | 0.45-1.07 | 0.098 | age, race/ethnicity, education, poverty ratio, employment status, sleep adequacy, total energy |
| Two-day-only female | 1,758 | Per 5 g/day | 0.65 | 0.45-0.94 | 0.022 | age, race/ethnicity, education, poverty ratio, employment status, sleep adequacy, total energy |
| **Energy-adjusted exposure model** |  |  |  |  |  |  |
| Energy-adjusted overall | 3,601 | Per 1 g/1000 kcal | 0.87 | 0.77-0.99 | 0.037 | age, sex, race/ethnicity, education, poverty ratio, employment status, sleep adequacy |
| Energy-adjusted male | 1,843 | Per 1 g/1000 kcal | 0.84 | 0.68-1.03 | 0.097 | age, race/ethnicity, education, poverty ratio, employment status, sleep adequacy |
| Energy-adjusted female | 1,758 | Per 1 g/1000 kcal | 0.89 | 0.78-1.02 | 0.101 | age, race/ethnicity, education, poverty ratio, employment status, sleep adequacy |
| **NHANES 2021-2023** | | | | | | |
| **Simplified covariate model** |  |  |  |  |  |  |
| Simplified overall | 1,390 | Per 5 g/day | 1.01 | 0.72-1.43 | 0.931 | age, sex, race/ethnicity, poverty ratio, total energy |
| Simplified male | 724 | Per 5 g/day | 1.08 | 0.77-1.51 | 0.635 | age, race/ethnicity, poverty ratio, total energy |
| Simplified female | 666 | Per 5 g/day | 0.86 | 0.50-1.48 | 0.565 | age, race/ethnicity, poverty ratio, total energy |
| **Two-day-only sensitivity model** |  |  |  |  |  |  |
| Two-day-only overall | 1,266 | Per 5 g/day | 1.09 | 0.74-1.62 | 0.645 | age, sex, race/ethnicity, education, poverty ratio, employment status, sleep adequacy, total energy |
| Two-day-only male | 654 | Per 5 g/day | 1.20 | 0.80-1.81 | 0.359 | age, race/ethnicity, education, poverty ratio, employment status, sleep adequacy, total energy |
| Two-day-only female | 612 | Per 5 g/day | 0.92 | 0.50-1.68 | 0.764 | age, race/ethnicity, education, poverty ratio, employment status, sleep adequacy, total energy |
| **Energy-adjusted exposure model** |  |  |  |  |  |  |
| Energy-adjusted overall | 1,390 | Per 1 g/1000 kcal | 1.01 | 0.88-1.17 | 0.859 | age, sex, race/ethnicity, education, poverty ratio, employment status, sleep adequacy |
| Energy-adjusted male | 724 | Per 1 g/1000 kcal | 1.07 | 0.92-1.26 | 0.344 | age, race/ethnicity, education, poverty ratio, employment status, sleep adequacy |
| Energy-adjusted female | 666 | Per 1 g/1000 kcal | 0.99 | 0.83-1.18 | 0.893 | age, race/ethnicity, education, poverty ratio, employment status, sleep adequacy |
| **Prescription-medication sensitivity model** |  |  |  |  |  |  |
| Exclude any prescription medication use | 415 | Per 5 g/day | 0.90 | 0.48-1.66 | 0.708 | Exploratory sensitivity; excludes any prescription medication use, not depression medication specifically. |

Abbreviations: NHANES, National Health and Nutrition Examination Survey; OR, odds ratio; CI, confidence interval. Odds ratios and 95% confidence intervals were estimated using survey-weighted logistic regression and represent the odds of depression. Estimated prebiotic intake was modeled per 5 g/day higher intake unless otherwise noted. The simplified covariate models adjusted for age, race/ethnicity, poverty ratio, and total energy intake, with sex additionally included in the overall model. The two-day-only sensitivity model was restricted to participants with two reliable dietary recalls. In the NHANES 2015-2018 analytic file, the two-day-only restriction did not further reduce the restricted regression sample. The energy-adjusted exposure model used estimated prebiotic intake in g/1000 kcal. The prescription-medication sensitivity analysis was conducted only for NHANES 2021-2023 and excluded participants reporting any prescription medication use; it was exploratory because this exclusion was not equivalent to excluding depression medication use. Analyses accounted for the complex NHANES survey design, including dietary sample weights, strata, and primary sampling units.
